# Supplementary material for: An efficient and robust laboratory workflow and tetrapod database for larger scale environmental DNA studies
Source: Gigascience. 2019 Apr 13;8(4):giz029. doi: 10.1093/gigascience/giz029 (PMC6461710; doi:10.1093/gigascience/giz029)
Supplement: GIGA-D-18-00219_Original_Submission.pdf [file giz029_giga-d-18-00219_original_submission.pdf]

GigaScience

An efficient and improved laboratory workflow and tetrapod database for larger scale eDNA studies

--Manuscript Draft--

**Manuscript Number:**	GIGA-D-18-00219
**Full Title:**	An efficient and improved laboratory workflow and tetrapod database for larger scale eDNA studies
**Article Type:**	Data Note
**Funding Information:**	German Federal Ministry of Education and Research (BMBF) (01LN1301A) — Dr. Andreas Wilting
**Abstract:**	Background<br>The use of environmental DNA, 'eDNA,' for species detection via metabarcoding is growing rapidly and now, even terrestrial mammals can be monitored via 'invertebrate-derived DNA' or 'iDNA' from hematophagous invertebrates. We present a co-designed lab workflow and bioinformatic pipeline to mitigate the two most important risks of e/iDNA: sample contamination and taxonomic mis-assignment. These risks arise from the need for amplification to detect the trace amounts of DNA and the necessity of using short target regions due to DNA degradation.<br>Findings<br>Here we present a high-throughput laboratory workflow that minimises these risks via a three-step strategy: (1) each sample is sequenced for two PCR replicates from each of two extraction replicates; (2) we use a 'twin-tagging,' two-step PCR protocol; (3) and a multi-marker approach targeting three mitochondrial loci: 12S, 16S and CytB. As a test, 1532 leeches were analysed from Sabah, Malaysian Borneo. Twin-tagging allowed us to detect and exclude chimeric sequences. The smallest DNA fragment (16S) amplified best for all samples but often at lower taxonomic resolution. We only accepted assignments that were found in both extraction replicates, totalling 174 assignments for 96 samples.<br>To avoid false taxonomic assignments, we also present an approach to create curated reference databases that can be used with the powerful taxonomic-assignment method PROTAX. For some taxonomic groups and some markers, curation resulted in over 50% of sequences being deleted from public reference databases, due mainly to: (1) limited overlap between our target amplicon and available reference sequences; (2) apparent mislabelling of reference sequences; (3) redundancy. A provided bioinformatics pipeline processes amplicons and conducts the PROTAX taxonomic assignment.<br>Conclusions<br>Our metabarcoding workflow should help research groups to increase the robustness of their results and therefore facilitate wider usage of e/iDNA, which is turning into a valuable source of ecological and conservation information on tetrapods.
**Corresponding Author:**	Jan Axtner<br>Leibniz Institute for Zoo and Wildlife Research<br>Berlin, Germany GERMANY
**Corresponding Author Secondary Information:**	
**Corresponding Author's Institution:**	Leibniz Institute for Zoo and Wildlife Research
**Corresponding Author's Secondary Institution:**	
**First Author:**	Jan Axtner
**First Author Secondary Information:**	
**Order of Authors:**	Jan Axtner<br>Alex Crampton-Platt<br>Lisa Hörig

Powered by Editorial Manager® and ProduXion Manager® from Aries Systems Corporation

	Azlan Mohamed
	Charles C.Y. Xu
	Douglas W. Yu
	Andreas Wilting
**Order of Authors Secondary Information:**	
**Additional Information:**	
**Question**	**Response**
Are you submitting this manuscript to a special series or article collection?	No
**Experimental design and statistics**<br><br>Full details of the experimental design and statistical methods used should be given in the Methods section, as detailed in our Minimum Standards Reporting Checklist. Information essential to interpreting the data presented should be made available in the figure legends.<br><br>Have you included all the information requested in your manuscript?	Yes
**Resources**<br><br>A description of all resources used, including antibodies, cell lines, animals and software tools, with enough information to allow them to be uniquely identified, should be included in the Methods section. Authors are strongly encouraged to cite Research Resource Identifiers (RRIDs) for antibodies, model organisms and tools, where possible.<br><br>Have you included the information requested as detailed in our Minimum Standards Reporting Checklist?	Yes
**Availability of data and materials**<br><br>All datasets and code on which the conclusions of the paper rely must be either included in your submission or deposited in publicly available repositories (where available and ethically appropriate), referencing such data using a unique identifier in the references and in the “Availability of Data and Materials” section of your manuscript.	Yes

Powered by Editorial Manager® and ProduXion Manager® from Aries Systems Corporation

Have you have met the above requirement as detailed in our Minimum Standards Reporting Checklist?	

Powered by Editorial Manager® and ProduXion Manager® from Aries Systems Corporation

Manuscript

Click here to download Manuscript Leeches_Methods_Database_draft_2018_06_13_v3.pdf

An efficient and improved laboratory workflow and tetrapod database for larger scale eDNA studies

Jan Axtner[1+], Alex Crampton-Platt[1], Lisa A. Hörig[1], Azlan Mohamed[1], Charles C.Y. Xu[2,3,4], Douglas W. Yu[2,5] and Andreas Wilting[1]

Affiliations:

[1] Leibniz Institute for Zoo and Wildlife Research (*Leibniz-IZW*), Alfred-Kowalke-Str. 17, 10315 Berlin, Germany

[2] State Key Laboratory of Genetic Resources and Evolution, Kunming Institute of Zoology, Chinese Academy of Sciences, 32 Jiaochang East Road, Kunming, Yunnan 650223, China

[3] Groningen Institute for Evolutionary Life Sciences, University of Groningen, P.O. Box 11103, 9700 CC Groningen, The Netherlands

[4] Redpath Museum and Department of Biology, McGill University, Montreal, QC, Canada

[5] School of Biological Sciences, University of East Anglia, Norwich Research Park, Norwich, Norfolk NR47TJ, UK

[+] corresponding author

email addresses:

Jan Axtner: axtner@izw-berlin.de
Alex Crampton-Platt: alex@naturemetrics.co.uk
Lisa A. Hörig: lisa.hoerig@arcor.de
Azlan Mohamed: mohamed@izw-berlin.de
Charles C.Y. Xu: charles.cong.xu@gmail.com
Douglas W. Yu: dougwyu@mac.com
Andreas Wilting: wilting@izw-berlin.de

Keywords:
metabarcoding, iDNA, eDNA, leeches

Abstract

Background

The use of environmental DNA, 'eDNA,' for species detection via metabarcoding is growing rapidly and now, even terrestrial mammals can be monitored via 'invertebrate-derived DNA' or 'iDNA' from hematophagous invertebrates. We present a co-designed lab workflow and bioinformatic pipeline to mitigate the two most important risks of e/iDNA: sample contamination and taxonomic mis-assignment. These risks arise from the need for amplification to detect the trace amounts of DNA and the necessity of using short target regions due to DNA degradation.

Findings

Here we present a high-throughput laboratory workflow that minimises these risks via a three-step strategy: (1) each sample is sequenced for two *PCR replicates* from each of two *extraction replicates*; (2) we use a 'twin-tagging,' two-step PCR protocol; (3) and a multi-marker approach targeting three mitochondrial loci: *12S*, *16S* and *CytB*. As a test, 1532 leeches were analysed from Sabah, Malaysian Borneo. Twin-tagging allowed us to detect and exclude chimeric sequences. The smallest DNA fragment (*16S*) amplified best for all samples but often at lower taxonomic resolution. We only accepted assignments that were found in both *extraction replicates*, totalling 174 assignments for 96 samples.

To avoid false taxonomic assignments, we also present an approach to create curated reference databases that can be used with the powerful taxonomic-assignment method *PROTAX*. For some taxonomic groups and some markers, curation resulted in over 50% of sequences being deleted from public reference databases, due mainly to: (1) limited overlap between our target amplicon and available reference sequences; (2) apparent mislabelling of reference sequences; (3) redundancy. A provided bioinformatics pipeline processes amplicons and conducts the *PROTAX* taxonomic assignment.

Conclusions

Our metabarcoding workflow should help research groups to increase the robustness of their results and therefore facilitate wider usage of e/iDNA, which is turning into a valuable source of ecological and conservation information on tetrapods.

Introduction

Monitoring, or even detecting, elusive or cryptic species in the wild can be challenging, particularly in dense vegetation or difficult terrain. In recent years there has been a rise in the availability of cost-effective DNA-based methods made possible by advances in high-throughput DNA sequencing (HTS). One such method is eDNA metabarcoding, which seeks to identify the species present in a habitat from traces of 'environmental DNA' (eDNA) in

substrates such as water, soil, or faeces. A recent variation of eDNA metabarcoding, known as 'invertebrate-derived DNA' (iDNA) metabarcoding, targets the genetic material of prey or host species extracted from copro-, sarco- or haematophagous invertebrates. Examples include ticks [1], blow or carrion flies [2, 3, 4, 5], mosquitoes [6, 7, 8, 9] and leeches [10, 11, 12,13]. Many of these parasites are ubiquitous, highly abundant, and easy to collect, making them an ideal source of biodiversity data, especially for terrestrial vertebrates that are otherwise difficult to detect [14, 15, 10]. In particular, the possibility for bulk collection and sequencing in order to screen large areas and minimise costs is attractive. However, most of the recent studies on iDNA studies focus on single-specimen DNA extracts and Sanger sequencing, and thus are not making use of the advances of HTS and a metabarcoding framework for carrying out larger scale biodiversity surveys.

That said, e/iDNA metabarcoding also poses several challenges, due to the low quality and low amounts of target DNA available, relative to non-target DNA (including the high-quality DNA of the live, invertebrate vector). In bulk iDNA samples comprised of many invertebrate specimens, this problem is further exacerbated by the variable time since each individual has fed, if at all, leading to differences in the relative amount and degradation of target DNA per specimen. This makes e/iDNA studies similar to ancient DNA samples, which also pose the problem of low quality and low amounts of target DNA [16, 17]. The great disparity in the ratio of target to non-target DNA and the low overall amount of the former requires an enrichment step, which is achieved via the amplification of a short target sequence (amplicon) by polymerase chain reaction (PCR), to obtain enough target material for sequencing. However, this enrichment step can result in false-positive species detections, either through contamination or through volatile short PCR amplicons in the laboratory, and false negative results, through primer bias and low concentrations of template DNA. Although laboratory standards to prevent and control for such false results are well established in the field of ancient DNA, there are still no best-practice guidelines for e/iDNA studies, and thus few studies sufficiently account for such problems (but see [18]).

The problem is exacerbated by the use of 'universal' primers used for the PCR, which maximise the taxonomic diversity of the amplified sequences. This makes the method a powerful biodiversity assessment tool, even where little is known *a priori* about which species might be found. However, using such primers, in combination with low quality and quantity of target DNA, which often requires a high number of PCR cycles to generate enough amplicon products for sequencing, makes metabarcoding studies particularly vulnerable to false-results [13, 19; 20]. The high number of PCR cycles, combined with the high sequencing depth of HTS, also increase the likelihood that contaminants are amplified and detected, possibly to the same or greater extent as some true-positive trace DNA. As e/iDNA have been proposed as tools to detect very rare and priority conservation species such as the Saola, *Pseudoryx nghetinhensis* [10], false detection might result in misguided conservation activities worth several hundreds of thousands of US dollars e.g. [21]. Therefore, similar to ancient DNA studies, great care must be taken to minimise the

possibility for cross-contamination in the laboratory and to maximise the correct detection of species through proper experimental design. Replication in particular is an important tool for reducing the incidence of false negatives and detection of false positives but the trade-off is increased cost, workload, and analytical complexity [19].

A second source of false-positive species detections is the incorrect assignment of taxonomies to the millions of short HTS reads generated by metabarcoding. Although there has been a proliferation of tools focused on this step, most can be categorised into just three groups depending on whether the algorithm utilises sequence similarity searches, sequence composition models, or phylogenetic methods [22, 23, 24]. The one commonality among all methods is the need for a reliable reference database of correctly identified sequences, yet there are few curated databases currently appropriate for use in e/iDNA metabarcoding. Two exceptions are SILVA [25] for the nuclear markers SSU and LSU rRNA used in microbial ecology, and BOLD (Barcode of Life Database; citation) for the COI 'DNA barcode' region. For other loci, a non-curated database downloaded from the INSDC (International Nucleotide Sequence Database Collaboration, e.g. GenBank) is generally used. However, the INSDC places the burden for metadata accuracy, including taxonomy, on the sequence submitters, with no restriction on sequence quality or veracity. For instance, specimen identification is often carried out by non-specialists, which increases error rates, and common laboratory contaminant species (e.g. human DNA sequences) are submitted in lieu of the sample itself. The rate of sequence mislabelling has not been assessed for GenBank, but for several curated microbial databases (Greengenes, LTP, RDP, SILVA), mislabelling rates have been estimated at between 0.2% and 2.5% [26]. It is likely that the true proportion of mislabelled samples in GenBank is higher than this given the lack of professional curation. Moreover, correctly identifying such errors is labour-intensive, so most metabarcoding studies simply base their taxonomic assignments on sequence-similarity searches of the whole INSDC database (e.g. with BLAST) [3, 10, 12] and thus can only detect errors if assignments are ecologically unlikely. Furthermore, reference sequences for the species that are likely to be sampled in iDNA studies are often underrepresented in or absent from these databases, which increases the possibility of incorrect assignment. For instance, fewer than 50% of species occurring in a tropical megadiverse rainforest are represented in Genbank (see findings below). When species-level matches are ambiguous, it might still be possible to assign a sequence to a higher taxonomic rank by using an appropriate algorithm such as MEGAN's Lowest Common Ancestor [27] or *PROTAX* [28].

We present here a complete laboratory workflow and complementary bioinformatics pipeline, starting from DNA extraction to taxonomic assignment of HTS reads using a curated reference database. The laboratory workflow allows for efficient screening of hundreds of e/iDNA samples: (1) two *extraction replicates* are separated during DNA extraction, and each is sequenced in two *PCR replicates* (Fig. 1); (2) a 'twin-tagged', two-step PCR protocol prevents cross-sample contamination as no unlabelled PCR products are

produced (Fig. 2); (3) robustness of the taxonomic assignment is improved by using up to three mitochondrial markers. Our bioinformatics pipeline includes a standardized, automated, and replicable approach to create a curated database, which allows updating as new reference sequences become available, and to be expanded to other amplicons with minimal additional effort. We also provide scripts for processing the raw data to quality-controlled dereplicated reads and for taxonomic assignment of these reads using *PROTAX* [28], a probabilistic method that has been shown to be robust even when reference databases are incomplete [23, 4] (all scripts are available from URL https://github.com/alexcrampton-platt/screenforbio-mbc).

Methods

iDNA samples

We used 242 collections of haematophagous terrestrial leeches stored in RNA*Later* (Sigma-Aldrich, Munich -Germany) from Deramakot Forest Reserve in Sabah, Malaysian Borneo as samples. Each sample consisted of one to 77 leech specimens (median 4). In total, 1532 leeches were collected, exported under the permit (JKM/MBS.1000-2/3 JLD.2 (8) issued by the Sabah Biodiversity Council), and analysed at the laboratories of the Leibniz-IZW.

Laboratory workflow

The laboratory workflow is designed to both minimize the risk of sample cross-contamination and to aid identification of any instances that do occur. All laboratory steps (extraction, pre and post PCR steps, sequencing) took place in separate laboratories and no samples or materials were allowed to re-enter upstream laboratories at any point in the workflow. All sample handling was carried out under specific hoods that were wiped with bleach, sterilized, and UV irradiated for 30 minutes after each use. All labs are further UV irradiated for four hours each night.

DNA extraction

DNA was extracted from each sample in bulk. Leeches were cut into small pieces with a fresh scalpel blade and incubated in lysate buffer (proteinase K and ATL buffer at a ratio of 1:10; 0.2 ml per leech) overnight at 55 °C (12 hours minimum) in an appropriately sized vessel for the number of leeches (2 or 5 ml reaction tube). For samples with more than 35 leeches, the reaction volume was split in two and recombined after lysis.

Each lysate was split into two *extraction replicates* (A and B; maximum volume 600 µl) and all further steps were applied to these independently. We followed the DNeasy 96 Blood & Tissue protocol for animal tissues (Qiagen, Hilden -Germany) on 96 plates for clean-up. DNA was eluted twice with 100 µl TE buffer. DNA concentration was measured with PicoGreen dsDNA Assay Kit (Quant-iT, ThermoFisherScientific, Waltham -USA) in 384-well plate format using an appropriate plate reader (200 PRO NanoQuant, Tecan Trading AG, Männedorf -Switzerland). Finally, all samples were diluted to a maximum concentration of 10 ng/µl.

Shot-gun sequencing to quantify mammalian DNA content

To estimate the proportion of mammalian DNA in the leech samples, we ran a 75-cycle paired-end, shot-gun sequencing on an Illumina MiSeq on a subset of 58 samples. We used BLAST to compare the reads to GenBank and used MEGAN to find the lowest common ancestor for each read.

PCR

Two-round PCR protocol. – We amplified three mitochondrial markers – a short 93 bp fragment of *16S* rRNA (*16S*), a 389 bp fragment of *12S* rRNA (*12S*), and a 302 bp fragment of cytochrome b (*CytB*). For each marker, we ran a two-round PCR protocol (Figs. 1, 2). The first round amplified the target gene. The second round added the Illumina adapters for sequencing.

Primer design. – We used 'twin-tagged' PCR primers, meaning that *both* the forward and reverse primers were given the *same* sample-identifying sequence (i.e. 'tags') added as primer extensions (Fig. 2). This ensured that unlabelled PCR products were never produced and allowed us later to detect and delete tag jumping events [29] (Fig. 2). Primer sequences are in Table 1 [30, 31].

In the first PCR round, we used 25 different 5-bp *sample*-identifying tags (*tag 1*), with a minimum pairwise distance of three (Faircloth et al, 2012; Supplement Table 1). These primers also contained different forward and reverse sequences (*Read 1* & *Read 2 sequence primers*) (Supplement table 1) to act priming sites for the second PCR round (Fig. 2).

In the second PCR round, we used 20 different 5-bp *plate*-identifying tags (*tag 2*), with a minimum pairwise distance of three [32]. These primers also contained the Illumina P5 and P7 adapter sequences (Fig. 2). The product of the second PCR round could thus be cleaned up, quantified, pooled, and sequenced without needing to carry out a separate library preparation step (e.g. Nextera, TruSeq).

Cycle number considerations. – Because we know that our target DNA is at low concentration in the samples, we are faced with a trade-off between (1) using fewer PCR cycles (e.g. 30 cycles) to minimise amplification bias (caused by some target DNA binding better to the primer sequences and thus outcompeting during PCR other target sequences that bind less well, [33]) and (2) using more PCR cycles (e.g. 40 cycles) to ensure that low-concentration target DNA is sufficiently amplified in the first place. Rather than choose between these two extremes, we ran both low- and a high-cycle protocols and sequenced both sets of amplicons.

Thus, each of the two *extraction replicates* A and B was split and amplified using different cycle numbers (*PCR replicates* 1 and 2) for a total of four (= 2 *extraction replicates* X 2 *PCR replicates* -> *A1*/*A2* and *B1*/*B2*) replicates per sample per marker (Fig. 1). For *PCR replicates* A1/B1, we used 30 cycles in the first PCR round to minimize the effect of amplification bias. For *PCR replicates* A2/B2, we used 40 cycles in the first PCR round to increase the likelihood of detecting species with very low input DNA (Fig. 1).

PCR protocol. – The first-round PCR reaction volume was 20 µl, including 0.1 µM primer mix, 0.2 mM dNTPs, 1.5 mM $MgCl_2$, 1x PCR buffer, 0.5 U AmpliTaq Gold™ (Invitrogen, Karlsruhe - Germany), and 2 µl of template DNA. Initial denaturation was 5 minutes at 95°C, followed by repeated cycles of 30 seconds at 95°C, 30 seconds at 54°C, and 45 seconds at 72°C. Final elongation was 5 minutes at 72°C. Samples were amplified in batches of 24 plus a negative (water) and a positive control (bank vole, *Myodes glareolus* DNA). All three markers were amplified simultaneously for each batch of samples in a single PCR plate. Non-target by-products were removed as required from some *12S* PCRs by purification with magnetic Agencourt AMPure beads (Beckman Coulter, Krefeld -Germany).

In the second-round PCR, we used the same PCR protocol as above with 2 µl of the product of the first-round PCR and 10 PCR cycles.

Quality control and sequencing

Amplification was visually verified after the second-round PCR by gel electrophoresis on 1.5% agarose gels. Controls were additionally checked with a TapeStation 2200 (D1000 ScreenTape assay, Agilent, Waldbronn -Germany). All samples were purified with AMPure beads, using a beads-to-template ratio of 0.7:1 for *12S* and *CytB* products, and a ratio of 1:1 for *16S* products. DNA concentration was measured with PicoGreen dsDNA as described above. Sequencing libraries were made for each PCR plate by equimolar pooling of all positive samples; final concentrations were between 2 and 4 nmol. Generally, *12S* and *CytB* products were combined in a single library, whereas *16S* products were always separate, because of the difference in amplicon length. Up to 11 libraries were sequenced on each run of Illumina MiSeq following standard protocols. Libraries were sequenced with MiSeq Reagent Kit V3 (600 cycles, 300 bp paired-end reads) and had a final concentration of 11 pM spiked with 20 to 30% of PhiX control.

Establishment of the tetrapod reference database

Reference database

A custom bash script was written to generate a tetrapod reference database for each of the three markers, and additionally for a 250 bp mitochondrial cytochrome *c* oxidase subunit I amplicon (*COI*), which has previously been used in iDNA studies [2]. An important time-saving step was the use of the FASTA-formatted MIDORI mitochondrial databases [34]. The script updated the FASTA files for a subset of target species, removed errors and redundancy, and output FASTA files with species names and GenBank accessions in the headers. The script accepts four data inputs, two of which are optional. The required inputs are: (i) the MIDORI sequences (December 2015 'UNIQUE', downloaded from http://www.reference-midori.info/download.php#) for the relevant genes and (ii) an initial reference taxonomy. This taxonomy is needed to find or generate a full taxonomic classification for each sequence. Here we used the Integrated Taxonomic Information System (ITIS) classification for Tetrapoda, obtained with the R package *taxize* version 0.9.0 [35], functions *downstream* and *classification*). The optional inputs are: (iii) supplementary FASTA files of reference sequences that should be added to the database, and (iv) a list of

target species to be queried on GenBank to capture any sequences published since the MIDORI set was generated. For this study, 72 recently published [36] and 7 unpublished partial mitochondrial mammal genomes (Accession Numbers MH464789, MH464790, MH464791, MH464792, MH464793, MH464794, MH464795, MH464796, MH464797, MH464798, MH464799, MH464800, MH464801) were added as input (iii). A list of 103 mammal species known to be present in the sampling area was added as input (iv).

With the above inputs, the seven curation steps are: 1) remove sequences not identified to species; 2) add any extra sequences from optional inputs (iii) and (iv) above; 3) select the target amplicon; 4) remove sequences with ambiguities; 5) compare species labels to the reference taxonomy from input (ii) and create a consensus taxonomy including any species known only from sequence data if genus already exists in reference; 6) identify and remove putatively mislabelled sequences; 7) discard redundant sequences, retaining one representative per haplotype per species.

The script is split into four modules, allowing optional manual curation at three key steps. The steps covered by each of the four modules are summarized in Table 2. The main programs used are highlighted and cited in the text where relevant, but many intermediate steps used common UNIX tools and unpublished lightweight utilities freely available from GitHub (Table 3).

Module 1 - The first step is to select the tetrapod sequences from the MIDORI database for each of the four selected loci (input (i) above). This, and the subsequent step to discard sequences without strict binomial species names and reduce subspecies identifications to species-level, are made possible by the inclusion of the full NCBI taxonomic classification of each sequence in the FASTA header by the MIDORI pipeline. The headers of the retained sequences are then reformatted to include just the species name and GenBank accession separated by underscores. If desired, additional sequences from local FASTA files are then added to the MIDORI set (input (iii)). The headers of these FASTA files are required to be in the same format. Next, optional queries are made to the NCBI GenBank and RefSeq databases for each species in a provided list (input (iv)) for each of the four target loci, using NCBI's Entrez Direct [37]. Matching sequences are downloaded in FASTA format, sequences prefixed as "UNVERIFIED" are discarded, the headers are simplified as previously, and those sequences not already in the MIDORI set are added. The region of each sequence matching to the relevant target marker was extracted with a two-step process in which *usearch* (-*search_pcr*) was used to select sequences where both primers were present, and these were in turn used as a reference to select partially matching sequences with *blastn* [38, 39]. Sequences with a hit length of at least 90% of the expected marker length were retained by extracting the relevant subsequence based on the BLAST hit co-ordinates. Sequences with ambiguous bases were discarded at this stage. In the final step in module 1 a multiple-sequence alignment was generated with MAFFT [40, 41] for each partially curated amplicon dataset. The script then breaks to allow the user to check for any obviously problematic sequences that should be discarded before continuing.

Module 2 - The species labels of the edited alignments are compared with the reference taxonomy (input (ii)). Any species not found is queried against the Catalogue of Life database (CoL) via *taxize* in case these are known synonyms, and their correct species label and classification is added to the reference taxonomy. The original species label is retained as a key to facilitate sequence renaming, and a note is added to indicate its status as a synonym. Finally, the genus name of any species not found in the CoL is searched against the consensus taxonomy, and if found, the novel species is added by taking the higher classification levels from of the other species in the genus. Orphan species labels are printed to a text file, and the script breaks to allow the user to check this list and manually create classifications for some or all if appropriate.

Module 3 - This module begins by checking for any manually generated classification files (from the end of Module 2) and merging them with the reference taxonomy from Module 2. Any remaining sequences with unverifiable classifications are removed at this step. The next steps convert the sequences and taxonomy file to the correct formats for SATIVA [26]. Sequence headers in the edited MAFFT alignments are reformatted to include only the GenBank accession and a taxonomy key file is generated with the correct classification listed for each accession number. In cases where the original species label was found to be a synonym, the corrected label is used. Putatively mislabelled sequences in each amplicon are then detected with SATIVA, and the script breaks to allow inspection of the results. The user may choose to make appropriate edits to the taxonomy key file or list of putative mislabels at this point.

Module 4 - Any sequences that are still flagged as mislabelled at the start of the fourth module are deleted from the SATIVA input alignments, and all remaining sequences are relabelled with the correct species name and accession. A final consensus taxonomy file is generated in the format required by *PROTAX*. Alignments are subsequently unaligned prior to species-by-species selection of a single representative per unique haplotype. Sequences that are the only representative of a species are automatically added to the final database. Otherwise, all sequences for each species are extracted in turn, aligned with MAFFT, and collapsed to unique haplotypes with *collapsetypes_4.6.pl* (zero differences allowed; [42]). Representative sequences are then unaligned and added to the final database.

Bioinformatics workflow

Read processing

Although the curation of the reference databases is our main focus, it is just one part of the bioinformatics workflow for e/iDNA metabarcoding. A custom bash script was used to process raw basecall files to demultiplexed, cleaned, and dereplicated reads in FASTQ format on a run-by-run basis. All runs and amplicons were processed with the same settings unless otherwise indicated. *bcl2fastq* (Illumina) was used to convert basecall files to demultiplexed, paired-end FASTQ files for each library, allowing up to 1 mismatch in each *tag 2.* Each library was further demultiplexed into samples via unique *tag 1* pairs with *AdapterRemoval* (Schubert, Lindgreen and Orlando 2016), again allowing up to 1 mismatch

in each tag. These steps allowed reads to be assigned to the correct samples via their four tags e.g. ABBA, ADDA, BDDB.

In all cases, amplicons were short enough to expect paired reads to overlap. Pairs were merged with *usearch* (*-fastq_mergepairs*; [43; 44]), and only successfully merged pairs were retained. Primer sequences were trimmed with *cutadapt* [45], and only successfully trimmed reads at least 90% of expected amplicon length were passed to a quality filtering step with *usearch* (*-fastq_filter*). Lastly, reads were dereplicated with *usearch* (*-derep_fulllength*) to retain only unique sequences, and singletons were discarded. The number of replicates that each unique sequence represented was also added to the read header at this step (option *-sizeout*).

Taxonomic assignment

The curated reference sequences and associated taxonomy were used for taxonomic classification of dereplicated reads using *PROTAX*, a recently published probabilistic method [28, 24]. *PROTAX* gives unbiased estimates of placement probability for each read at each taxonomic rank, allowing some assignments to be made to a higher rank even when there is a high degree of uncertainty at the species level. In other words, and unlike other taxonomic assignment methods, *PROTAX* can estimate the probability that a sequence belongs to a taxon that is not included in the reference database. This was considered an important feature due to the expected incompleteness of the reference databases for tetrapods in the sampled location. As other studies have compared *PROTAX* with more established methods, e.g. MEGAN [27] (see [28, 4]), it was beyond the scope of this study to evaluate the performance of *PROTAX.*

Classification with *PROTAX* is a two-step process. Firstly, PROTAX selected a subset of the reference database that was used as training data to parameterise a *PROTAX* model for each marker, and secondly, the fitted models were used to assign four taxonomic ranks (species, genus, family, order) to each of the dereplicated reads, along with a probability estimate at each level. We also included the best similarity score of the assigned species or genus, mined from the LAST results (see below) for each read. This was helpful for flagging problematic assignments for downstream manual inspection, i.e. high probability assignments based on low similarity scores (implying that there are no better matches available) and low probability assignments based on high similarity scores (indicates conflicting database signal from several species with highly similar sequences).

Fitting the *PROTAX* model followed Somervuo et al. [24] except that 5000 training sequences were randomly selected for each target marker due to the large size of the reference database. In each case, 4500 training sequences represented a mix of known species with reference sequences (conspecific sequences retained in the database) and known species without reference sequences (conspecific sequences omitted, simulating species missing from the database), and 500 sequences represented previously unknown lineages distributed evenly across the four taxonomic levels (i.e. mimicked a mix of completely novel species, genera, families and orders). Pairwise sequence similarities of

queries and references were calculated with LAST [46] following the approach of Somervuo et al. [24]. The models were weighted towards the Bornean mammals expected in the sampled area by assigning a prior probability of 90% to these 103 species and a 10% probability to all others ([24]; Supplement table 2). In cases of missing interspecific variation this helped to avoid unlikely assignments, especially in case of the very short 93 bp fragment of *16S*. Maximum *a posteriori* (MAP) parameter estimates were obtained following the approach of Somervuo et al. [28], but the models were parameterised for each of the four taxonomic levels independently, with a total of five parameters at each level (four regression coefficients and the probability of mislabelling).

Dereplicated reads for each sample were then classified using a custom bash script on a run-by-run basis. For each sample, reads in FASTQ format were converted to FASTA, and pairwise similarities were calculated against the full reference sequence database for the applicable marker with LAST. Assignments of each read to a taxonomic node based on these sequence similarities were made using a Perl script and the trained model for that level. The taxonomy of each node assignment was added with a second Perl script for a final table including the node assignment, probability, taxonomic level, and taxonomic path for each read. Read count information was included directly in the classification output via the size annotation added to the read headers during dereplication. All Perl scripts to convert input files into the formats expected by *PROTAX*, *R* code for training the model following Somervuo et al. [24], and Perl scripts for taxonomic assignment were provided by P. Somervuo (personal communication).

Acceptance criteria

In total we had twelve PCR reactions per sample: two *extraction replicates A* and *B* X two *PCR replicates* 1 and 2 per extraction replication X the three markers (Fig. 1). We only accepted taxonomic assignments that were positively detected in both *extraction replicates* (*A* & *B*, Figure 3). The reason for conservatively omitting assignments that appeared in only one extraction replicate was to rule out sample cross-contamination during DNA extraction. In addition, we only accepted assignments with ten or more reads per marker, if only one marker was sequenced. If a species was assigned in more than one marker (e.g. *12S* and *16S*), we accepted the assignment even if in one sequencing run the number of reads was below ten.

Due to the imperfect PCR amplification of markers (the small *16S* fragment amplified better than the longer *CytB* fragment) and missing reference sequences in the database or shared sequence motifs between species, reads sometimes were assigned to species level for one marker but only to genus level for another marker. Thus, the final identification of species could not be automated and manual inspection and curation was needed. For each assignment, three parameters were taken into consideration: number of sequencing reads, the mean probability estimate derived from *PROTAX*, and the mean sequence similarity to the reference sequences based on LAST.

Findings & Discussion

Database curation

The MIDORI UNIQUE database (December 2015 version) contains 1,019,391 sequences across the four mitochondrial loci of interest (*12S*: 66,937; *16S*: 146,164; *CytB*: 223,247; *COI*: 583,043), covering all Metazoa. Of these, 258,225 (25.3%) derive from the four tetrapod classes (Amphibia: 55,254; Aves: 51,096; Mammalia: 101,106; Reptilia: 50,769). The distribution of these sequences between classes and loci, and the losses at each curation step are shown in Figure 4. In three of the four classes, there is a clear bias towards *CytB* sequences, with over 50% of sequences derived from this locus. In both Aves and Mammalia, the *16S* and *12S* loci are severely underrepresented at less than 10% each, while for Reptilia, *COI* is the least sequenced locus in the database.

The numbers of sequences and rates of loss due to our curation steps varied among taxonomic classes and the four loci, although losses were observed between steps in almost all instances. The most significant losses followed amplicon selection and removal of non-unique sequences. Amplicon selection led to especially high losses in Amphibia and *16S*, indicating that data published on GenBank for this class and marker do not generally overlap with the primer sets used here. Meanwhile, the high level of redundancy in public databases was highlighted by the significant reduction in the number of sequences during the final step of removing redundant sequeces – in all cases over 10% of sequences were discarded, but some losses exceeded 50% (Mammalia: *COI*, *CytB*, *16S*; Amphibia: *16S*).

Data loss due to apparent mislabelling ranged between 1.9% and 7.4% and was thus generally higher than similar estimates for curated microbial databases [26]. SATIVA flags potential mislabels and suggests an alternative label supported by the phylogenetic placement of the sequences, allowing the user to make an appropriate decision on a case by case basis. The pipeline pauses after this step to allow such manual inspection to take place. However, for the current database, the number of sequences flagged was large (4378 in total), and the required taxonomic expertise was lacking, so all flagged sequences from non-target species were discarded to be conservative. The majority of mislabels were identified at species level (3053), but there were also significant numbers at genus (788), family (364) and order (102) level. Two to three sequences from Bornean mammal species were unflagged in each amplicon to retain the sequences in the database. This was important as in each case these were the only reference sequences available for the species. Additionally, *Muntiacus vaginalis* sequences that were automatically synonymised to *M. muntjak* based on the available information in the Catalogue of Life were revised back to their original identifications to reflect current taxonomic knowledge.

Database composition

The final database was skewed even more strongly towards *CytB* than was the raw database. It was the most abundant locus for each class and representing over 60% of sequences for both Mammalia and Reptilia. In all classes, *16S* made up less than 10% of the final database, with Reptilia *COI* also at less than 10%.

Figure 5 (frequency distributions) shows that most species represented in the curated database for any locus have just one unique haplotype against which HTS reads can be compared, while a few species have many haplotypes. The prevalence of species with 20 or more haplotypes is particularly notable in *CytB* where the four classes have between 25 (Aves) and 265 (Mammalia) species in this category. Figure 5 (coloured circles in each plot) also shows, that the species in the taxonomy are incompletely sampled across all loci, but coverage varies significantly between categories. In spite of global initiatives to generate *COI* sequences [47], this marker does not offer the best species-level coverage in any class and is a poor choice for Amphibia and Reptilia (<15% of species included). Even the best performing marker, *CytB*, is not a universally appropriate choice, as Amphibia is better covered by *12S*. These differences in underlying database composition will impact the likelihood of obtaining accurate taxonomic assignment for any one species from any single marker. Further barcoding campaigns are clearly needed to fill gaps in all markers and all classes to increase the power of future e/iDNA studies. As the costs of HTS decrease, we expect that such gap-filling will increasingly shift towards whole mitochondrial genomes [36], reducing the effect of marker choice on detection likelihood. In the meantime, however, the total number of species covered by the database can be increased by combining multiple loci (here, up to four) and thus the impacts of database gaps on correctly detecting species can be minimized ([48]; Fig. 6).

In the present study, the primary target for iDNA sampling was the mammal fauna of Malaysian Borneo, and the 103 species expected in the sampling area represent an informative case study highlighting the deficiencies in existing databases (Fig. 6). Nine species are completely unrepresented while only slightly over half (554 species) have at least one sequence for all of the loci. Individually, each marker covers over half of the target species, but none achieves more than 85% coverage (*12S*: 75 species; *16S*: 68; *CytB*: 88; *COI*: 66). Equally striking is the lack of within-species diversity, as most of the incorporated species are represented by only a single haplotype per locus. Some of the species have large distribution ranges, so it is likely that in some cases the populations on Borneo differ genetically from the available reference sequences, possibly limiting assignment success. Only a few expected species have been sequenced extensively, and most are of economic importance to humans (e.g. *Bos taurus*, *Bubalus bubalis*, *Macaca* spp, *Paradoxurus hermaphroditus*, *Rattus* spp, *Sus scrofa*), with as many as 100 haplotypes available (*Canis lupus*). Other well-represented species (≥20 haplotypes) present in the sampling area include several Muridae (*Chiropodomys gliroides*, *Leopoldamys sabanus*, *Maxomys surifer*, *Maxomys whiteheadi*) and leopard cat (*Prionailurus bengalensis*).

Laboratory workflow

Shotgun sequencing of a subset of our samples revealed that the median mammalian DNA content was only 0.9%, ranging from 0% to 98%. These estimates are approximate, but with more than 75% of the samples being below 5%, this shows clearly the scarcity of target DNA in bulk iDNA samples. The generally low DNA content and the fact that the target DNA is

often degraded make enrichment of the target barcoding loci necessary. We used PCR with high cycle numbers to obtain enough DNA for sequencing. However, this second step increases the risk of PCR error: artificial sequence variation, non-target amplification, and/or raising contaminations up to a detectable level.

We addressed these problems by running two *extraction replicates*, two *PCR replicates*, and a multi-marker approach. The need for *PCR replicates* has been acknowledged and addressed extensively in ancient DNA studies [16] and has also been highlighted for metabarcoding studies [18, 19, 20, 49]. Despite this, many e/iDNA studies do not carry out multiple *PCR replicates* to detect and omit potential false sequences. In addition, *extraction replicates* are seldom applied, despite the evidence that cross-sample DNA contamination can occur during DNA extraction [50, 51, 52]. Here we only accepted sequences that appeared in a minimum of two independent PCRs, one from each *extraction replicate A* and *B* (Fig. 1).

We also used three different loci to correct for potential PCR-amplification biases. We were, however, unable to quantify this bias in this study due to the high degradation of the target mammalian DNA, which resulted in much higher overall amplification rates for *16S*, the shortest of our PCR amplicons. For *16S*, 85% of the samples amplified, whereas for *CytB* and *12S*, only 57% and 44% amplified, respectively. Despite the greater taxonomic resolution of the longer *12S* and *CytB* fragments, our poorer amplification results for these longer fragments emphasize that e/iDNA studies should generally focus on short PCR fragments to increase the likelihood of positive amplifications of the degraded target DNA. In the case of mammal-focussed e/iDNA studies, a shorter (100 bp) *CytB* fragment will likely be very useful.

Our second major precaution was the use of twin-tagging for both PCRs (Fig. 2). This ensures that unlabelled PCR products are never produced and allows us to multiplex a large number of samples on a single run of Illumina MiSeq run. Just 24 sample *tags 1* and 20 plate *tags 2* allow the differentiation of up to 480 samples. This greatly reduced sequencing and primer purchase costs while also largely eliminating sample-misassignment via tag jumping, because tag jump sequences have non-matching forward and reverse *tag 1* sequences [29]. For our sequenced PCR plates, the rate of correct matching *tag 2* tags was 96%. We estimated the rate of tag jumps producing chimeric *tag 1* sequences to be of 1 to 5 % and these were removed from the dataset (Table 4). Twin-tagging increases costs because of the need to purchase a larger number of primer pairs. However, the risk of reporting false positives should compensate this, especially when it comes to rare or threated species.

For the second PCR round, we used the same tag pair *tag 2* for all 24 samples of a PCR plate. In order to reduce cost we tested pooling these 24 samples prior to the second PCR round, but we detected a very high tag jumping rate of over 40% (Table 4), which ultimately would increase cost through reduced sequencing efficiency.

Tagging primers in the first PCR reduces the risk of cross-contamination via aerosolised PCR products. Previous studies have shown that unlabelled volatile PCR products pose a great risk of false detections [53], a risk that is greatly increased if a high number of samples are analysed in the laboratories [13]. Also, in laboratories where other research projects are conducted, this approach allows the detection of cross-experiment contamination. Therefore, we see a clear advantage of our approach over ligation techniques when it comes to producing sequencing libraries, as the Illumina tags are only added after the first PCR, and thus the risk of cross contamination with unlabelled PCR amplicons is very low.

Assignment results

A robust assignment of species is an important factor in metabarcoding as an incorrect identification might result incorrect management interventions. The reliability of taxonomic assignments is expected to vary with respect to both marker choice and database completeness, and this is reflected in the probability estimates provided by *PROTAX*. In a recent study, less than 10% of the mammal assignments made at species level against a worldwide reference database were considered reliable with the short 16S amplicon, but this increased to 46% with full-length 16S sequences [24]. In contrast, in the same study over 80% of insect assignments at species level were considered reliable with a more complete, geographically restricted database of full-length COI barcodes. A similar pattern was observed in our data during manual curation of the assignment results – there was more ambiguity in the results for the short *16S* amplicon than for other markers. However, due to the limited amount of often degraded target DNA in e/iDNA samples, short amplicons amplify much better. In our case, this had the drawback that some species lacked any interspecific variation, and thus sequencing reads shared 99%-100% identity for several species. For example, our only *16S* reference of *Sus barbatus* was 100% identical to *S. scrofa*. But as latter species does not occur in the studied area we could assign all reads manually to *S. barbatus*. In several cases we were able to confirm *S. barbatus* by additional *CytB* results, highlighting the advantage of using multiple markers. Another important advantage of multiple markers is the opportunity to fill gaps in the reference database. For example, we lacked *16S* reference sequences for *Hystrix brachyura*, and reads were assigned by *PROTAX* only to the genus level: *Hystrix sp.*. In one sample, however, almost 5000 *CytB* reads were assigned to *Hystrix brachyura* and thus we used the *Hystrix sp*. 16S sequences in the same sample to build a consensus *16S* reference sequence for *Hystrix brachyura* for future analyses. We also inferred that PCR and sequencing errors resulted in reads being assigned to sister taxa. We observed that a high number of reads of a true sequence were assigned to a species and a lower number of noise sequences were assigned to a sister taxa. Such a pattern was observed for ungulates, especially deer that showed little variance in *16S*. It is hard to identify and control for such pattern automatically, and it highlights the importance of visual inspection of the results.

In total, we accepted 174 vertebrate detections (i.e. having positive detections in both *extraction replicates A* and *B*) within 96 bulk samples. 48% of these assignments were

present in all four *A1*, *A2*, *B1* and *B2*. 35% were present in at least three of replicates (e.g. A1, A2, B1). Although the true occurrence of species within our leeches was unknown, by accepting only positive *AB* assignment results, we increase the confidence of species detection, even if the total number of reads for that species was low. In almost all cases, however, the number of reads was high (median= 52,386; mean= 300,996; SD= 326,883). Keeping this in mind we do not believe that raw read numbers are the most reliable indicators of tetrapod DNA quantity in iDNA samples. PCR stochasticity, primer biases, multiple species in individual samples, and pooling of samples exert too many uncertainties that could bias the sequencing results. Replication of detection is inherently more reliable. In contrast to our expectation that higher cycle number might be necessary to amplify even the lowest amounts of target DNA, our data does not support this hypothesis. Although we observed an increase in positive PCRs for A2/B2 (the 40-cycle PCR replicates), the total number of accepted assignments in *A1/B1* and *A2/B2* samples did not differ. This indicates first that high PCR cycle numbers mainly increased the risk of false positives and second that our multiple precautions successfully minimized the acceptance of false detections.

Conclusion

Metabarcoding of e/iDNA samples will certainly become a very valuable tool in assessing biodiversity, as it allows to detect species non-invasively without the need to capture and handle the animals [54]. However, the technical and analytical challenges linked to sample types (low quantity and quality DNA) and poor reference databases have so far been insufficiently recognized. In contrast to ancient DNA studies where standardized laboratory procedures and specialized bioinformatics pipelines have been established and are followed in most cases, there is limited methodological consensus in e/iDNA studies, which reduces rigour. In this study, we present a robust metabarcoding workflow for e/iDNA studies. We hope that the provided scripts and protocols facilitate further development of rigour in this field. The use of e/iDNA metabarcoding to study the rarest and most endangered species such as the saola is exciting, but geneticists bear the heavy responsibility of providing correct answers to conservationists.

Acknowledgements

All authors thank the German Federal Ministry of Education and Research (BMBF FKZ: 01LN1301A) and the Leibniz-IZW for funding this study. We also thank the Sabah Forestry Department, especially Johnny Kissing, Peter Lagan and Datuk Sam Mannan for supporting the fieldwork and the Sabah Biodiversity Council for providing research, collction and export permits for this work. We are grateful to John Mathai, Seth Timothy Wong for conducting the field work and collecting the leeches. The laboratory analysis was supported by Sebastian Wieser.

References

[1] Gariepy TD, Lindsay R, Odgen N, Greory TR. Identifying the last supper: utility of the DNA barcode library for bloodmeal identification in ticks. Mol Ecol Res. 2012; 12: 646-52; doi: 10.1111/j.1755-0998.2012.03140.x

[2] Lee P-S, Gan HM, Clements GR, Wilson J-J. Field calibration of blowfly-derived DNA against traditional methods for assessing mammal diversity in tropical forests. Genome 2016; 59: 1008-22; doi:10.1139/gen-2015-0193

[3] Calvignac-Spencer S, Merkel K, Kutzner N, et al.. Carrion fly-derived DNA as a tool for comprehensive and cost-effective assessment of mammalian biodiversity. Mol Ecol. 2013; 22: 915-24; doi:10.1111/mec.12183

[4] Rodgers, TW, Xu CCY, Giacalcone J, et al.. Carrion fly-derived DNA metabarcoding is an effective tool for mammal surveys: Evidence from a known tropical mammal community. Mol Ecol Res. 2017; 17(6): 1-13; doi:10.1111/1755-0998.12701

[5] Hoffmann C, Merkel K, Sachse A, et al.. Blow flies as urban wildlife sensors. Mol Ecol Res 2018; 18(3): 502-10; doi: 10.1111/1755-0998.12754

[6] Schönberger AC, Wagner S, Tuten HC, et al.. Host preferences in host-seeking and blood-fed mosquitoes in Switzerland. Med Vet Entomol. 2015; 30(1): 39-52.

[7] Taylor L, Cummings RF, Velten R, et al.. Host (Avian) Biting Preference of Southern California Culex Mosquitoes (Diptera: Culicidae). J Med Entomol. 2012; 49(3): 687-96.

[8] Townzen JS, Brower AVZ, Judd DD. Identification of mosquito bloodmeals using mitochondrial cytochrome oxidase subunit I and cytochrome b gene sequences. Med Vet Entomol. 2008; 22. 386-93.

[9] Kocher A, Thoisy B, Catzeflies F, et al.. iDNA screening: Disease vectors as vertebrate samplers. Mol Ecol. 2017; 26(22): 6478-86.

[10] Schnell IB, Thomsen PF, Wilkinson N, et al.. Screening mammal biodiversity using DNA from leeches. Curr Biol. 2012, 22(8): R262—3.

[11] Tessler M, Weiskopf SR, Berniker L, et al.. Bloodlines: mammals, leeches, and conservation in southern Asia. Syst Biodivers. 2018; 1-9.

[12] Weiskopf SR, McCarthy KP, Tessler M, et al.. Using terrestrial haematophagous leeches to enhance tropical biodiversity monitoring programmes in Bangladesh. J Appl Ecol. 2018: 1-11.

[13] Schnell IB, Bohmann K, Schultze SE, et al.. Debugging diversity - a pan-continental exploration of the potential of terrestrial blood-feeding leeches as a vertebrate monitoring tool. Mol Ecol Res.2018; doi: 10.1111/1755-0998.12912

[14] Calvignac-Spencer S, Leendertz FH, Gilbert MT, Schubert G. An invertebrate stomach's view on vertebrate ecology: certain invertebrates could be used as "vertebrate

samplers" and deliver DNA-based information on many aspects of vertebrate ecology. BioEssays. 2013; 35(11): 1004-13.

[15] Schnell IB, Sollmann R, Calvignac-Spencer S, et al.. iDNA from terrestrial haematophagous leeches as a wildlife surveying and monitoring tool – prospects, pitfalls and avenues to be developed. Front Zool. 2015; 12:24.

[16] Pääbo S, Poinar H, Serre D, et al.. Genetic analyses from ancient DNA. Annu Rev Genet. 2004; 38: 645-79.

[17] Hofreiter M, Paijmans JL, Goodchild H, et al. The future of ancient DNA: Technical advances and conceptual shifts. BioEssays. 2015; 37(3): 284-93.

[18] Bonin A, Taberlet P, Zinger L, Coissac E. Environmental DNA: For Biodiversity Research and Monitoring. 1st ed. Oxford University Press; 2018.

[19] Ficetola GF, Pansu J, Bonin A, et al.. Replication levels, false presences and the estimation of the presence/absence from eDNA metabarcoding data. Mol Ecol Res. 2014; 15(3): 543-56.

[20] Ficetola GF, Taberlet P., Coissac E. How to limit false positives in environmental DNA and metabarcoding? Mol Ecol Res. 2016; 16(3): 604-7.

[21] Dalton R. Still looking for that woodpecker. Nature. 2010; 463: 718-9.

[22] Bazinet AL, Cummings MP. A comparative evaluation of sequence classification programs. BMC bioinformatics. 2012; 13(1): 92.

[23] Richardson RT, Bengtsson-Palme J, Johnson RM. Evaluating and optimizing the performance of software commonly used for the taxonomic classification of DNA metabarcoding sequence data. Mol Ecol Res. 2017; 17(4): 760-9.

[24] Somervuo P, Yu DW, Xu CC, Ji Y, et al.. Quantifying uncertainty of taxonomic placement in DNA barcoding and metabarcoding. Methods Ecol Evol. 2017; 8(4): 398-407.

[25] Quast C, Gerken J, schweer T, et al. SILVA Databases. In: Nelson KE. Encyclopedia of Metagenomics. 1st ed. Springer US; 2015. p. 626-635.

[26] Kozlov AM, Zhang J, Yilmaz P, Glöckner FO, Stamatakis A. (2016). Phylogeny-aware identification and correction of taxonomically mislabeled sequences. Nucleic Acids Res. 2016; 44(11): 5022-33.

[27] Huson DH, Auch AF, Qi J, Schuster SC. MEGAN analysis of metagenomic data. Genome Res. 2007; 17(3): 377-86.

[28] Somervuo P, Koskela S, Pennanen J, Henrik Nilsson R, Ovaskainen O. Unbiased probabilistic taxonomic classification for DNA barcoding. Bioinformatics. 2016; 32(19): 2920-7.

[29] Schnell IB, Bohmann K, Gilbert MTP. (2015). Tag jumps illuminated–reducing sequence-to-sample misidentifications in metabarcoding studies. Mol Ecol Res. 2015; 15(6): 1289-1303.

[30] Kocher TD, Thomas WK, Meyer A, et al.. Dynamics of mitochondrial DNA evolution in animals: amplification and sequencing with conserved primers. Proc. Natl. Acad. Sci. U.S.A.. 1989; 86(16): 6196-6200.

[31] Taylor PG. Reproducibility of ancient DNA sequences from extinct Pleistocene fauna. Mol Biol Evol. 2996; 13(1): 283-5.

[32] Faircloth BC, Glenn TC. Not all sequence tags are created equal: designing and validating sequence identification tags robust to indels. PloS One. 2012; 7(8): e42543

[33] Murray DC, Coghlan ML, Bunce M. From benchtop to desktop: important considerations when designing amplicon sequencing workflows. PLoS One. 2015; 10(4): e0124671.

[34] Machida RJ, Leray M, Ho SL, Knowlton N. Metazoan mitochondrial gene sequence reference datasets for taxonomic assignment of environmental samples. Sci Data. 2017; 4: 170027.

[35] Chamberlain SA, Szöcs E. taxize: taxonomic search and retrieval in R. Version 2. F1000Res. 2013; 2: 191.

[36] Salleh FM, Ramos-Madrigal J, Peñaloza F, et al.. An expanded mammal mitogenome dataset from Southeast Asia. GigaScience. 2017; 6(8): 1-8

[37] Kans, Jonathan. Entrez Direct: E-utilities on the UNIX Command Line. In: Entrez Programming Utilities Help [Internet]. Bethesda (MD): National Center for Biotechnology Information (US). 2010.

[38] Altschul SF, Gish W, Miller W, Myers EW, Lipman DJ. Basic local alignment search tool. Journal of molecular biology. 1990; 215(3):, 403-10.

[39] Camacho C, Coulouris G, Avagyan V, et al.. BLAST+: architecture and applications. BMC bioinformatics. 2009; 10(1): 421.

[40] Katoh K, Standley DM. (2013). MAFFT multiple sequence alignment software version 7: improvements in performance and usability. Mol Biol Evol. 2013; 30(4): 772-80.

[41] Katoh K, Misawa K, Kuma KI, Miyata T. MAFFT: a novel method for rapid multiple sequence alignment based on fast Fourier transform. Nucleic Acids Res. 2002; 30(14): 3059-66.

[42] Chesters D. (2013) collapsetypes.pl [computer software available at http://sourceforge.net/projects/collapsetypes/]

[43] Edgar RC. Search and clustering orders of magnitude faster than BLAST. Bioinformatics. 2010; 26(19): 2460-2461.

[44] Edgar RC, Flyvbjerg H. Error filtering, pair assembly and error correction for next-generation sequencing reads. Bioinformatics. 2015; 31(21): 3476-82.

[45] Martin M. Cutadapt removes adapter sequences from high-throughput sequencing reads. EMBnet. Jjournal. 2011; 17(1): 10-12.

[46] Kiełbasa SM, Wan R, Sato K, Horton P, Frith MC. Adaptive seeds tame genomic sequence comparison. Genome Res. 2011; 21(3): 487-493.

[47] Ratnasingham S, Hebert PDN. BOLD: The Barcode of Life Data System (www.barcodinglife.org). Mol Ecol Notes. 2007; 3: 355-64.

[48] Evans NT, Li Y, Renshaw MA, et al. Fish community assessment with eDNA metabarcoding: effects of sampling design and bioinformatic filtering. Can J Fish Aquat Sci. 2017; 74(9):, 1362-74.

[49] Zepeda-Mendoza ML, Bohmann K, Baez AC, Gilbert MTP. DAMe: a toolkit for the initial processing of datasets with PCR replicates of double-tagged amplicons for DNA metabarcoding analyses. BMC Res Notes. 2016; 9(1): 255.

[50] Racimo F, Renaud G, Slatkin M. Joint estimation of contamination, error and demography for nuclear DNA from ancient humans. PLoS Genet. 2016; 12(4): e1005972.

[51] Orlando L, Gilbert MTP, Willerslev E. Reconstructing ancient genomes and epigenomes. Nat Rev Genet 2015; 16(7): 395

[52] Laurin-Lemay S, Brinkmann H, Philippe H. Origin of land plants revisited in the light of sequence contamination and missing data. Current Biology. 2012; 22(15): R593-4.

[53] Kwok S, Higuchi R. Avoiding false positives with PCR. Nature. 1989; 339: 237-8.

[54] Bush A, Sollmann R, Wilting A, et al.. Connecting Earth observation to high-throughput biodiversity data. Nat Ecol Evol 2017; 1(7): 0176.

Table 1: Sequence motifs that compose the 25 different target primers for the first and the second PCR. First PCR primers consist of target specific primer followed by an overhang out of sample specific *tag 1* and *read 1* and *read 2* sequencing primer, respectively. The second PCR primers consist of the *read 1* or the *read 2* sequencing primer followed by an plate specific *tag 2* and the P5 and P7 adapters, respectively (see also Fig. 2).

Name	Sequence	Reference
tag A	TGCAT	Faircloth & and Glenn 2012
tag B	TCAGC	Faircloth & and Glenn 2012
tag C	AAGCG	Faircloth & and Glenn 2012
tag D	ACAAG	Faircloth & and Glenn 2012
tag E	AGTGG	Faircloth & and Glenn 2012
tag F	TTGAC	Faircloth & and Glenn 2012
tag G	CCTAT	Faircloth & and Glenn 2012
tag H	GGATG	Faircloth & and Glenn 2012
tag I	CTAGG	Faircloth & and Glenn 2012
tag K	CACCT	Faircloth & and Glenn 2012
tag L	GTCAA	Faircloth & and Glenn 2012
tag M	GAAGT	Faircloth & and Glenn 2012
tag N	CGGTT	Faircloth & and Glenn 2012
tag O	ACCGA	Faircloth & and Glenn 2012
tag P	ACGTC	Faircloth & and Glenn 2012
tag Q	AGACT	Faircloth & and Glenn 2012
tag R	AGGAA	Faircloth & and Glenn 2012
tag S	ATTCC	Faircloth & and Glenn 2012
tag T	CAATC	Faircloth & and Glenn 2012
tag V	CATGA	Faircloth & and Glenn 2012
tag W	CCACA	Faircloth & and Glenn 2012
tag X	GCTTA	Faircloth & and Glenn 2012
tag Y	GGTAC	Faircloth & and Glenn 2012
tag Z	AACAC	Faircloth & and Glenn 2012
Tag Control	ATCTG	Faircloth & and Glenn 2012
CytB-fw	AAAAAGCTTCCATCCAACATCTCAGCATGATGAAA	Kocher et al. 1989
CytB-rv	AAACTGCAGCCCCTCAGAATGATATTTGTCCTCA	Kocher et al. 1989
16S-fw	CGGTTGGGGTGACCTCGGA	Taylor 1996
16S-rv	GCTGTTATCCCTAGGGTAACT	Taylor 1996
12S-fw	AAAAAGCTTCAAACTGGGATTAGATACCCCACTAT	Kocher et al. 1989
12S-rv	TGACTGCAGAGGGTGACGGGCGGTGTGT	Kocher et al. 1989
Read 1 sequence primer	ACACTCTTTCCCTACACGACGCTCTTCCGATCT	Illumina Document # 1000000002694 v03
Read 2 sequence primer	GTGACTGGAGTTCAGACGTGTGCTCTTCCGATCT	Illumina Document # 1000000002694 v03
P5 adapter	AATGATACGGCGACCACCGAGATCTACAC	Illumina Document # 1000000002694 v03
P7 adapter	CAAGCAGAAGACGGCATACGAGAT	Illumina Document # 1000000002694 v03

Table 2: Main steps undertaken by each module of the database curation script.

MODULE	STEPS
Module 1	Extract subset of raw MIDORI database for query taxon and loci.
	Remove sequences with non-binomial species names, reduce subspecies to species labels
	Add local sequences (optional)
	Check for relevant new sequences for list of query species on NCBI (GenBank and RefSeq) (optional)
	Select amplicon region and remove primers
	Remove sequences with ambiguous bases
	Align
	End of module: Optional check of alignments
Module 2	Compare sequence species labels with taxonomy
	Non-matching labels queried against Catalogue of Life to check for known synonyms
	Remaining mismatches kept if genus already exists in taxonomy, otherwise flagged for removal
	End of module: Optional check of flagged species labels
Module 3	Discard flagged sequences
	Update taxonomy key file for sequences found to be incorrectly labelled in Module 2
	Run SATIVA
	End of module: Optional check of putatively mislabelled sequences
Module 4	Discard flagged sequences
	Finalise consensus taxonomy and relabel sequences with correct species label and accession number
	Select one representative sequence per haplotype per species

Table 3: GNU core utilities and other lightweight tools used for manipulation of text and sequence files

TOOL	FUNCTION	SOURCE
awk, cut, grep, join, sed, sort, tr	Processing text files	GNU core utilities
seqbuddy	Processing FASTA/Q files	https://github.com/biologyguy/BuddySuite
seqkit	Processing FASTA/Q files	https://github.com/shenwei356/seqkit
seqtk	Processing FASTA/Q files	https://github.com/lh3/seqtk
tabtk	Processing tab-delimited text files	https://github.com/lh3/tabtk

Table 4: Number of reads per sequencing run and the numbers of reads with matching, chimeric or unidentifiable tags.

	total reads	matching tag 2 reads	chimeric tag 2 reads	%[1]	matching tag 1 reads	chimeric tag 1 reads	%[2]	erroneous tag 1 reads	%[2]
**SeqRun01**	18,438,517	18,102,702	282,419	1.5	17,514,515	451,028	2.5	137,159	0.8
**SeqRun02**	25,385,558	24,596,380	626,245	2.5	23,426,084	612,045	2.5	558,251	2.3
**SeqRun03**	14,875,796	14,393,884	343,528	2.3	13,766,187	426,181	3.0	201,516	1.4
**SeqRun04**	2,027,794	1,935,149	56,077	2.8	1,806,655	88,307	4.6	40,187	2.1
**SeqRun05**	18,221,504	17,500,366	421,588	2.3	16,793,851	482,365	2.8	161,458	0.9
**SeqRun06**	20,718,202	19,874,913	429,048	2.1	19,317,305	371,048	1.9	81,422	0.4
**SeqRun07**	24,604,610	23,746,938	663,730	2.7	22,446,187	497,366	2.1	803,385	3.4
**Total**	124,271,981	120,150,332	2,822,635	2.3	115,070,784	2,928,340	2,5	1,983,378	1,7
**IndexRun**	10,276,093	10,116,808	NA	NA	5,841,190	4,186,688	41.4	88,930	0.9

[1] refers to total reads

[2] refers to matching tag 2

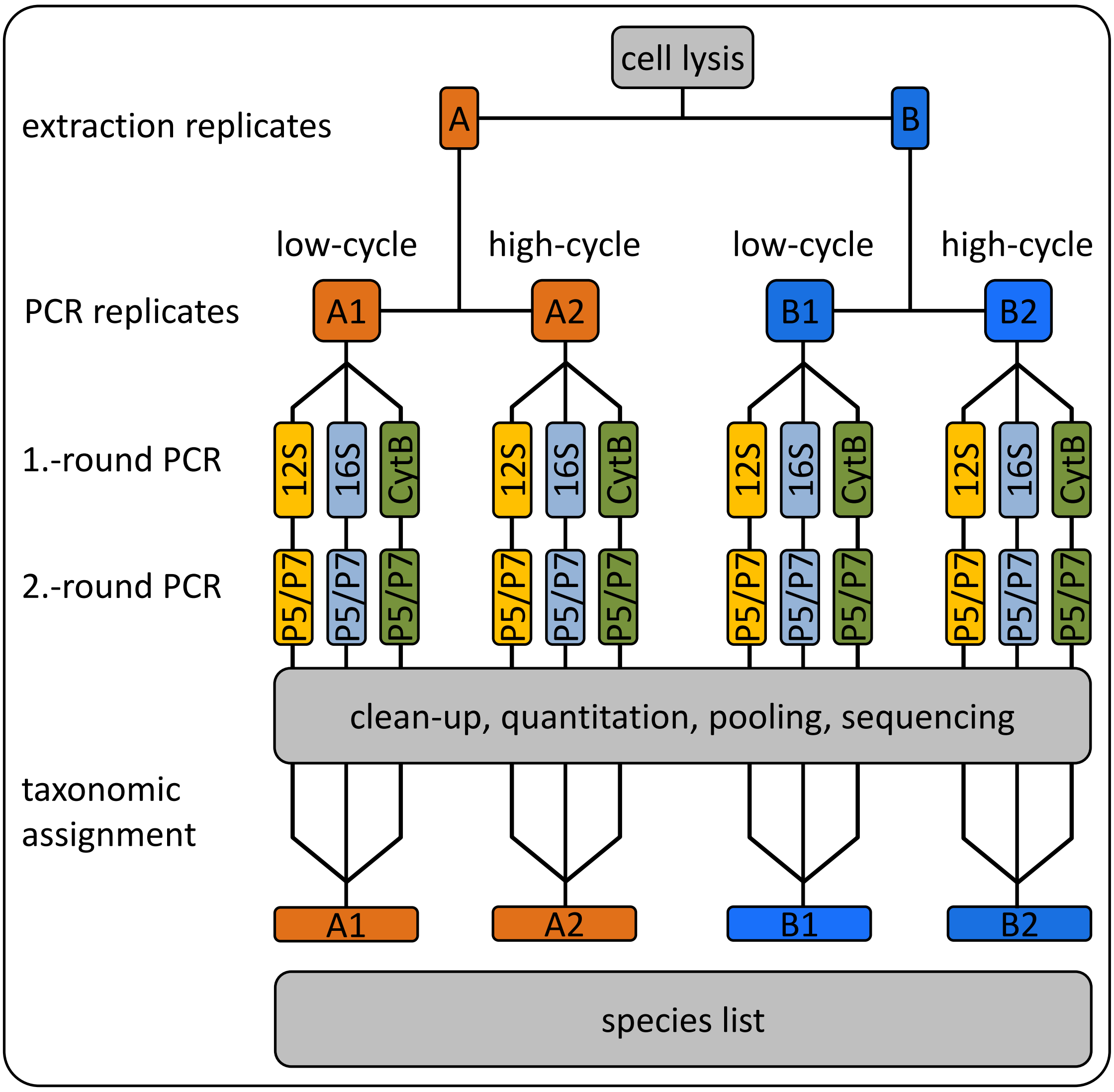

Figure 1: laboratory scheme; during DNA extraction the sample is split into two extraction replicates A & B. Our Protocol consists of two rounds of PCR that were the sample tags, the necessary sequencing primer and sequencing adapters are added to the the amplicons. For each extraction replicate we ran a low cycle PCR and a high cycle PCR for each marker that we have twelve independent PCR replicates per sample. All PCR products were sequenced and the obtained reads were taxonomically identified with PROTAX.

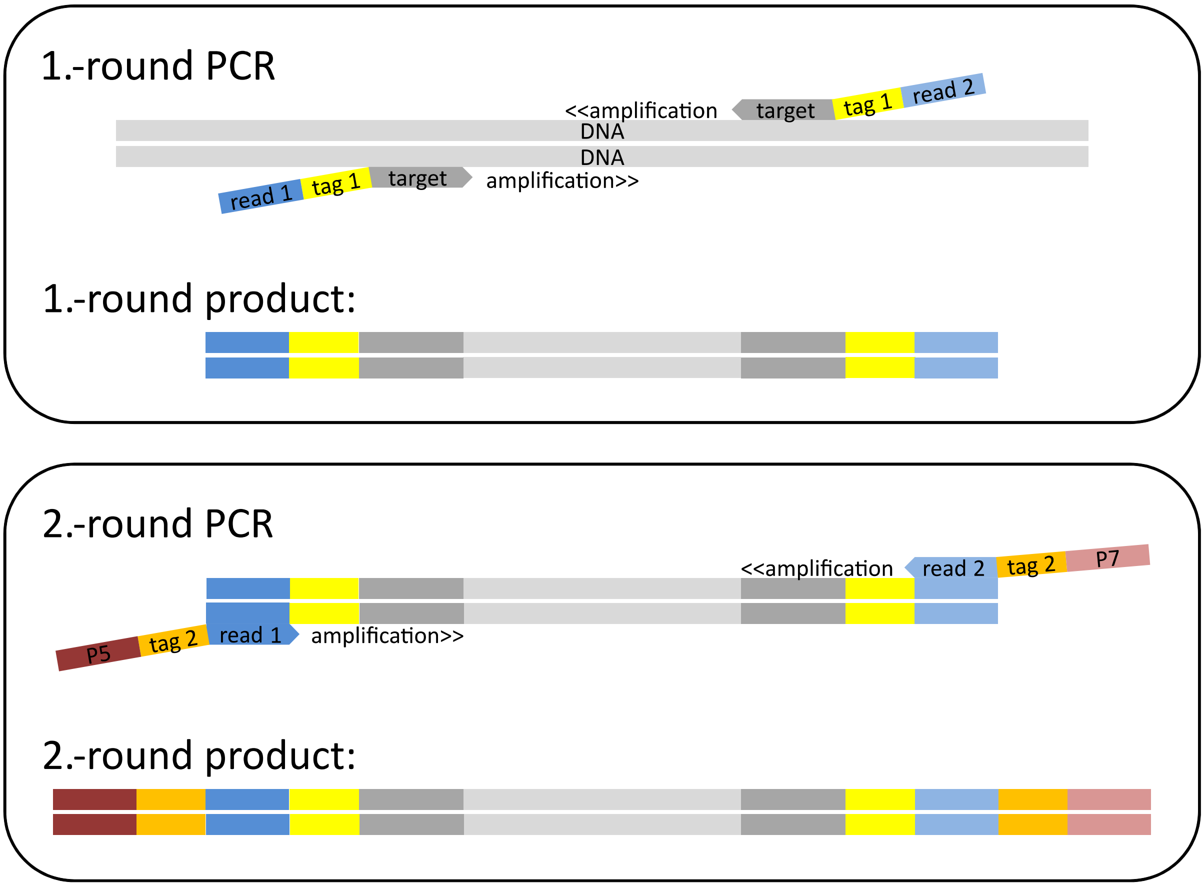

Figure 2: Scheme to build double ‘twin-tagged’ PCR libraries. The first round of PCR uses target-specific primers (12S, 16S, or CytB, dark grey) that have both been extended with the same (i.e. ‘twin’) sample-identifying *tag* sequences *tag 1* (yellow) and then with the different *read 1* (dark blue) and *read 2* (light blue) sequence primers. The second round of PCR uses the priming sites of the *read 1* and *read 2* sequencing primers to add twin plate-identifying *tag* sequences *tag 2* (orange) and the P5 (dark red) and P7 (light red) Illumina adapters.

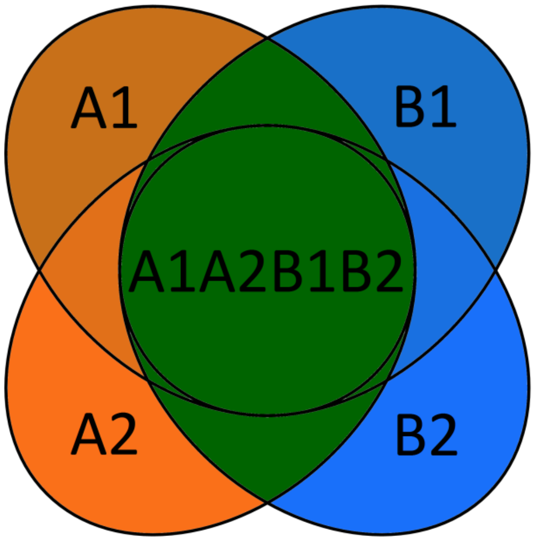

Figure 3: We only accepted taxonomic assignments that were positively detected in both *extraction replicates* A and B (green colour).

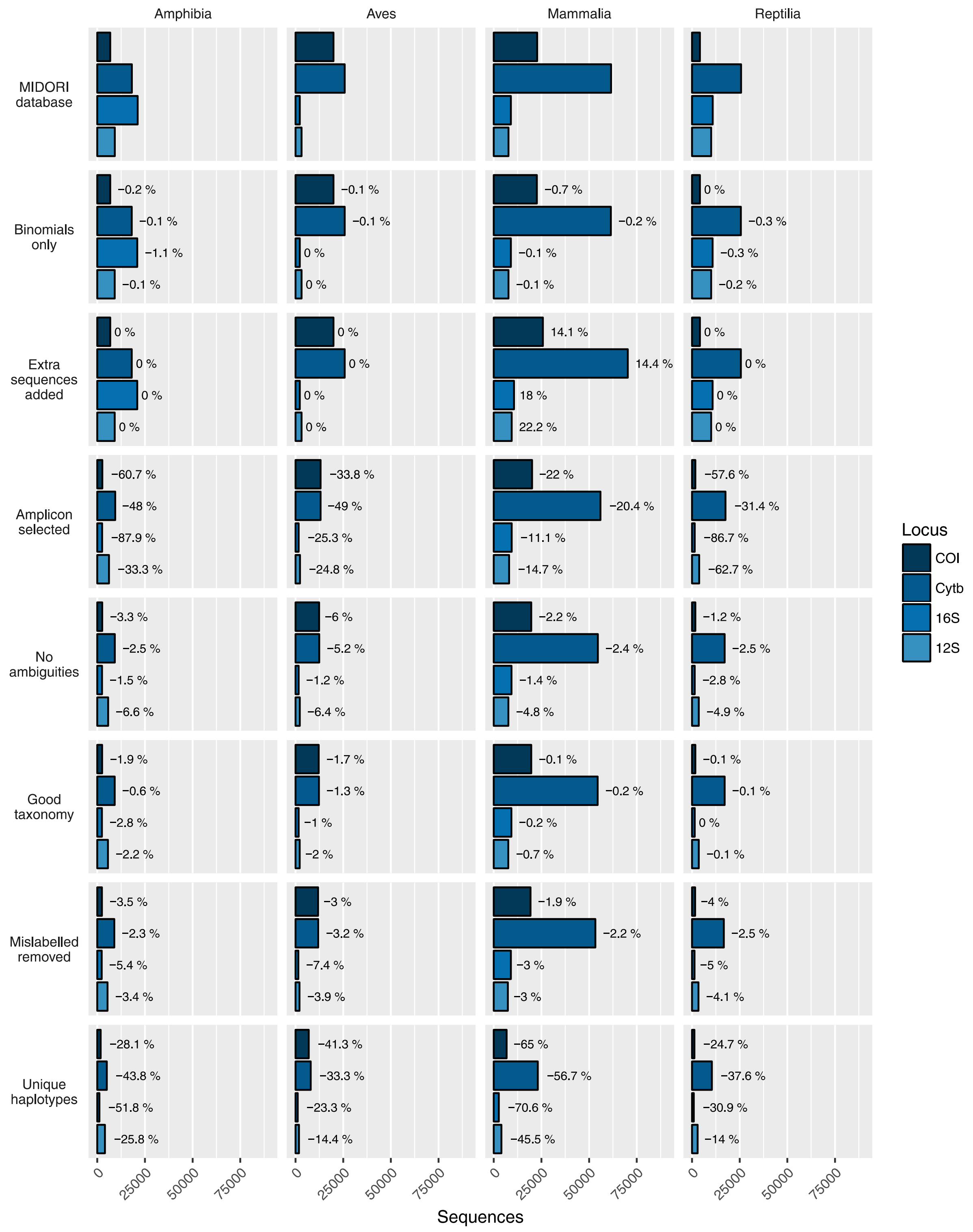

Figure 4: Data availability and percentage loss at each major step in the database curation procedure for each target amplicon and class of Tetrapoda. The number of sequences decreases between steps except “Extra sequences added” where additional target sequences are included for Mammalia and there is no change for the other three classes.

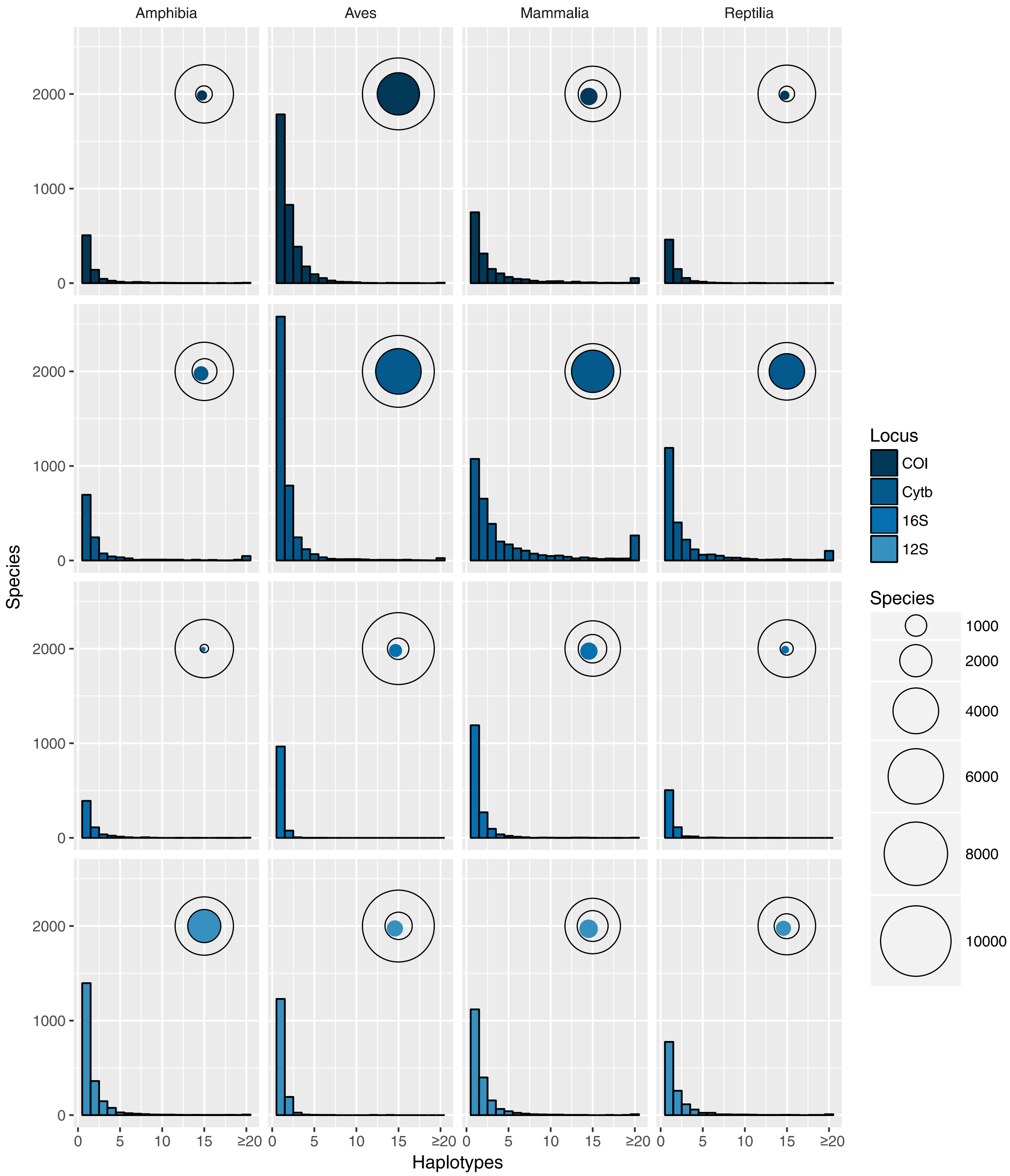

Figure 5: Haplotype number by species (frequency distribution) and the total number of species with at least one haplotype, shown relative to the total number of species in the taxonomy for that category (bubbles), shown for each marker and class of Tetrapoda. The proportion of species covered by the database varies between categories but in all cases a majority of recovered species are represented by a single unique haplotype.

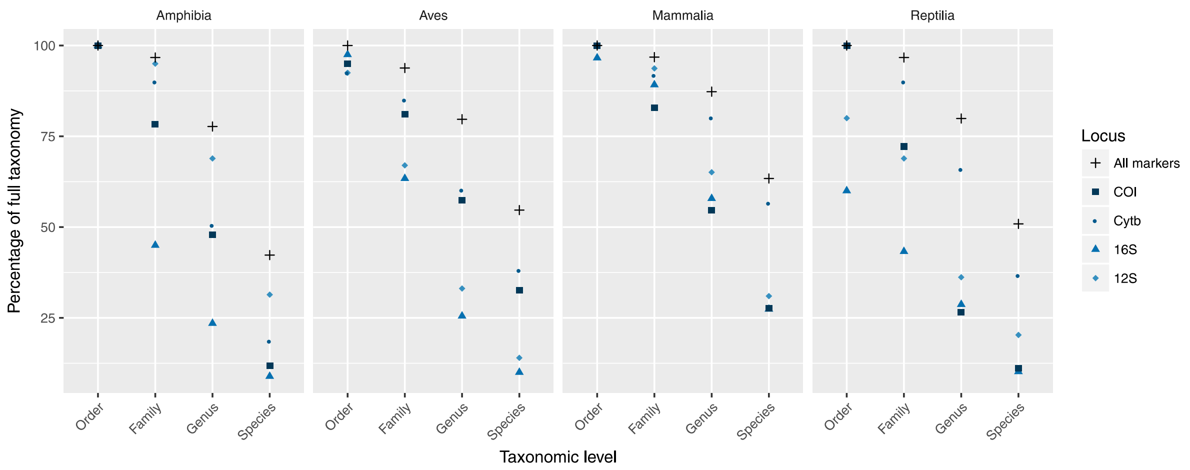

Figure 6: The percentage of the full taxonomy covered by the final database at each taxonomic level for each class of Tetrapoda. Includes the percentage of taxa represented by each marker and all markers combined. In all cases taking all four markers together increases the proportion of species, genera and families covered by the database but it remains incomplete when compared with the full taxonomy.

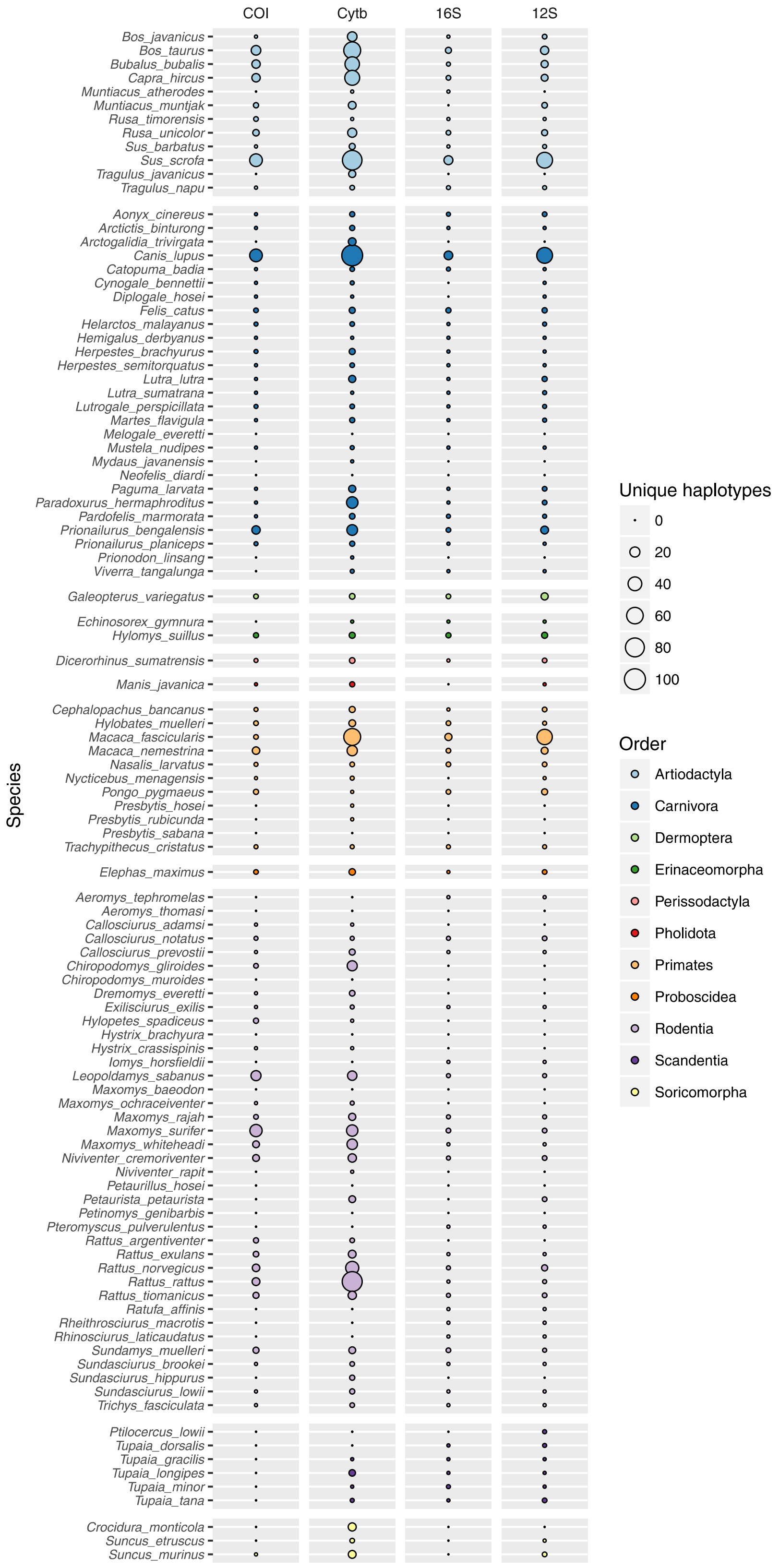

Figure 7: The number of unique haplotypes per marker for each of the 103 mammal species expected in the study area. Bubble size is proportional to the number of haplotypes and varies between 0 and 100. Only 554 species have at least one sequence per marker and nine species are completely unrepresented in the current database.

supplemental figure 1

Click here to access/download
Supplementary Material
Supplement figure 1.pdf

supplemental table 1

Click here to access/download
Supplementary Material
Supplement table 1.pdf

supplemental table 2

Click here to access/download
Supplementary Material
Supplement table 2.pdf
