# Supplementary material for: An efficient and robust laboratory workflow and tetrapod database for larger scale environmental DNA studies
Source: Gigascience. 2019 Apr 13;8(4):giz029. doi: 10.1093/gigascience/giz029 (PMC6461710; doi:10.1093/gigascience/giz029)
Supplement: GIGA-D-18-00219_Revision_1.pdf [file giz029_giga-d-18-00219_revision_1.pdf]

GigaScience

An efficient and robust laboratory workflow and tetrapod database for larger scale eDNA studies

--Manuscript Draft--

**Manuscript Number:**	GIGA-D-18-00219R1
**Full Title:**	An efficient and robust laboratory workflow and tetrapod database for larger scale eDNA studies
**Article Type:**	Data Note
**Funding Information:**	German Federal Ministry of Education and Research (BMBF) (01LN1301A) — Dr. Andreas Wilting
**Abstract:**	Background<br>The use of environmental DNA, 'eDNA,' for species detection via metabarcoding is growing rapidly and now, even terrestrial mammals can be monitored via 'invertebrate-derived DNA' or 'iDNA' from hematophagous invertebrates. We present a co-designed lab workflow and bioinformatic pipeline to mitigate the two most important risks of e/iDNA: sample contamination and taxonomic mis-assignment. These risks arise from the need for amplification to detect the trace amounts of DNA and the necessity of using short target regions due to DNA degradation.<br>Findings<br>Here we present a high-throughput laboratory workflow that minimises these risks via a three-step strategy: (1) each sample is sequenced for two PCR replicates from each of two extraction replicates; (2) we use a 'twin-tagging,' two-step PCR protocol; (3) and a multi-marker approach targeting three mitochondrial loci: 12S, 16S and CytB. As a test, 1532 leeches were analysed from Sabah, Malaysian Borneo. Twin-tagging allowed us to detect and exclude chimeric sequences. The smallest DNA fragment (16S) amplified best for all samples but often at lower taxonomic resolution. We only accepted assignments that were found in both extraction replicates, totalling 174 assignments for 96 samples.<br>To avoid false taxonomic assignments, we also present an approach to create curated reference databases that can be used with the powerful taxonomic-assignment method PROTAX. For some taxonomic groups and some markers, curation resulted in over 50% of sequences being deleted from public reference databases, due mainly to: (1) limited overlap between our target amplicon and available reference sequences; (2) apparent mislabelling of reference sequences; (3) redundancy. A provided bioinformatics pipeline processes amplicons and conducts the PROTAX taxonomic assignment.<br>Conclusions<br>Our metabarcoding workflow should help research groups to increase the robustness of their results and therefore facilitate wider usage of e/iDNA, which is turning into a valuable source of ecological and conservation information on tetrapods.
**Corresponding Author:**	Jan Axtner<br>Leibniz Institute for Zoo and Wildlife Research<br>Berlin, Germany GERMANY
**Corresponding Author Secondary Information:**	
**Corresponding Author's Institution:**	Leibniz Institute for Zoo and Wildlife Research
**Corresponding Author's Secondary Institution:**	
**First Author:**	Jan Axtner
**First Author Secondary Information:**	
**Order of Authors:**	Jan Axtner<br>Alex Crampton-Platt<br>Lisa Hörig

Powered by Editorial Manager® and ProduXion Manager® from Aries Systems Corporation

	Azlan Mohamed
	Charles C.Y. Xu
	Douglas W. Yu
	Andreas Wilting
**Order of Authors Secondary Information:**	
**Response to Reviewers:**	Reviewer #1: In this manuscript Axtner et al. 1) used metabarcoding to assess mammal diversity from leeches and 2) developed a pipeline to build a curated reference database for taxonomic assignment. An emphasis of the metabarcoding was replication, including replication at the extraction, amplification, and locus levels. This work is of interest because 1) more robust inferences from metabarcoding may be possible by looking for concordance across replicates at multiple levels in the analysis process and 2) accurate taxonomic assignment is often limited by database accuracy and completeness. Thus, the manuscript is likely of interest not only to other iDNA users, but to metabarcoding users generally (e.g., eDNA, diet analysis, plant-pollinator interactions). I have three major comments and some more minor comments below. Generally, I think the authors could build a stronger case for their extensive lab and database work by taking a more quantitative approach to assessing success. Also, it was not very clear throughout the manuscript where to access the raw sequence data (FASTQ files from bcl2fastq probably fine), taxonomic assignments, scripts, etc. Places like Line 404 should include info on where to get the script.<br><br>Major comment 1:<br><br>Although I appreciate the value of replication, I think the manuscript would benefit from a quantitative assessment of the effect that replication had on inference accuracy. In the title the authors say this workflow is "improved". How can you demonstrate this?<br>-->We agree with the reviewer that we should have been more careful with our word choice in the title and that the word improved could raise the expectation of the reader that our approach was tested against existing protocols. As this was not the intention of this manuscript, we have replaced the word “improved” with “robust”. The title now reads:<br>“An efficient and robust laboratory workflow and tetrapod database for larger scale eDNA studies”<br>We used the word improved in the first version as we wanted to highlight that there are currently no gold-standard protocols for e/iDNA studies, and results are often presented without a thorough reporting on how they were generated. This is the sense in which we expect our workflow to improve current practice.<br>The development of our workflow was driven by the need to analyse over 1000 samples with over 7500 leeches. Afterwards, we realized during discussions with colleagues that our approach and the pipelines we developed would be of value for other researchers. In particular, despite its obvious value to the community, PROTAX is very difficult to deploy, the original developer is focused on other projects, and so this manuscript is the only step-by-step explanation of how to implement it properly. We think the word robust is very defensible, given results from other publications (e.g. Protax shown to be improved over standard methods in Rodgers et al. 2017, and the use of multiple PCR replicates shown to be improved over standard methods in Zepeda-Mendoza 2016). Bringing this all together has not been done, which is where our publication comes in.<br><br><br><br>One of the main points of this manuscript is the value of technical replication to reduce false positive errors. Thus, each sample has replicate extractions, each extraction replicate loci, and each loci replicate PCR. As described, this is probably intuitively of value to folks who work with low-DNA applications. The idea being that something that is real should be something that you can detect repeatedly. What's lacking to me is a quantitative justification or assessment of these replication levels and the thresholds assigned to them for interpretation. Can you provide a quantitative answer to these questions?<br>-Does the rule of detection in 2/2 extraction replicates reduce estimated false positive rates compared to only 1/2 replicates?

Powered by Editorial Manager® and ProduXion Manager® from Aries Systems Corporation

-->Yes. We have added a simulation study to the supplements using different PCR detection rates (please see supplemental figure 1). This simulation shows that false positive detections can be reduced by the stringent A/B acceptance criterion (see below and supplemental figure 1) compared to more a lax acceptance criterion where we accepted assignment with at least two positives in any the PCR replicates. Our lax approach refers to one of the approaches of Ficetola et al. 2015 where they evaluated different statistical approaches developed to estimate occupancy in the presence of observational errors and has been applied in other studies (e.g. Schnell et al. 2018). At the same time we see in this simulation that the rate of false negatives increased. We argue in the discussion that in some cases, e.g. rare and threatened species, it is more important to reduce false positives than to reduce false negatives. This is because there already exists a well-developed statistical procedure, occupancy modelling, that can correct for false negatives, whereas nothing nearly as well developed exists for correcting for false positives (see l. 550-61 in the manuscript). Non-detection within the laboratory process is just one of the factors influencing overall detection probability, which can be modeled in hierarchical occupancy models (see also Schnell et al. 2015). However, our workflow design allows the flexibility to address this issue if necessary and to use different acceptance criteria. To show and to address this we included now results on both, the stringent and the more lax approach in comparison in the manuscript.

-Is only requiring detection in 1/2 PCR replicates per marker sufficient, or would requiring 2/2 PCR replicates reduce the estimate false positive rate?
-->It turns out that requiring even 1/2 detections per marker would be unachievable for the 12S and CytB markers, because those markers had low amplification success, since the amplicon length is longer than the more successful 16S and we are working with degraded DNA. Furthermore the read quality decreased significantly with read length as we could demonstrate (see l. 566-68). Nevertheless these longer fragments helped often to disentangle the taxonomic inconsistencies caused by too high inter-specific genetic similarities in the 16S sequences or missing references in the database. In line with other publications (Ficetola et al 2015, Zepeda-Mendoza 2016, Schnell et al 2018) we think that reproducibility is important and that a detection of minimum two detection out of the PCR replicates (independently of the loci) is needed (added relaxed approach). But, in the manuscript, we also advocate using the criterion of requiring replicates from both A and B extractions to detect species if necessary, as this filter also removes possible contamination during DNA extraction and gives even higher confidence in the results. Our workflow does allow to apply such a more strict view if it is needed.

-What is the effect of the used 10 reads threshold versus other thresholds (e.g., 5, 50, 100) on the estimate false positive rate? How did you --determine that this threshold could be dropped if the taxon was detected with >1 locus?
-->10 reads was chosen because we realized that often a low number of reads were assigned to a sister taxon due to amplification or sequencing errors etc. (see l. 642-47). Obviously a threshold of 10 is chosen somewhat arbitrarily and it certainly depends on many factors such as sequencing depth, used PCR cycles, amplicon length and/or other factors affecting PCR stochasticity. Thus it needs to be evaluated based on the individual setup and dataset, but after analyzing hundreds of leech samples, this threshold proved to give internally consistent results. As the most likely contaminations in the lab would be amplicons we dropped the threshold if a taxon occurred in more than one marker as we believe it is unlikely that amplicons of two markers of the same taxon contaminate the same other sample. Note that other researchers are not held to this specific threshold and will need to make a judgement as well, guided by the internal consistency of the results.

I'd suggest that if you can't answer these with empirical data or a reasonable probability model, then you can't really argue that your replication approaches were any "better" than any other given approach.
-->As said above, we have changed our title and wording to emphasize that our goal is

Powered by Editorial Manager® and ProduXion Manager® from Aries Systems Corporation

to present a detailed workflow with many internal checks, all of which are expected to reduce false-positive detections. Perhaps the biggest value of this manuscript is that it will help other researchers consider all the possible sources of error in their own e/iDNA pipelines. We of course provide a cost-efficient mitigation for those sources of error, but other researchers will likely come up with others. However, our workflow offers some advantages, 1. it reduces the risk of false identifications due to wrong annotated references, 2. the multiple-marker approach allows to identify more species with regard to incomplete reference databases, 3. it gives you more control over different levels uncertainties during the laboratory steps and thus more confidence if this is needed.
That said, we did compare our results to the DAMe pipeline (now added in l. 588-94), which also uses twin-tagging to detect and remove tag-jump events, but they used a blunt-ligation technique to carry out library prep (in contrast to our PCR-based method). DAMe's authors report tag jumping in 19.15% and 23.1% of sequences (Zepeda-Mendoza 2016, table 1), whereas we found only 4.6% of reads with non-matching tags (t1 or t2) (table 4). Our number might not be one to one comparable as they counted unique sequences and we reported on read numbers, but this is a big difference and is in favour of our method.
More generally, as the reviewer alludes to, the field of metabarcoding (in a/i/eDNA) has already identified many sources of error and also suggested remedies (e.g. Ficetola et al. 2015). Our contribution here is to synthesize this knowledge in an integrated workflow that considers both the wet- and dry-lab portion, including extensive work on the taxonomic-assignment step, which is, to our mind, the least well developed step in metabarcoding currently.
Also, we do not pretend to define universal filtering thresholds for all studies, as the desirable stringency depends in part on one's research question. For occupancy modelling, it is crucial to avoid false-positives, even at the expense of false-negatives. False-positive detections can lead to misdirected conservation effort, especially for very rare, very high-value species like the Saola, the large-antlered Muntiac or Sumatran rhino. If, on the other hand, one is working on more abundant and widespread species, then a low level of false-positives will not likely influence the inferred species distributions.
Finally, identifying assignments as likely false negatives still allows practitioners to flag up those unreliable assignments and make a management decision on what to do, such as to combine with other information about the sample site and perhaps to increase sampling effort in those sites. We have highlighted this more clearly in the manuscript now by adding the following section and supplemental figure 1:
L. 543-57 "...We only accepted sequences that appeared in a minimum of two independent PCRs for the lax and for the stringent criterion, where it has to occur in each extraction replicate A and B (figure 1). The latter acceptance criterion is quite conservative and produces higher false negative rates than e.g. accepting occurrence of at least two positives. However, it also reduces the risk of accepting a false positives compared to it (see supplemental figure 1. for a simulation of false positive and false negatives rates within a PCR), especially with increasing risk of false positive occurrence in a PCR for e.g. example due to higher risk of contamination etc.. Metabarcoding studies are very prone to false negatives, and downstream analyses like occupancy models for species distributions can account for imperfect detection and false negatives. However, methods for discounting false positive detections are not well developed [60]. Thus we think it is more important to avoid false positives, especially if the results will be used to make management decisions regarding rare or endangered species. In contrast, it might be acceptable to use a relaxed acceptance criterion for more common species, as long as the rate false-positives/true-positives is small and does not affect species distribution estimates. Employing both of our tested criteria researchers could flag unreliable assignments and management decisions can still use this information, but now in a forewarned way. ..."

There's another potential issue here, which is not discussed, which is the false negative rate. By requiring replicated detections, you drive down your false positive rate, but drive up your false negative rate. If the false negative rate per extraction/PCR is very low, maybe this doesn't matter much, but it could be quite large. For example, there is a recent discussion in the literature related to this idea with a focus on PCR

replicates:
Ficetola et al. 2015. Replication levels, false presences and the estimation of the presence/absence from eDNA metabarcoding data. Molecular Ecology Resources. https://doi.org/10.1111/1755-0998.12338
Lahoz-Monfort et al. 2016. Statistical approaches to account for false-positive errors in environmental DNA samples. Molecular Ecology Resources
Ficetola et al. 2016. How to limit false positives in environmental DNA and metabarcoding? Molecular Ecology Resources
--> Yes, we are aware of this discussion and have cited the relevant papers. As said above we have expanded our discussion on false positives and false negative rates in the manuscript and also added the simulation in the supplements. As we discuss above, we generally lean toward trying to reduce the false-positive rate at the cost of driving up the false-negative rate, due to the fact that occupancy modelling is built for the former error. And also, driving up the false-negative rate does not mean deleting the data. It just means flagging some assignments as being unreliable, and management decisions can still use this information, but now in a forewarned way. More quantitatively, with our stringent criteria we are increasing our false negative rate (see added supplementary figure 1). Here is a simple example from our simulation: If we assume a low detection probability of 0.25 for every PCR, and if we treat all 12 replicates alike and accept every assignment that occurs in at least 2 replicates (lax criteria), we would have a chance of 84% to detect a species correctly. If we instead use the stringent criteria, this chance decreases to 64%. But if we assume a 50% chance to detect a present DNA with the PCR both acceptance criteria are already below a 5% to accept a false negative. The tricky thing remains to decide what our chances are to detect a present DNA correctly.
However, the stringent criterion evidently also decreases the risk of accepting false positives especially for higher risks (see supplementary figure 1), e.g. if you have low PCR detection probabilities due to low amounts of target DNA and you have to run high cycle PCR. We agree and think researchers have to be aware of these risks and due to your comment we decided to present now the two acceptance criteria. To us most important is that our workflow design gives users the flexibility to apply a acceptance criteria that fits their needs and that users are aware of the risks applying too lax approaches. In some cases it is more important to avoid false positives than having false negatives. Lahoz-Monfort et al. (2016) also pointed out that multiscale occupancy models that account for both FN and FP errors are not yet developed and that FP errors can originate at several stages of eDNA collection and analysis. As said, occupancy model are very sensitive to false positives in the datasets but assume an imperfect detection and thus are less affected by false negatives. In the manuscript we also discuss the meaning of false positives for conservation measurements and why we think it is most important to avoid them while accounting for false negatives (see l. 107-110).

Major comment 2:

For the database, I would have liked to see the authors show that their database curation decreased the false positive rate or in some other way increased the accuracy of their inferences. Is this curated database approach necessary to apply PROTAX, or could the original sequences with redundancy and mislabeling have been used as well? If this pipeline is a major product in the manuscript (as the Abstract suggests) how can you quantitatively demonstrate to the reader that it is worth using?
-->The curated database is not required for using Protax, and Protax of course is not required for using the curated database (or for using an uncurated database, of course). We do not have estimates here of the reduction in error when using Protax, but Rodgers et al. (2017) did this analysis already, using an earlier version of Protax in Barro Colorado Island, Panama. The benefit of that study is that the vertebrate community is very well characterized, and the fly metabarcoding results could be checked against this a priori knowledge. In their Figure 2, Protax resulted in a much lower false positive rate (esp. for 12S species-rank assignments, and especially if we weighted the results to favor species known from Panama or from BCI itself).
On the other side, Protax was as good or better than BLAST or BLAST+Megan for true-positive detections (although this varied a bit depending on which gene and which

taxonomic rank). These results were run on a non-curated database.
The other part of the question is whether curation is necessary. We think the answer to this is a self-evident yes. Errors in a reference database cannot help but result in errors, either to an overly confident assignment or to an overly vague assignment. As an easily replicated example, human sequences are often assigned by BLAST+Megan to very high ranks, such as Eukarya, because human sequences have been incorrectly uploaded in place of other taxa of interest (ranging all over the Eukarya). There is good reason to believe that Genbank contains a large number of errors, as our own results showed (e.g. the many sequences flagged by SATIVA showing inconsistency of taxonomy with phylogenetic location) and also by Nilsson et al. (2006), who suggest that as many as 20% of GenBank fungal sequences are mislabeled.

Major comment 3:

It took be a bit of time to grasp the potential value of doing two rounds of PCR with sets of doubly-indexed primers. It wasn't completely clear to me that different combinations of first round and second round indices were used to increase the level of multiplexing. I think the proposed benefits of this approach could use a bit more explanation, including some caveats.
-->We apologize that the presentation including the benefits of our approach were not clear enough. We rephrased this section to make it clearer for the reader. These sections read now:
L. 294-309, Methods: "...We modified primers of the three markers to avoid the production of unlabelled PCR products, to allow the detection and deletion of tag-jumping events [43], and to reduce the cost of primers and library preparation. We used two rounds of PCR. The first round amplified the target gene and attached one of 25 different 'twin-tag' pairs (tag 1), identifying the sample within a given PCR. By 'twin-tag,' we mean that both the forward and reverse primers were given the same sample-identifying sequence ('tags') added as primer extensions (Fig. 2). The tags differed with a minimum pairwise distance of three nucleotides ([43]; Supplemental Table 1). These primers also contained different forward and reverse sequences (Read 1 & Read 2 sequence primers) (Supplemental Table 1) to act priming sites for the second PCR round (Fig. 2).
The second round added the Illumina adapters for sequencing and attached one of 20 twin-tag pairs (tag 2) identifying the PCR, with a minimum pairwise distance of three [44]. These primers also contained the Illumina P5 and P7 adapter sequences (Fig. 2). Thus no unlabelled PCR products were ever produced, and the combination of tags 1 and 2 allowed the pooling of up to 480 (=24 X 20) samples in a single library preparation step (one tag 1 was reserved for controls). Twin tags allowed us later to detect and delete tag jumping events [43] (Fig. 2)....."
L. 575-85, Findings: "...This ensures that unlabelled PCR products are never produced and allows us to multiplex a large number of samples on a single run of Illumina MiSeq run. Just 24 sample tags 1 and 20 plate tags 2 allow the differentiation of up to 480 samples with matching tags on both ends. The same number of individual primers would have needed longer tags to maintain enough distance between them and would have resulted in an even longer adapter-tag overhang compared to primer length. This would have most likely resulted in lower binding efficiencies due to steric hindrances of the primers. Furthermore, this would have resulted in increased primer costs. Thus our approach reduced sequencing and primer purchase costs while at the same time largely eliminating sample mis-assignment via tag jumping, because tag-jump sequences have non-matching forward and reverse tag 1 sequences [43]......"
L. 601-05, Findings: "...However, we would not be able to detect a contamination prior the second PCR from one plate to another, as we used the same 24 tags (tag 1) for all plates. Nevertheless such a contamination is very unlikely to result in any accepted false positive as it would be improbable to contaminate both the A and B replicates, given the exchange of all reagents and the time gap between the PCRs...."
Although we do not necessarily see important caveats we highlighted now that the costs to purchase the long oligos are more expensive and that the laboratory analysis might be more time consuming, as each sample requires an individual PCR master mix. Besides this we actually see only advantages of the double twin-tagging (see l. 591-99)

Powered by Editorial Manager® and ProduXion Manager® from Aries Systems Corporation

1) This approach *does* potentially reduce contamination risk as compared to two-round PCR metabarcoding protocols where the first round of amplification is done with tailed, unlabeled primers (or when adaptors are ligated). However, if you re-use first-round indices for multiple libraries, you will generate PCR products with the *same* labels in the first round of PCR. Perhaps this issue may be somewhat mitigated by preparing libraries in batches so that no libraries with the same indexing primers are prepared simultaneously. This caveat probably also applies to the discussion in Line 545. If you re-use first-round indices for multiple projects, PCR products from one study can show up in another. Re-using indexing primers seems highly likley given the expense of long, purified oligos - it doesn't seem affordable to use the first-round primers for only a single library prep. The risk *is* probably lower (because 1/25 libraries have that index, as opposed to 25/25 when unlabeled), but it's not completely unambiguous.
-->Yes, we can never rule out the risk of contamination in the lab, we can only try to minimize it. One option would have been to increase the number of available tags, but this would result –as said by the reviewer- in an increase in costs and in an even longer adapter and tag overhang compared to primer length. This might as well result in lower binding efficiencies due to steric hindrances. Being limited from this side we decided to go for the two-round PCR. At the moment we have about 1200 leech samples so there is no way not to re-use a tag for another sample. However, we tried to minimize contamination risk by lab-workflow measures. As the reviewer suggested, we never used tags simultaneously, we used always 24 different t1 tags on one PCR plate. The only chance would be a contamination of amplicons from the first PCR round to another PCR plate prior the second PCR round. Our laboratory workflow tried to minimize that risk. But even if we have a contamination of a PCR product of the first round, it would have to have contaminated at least two replicates of the same sample, which seems unlikely to us, as we took care to use fresh reagents for each PCR replicate.

2) I am not convinced by the current description that this approach allows removal of chimeric sequences. However, my uncertainty may largely stem from my confusion about what you mean by "chimeric sequence". My understanding is that a "chimera" or "chimeric sequence" is a single molecule that came from two different transcripts. For example, an incompletely-extended PCR product anneals to and extends on a similar, but different template from the original. Resulting reads reflect a composite sequence formed by PCR.
Such a "chimeric sequence" that forms *within* a single library cannot be detected based on paired index sequences. All of the PCR products have the same index sequences on each both ends. Thus, a chimera formed between species A and species B is indistinguishable from a PCR product from species A based on the index sequences alone. I don't think that this is the type of "chimeric sequence" that you're worried about, but it can affect taxonomic assignment (perhaps the authors can explain the sensitivity of PROTAX to these types of errors).
The other type of chimeric sequence that is more problematic is when a molecule has an index for library #1 on one end and an index for library #2 on the other. If you have double-indexed libraries with only one P5/P7 combination per library, then you can remove reads from these PCR products. I think this is the type of chimeric sequence the authors are concerned about? In which case, I'm a bit confused about two points: First, how is it possible to form physical chimeras if each library is amplified by itself and pooled only for sequencing? My understanding is that incorrectly-tagged reads from this protocol come from sequencing errors on the flow cell, rather than being due to the presence of chimeric molecules. Maybe carefully distinguishing between sequences (molecules) and reads (MiSeq output) would help me to track with you. Second why would two-rounds of indexing be better at detecting these types of errors than a single round? Can you show me with a cartoon on Figure 2?
-->You are right; our wording was not precise enough in this context. Real chimeric sequences, as you describe them, cannot be detected within a single sample. If they

occur they are, however, very unlikely to result in any proper assignment. We were talking of reads with different tags on both ends and -as the reviewer points out correctly- the two-rounds PCR does not help to detect them. Only the twin-tagging described here allows the identification of such non-matching tags. Such reads have been observed before (Schnell et al. 2015) and they increase the risk of mis-identification of reads. Hypotheses to explain their occurrence are so far that such “tag jumps” happen during blunt-ending of indexed amplicons or during bulk amplification during library index PCR (see Schnell et al. 2015). We see this hypothesis confirmed by that fact that tag-jumps increased if we pooled the samples prior to the second PCR. Thus the bulk amplification increased the tag-jump rate drastically. However both explanations fail for our described protocol. Thus we were quite surprised to see, nonetheless, 2.5% reads with non-matching tags. We see only two potential explanations here, both would require further testing; first contaminated primers or second, mixed clusters during sequencing. However, we exchanged the word chimeric to non-matching to avoid any confusion.

More minor comments:

Line 187: Later you report a range of values for percent reads from Mammalia, so these must be 58 individually-indexed libraries? How were the libraries prepared (e.g., shearing, indexing, how was quantity assessed for pooling)? Bioinformatics for these unclear. We assume there was some quality filtering steps and rules associated with assignment? If your goal is to assess enrichment success with PCR, would you want to use a comparable pipeline across this experiment and the amplified libraries?
--> We apologize that we have not provided the details about the shot-gun sequencing. We rather saw this as additional data. However for completeness we have now added the protocols and details. The section reads now:
L. 440-450: “...As the success of the metabarcoding largely depends on the mammal DNA quantity in our leech bulk samples we quantified the mammalian DNA content in a subset of 58 of our leech samples using shotgun sequencing. Extracted DNA was sheared with a Covaris M220 focused-ultra-sonicator to a peak target size of 100-200 bp, and re-checked for size distribution. Double-stranded Illumina sequencing libraries were prepared according to a ligation protocol designed by Fortes and Paijmans [51] with single 8 nt indices. All libraries were pooled equimolarly and sequenced on the MiSeq using the v3 150-cycle kit. We demultiplexed reads using bcl2fastq and cutadapt for trimming the adapters. We used BLAST search to identify reads and applied Metagenome Analyzer MEGAN [30] to explore the taxonomic content of the data based on the NCBI taxonomy. Finally we used KRONA [52] for visualisation of the results. ...”

Line 191: Would be helpful to justify these primer sets a bit. Why would we expect them to be suitable for this application?
--> We added the following justification to the manuscript:
L. 285-88: “...The primers were chosen on the expectation of successful DNA amplification over a large number of tetrapod species [41; 42], and we tested the fit of candidate primers on an alignment of available mitochondrial sequences of 134 Southeast-Asian mammal species. Primer sequences are in Table 1 …”

Line 253: Spell out acronym on first use.
-->It seems like MIDORI is not an acronym (although it sounds like one) but a name and we have found inconsistent writing of it in the according papers (Machida et al. 2017, Leray et al. 2018). In Japanese it seems to be the word for “green” thus we assume it is just a name and changed it to “Midori” throughout the manuscript.

Line 266: If there are 7 previously unpublished mitochondrial genomes, why are there

Powered by Editorial Manager® and ProduXion Manager® from Aries Systems Corporation

	13 Accession Numbers here? Are these GenBank Accession Numbers? Entering a few of them into GenBank did not result in any sequences.<br>-->For Mustela nuidpes and Nesolagus timminsi we only uploaded the 12S, 16S, COI and CytB fragments that were part of our reference database (8 Acc.No.). The full mitogenomes of these two species are part of studies of collaborators and thus we could not release the full mitogenoms. For Herpestes semitorquatus, Diplogale hosei, Hemigalus derbyanus, Viverra tangalunga, Paradoxurus jerdoni we uploaded complete or almost complete mitogenomes (5 Acc.No.). During submission the GenBank entries were still held confidential but we released them now.<br>In addition to the sequences submitted to Genbank, fastq files from the sequencing runs for each leech sample are uploaded to the European Nucleotide Archive ENA and can be found via the study accession number ERP109441.<br><br>Line 485: Not sure which 554 species this is. I thought we were talking about the 103 species expected in the sampling area.<br>--> Thank you for reading so carefully, it is in fact a mistake and we apologize for it. The correct number is 55, we corrected it.<br><br>It was not super clear - when a locus did not amplify (checked via gel electrophoresis), did you drop those PCR products from the library pool? Were all amplicons pooled equimolarly (you say "samples" here)?<br>-->First we tried not to drop the samples from the library pool in order not to miss something in case the staining did not work correct. But including these “DNA-free” samples resulted in much diluted libraries, which could not be sequenced as a certain molarity is needed for sequencing. Thus we needed to exclude those samples after attempts failed to up-concentrate the libraries (a step which might also bring additional risks of contamination). We pooled the amplicons of a genetic marker equimolarly, so all A1-16S PCR products were pooled equimolarly together, all A2-16S PCR products.... We clarified in the manuscript which amplicons were pooled together. This section reads now:<br>L 336-42: “....Sequencing libraries were made by equimolar pooling of all positive amplifications; final concentrations were between 2 and 4 nmol. Because of different amplicon lengths and therefore different binding affinities to the flow cell, 12S and CytB products were combined in a single library, whereas positive 16S products were always combined in a separate library. Apart from our negative controls, we did not include samples that did not amplify, as this would have resulted in highly diluted libraries. Up to 11 libraries were sequenced on each run of Illumina MiSeq, following standard protocols....”<br><br>How did you make the list of 103 mammal species known to be present? Why is Homo sapiens not in this list?<br>-->We are working on Borneo for more than ten years, mostly biodiversity studies based on camera trapping, thus we have accumulated extensive knowledge about the occurrence of species within our study site. For smaller mammalian groups we used additional information from collaborators working in Sabah and for species without existing knowledge about their distribution we included these species in the list. In fact Homo sapiens and our Myodes glareolus, our positive control, were added also to the prior list. So in fact it was 103 Bornean species plus human and bank vole. We apologize for causing confusion on this and have changed the lines 185-187 accordingly. However we eliminated Homo sapiens from the final results as we could not exclude that this was contamination during collection of leeches.<br><br>Structure: In the Methods section, the lab work comes first, in the Results/Discussion, the database construction comes first. Consider selecting a structure that is repeated throughout the paper and use corresponding sub-headers to help the reader track the flow throughout.

Powered by Editorial Manager® and ProduXion Manager® from Aries Systems Corporation

-->We apologize for this. The reason was that we wanted to start with the samples and this is followed by the lab procedure and later only the bioinformatics methods (the information about the study site given in the description of the samples is required for this). However, in the results we actually first needed to have the bioinformatics to then present the results of the laboratory work. We realized however that this is confusing and now have changed the order. The new order in the method section is now that the database curation comes first, followed by the lab work, followed by the bioinformatics on read processing and taxonomic assignment.

I was a bit confused - why was COI of interest, and what portion of it was of interest, if it wasn't one of the three loci in the empirical work?
-->COI is one of the standard markers for metabarcoding and particularly widely used for invertebrates. Therefore we initially wanted to use COI as a fourth marker. But it turned out that the primers did not perform reliable during PCR thus we stopped using COI. But as we had the database already built we decided to publish this together with the bioinformatics pipeline, as we expect that other studies might use this additional marker.

Figure 5: Colors are too close for differentiating loci. Consider simply labeling the rows.
-->We changed this figure using a consistent color code for all four markers for all figures. We the colors are discriminable enough we are happy to label the rows if necessary.

Figure 6: Small points make figure difficult to read.
-->Thank you for the comment. We changed that figure to a barplot using a consistent color code for the four genetic markers for all figures. We hope it improved.

-->cited literature:
Ficetola et al.. Replication levels, false presences and the estimation of the presence/absence from eDNA metabarcoding data. Mol Ecol Res. 2014; 15(3): 543-56.
Lahoz-Monfort et al.. Statistical approaches to account for false-positive errors in environmental DNA samples. Mol Ecol Res. 2015; 16: 673-85.
Leray et al.. MIDORI server: a webserver for taxonomic assignment of unknown metazoan mitochondrial-encoded sequences using a curated database. Bioinformatics 2018; 1: 2.
Machida et al.. Metazoan mitochondrial gene sequence reference datasets for taxonomic assignment of environmental samples. Sci Data. 2017; 4: 170027.
Nilsson & Ryberg. Taxonomic Reliability of DNA Sequences in Public Sequence Databases: A Fungal Persepctive. PLoS ONE. 1, 2006; 1.
Rodgers et al.. Carrion fly-derived DNA metabarcoding is an effective tool for mammal surveys: Evidence from a known tropical mammal community. Mol Ecol Res. 2017; 17(6):1-13
Schnell et al.. Tag jumps illuminated–reducing sequence-to-sample misidentifications in metabarcoding studies. Mol Ecol Res. 2015; 15(6): 1289-1303.
Schnell et al.. Debugging diversity - a pan-continental exploration of the potential of terrestrial blood-feeding leeches as a vertebrate monitoring tool. Mol Ecol Res. 2018.
Zepeda-Mendoza et al.. DAMe: a toolkit for the initial processing of datasets with PCR replicates of double-tagged amplicons for DNA metabarcoding analyses. BMC Res Notes 2016; 9.

Reviewer #2:
Despite the advent of high throughput sequencing, incorporating this technology

Powered by Editorial Manager® and ProduXion Manager® from Aries Systems Corporation

particularly for confident detection of rare species via iDNA/eDNA is still technically challenging. Hence, I applaud the authors for taking the initiave to investigate and tackle this problem.
There are a lot of variables for this sort of work that are not likely to be fully addressed in a single manuscript so I do not fault the authors for not being overly comprehensive in doing benchmarks. However, I feel there are a few additional works to need be done prior to making a recommendation that potentially will change or pave the way forward for such study.

1. Performing an iniail analysis on a mock community - will this be a good start rather than diving straight into to real test?
-->Our manuscript should be viewed in the context that the community has learned a great deal about the errors that arise in metabarcoding (e.g. Schnell et al. 2015, Somervuo et al. 2016, Evans et al. 2017, Racimo et al. 2016) and what can be about those errors (e.g. Zepeda-Mendoza et al. 2016, Somervuo et al. 2017, Rodgers et al. 2017). Thus, while an analysis of a mock community would be useful to show the problems, these problems and their solutions are largely known. The challenge now is to design cost-effective pipelines that use these solutions systematically to mitigate these known sources of error. Our workflow was developed out of the need to analyse over 1000 samples with over 7500 leeches. We initially started published methods but experienced both contamination and/or tag jumping issues. Our use of double twin-tagging solves the tag-jump problem at a relatively low cost (compared to buying, say, a hundred+ pairs of twin-tagged primers). And our curation and Protax pipeline confronts known problems of overconfidence and outright error in taxonomic assignment.
Thus, we think the workflow will be of value for other researchers facing the same problems as we did and we fully expect other researchers to alter and even improve our workflow. To facilitate this, we are using this manuscript to make all our protocols and scripts available. Due to the exponential increase in e/iDNA metabarcoding studies, we think that our manuscript will be of great interest for many researchers. Nevertheless we have now added additional quantifications on false negative and false positive rates (l. 418-30 & l. 543-561, supplemental figure 1), comparison of our results if we had used a more relaxed acceptance criteria (l. 648-61, table 5) and comparisons to other approaches as far as this was possible.

2. mtDNA markers are of diff fragment size, with that you will have a few variables .
-primer binding efficiency should be tested in-silico for a start, could that be why taxonomic assignment/recovery was affected
- Would it be a good time to design a more suitable primers with in-silico validation for such work? Tetrapod specific but targeting more variable region and with a amplicon size that give better compromise between PCR recovery of high degraded DNA and taxonomic resolution.
--> The reviewer raised important issues and challenges here and we absolutely agree to his concerns. We were also worried on the performance of our primer system and tried to validate their performance using real-time PCR. We tested qPCR conditions on a BioRad plate Cycler and cycling conditions were 5min at 95°C, followed by 50 cycles of 30s at 95°C, 30s at 54°C, and 45s at 72°C with a detection at the end of every cycle and a final extension of 5 min at 72°C. This was followed by a melt curve analysis from 60° to 95°C in 0.5°C steps. As you can see from the example below not all samples performed well and all came very late (> 30 cycles) which means it was impossible to run dilution series to estimate the PCR efficiency. Instead we used LinRegPCR (Ramakers et al., NeuroSci Lett 2003) to calculate the individual reaction efficiencies based on the amplification curve. This might give only a rough estimate of the PCR efficiency but they were all comparably well in a range of 1.8 to 2. However, the variance between the replicates of the tested samples in the qPCR was quite high due to the late fluorescence signal and as the estimated efficiencies were normal we believe that the amount of target DNA in a sample is much more important than the PCR performance of the primers.
At the same time our results clearly showed that amplification success and sequencing quality for the long PCR fragments (12S and Cytb) was much lower than for the short 16S marker (l. 560-67, suppl. table 3). While this reinforces the fact that DNA in leeches is highly degraded, it also means that designing shorter Cytb and 12S primers

might be a useful next step forward. We highlighted this in the manuscript in line 569-74, which reads: “...Despite the greater taxonomic resolution of the longer 12S and CytB fragments, our poorer amplification and sequencing results for these longer fragments emphasize that e/iDNA studies should generally focus on short PCR fragments to increase the likelihood of positive amplifications of the degraded target DNA. In the case of mammal-focussed e/iDNA studies, developing a shorter (100 bp) CytB fragment would likely be very useful. ...”

3. the cytb gene seem to be the most promising given its high representation for mammal, so that could be a good candidate for primer design to generate a smaller amplicon than its current primer pairs.
--> Yes, absolutely correct and we are certain that is could improve the workflow. But it is a challenging task to find primers that fulfill the conditions and amplify over such a large number of taxa. At this point we were unable to test additional primers, but hope that with the publication of this manuscript, other groups will be inspired to work on this. However, this does not affect the current workflow and one could also use more taxon-specific primers.

4. Authors should show the number of reads generated for each sample and also the number of reads per marker because sequencing depth obviously will have an effect as well on the detection sensitivity.
--> One of the outputs of the pipeline is a tab delimited txt-file reporting on the read numbers for each marker per sample and that were processed in the various steps of the read-preprocessing.sh script. This includes the raw number of reads, merging of R1/R2 reads, primer clipping and trimming of reads, quality filtering and dereplication. We added a note on this in line 363-65. Furthermore we summarized these results for all eight sequencing runs in the supplemental table 3. We also summarized the number reads per sample that entered the taxonomic assignment for each of the eight sequencing runs (supplemental table 4). In the main body of the text we refer to these results in lines 566-69 and lines 665-68.

5. Seems like authors used Ampli-Gold Taq instead of a high fidelity polymerase (such as but not limited to KAPA HIFI, Q5 polymerase)for their amplification. higher taq polymerase error rate coupled with the use of dereplication without error-correction is likely going to generate way more unique reads. So will using a proof reading high fidelity polymerase followed by chimera removal and error correction bioinformatic tool e.g. UNOISE3 be useful in eliminating spurious product from high amplification cycle?
-->This is true; a high-fidelity tag polymerase would decrease the noise signal around the real sequence. But it is a question if this lower noise signal affects the final assignment much in the end and if it is worth the additional costs, given that we are already using replication (and usearch filters) to filter out erroneous amplicons. Specifically, we don’t expect chimeric sequences to be a problem because it is very unlikely that they result in a believable and high-confidence taxonomic assignment, given the very low probability that the same chimeric sequence would occur in two replicates of the same sample in the same way and thus it would never be accepted as a true positive.
The price for high fidelity taq polymerase is about twice the one for hot start polymerase, which goes against one of our goals, which is to increase cost-efficiency for large-scale biodiversity screenings. With over 1000 leech samples we have over 24,000 PCR reactions. Doubling the tag costs would certainly make it very difficult to implement such large scale projects. We have worked with high fidelity Taq in other projects and recognized the advantages, but for this work, and given our use of PCR and marker replicates, we do not believe that our assignment results would improve in a way which would justify these additional costs.

6. The read2 from MiSeq 600 cycle kit is known to be very poor in quality towards the

Powered by Editorial Manager® and ProduXion Manager® from Aries Systems Corporation

end and that will affect overlap for the bigger fragment, authors might want to look into this and do proper trimming.
--> Yes, we are aware of this problem and addressed these issues in lines 356-365: “...In all cases, amplicons were short enough to expect paired reads to overlap. For libraries with more than 1000 reads pairs were merged with usearch (-fastq_mergepairs; [47; 48]), and only successfully merged pairs were retained. For libraries with more than 500 merged pairs the primer sequences were trimmed away with cutadapt [49], and only successfully trimmed reads at least 90% of expected amplicon length were passed to a quality filtering step with usearch (-fastq_filter). Lastly, reads were dereplicated with usearch (-derep_fulllength), and singletons were discarded. The number of replicates that each unique sequence represented was also added to the read header at this step (option -sizeout). The number of reads processed at each step for each sample are reported in a standard tab delimited txt-file. ...”,
as well as in lines 566-69: “...Also the read losses due to trimming and quality filtering were significantly lower for the 16S sequencing runs (1.3% and 5.3% in average, Supplemental Table 3) compared to the sequencing runs for the longer fragments of 12S and CytB (65.3% and 44.3% in average, Supplemental Table 3). ...”
Because low quality reads were excluded with usearch we do not believe that read quality causes severe problems in our study. However, Taberlet et al. 2007 demonstrated that the presence of a homopolymer in a metabarcoding sequence leads to a systematic decrease of read quality. Thus average quality can change consistently from one species to another and they argue that quality based trimming and filtering might bias diversity estimates in metabarcoding samples to an unknown extant (see in Bonin et al. 2018).

7. From 6, it will be good to how # of reads generated then # of reads lost after each QC/overlap/binning.
--> Thank you for the comment, for trimming and merging of reads we use already well-established standard tools for post-run processing of Illumina sequencing reads (see our answer to your point 6). As we already said we have added the supplemental tables 3 reporting on the read losses during read processing. However, given length limits, we think it is much more important to focus on how we avoided contamination and false positives and ended up with a species list that stakeholders can trust and base decisions on. That we hope to find your agreement to leave those tables in the supplement as they refer more to sequencing quality than metabarcoding.

8. Can the authors also normalize the final reads prior to comparing their sensitivity/efficiency in recovering species? My concern is that some species was not recovered because of low sequencing depth in one of the replicates.
--> Regarding the sensitivity in recovering species we added some simulation data on false negative rates for varying detection probabilities (Supplemental Figure 1) and added another acceptance criteria (l. 418-30) more discussion on this topic in lines 543-574.
We understand the reviewers concern on non-detection due to missing sequencing depth. We analysed 242 samples and only pooled the PCR with a positive PCR equimolar together, so each sample in the sequenced library should contribute with the same amount of DNA to the pool. Even if all 242 samples would have been positive, if we assume 20 million reads from an Illumina V3 kit and if we lose 30% to spiked in PhiX control, we would end up with 14,000,000 reads/242 samples = 57,851 reads/sample. Thus we are less worried about sequencing depth for our study. However, to address this concern we provided now an additional table on the number of reads per sample, as requested in point 4 (Supplemental Table 4). Although we understand the reviewer’s concern on imperfect detection due to low read numbers per sample, it is unfortunately not feasible to adjust sequencing depth to individual samples of unknown content in a high-throughput workflow. The risk of an imperfect detection and such false negatives is always given in metabarcoding studies. However, our lowest median read number per sample per sequencing run was 8218 reads, which we think should be sufficient. Furthermore read numbers are largely influenced by PCR stochasticity and we believe that reproducibility is the more reliable factor for species detection than read numbers.

Powered by Editorial Manager® and ProduXion Manager® from Aries Systems Corporation

9. What sort of sequening depth would be recommended for detection of low abudance/rare species? What if increasing sequenicng depth to 1 million reads allow recovery ? It will be good to have a spike-in DNA as control to check that out coupled with qPCR + tqman probe to compare sensitivity. The last thing we want is making claims on species presence/absence based on a specific sequencing depth and without complementary data to support. Absence of evidence sometimes is not evidence of absence and this is really one of the biggest challenges in using NGS for such work - how deep is deep enough?
--> The main consideration for not using a spike-in is that such DNA is high quality and thus likely to outcompete our degraded target DNA in the PCR reaction, leading to even lower detection probabilities. DNA degradation varies to such a great extent in the leech samples that any spike-in experiment even if we use degraded ancient DNA could never represent the complexity of a bulk leech sample. We therefore decided to accept this trade off and accept that some false negative will occur in the dataset. As outlined above such false negatives are incorporated in the detection probability estimation of the hierarchical occupancy models.
Increasing sequencing depth is another method for increasing detection, but sticking to the example with 1 million reads, we could sequence then 20 PCRs in one MiSeq run with 20 million reads. Even if only one third of our over 24,000 PCRs contains target DNA this would mean we would have to run 400 sequencing runs and one 600 cycle V3 kit is about 1500,- €...
We very much agree that there are options to improve detectability, but for large scale screening we are soon facing severe trade-offs in terms of costs and time.

10. Regarding the 103 mammal species. Are the authors assuming that their species designation is correct? And will it be correct to assume that leech will feed on all 103 mammal species in general? Also mtDNA database of the actual species sequenced from the location will be good. Borneo is an isolated island, there might be potentially new species despite morphological similarity to known species.
--> The 103 species are currently accepted species. That said, there will certainly be cryptic species/subspecies on Borneo. Our team has been involved in numerous phylogenetic studies in the region and is aware of these difficulties. You are right that genetic reference from the particular research area would be desirable to reflect geographic difference. But we simply have to work with what we have and hope that the intraspecific variance is still lower than the interspecific one.
In the course of this study, we helped to build a mitogenomic database of Southeast Asian mammals and we published in 2017 in Gigascience 57 additional mitogenomes. In addition, as a part of this manuscript we have released additional mitogenomes and mtDNA sequences. We realize the need for additional mitogenome sequencing and barcoding effort (as we say in l. 503-505 of the manuscript). However, obtaining samples, particularly of rare and endemic species is extremely difficult (both in terms of time and permission), and thus there will be always some questions in respect to the actual species designation. As said above the 103 species were selected based on over 10 years of field research in Sabah Malaysia and together with a number of long-term scientists working in Sabah. It is of course unknown if leeches feed on all of these 103 species (we are the first study looking at leeches from our sites), but it is important to include all potential species in the list to ensure that PROTAX does not assign a read to a sister species, just in consequence that the “true” species was not included.

11. "As the costs of HTS decrease, we expect that such gap-filling will increasingly shift towards whole mitochondrial genomes [36], reducing the effect of marker choice on detection likelihood " - I think authors need to tone down on this. Even with the Novaseq6000, the depth required for sequencing just to recover whole mtDNA will be hard because first, it will be the leech DNA/genome that will dominate the data. Second, from the remaining non-host reads, the chance of mtDNA recovery will depend on how many diff species of mammals the leech has attached to previously. More importantly, blood in genera is relatively low in mtDNA unlike muscle.

	--> Sorry for the misunderstanding here. We did not mean that leeches could be used to construct mammalian mitogenomes. We meant that with the decreasing costs in HTS, there will hopefully be additional reference mitogenomes in the future including more species and covering larger geographical areas. We have rephrased this section: l. 505-08: "...As the costs of HTS decrease, we expect that such gap-filling will increasingly shift towards sequencing of whole mitochondrial genomes of specimen obtained from museum collections, trapping campaigns etc. [34], reducing the effect of marker choice on detection likelihood....."<br><br>12. I also couldn't find the term "SRA" in the manuscript so I wonder if the data has been submitted to NCBI database.<br>Fastq files from the sequencing runs for each leech sample are uploaded to the European Nucleotide Archive ENA and can be found via the study accession number ERP109441. https://www.ebi.ac.uk/ena/data/view/ERP109441<br><br>--> cited literature:<br>Bonin et al.. Environmental DNA: For Biodiversity Research and Monitoring. 1st ed. Oxford University Press; 2018.<br>Evans et al. Fish community assessment with eDNA metabarcoding: effects of sampling design and bioinformatic filtering. Can J Fish Aquat Sci. 2017; 74(9):, 1362-74.<br>Racimo et al. Joint estimation of contamination, error and demography for nuclear DNA from ancient humans. PLoS Genet. 2016; 12(4): e1005972.<br>Rodgers et al.. Carrion fly-derived DNA metabarcoding is an effective tool for mammal surveys: Evidence from a known tropical mammal community. Mol Ecol Res. 2017; 17(6):1-13<br>Schnell et al.. Tag jumps illuminated–reducing sequence-to-sample misidentifications in metabarcoding studies. Mol Ecol Res. 2015; 15(6): 1289-1303.<br>Schnell et al.. Debugging diversity - a pan-continental exploration of the potential of terrestrial blood-feeding leeches as a vertebrate monitoring tool. Mol Ecol Res. 2018.<br>Somervuo P et al.. Unbiased probabilistic taxonomic classification for DNA barcoding. Bioinformatics. 2016; 32(19): 2920-7.<br>Somervuo et al.. Quantifying uncertainty of taxonomic placement in DNA barcoding and metabarcoding. Methods Ecol Evol. 2017; 8(4): 398-407.<br>Zepeda-Mendoza et al.. DAMe: a toolkit for the initial processing of datasets with PCR replicates of double-tagged amplicons for DNA metabarcoding analyses. BMC Res Notes 2016; 9.
**Additional Information:**	
**Question**	**Response**
Are you submitting this manuscript to a special series or article collection?	No
**Experimental design and statistics**<br><br>Full details of the experimental design and statistical methods used should be given in the Methods section, as detailed in our Minimum Standards Reporting Checklist. Information essential to interpreting the data presented should be made available in the figure legends.<br><br>Have you included all the information requested in your manuscript?	Yes

**Resources**<br><br>A description of all resources used, including antibodies, cell lines, animals and software tools, with enough information to allow them to be uniquely identified, should be included in the Methods section. Authors are strongly encouraged to cite Research Resource Identifiers (RRIDs) for antibodies, model organisms and tools, where possible.<br><br>Have you included the information requested as detailed in our Minimum Standards Reporting Checklist?	Yes
**Availability of data and materials**<br><br>All datasets and code on which the conclusions of the paper rely must be either included in your submission or deposited in publicly available repositories (where available and ethically appropriate), referencing such data using a unique identifier in the references and in the "Availability of Data and Materials" section of your manuscript.<br><br>Have you have met the above requirement as detailed in our Minimum Standards Reporting Checklist?	Yes

Powered by Editorial Manager® and ProduXion Manager® from Aries Systems Corporation

Manuscript

Click here to view linked References

Click here to access/download;Manuscript;Leeches_Methods_Database_dr

An efficient and robust laboratory workflow and tetrapod database for larger scale eDNA studies

Jan Axtner[1+], Alex Crampton-Platt[1], Lisa A. Hörig[1], Azlan Mohamed[1], Charles C.Y. Xu[2,3,4], Douglas W. Yu[2,5] and Andreas Wilting[1]

Affiliations:

[1] Leibniz Institute for Zoo and Wildlife Research (*Leibniz-IZW*), Alfred-Kowalke-Str. 17, 10315 Berlin, Germany

[2] State Key Laboratory of Genetic Resources and Evolution, Kunming Institute of Zoology, Chinese Academy of Sciences, 32 Jiaochang East Road, Kunming, Yunnan 650223, China

[3] Groningen Institute for Evolutionary Life Sciences, University of Groningen, P.O. Box 11103, 9700 CC Groningen, The Netherlands

[4] Redpath Museum and Department of Biology, McGill University, Montreal, QC, Canada

[5] School of Biological Sciences, University of East Anglia, Norwich Research Park, Norwich, Norfolk NR47TJ, UK

[+] corresponding author

email addresses:

Jan Axtner:	axtner@izw-berlin.de
Alex Crampton-Platt:	alex@naturemetrics.co.uk
Lisa A. Hörig:	lisa.hoerig@arcor.de
Azlan Mohamed:	mohamed@izw-berlin.de
Charles C.Y. Xu:	charles.cong.xu@gmail.com
Douglas W. Yu:	dougwyu@mac.com
Andreas Wilting:	wilting@izw-berlin.de

Keywords:
metabarcoding, iDNA, eDNA, leeches

Abstract

Background

The use of environmental DNA, ‘eDNA,’ for species detection via metabarcoding is growing rapidly. We present a co-designed lab workflow and bioinformatic pipeline to mitigate the two most important risks of eDNA: sample contamination and taxonomic mis-assignment. These risks arise from the need for PCR amplification to detect the trace amounts of DNA combined with the necessity of using short target regions due to DNA degradation.

Findings

Our high-throughput workflow minimises these risks via a four-step strategy: (1) technical replication with two *PCR replicates* and two *extraction replicates*; (2) using multi-markers (*12S*, *16S*, *CytB*); (3) a ‘twin-tagging,’ two-step PCR protocol;(4) use of the probabilistic taxonomic assignment method *PROTAX*, which can account for incomplete reference databases.

As annotation errors in the reference sequences can result in taxonomic mis-assignment, we supply a protocol for curating sequence datasets. For some taxonomic groups and some markers, curation resulted in over 50% of sequences being deleted from public reference databases, due to (1) limited overlap between our target amplicon and reference sequences; (2) mislabelling of reference sequences; (3) redundancy.

Finally, we provide a bioinformatic pipeline to process amplicons and conduct *PROTAX* assignment and tested it on an ‘invertebrate derived DNA’ (iDNA) dataset from 1532 leeches from Sabah, Malaysia. Twin-tagging allowed us to detect and exclude sequences with non-matching tags. The smallest DNA fragment (*16S*) amplified most frequently for all samples, but was less powerful for discriminating at species rank. Using a stringent and lax acceptance criteria we found 170 (stringent) and 196 (lax) vertebrate detections of 97 (stringent) and 109 (lax) leech samples.

Conclusions

Our metabarcoding workflow should help research groups increase the robustness of their results and therefore facilitate wider usage of e/iDNA, which is turning into a valuable source of ecological and conservation information on tetrapods.

Introduction

Monitoring, or even detecting, elusive or cryptic species in the wild can be challenging. In recent years there has been a rise in the availability of cost-effective DNA-based methods made possible by advances in high-throughput DNA sequencing (HTS). One such method is eDNA metabarcoding, which seeks to identify the species present in a habitat from traces of 'environmental DNA' (eDNA) in substrates such as water, soil, or faeces. A variant of eDNA metabarcoding, known as 'invertebrate-derived DNA' (iDNA) metabarcoding, targets the genetic material of prey or host species extracted from copro-, sarco- or haematophagous invertebrates. Examples include tick [1] s, blow or carrion flies [2; 3; 4; 5], mosquitoes [6; 7; 8; 9] and leeches [10; 11; 12; 13]. Many of these parasites are ubiquitous, highly abundant, and easy to collect, making them an ideal source of biodiversity data, especially for terrestrial vertebrates that are otherwise difficult to detect [10; 14; 15]. In particular, the possibility for bulk collection and sequencing in order to screen large areas and minimise costs is attractive. However, most of the recent studies on iDNA studies focus on single-specimen DNA extracts and Sanger sequencing and thus are not making use of the advances of HTS and a metabarcoding framework for carrying out larger scale biodiversity surveys.

That said, e/iDNA metabarcoding also poses several challenges, due to the low quality and low amounts of target DNA available, relative to non-target DNA (including the high-quality DNA of the live-collected, invertebrate vector). In bulk iDNA samples comprised of many invertebrate specimens, this problem is further exacerbated by the variable time since each individual has fed, if at all, leading to differences in the relative amounts and degradation of target DNA per specimen. This makes e/iDNA studies similar to ancient DNA samples, which also pose the problem of low quality and low amounts of target DNA [16; 17]. The great disparity in the ratio of target to non-target DNA and the low overall amount of the former requires an enrichment step, which is achieved via the amplification of a short target sequence (amplicon) by polymerase chain reaction (PCR) to obtain enough target material for sequencing. However, this enrichment step can result in false positive species detections, either through sample cross-contamination or through volatile short PCR amplicons in the laboratory, and in false-negative results, through primer bias and low concentrations of template DNA. Although laboratory standards to prevent and control for such false results are well established in the field of ancient DNA, there are still no best-practice guidelines for e/iDNA studies, and thus few studies sufficiently account for such problems [18].

The problem is exacerbated by the use of 'universal' primers used for the PCR, which maximise the taxonomic diversity of the amplified sequences. This makes the method a powerful biodiversity assessment tool, even where little is known *a priori* about which species might be found. However, using such primers, in combination with low quality and quantity of target DNA, which often requires a high number of PCR cycles to generate enough amplicon products for sequencing, makes metabarcoding studies particularly vulnerable to false results [13; 19; 20]. The high number of PCR cycles, combined with the

high sequencing depth of HTS, also increase the likelihood that contaminants are amplified and detected, possibly to the same or greater extent as some true-positive trace DNA. As e/iDNA have been proposed as tools to detect very rare and priority conservation species such as the Saola, *Pseudoryx nghetinhensis* [10], false detection might result in misdirected conservation activities worth several hundreds of thousands of US dollars like for the ivory-billed woodpecker where most likely false evidence of the bird's existence have been overemphasized to shore up political and financial support for saving it [21]. Therefore, similar to ancient DNA studies, great care must be taken to minimise the possibility for cross-contamination in the laboratory and to maximise the correct detection of species through proper experimental and analytical design. Replication in particular is an important tool for reducing the incidence of false negatives and detection of false positives but the trade-off is increased cost, workload, and analytical complexity [19].

An important source of false positive species detections is the incorrect assignment of taxonomies to the millions of short HTS reads generated by metabarcoding. Although there has been a proliferation of tools focused on this step, most can be categorised into just three groups depending on whether the algorithm utilises sequence similarity searches, sequence composition models, or phylogenetic methods [22; 23; 24]. The one commonality among all methods is the need for a reliable reference database of correctly identified sequences, yet there are few curated databases currently appropriate for use in e/iDNA metabarcoding. Two exceptions are SILVA [25] for the nuclear markers SSU and LSU rRNA used in microbial ecology, and BOLD (Barcode of Life Database; citation) for the COI 'DNA barcode' region. For other loci, a non-curated database downloaded from the INSDC (International Nucleotide Sequence Database Collaboration, e.g. GenBank) is generally used. However, the INSDC places the burden for metadata accuracy, including taxonomy, on the sequence submitters, with no restriction on sequence quality or veracity. For instance, specimen identification is often carried out by non-specialists, which increases error rates, and common laboratory contaminant species (e.g. human DNA sequences) are sometimes submitted in lieu of the sample itself. The rate of sequence mislabelling in fungi has been assessed for GenBank where it was up to 20% [26] and it is an issue that is often neglected [27; 28]. For several curated microbial databases (Greengenes, LTP, RDP, SILVA), mislabelling rates have been estimated at between 0.2% and 2.5% [29]. Given the lack of professional curation it is likely that the true proportion of mislabelled samples in GenBank is somewhere between these numbers. Moreover, correctly identifying such errors is labour-intensive, so most metabarcoding studies simply base their taxonomic assignments on sequence-similarity searches of the whole INSDC database (e.g. with BLAST) [3; 10; 12] and thus can only detect errors if assignments are ecologically unlikely. Furthermore, reference sequences for the species that are likely to be sampled in e/iDNA studies are often underrepresented in or absent from these databases, which increases the possibility of incorrect assignment. For instance, fewer than 50% of species occurring in a tropical megadiverse rainforest are represented in Genbank (see findings below). When species-level matches are ambiguous, it might still be possible to assign a sequence to a higher

taxonomic rank by using an appropriate algorithm such as Metagenome Analyzer's (MEGAN) Lowest Common Ancestor [30] or *PROTAX* [31].

We present here a complete laboratory workflow and complementary bioinformatics pipeline, starting from DNA extraction to taxonomic assignment of HTS reads using a curated reference database. The laboratory workflow allows for efficient screening of hundreds of e/iDNA samples. The workflow includes (1) two *extraction replicates* are separated during DNA extraction, and each is sequenced in two *PCR replicates* (Fig. 1); (2) robustness of taxonomic assignment is improved by using up to three mitochondrial markers; (3) a 'twin-tagged', two-step PCR protocol prevents cross-sample contamination as no unlabelled PCR products are produced (Fig. 2) while also allowing for hundreds of PCR products to be pooled before costly Illumina library preparation; (4) our bioinformatics pipeline includes a standardized, automated, and replicable protocol to create a curated database, which allows updating as new reference sequences become available, and to be expanded to other amplicons. We provide scripts for processing raw sequence data to quality-controlled dereplicated reads and for taxonomic assignment of these reads using *PROTAX* [31], a probabilistic method that has been shown to be robust even when reference databases are incomplete [23; 4] (all scripts are available from URL https://github.com/alexcrampton-platt/screenforbio-mbc).

Methods

Establishment of the tetrapod reference database

Reference database

A custom bash script was written to generate a tetrapod reference database for up to four mitochondrial markers – a short 93 bp fragment of *16S* rRNA (*16S*), a 389 bp fragment of *12S* rRNA (*12S*), a 302 bp fragment of cytochrome b (*CytB*), and a 250 bp mitochondrial cytochrome c oxidase subunit I amplicon (*COI*) that has previously been used in iDNA studies [2]. An important time-saving step was the use of the FASTA-formatted Midori mitochondrial database [32], which is a lightly curated subset of Genbank. Our script updated the FASTA files with a subset of target species, removed errors and redundancy, trimmed the sequences to include only the amplicon regions, and output FASTA files with species names and GenBank accessions in the headers.

The script accepts four data inputs, two of which are optional. The required inputs are: (i) the Midori sequences (December 2015 'UNIQUE', downloaded from http://www.reference-midori.info/download.php#) for the relevant genes and (ii) an initial reference taxonomy of tetrapods. This taxonomy is needed to find or generate a full taxonomic classification for each sequence because the taxonomies in Midori are from Genbank and thus include incorrect, synonymized, or incomplete taxonomies. Here we used the Integrated Taxonomic Information System (ITIS) classification for Tetrapoda, obtained with the R package *taxize* version 0.9.0 ([33], functions *downstream* and *classification*). The optional inputs are: (iii) supplementary FASTA files of reference sequences that should be added to the database,

and (iv) a list of target species to be queried on GenBank to capture any sequences published since the December 2015 Midori dataset was generated.

For this study, 72 recently published [34) and 7 unpublished partial mitochondrial mammal genomes (Accession Numbers MH464789, MH464790, MH464791, MH464792, MH464793, MH464794, MH464795, MH464796, MH464797, MH464798, MH464799, MH464800, MH464801) were added as input (iii). A list of 103 mammal species known to be present in the sampling area plus *Homo sapiens* and our positive control *Myodes glareolus* was added as input (iv).

With the above inputs, the seven curation steps are: 1) remove sequences not identified to species; 2) add extra sequences from optional inputs (iii) and (iv) above; 3) trim the sequences to leave only the target amplicon; 4) remove sequences with ambiguities; 5) compare species names from the Midori dataset to the reference taxonomy from input (ii) and replace with a consensus taxonomy; 6) identify and remove putatively mislabelled sequences; 7) dereplicate sequences, retaining one haplotype per species.

The script is split into four modules, allowing optional manual curation at three key steps. The steps covered by each of the four modules are summarized in Table 2. The main programs used are highlighted and cited in the text where relevant, but many intermediate steps used common UNIX tools and unpublished lightweight utilities freely available from GitHub (Table 3).

Module 1 - The first step is to select the tetrapod sequences from the Midori database for each of the four selected loci (input (i) above). This, and the subsequent step to discard sequences without strict binomial species names and reduce subspecies identifications to species-level, are made possible by the inclusion of the full NCBI taxonomic classification of each sequence in the FASTA header by the Midori pipeline. The headers of the retained sequences are then reformatted to include just the species name and GenBank accession separated by underscores. If desired, additional sequences from local FASTA files are now added to the Midori set (input (iii)). The headers of these FASTA files are required to be in the same format. Next, optional queries are made to the NCBI GenBank and RefSeq databases for each species in a provided list (input (iv)) for each of the four target loci, using NCBI's Entrez Direct [35]. Matching sequences are downloaded in FASTA format, sequences prefixed as "UNVERIFIED" are discarded, the headers are simplified as previously, and those sequences not already in the Midori set are added. Trimming each sequence down to the relevant target marker was carried out in a two-step process in which *usearch* (*-search_pcr*) was used to select sequences where both primers were present, and these were in turn used as a reference dataset for *blastn* to select partially matching sequences from the rest of the dataset [36; 37]. Sequences with a hit length of at least 90% of the expected marker length were retained by extracting the relevant subsequence based on the BLAST hit co-ordinates. Sequences with ambiguous bases were discarded at this stage. In the final step in module 1, a multiple-sequence alignment was generated with MAFFT [38; 39] for each partially curated amplicon dataset (for the SATIVA step below). The script then breaks to

allow the user to check for any obviously problematic sequences that should be discarded before continuing.

Module 2 - The species labels of the edited alignments are compared with the reference taxonomy (input (ii)). Any species not found is queried against the Catalogue of Life database (CoL) via *taxize* in case these are known synonyms, and the correct species label and classification is added to the reference taxonomy. The original species label is retained as a key to facilitate sequence renaming, and a note is added to indicate its status as a synonym. Finally, the genus name of any species not found in the CoL is searched against the consensus taxonomy, and if found, the novel species is added by taking the higher classification levels from one of the other species in the genus. Orphan species labels are printed to a text file, and the script breaks to allow the user to check this list and manually create classifications for some or all if appropriate.

Module 3 - This module begins by checking for any manually generated classification files (from the end of Module 2) and merging them with the reference taxonomy from Module 2. Any remaining sequences with unverifiable classifications are removed at this step. The next steps convert the sequences and taxonomy file to the correct formats for SATIVA [29], which detects possibly mislabelled sequences by generating a maximum likelihood phylogeny from the alignment in Module 1 and comparing each sequence's taxonomy against its phylogenetic neighbors. Sequence headers in the edited MAFFT alignments are reformatted to include only the GenBank accession, and a taxonomy key file is generated with the correct classification listed for each accession number. In cases where the original species label is found to be a synonym, the corrected label is used. Putatively mislabelled sequences in each amplicon are then detected with SATIVA, and the script breaks to allow inspection of the results. The user may choose to make appropriate edits to the taxonomy key file or list of putative mislabels at this point.

Module 4 - Any sequences that are still flagged as mislabelled at the start of the fourth module are deleted from the SATIVA input alignments, and all remaining sequences are relabelled with the correct species name and accession. A final consensus taxonomy file is generated in the format required by *PROTAX*. Alignments are subsequently unaligned prior to species-by-species selection of a single representative per unique haplotype. Sequences that are the only representative of a species are automatically added to the final database. Otherwise, all sequences for each species are extracted in turn, aligned with MAFFT, and collapsed to unique haplotypes with *collapsetypes_4.6.pl* (zero differences allowed; [40]). Representative sequences are then unaligned and added to the final database.

iDNA samples

We used 242 collections of haematophagous terrestrial leeches from Deramakot Forest Reserve in Sabah, Malaysian Borneo stored in RNA fixating saturated ammonium sulfate solution as samples. Each sample consisted of one to 77 leech specimens (median 4). In total, 1532 leeches were collected, exported under the permit (JKM/MBS.1000-2/3 JLD.2 (8)

issued by the Sabah Biodiversity Council), and analysed at the laboratories of the Leibniz-IZW.

Laboratory workflow

The laboratory workflow is designed to both minimize the risk of sample cross-contamination and to aid identification of any instances that do occur. All laboratory steps (extraction, pre and post PCR steps, sequencing) took place in separate laboratories and no samples or materials were allowed to re-enter upstream laboratories at any point in the workflow. All sample handling was carried out under specific hoods that were wiped with bleach, sterilized, and UV irradiated for 30 minutes after each use. All labs are further UV irradiated for four hours each night.

DNA extraction

DNA was extracted from each sample in bulk. Leeches were cut into small pieces with a fresh scalpel blade and incubated in lysate buffer (proteinase K and ATL buffer at a ratio of 1:10; 0.2 ml per leech) overnight at 55 °C (12 hours minimum) in an appropriately sized vessel for the number of leeches (2 or 5 ml reaction tube). For samples with more than 35 leeches, the reaction volume was split in two and recombined after lysis.

Each lysate was split into two *extraction replicates* (A and B; maximum volume 600 µl) and all further steps were applied to these independently. We followed the DNeasy 96 Blood & Tissue protocol for animal tissues (Qiagen, Hilden -Germany) on 96 plates for cleanup. DNA was eluted twice with 100 µl TE buffer. DNA concentration was measured with PicoGreen dsDNA Assay Kit (Quant-iT, ThermoFisherScientific, Waltham -USA) in 384-well plate format using an appropriate plate reader (200 PRO NanoQuant, Tecan Trading AG, Männedorf -Switzerland). Finally, all samples were diluted to a maximum concentration of 10 ng/µl.

Two-round PCR protocol

We amplified three mitochondrial markers – a short 93 bp fragment of *16S* rRNA (*16S*), a 389 bp fragment of *12S* rRNA (*12S*), and a 302 bp fragment of cytochrome b (*CytB*). For each marker, we ran a two-round PCR protocol (Figs. 1, 2). The primers were chosen on the expectation of successful DNA amplification over a large number of tetrapod species [41; 42], and we tested the fit of candidate primers on an alignment of available mitochondrial sequences of 134 Southeast-Asian mammal species. Primer sequences are in Table 1.

Primer modification. – We modified primers of the three markers to avoid the production of unlabelled PCR products, to allow the detection and deletion of tag-jumping events [43], and to reduce the cost of primers and library preparation. We used two rounds of PCR. The first round amplified the target gene and attached one of 25 different 'twin-tag' pairs (*tag 1*), identifying the sample within a given PCR. By 'twin-tag,' we mean that both the forward and reverse primers were given the *same* sample-identifying sequence ('tags') added as primer extensions (Fig. 2). The tags differed with a minimum pairwise distance of three nucleotides ([43]; Supplemental Table 1). These primers also contained different forward

and reverse sequences (*Read 1* & *Read 2 sequence primers*) (Supplemental Table 1) to act priming sites for the second PCR round (Fig. 2).

The second round added the Illumina adapters for sequencing and attached one of 20 twin-tag pairs (*tag 2*) identifying the PCR, with a minimum pairwise distance of three [44]. These primers also contained the Illumina P5 and P7 adapter sequences (Fig. 2). Thus no unlabelled PCR products were ever produced, and the combination of *tags 1* and *2* allowed the pooling of up to 480 (=24 X 20) samples in a single library preparation step (one *tag 1* was reserved for controls). Twin tags allowed us later to detect and delete tag jumping events [43] (Fig. 2).

Cycle number considerations. – Because we know that our target DNA is at low concentration in the samples, we are faced with a trade-off between (1) using fewer PCR cycles (e.g. 30) to minimise amplification bias (caused by some target DNA binding better to the primer sequences and thus outcompeting other target sequences that bind less well [45]) and (2) using more PCR cycles (e.g. 40) to ensure that low-concentration target DNA is sufficiently amplified in the first place. Rather than choose between these two extremes, we ran both low- and high-cycle protocols and sequenced both sets of amplicons.

Thus, each of the two *extraction replicates* A and B was split and amplified using different cycle numbers (*PCR replicates* 1 and 2) for a total of four (= 2 *extraction replicates* x 2 *PCR replicates* -> *A1*/*A2* and *B1*/*B2*) replicates per sample per marker (Fig. 1). For *PCR replicates* A1/B1, we used 30 cycles in the first PCR round to minimize the effect of amplification bias. For *PCR replicates* A2/B2, we used 40 cycles in the first PCR round to increase the likelihood of detecting species with very low input DNA (Fig. 1).

PCR protocol. – The first-round PCR reaction volume was 20 µl, including 0.1 µM primer mix, 0.2 mM dNTPs, 1.5 mM $MgCl_2$, 1x PCR buffer, 0.5 U AmpliTaq Gold™ (Invitrogen, Karlsruhe - Germany), and 2 µl of template DNA. Initial denaturation was 5 minutes at 95°C, followed by repeated cycles of 30 seconds at 95°C, 30 seconds at 54°C, and 45 seconds at 72°C. Final elongation was 5 minutes at 72°C. Samples were amplified in batches of 24 plus a negative (water) and a positive control (bank vole, *Myodes glareolus* DNA). All three markers were amplified simultaneously for each batch of samples in a single PCR plate. Non-target by-products were removed as required from some *12S* PCRs by purification with magnetic Agencourt AMPure beads (Beckman Coulter, Krefeld -Germany).

In the second-round PCR, we used the same PCR protocol as above with 2 µl of the product of the first-round PCR and 10 PCR cycles.

Quality control and sequencing

Amplification was visually verified after the second-round PCR by gel electrophoresis on 1.5% agarose gels. Controls were additionally checked with a TapeStation 2200 (D1000 ScreenTape assay, Agilent, Waldbronn -Germany). All samples were purified with AMPure beads, using a bead-to-template ratio of 0.7:1 for *12S* and *CytB* products, and a ratio of 1:1 for *16S* products. DNA concentration was measured with PicoGreen dsDNA as described

above. Sequencing libraries were made by equimolar pooling of all positive amplifications; final concentrations were between 2 and 4 nmol. Because of different amplicon lengths and therefore different binding affinities to the flow cell, *12S* and *CytB* products were combined in a single library, whereas positive *16S* products were always combined in a separate library. Apart from our negative controls, we did not include samples that did not amplify, as this would have resulted in highly diluted libraries. Up to 11 libraries were sequenced on each run of Illumina MiSeq, following standard protocols. Libraries were sequenced with MiSeq Reagent Kit V3 (600 cycles, 300 bp paired-end reads) and had a final concentration of 11 pM spiked with 20 to 30% of PhiX control.

Bioinformatics workflow

Read processing

Although the curation of the reference databases is our main focus, it is just one part of the bioinformatics workflow for e/iDNA metabarcoding. A custom bash script was used to process raw basecall files into demultiplexed, cleaned, and dereplicated reads in FASTQ format on a run-by-run basis. All runs and amplicons were processed with the same settings unless otherwise indicated. *bcl2fastq* (Illumina) was used to convert the basecall file from each library to paired-end FASTQ files, demultiplexed into the separate PCRs via the *tag 2* pairs, allowing up to 1 mismatch in each *tag 2.* Each FASTQ file was further demultiplexed into samples via the *tag 1* pairs using *AdapterRemoval* [46], again allowing up to 1 mismatch in each tag. These steps allowed reads to be assigned to the correct samples.

In all cases, amplicons were short enough to expect paired reads to overlap. For libraries with more than 1000 reads pairs were merged with *usearch* (*-fastq_mergepairs*; [47; 48]), and only successfully merged pairs were retained. For libraries with more than 500 merged pairs the primer sequences were trimmed away with *cutadapt* [49], and only successfully trimmed reads at least 90% of expected amplicon length were passed to a quality filtering step with *usearch* (*-fastq_filter*). Lastly, reads were dereplicated with *usearch* (-*derep_fulllength*), and singletons were discarded. The number of replicates that each unique sequence represented was also added to the read header at this step (option -*sizeout*). The number of reads processed at each step for each sample are reported in a standard tab delimited txt-file.

Taxonomic assignment

The curated reference sequences and associated taxonomy were used for *PROTAX* taxonomic assignment of the dereplicated reads [24; 31]. *PROTAX* gives unbiased estimates of placement probability for each read at each taxonomic rank, allowing assignments to be made to a higher rank even when there is uncertainty at the species level. In other words, and unlike other taxonomic assignment methods, *PROTAX* can estimate the probability that a sequence belongs to a taxon that is not present in the reference database. This was considered an important feature due to the known incompleteness of the reference databases for tetrapods in the sampled location. As other studies have compared *PROTAX*

with more established methods, e.g. MEGAN [30] (see [4; 24]), it was beyond the scope of this study to evaluate the performance of *PROTAX.*

Classification with *PROTAX* is a two-step process. Firstly, PROTAX selected a subset of the reference database that was used as training data to parameterise a *PROTAX* model for each marker, and secondly, the fitted models were used to assign four taxonomic ranks (species, genus, family, order) to each of the dereplicated reads, along with a probability estimate at each level. We also included the best similarity score of the assigned species or genus, mined from the LAST results (see below) for each read. This was helpful for flagging problematic assignments for downstream manual inspection, i.e. high probability assignments based on low similarity scores (implying that there are no better matches available) and low probability assignments based on high similarity scores (indicates conflicting database signal from several species with highly similar sequences).

Fitting the *PROTAX* model followed Somervuo et al. [31] except that 5000 training sequences were randomly selected for each target marker due to the large size of the reference database. In each case, 4500 training sequences represented a mix of known species with reference sequences (conspecific sequences retained in the database) and known species without reference sequences (conspecific sequences omitted, simulating species missing from the database), and 500 sequences represented previously unknown lineages distributed evenly across the four taxonomic levels (i.e. mimicked a mix of completely novel species, genera, families and orders). Pairwise sequence similarities of queries and references were calculated with LAST [50] following the approach of Somervuo et al. [31]. The models were weighted towards the Bornean mammals expected in the sampled area by assigning a prior probability of 90% to these 103 species and a 10% probability to all others ([31]; Supplemental Table 2). In cases of missing interspecific variation, this helped to avoid assignments to geographically impossible taxa, especially in case of the very short 93 bp fragment of *16S*. Maximum *a posteriori* (MAP) parameter estimates were obtained following the approach of Somervuo et al. [24], but the models were parameterised for each of the four taxonomic levels independently, with a total of five parameters at each level (four regression coefficients and the probability of mislabelling).

Dereplicated reads for each sample were then classified using a custom bash script on a run-by-run basis. For each sample, reads in FASTQ format were converted to FASTA, and pairwise similarities were calculated against the full reference sequence database for the applicable marker with LAST. Assignments of each read to a taxonomic node based on these sequence similarities were made using a Perl script and the trained model for that level. The taxonomy of each node assignment was added with a second Perl script for a final table including the node assignment, probability, taxonomic level, and taxonomic path for each read. Read count information was included directly in the classification output via the size annotation added to the read headers during dereplication. All Perl scripts to convert input files into the formats expected by *PROTAX*, *R* code for training the model following

Somervuo et al. [31], and Perl scripts for taxonomic assignment were provided by P. Somervuo (personal communication).

Acceptance criteria

In total we had twelve PCR reactions per sample: two *extraction replicates A* and *B* X two *PCR replicates* 1 and 2 per extraction replication X the three markers (Fig. 1). We applied two different acceptance criteria to the data with different stringency regimes. One more naive one that accepted any two positives out of the twelve *PCR replicates* (from now on referred to as lax), and one stringent one that only accepted taxonomic assignments that were positively detected in both *extraction replicates* (*A* & *B*, Fig. 3). Our lax approach refers to one of the approaches of Ficetola et al. [19] where they evaluated different statistical approaches developed to estimate occupancy in the presence of observational errors and has been applied in other studies (e.g. [13]). The reason for conservatively omitting assignments that appeared in only one *extraction replicate* was to rule out sample cross-contamination during DNA extraction. In addition, we only accepted assignments with ten or more reads per marker, if only one marker was sequenced. If a species was assigned in more than one marker (e.g. *12S* and *16S*), we accepted the assignment even if in one sequencing run the number of reads was below ten.

Due to the imperfect PCR amplification of markers (the small *16S* fragment amplified better than the longer *CytB* fragment) and missing reference sequences in the database or shared sequence motifs between species, reads sometimes were assigned to species level for one marker but only to genus level for another marker. Thus, the final identification of species could not be automated, and manual inspection and curation was needed. For each assignment, three parameters were taken into consideration: number of sequencing reads, the mean probability estimate derived from *PROTAX*, and the mean sequence similarity to the reference sequences based on LAST.

Shot-gun sequencing to quantify mammalian DNA content

As the success of the metabarcoding largely depends on the mammal DNA quantity in our leech bulk samples we quantified the mammalian DNA content in a subset of 58 of our leech samples using shotgun sequencing. Extracted DNA was sheared with a Covaris M220 focused-ultra-sonicator to a peak target size of 100-200 bp, and re-checked for size distribution. Double-stranded Illumina sequencing libraries were prepared according to a ligation protocol designed by Fortes and Paijmans [51] with single 8 nt indices. All libraries were pooled equimolarly and sequenced on the MiSeq using the v3 150-cycle kit. We demultiplexed reads using bcl2fastq and cutadapt for trimming the adapters. We used BLAST search to identify reads and applied Metagenome Analyzer MEGAN [30] to explore the taxonomic content of the data based on the NCBI taxonomy. Finally we used KRONA [52] for visualisation of the results.

Findings & Discussion

Database curation

The Midori UNIQUE database (December 2015 version) contains 1,019,391 sequences across the four mitochondrial loci of interest (*12S*: 66,937; *16S*: 146,164; *CytB*: 223,247; *COI*: 583,043), covering all Metazoa. Of these, 258,225 (25.3%) derive from the four tetrapod classes (Amphibia: 55,254; Aves: 51,096; Mammalia: 101,106; Reptilia: 50,769). The distribution of these sequences between classes and loci, and the losses at each curation step are shown in Figure 4. In three of the four classes, there is a clear bias towards *CytB* sequences, with over 50% of sequences derived from this locus. In both Aves and Mammalia, the *16S* and *12S* loci are severely underrepresented at less than 10% each, while for Reptilia, *COI* is the least sequenced locus in the database.

The numbers of sequences and rates of loss due to our curation steps varied among taxonomic classes and the four loci, although losses were observed between steps in almost all instances. The most significant losses followed amplicon trimming and removal of non-unique sequences. Amplicon trimming led to especially high losses in Amphibia and *16S*, indicating that data published on GenBank for this class and marker do not generally overlap with our amplicons. Meanwhile, the high level of redundancy in public databases was highlighted by the significant reduction in the number of sequences during the final step of removing redundant sequences – in all cases over 10% of sequences was discarded, with some losses exceeding 50% (Mammalia: *COI*, *CytB*, *16S*; Amphibia: *16S*).

Data loss due to apparent mislabelling ranged between 1.9% and 7.4% and was thus generally higher than similar estimates for curated microbial databases [29]. SATIVA flags potential mislabels and suggests an alternative label supported by the phylogenetic placement of the sequences, allowing the user to make an appropriate decision on a case by case basis. The pipeline pauses after this step to allow such manual inspection to take place. However, for the current database, the number of sequences flagged was large (4378 in total), and the required taxonomic expertise was lacking, so all flagged sequences from non-target species were discarded to be conservative. The majority of mislabels were identified at species level (3053), but there were also significant numbers at genus (788), family (364) and order (102) level. Two to three sequences from Bornean mammal species were unflagged in each amplicon to retain the sequences in the database. This was important as in each case these were the only reference sequences available for the species. Additionally, *Muntiacus vaginalis* sequences that were automatically synonymised to *M. muntjak* based on the available information in the Catalogue of Life were revised back to their original identifications to reflect current taxonomic knowledge.

Database composition

The final database was skewed even more strongly towards *CytB* than was the raw database. It was the most abundant locus for each class and represented over 60% of sequences for both Mammalia and Reptilia. In all classes, *16S* made up less than 10% of the final database, with Reptilia *COI* also at less than 10%.

Figure 5 shows that most species represented in the curated database for any locus have just one unique haplotype against which HTS reads can be compared; only a few species have many haplotypes. The prevalence of species with 20 or more haplotypes is particularly notable in *CytB* where the four classes have between 25 (Aves) and 265 (Mammalia) species in this category. The coloured circles in Figure 5 also show that the species of the taxonomy are incompletely represented across all loci, and that coverage varies significantly between taxonomic groups. In spite of global initiatives to generate *COI* sequences [53], this marker does not offer the best species-level coverage in any class and is a poor choice for Amphibia and Reptilia (<15% of species included). Even the best performing marker, *CytB*, is not a universally appropriate choice, as Amphibia is better covered by *12S*. These differences in underlying database composition will impact the likelihood of obtaining accurate taxonomic assignment for any one species from any single marker. Further barcoding campaigns are clearly needed to fill gaps in the reference databases for all markers and all classes to increase the power of future e/iDNA studies. As the costs of HTS decrease, we expect that such gap-filling will increasingly shift towards sequencing of whole mitochondrial genomes of specimen obtained from museum collections, trapping campaigns etc. [34], reducing the effect of marker choice on detection likelihood. In the meantime, however, the total number of species covered by the database can be increased by combining multiple loci (here, up to four) and thus the impacts of database gaps on correctly detecting species can be minimized ([54]; Fig. 6).

In the present study, the primary target for iDNA sampling was the mammal fauna of Malaysian Borneo, and the 103 species expected in the sampling area represent an informative case study highlighting the deficiencies in existing databases (Fig. 7). Nine species are completely unrepresented while only slightly over half (55 species) have at least one sequence for all of the loci. Individually, each marker covers over half of the target species, but none achieves more than 85% coverage (*12S*: 75 species; *16S*: 68; *CytB*: 88; *COI*: 66). Equally striking is the lack of within-species diversity, as most of the incorporated species are represented by only a single haplotype per locus. Some of the species have large distribution ranges, so it is likely that in some cases the populations on Borneo differ genetically from the available reference sequences, possibly limiting assignment success. Only a few expected species have been sequenced extensively, and most are of economic importance to humans (e.g. *Bos taurus*, *Bubalus bubalis*, *Macaca* spp, *Paradoxurus hermaphroditus*, *Rattus* spp., *Sus scrofa*), with as many as 100 haplotypes available (*Canis lupus*). Other well-represented species (≥20 haplotypes) present in the sampling area include several Muridae (*Chiropodomys gliroides*, *Leopoldamys sabanus*, *Maxomys surifer*, *Maxomys whiteheadi*) and the leopard cat (*Prionailurus bengalensis*).

Laboratory workflow

Shotgun sequencing of a subset of our samples revealed that the median mammalian DNA content was only 0.9%, ranging from 0% to 98%. These estimates are approximate, but with more than 75% of the samples being below 5%, this shows clearly the scarcity of target DNA

in bulk iDNA samples. The generally low DNA content and the fact that the target DNA is often degraded make enrichment of the target barcoding loci necessary. We used PCR with high cycle numbers to obtain enough DNA for sequencing. However, this second step increases the risk of PCR error: artificial sequence variation, non-target amplification, and/or raising contaminations up to a detectable level.

We addressed these problems by running two *extraction replicates*, two *PCR replicates*, and a multi-marker approach. The need for *PCR replicates* has been acknowledged and addressed extensively in ancient DNA studies [16] and has also been highlighted for metabarcoding studies [19; 20; 55; 56]. Despite this, many e/iDNA studies do not carry out multiple *PCR replicates* to detect and omit potential false sequences. In addition, *extraction replicates* are seldom applied, despite the evidence that cross-sample DNA contamination can occur during DNA extraction [57; 58; 59]. We only accepted sequences that appeared in a minimum of two independent PCRs for the lax and for the stringent criterion, where it has to occur in each *extraction replicate A* and *B* (Fig. 1). The latter acceptance criterion is quite conservative and produces higher false negative rates than e.g. accepting occurrence of at least two positives. However, it also reduces the risk of accepting a false positives compared to it (see Supplemental Fig. 1. for a simulation of false positive and false negatives rates within a PCR), especially with increasing risk of false positive occurrence in a PCR for e.g. example due to higher risk of contamination etc.. Metabarcoding studies are very prone to false negatives, and downstream analyses like occupancy models for species distributions can account for imperfect detection and false negatives. However, methods for discounting false positive detections are not well developed [60]. Thus we think it is more important to avoid false positives, especially if the results will be used to make management decisions regarding rare or endangered species. In contrast, it might be acceptable to use a relaxed acceptance criterion for more common species, as long as the rate false-positives/true-positives is small and does not affect species distribution estimates. Employing both of our tested criteria researchers could flag unreliable assignments and management decisions can still use this information, but now in a forewarned way. An alternative to our acceptance criteria could be use the PCR replicates itself to model the detection probability within a sample using an occupancy framework [20; 60; 61].

We used three different loci to correct for potential PCR-amplification biases. We were, however, unable to quantify this bias in this study due to the high degradation of the target mammalian DNA, which resulted in much higher overall amplification rates for *16S*, the shortest of our PCR amplicons. For *16S*, 85% of the samples amplified, whereas for *CytB* and *12S*, only 57% and 44% amplified, respectively. Also the read losses due to trimming and quality filtering were significantly lower for the *16S* sequencing runs (1.3% and 5.3% in average, Supplemental Table 3) compared to the sequencing runs for the longer fragments of *12S* and *CytB* (65.3% and 44.3% in average, Supplemental Table 3). Despite the greater taxonomic resolution of the longer *12S* and *CytB* fragments, our poorer amplification and sequencing results for these longer fragments emphasize that e/iDNA studies should

generally focus on short PCR fragments to increase the likelihood of positive amplifications of the degraded target DNA. In the case of mammal-focussed e/iDNA studies, developing a shorter (100 bp) *CytB* fragment would likely be very useful.

Another major precaution was the use of twin-tagging for both PCRs (Fig. 2). This ensures that unlabelled PCR products are never produced and allows us to multiplex a large number of samples on a single run of Illumina MiSeq run. Just 24 sample *tags 1* and 20 plate *tags 2* allow the differentiation of up to 480 samples with matching tags on both ends. The same number of individual primers would have needed longer tags to maintain enough distance between them and would have resulted in an even longer adapter-tag overhang compared to primer length. This would have most likely resulted in lower binding efficiencies due to steric hindrances of the primers. Furthermore, this would have resulted in increased primer costs. Thus our approach reduced sequencing and primer purchase costs while at the same time largely eliminating sample mis-assignment via tag jumping, because tag-jump sequences have non-matching forward and reverse *tag 1* sequences [43]. We estimated the rate of tag jumps producing non-matching *tag 1* sequences to be 1 to 5%, and these were removed from the dataset (Table 4). For our sequenced PCR plates, the rate of non-matching *tag 2* tags was 2%. These numbers are smaller than data from Zepeda-Mendoza et al. [56] who reported on sequence losses of 19% to 23% due to unused tag combinations when they tested their DAMe pipeline to different datasets built using standard blunt-end ligation technique. Although their numbers might not be one-to-one comparable to our results as they counted unique sequences, and we report on read numbers, our PCR libraries with matching barcodes seem reduce the risk of tag jumping compared to blunt-end ligation techniques. For the second PCR round, we used the same tag pair *tag 2* for all 24 samples of a PCR plate. In order to reduce cost we tested pooling these 24 samples prior to the second PCR round, but we detected a very high tag jumping rate of over 40% (Table 4), which ultimately would increase cost through reduced sequencing efficiency. Twin-tagging increases costs because of the need to purchase a larger number of primer pairs but at the same time it increases confidence in the results.

Tagging primers in the first PCR reduces the risk of cross-contamination via aerosolised PCR products. However, we would not be able to detect a contamination prior the second PCR from one plate to another, as we used the same 24 tags (*tag 1*) for all plates. Nevertheless such a contamination is very unlikely to result in any accepted false positive as it would be improbable to contaminate both the A and B replicates, given the exchange of all reagents and the time gap between the PCRs. Previous studies have shown that unlabelled volatile PCR products pose a great risk of false detections [62], a risk that is greatly increased if a high number of samples are analysed in the laboratories [13]. Also, in laboratories where other research projects are conducted, this approach allows the detection of cross-experiment contamination. Therefore, we see a clear advantage of our approach over ligation techniques when it comes to producing sequencing libraries, as the Illumina tags are

only added after the first PCR, and thus the risk of cross contamination with unlabelled PCR amplicons is very low.

Assignment results

A robust assignment of species is an important factor in metabarcoding as an incorrect identification might result incorrect management interventions. The reliability of taxonomic assignments is expected to vary with respect to both marker information content and database completeness, and this is reflected in the probability estimates provided by *PROTAX*. In a recent study, less than 10% of the mammal assignments made at species level against a worldwide reference database were considered reliable with the short *16S* amplicon, but this increased to 46% with full-length *16S* sequences [31]. In contrast, in the same study over 80% of insect assignments at species level were considered reliable with a more complete, geographically restricted database of full-length COI barcodes. A similar pattern was observed in our data during manual curation of the assignment results – there was more ambiguity in the results for the short *16S* amplicon than for other markers. However, due to the limited amount of often degraded target DNA in e/iDNA samples, short amplicons amplify much better. In our case, this had the drawback that some species lacked any interspecific variation, and thus sequencing reads shared 99%-100% identity for several species. For example, our only *16S* reference of *Sus barbatus* was 100% identical to *S. scrofa*. But as latter species does not occur in the studied area we could assign all reads manually to *S. barbatus*. In several cases we were able to confirm *S. barbatus* by additional *CytB* results, highlighting the usefulness of multiple markers.

Another advantage of multiple markers is the opportunity to fill gaps in the reference database. For example, we lacked *16S* reference sequences for *Hystrix brachyura*, and reads were assigned by *PROTAX* only to the unknown species *Hystrix* sp.. In one sample, however, almost 5000 *CytB* reads could be confidently assigned to *Hystrix brachyura*, and thus we used the *Hystrix* sp. 16S sequences in the same sample to build a consensus *16S* reference sequence for *Hystrix brachyura* for future analyses. In another example we had *CytB* reads assigned to *Mydaus javanicus*, the Sunda stink-badger in one sample but *12S* reads assigned to *Mydaus* sp. in another one. As we lacked a *12S Mydaus* reference and as there is only one *Mydaus* species on Borneo we could assume that this second sample is most likely also *Mydaus javanicus*.

We also inferred that PCR and sequencing errors resulted in reads being assigned to sister taxa. We observed that a high number of reads of a true sequence were assigned to a species and a lower number of noise sequences were assigned to a sister taxon. Such a pattern was observed for ungulates, especially deer that showed little variance in *16S*. It is hard to identify and control for such pattern automatically, and it highlights the importance of visual inspection of the results.

For the more lax criterion (two positive *PCR replicates*) we accepted 196 species assignments out of 109 leech samples. Under the stringent criterion (i.e. having positive detections in both *extraction replicates A* and *B*) we accepted about 14% assignments less;

in total 170 vertebrate detections within 97 bulk samples (Table 5). For 65% of the species frequencies did not change and almost half of the not accepted assignments were from the most frequent species *Rusa unicolor* and *Sus barbatus*. However, with the more stringent criterion we did not accept two species (1x *Macaca fascicularis* & 2x *Mydaus javanensis*). In five cases the stringent criterion would not accept assignments that could be made only to unknown species (1x *Hystrix* sp., 3x *Macaca* sp., 1x *Tragulus* sp.) (Table 5). For the all these gernera we have two occurring species in the area. As the true occurrence of species within our leeches was unknown we cannot evaluate how many of the additional 27 detections in the lax criterion are false positives and how many might be false negatives for the stricter criterion. However, by accepting only positive *AB* assignment results, we increase the confidence of species detection, even if the total number of reads for that species was low. When it comes to rare or threated species this outweighs the risk of reporting false positives to our opinion. 48% of the assignments with the stringent criterion were present in all four *A1*, *A2*, *B1* and *B2*. 35% were present in at least three replicates (e.g. A1, A2, B1).

The mean number of reads per sample used for the taxomomic assignment varied from 162,487 *16S* reads for SeqRun01 to 7,638 *CytB* reads for SeqRun05 (Supplemental Table 4). In almost all cases, however, the number of reads of an accepted assignment was high (median= 52,386; mean= 300,996; SD= 326,883). PCR stochasticity, primer biases, multiple species in individual samples, and pooling of samples exert too many uncertainties that could bias the sequencing results [63; 64]. Thus we do not believe that raw read numbers are the most reliable indicators of tetrapod DNA quantity in iDNA samples. Replication of detection is inherently more reliable. In contrast to our expectation that higher cycle number might be necessary to amplify even the lowest amounts of target DNA, our data do not support this hypothesis. Although we observed an increase in positive PCRs for A2/B2 (the 40-cycle PCR replicates), the total number of accepted assignments in *A1/B1* and *A2/B2* samples did not differ. This indicates first that high PCR cycle numbers mainly increased the risk of false positives and second that our multiple precautions successfully minimized the acceptance of false detections.

Conclusion

Metabarcoding of e/iDNA samples will certainly become a very valuable tool in assessing biodiversity, as it allows to detect species non-invasively without the need to capture and handle the animals [65] and because sampling effort can often be greatly reduced. However, the technical and analytical challenges linked to sample types (low quantity and quality DNA) and poor reference databases have so far been insufficiently recognized. In contrast to ancient DNA studies where standardized laboratory procedures and specialized bioinformatics pipelines have been established and are followed in most cases, there is limited methodological consensus in e/iDNA studies, which reduces rigour. In this study, we present a robust metabarcoding workflow for e/iDNA studies. We hope that the provided scripts and protocols facilitate further technical and analytical developments. The use of e/iDNA metabarcoding to study the rarest and most endangered species such as the Saola is

exciting, but geneticists bear the heavy responsibility of providing correct answers to conservationists.

Acknowledgements

All authors thank the German Federal Ministry of Education and Research (BMBF FKZ: 01LN1301A) and the Leibniz-IZW for funding this study. We also thank the Sabah Forestry Department, especially Johnny Kissing, Peter Lagan and Datuk Sam Mannan for supporting the fieldwork and the Sabah Biodiversity Council for providing research, collection and export permits for this work. We are grateful to John Mathai, Seth Timothy Wong for conducting the field work and collecting the leeches. We are also grateful to Jörns Fickel, head of the Department Evolutionary Genetics of the Leibniz-IZW for continuous support and collaboration. Furthermore we would like to thank Sebastian Wieser for lab-support, Dorina Lenz and Anke Schmidt for their help and fruitful discussions. D.W. Yu and C.C.Y. Xu were supported by the National Natural Science Foundation of China (41661144002, 31670536, 31400470, 31500305), the Key Research Program of Frontier Sciences, CAS (QYZDY-SSW-SMC024), the Bureau of International Cooperation project (GJHZ1754), the Strategic Priority Research Program of the Chinese Academy of Sciences (XDA20050202, XDB31000000), the Ministry of Science and Technology of China (2012FY110800), and the State Key Laboratory of Genetic Resources and Evolution at the Kunming Institute of Zoology.

References

[1] Gariepy TD, Lindsay R, Odgen N, Greory TR. Identifying the last supper: utility of the DNA barcode library for bloodmeal identification in ticks. Mol Ecol Res. 2012; 12: 646-52.

[2] Lee P-S, Gan HM, Clements GR, Wilson J-J. Field calibration of blowfly-derived DNA against traditional methods for assessing mammal diversity in tropical forests. Genome 2016;59: 1008-22.

[3] Calvignac-Spencer S, Merkel K, Kutzner N, et al.. Carrion fly-derived DNA as a tool for comprehensive and cost-effective assessment of mammalian biodiversity. Mol Ecol 2013; 22: 915-24.

[4] Rodgers TW, Xu CCY, Giacalcone J, et al.. Carrion fly-derived DNA metabarcoding is an effective tool for mammal surveys: Evidence from a known tropical mammal community. Mol Ecol Res. 2017; 17(6):1-13

[5] Hoffmann C, Merkel K, Sachse A, et al.. Blow flies as urban wildlife sensors. Mol Ecol Res. 2018; 18(3):502-10

[6] Schönberger AC, Wagner S, Tuten HC, et al.. Host preferences in host-seeking and 632 blood-fed mosquitoes in Switzerland. Med Vet Entomol. 2015; 30(1): 39-52.

[7] Townzen JS, Brower AVZ, Judd DD. Identification of mosquito bloodmeals using mitochondrial cytochrome oxidase subunit I and cytochrome b gene sequences. Med Vet Entomol. 2008; 22. 386-93.

[8] Kocher A, Thoisy B, Catzeflies F, et al.. iDNA screening: Disease vectors as vertebrate samplers. Mol Ecol. 2017; 26(22): 6478-86.

[9] Taylor L, Cummings RF, Velten R, et al.. Host (Avian) Biting Preference of Southern California Culex Mosquitoes (Diptera: Culicidae). J Med Entomol. 2012; 49(3): 687-96.

[10] Schnell IB, Thomsen PF, Wilkinson N, et al.. Screening mammal biodiversity using DNA from leeches. Curr Biol. 2012, 22(8): R262—3.

[11] Tessler M, Weiskopf SR, Berniker L, et al.. Bloodlines: mammals, leeches, and conservation in southern Asia. Syst Biodivers. 2018; 1-9.

[12] Weiskopf SR, McCarthy KP, Tessler M, et al.. Using terrestrial haematophagous leeches to enhance tropical biodiversity monitoring programmes in Bangladesh. J Appl Ecol. 2018: 1-11.

[13] Schnell IB, Bohmann K, Schultze SE, et al.. Debugging diversity - a pan-continental exploration of the potential of terrestrial blood-feeding leeches as a vertebrate monitoring tool. Mol Ecol Res. 2018.

[14] Calvignac-Spencer S, Leendertz FH, Gilbert MT, Schubert G. An invertebrate stomach's view on vertebrate ecology: certain invertebrates could be used as "vertebrate samplers" and deliver DNA-based information on many aspects of vertebrate ecology. BioEssays. 2013; 35(11): 1004-13.

[15] Schnell IB, Sollmann R, Calvignac-Spencer S, et al.. iDNA from terrestrial haematophagous leeches as a wildlife surveying and monitoring tool – prospects, pitfalls and avenues to be developed. Front Zool. 2015; 12:24.

[16] Pääbo S, Poinar H, Serre D, et al.. Genetic analyses from ancient DNA. Annu Rev Genet. 2004; 38: 645-79.

[17] Hofreiter M, Paijmans JL, Goodchild H, et al. The future of ancient DNA: Technical advances and conceptual shifts. BioEssays. 2015; 37(3): 284-93.

[18] Cristescu ME, Hebert , PDN. Uses and Misuses of Environmental DNA in Biodiversity Science and Conservation. Cristescu, Melania E und Hebert, Paul D N. 1, 2018, Annu Rev Ecol Evol Syst 2018; 49.

[19] Ficetola GF, Pansu J, Bonin A, et al.. Replication levels, false presences and the estimation of the presence/absence from eDNA metabarcoding data. Mol Ecol Res. 2014; 15(3): 543-56.

[20] Ficetola GF, Taberlet P., Coissac E. How to limit false positives in environmental DNA and metabarcoding? Mol Ecol Res. 2016; 16(3): 604-7.

[21] Dalton R. Still looking for that woodpecker. Nature 2010; 463: 718-9.

[22] Bazinet AL, Cummings MP. A comparative evaluation of sequence classification programs. BMC bioinformatics. 2012; 13(1): 92.

[23] Richardson RT, Bengtsson-Palme J, Johnson RM. Evaluating and optimizing the performance of software commonly used for the taxonomic classification of DNA metabarcoding sequence data. Mol Ecol Res. 2017; 17(4): 760-9.

[24] Somervuo P, Koskela S, Pennanen J, Henrik Nilsson R, Ovaskainen O. Unbiased probabilistic taxonomic classification for DNA barcoding. Bioinformatics. 2016; 32(19): 2920-7.

[25] Quast C, Pruesse E, Gerken J, et al.. SILVA Databases. In: Nelson KE (eds) Encyclopedia of Metagenomics. Springer, Boston 2015; 626-35.

[26] Nilsson RH, Ryberg M. Taxonomic Reliability of DNA Sequences in Public Sequence Databases: A Fungal Persepctive. PLoS ONE. 1, 2006; 1.

[27] Forster P. To Err is Human. Ann Hum Gen 2003; 67: 2-4.

[28] Harris JD. Can you bank on GenBank? Trends Ecol Evol. 2003; 18: 317-9. doi:10.1016/S0169-5347(03)00150-2.

[29] Kozlov AM, Zhang J, Yilmaz P, Glöckner FO, Stamatakis A. Phylogeny-aware identification and correction of taxonomically mislabeled sequences. Nucleic Acids Res. 2016; 44(11): 5022-33.

[30] Huson DH, Auch AF, Qi J, Schuster SC. MEGAN analysis of metagenomic data. Genome Res. 2007; 17(3): 377-86.

[31] Somervuo P, Yu DW, Xu CC, Ji Y, et al.. Quantifying uncertainty of taxonomic placement in DNA barcoding and metabarcoding. Methods Ecol Evol. 2017; 8(4): 398-407.

[32] Machida RJ, Leray M, Ho SL, Knowlton N. Metazoan mitochondrial gene sequence reference datasets for taxonomic assignment of environmental samples. Sci Data. 2017; 4: 170027.

[33] Chamberlain SA, Szöcs E. taxize: taxonomic search and retrieval in R. Version 2. F1000Res. 2013; 2: 191.

[34] Salleh FM, Ramos-Madrigal J, Peñaloza F, et al.. An expanded mammal mitogenome dataset from Southeast Asia. GigaScience. 2017; 6(8): 1-8

[35] Kans, Jonathan. Entrez Direct: E-utilities on the UNIX Command Line. In: Entrez Programming Utilities Help [Internet]. Bethesda (MD): National Center for Biotechnology Information (US). 2010.

[36] Altschul SF, Gish W, Miller W, Myers EW, Lipman DJ. Basic local alignment search tool. Journal of molecular biology. 1990; 215(3):, 403-10.

[37] Camacho C, Coulouris G, Avagyan V, et al.. BLAST+: architecture and applications. BMC bioinformatics. 2009; 10(1): 421.

[38] Katoh K, Standley DM. (2013). MAFFT multiple sequence alignment software version 7: improvements in performance and usability. Mol Biol Evol. 2013; 30(4): 772-80.

[39] Katoh K, Misawa K, Kuma KI, Miyata T. MAFFT: a novel method for rapid multiple sequence alignment based on fast Fourier transform. Nucleic Acids Res. 2002; 30(14): 3059-66.

[40] Chesters D. (2013) collapsetypes.pl [computer software available at http://sourceforge.net/projects/collapsetypes/]

[41] Kocher TD, Thomas WK, Meyer A, et al.. Dynamics of mitochondrial DNA evolution in animals: amplification and sequencing with conserved primers. Proc. Natl. Acad. Sci. U.S.A.. 1989; 86(16): 6196-6200.

[42] Taylor PG. Reproducibility of ancient DNA sequences from extinct Pleistocene fauna. Mol Biol Evol. 2996; 13(1): 283-5.

[43] Schnell IB, Bohmann K, Gilbert MTP. Tag jumps illuminated–reducing sequence-to-sample misidentifications in metabarcoding studies. Mol Ecol Res. 2015; 15(6): 1289-1303.

[44] Faircloth BC, Glenn TC. Not all sequence tags are created equal: designing and validating sequence identification tags robust to indels. PloS One. 2012; 7(8): e42543

[45] Murray DC, Coghlan ML, Bunce M. From benchtop to desktop: important considerations when designing amplicon sequencing workflows. PLoS One. 2015; 10(4): e0124671.

[46] Schubert M, Lindgreen S, Orlando L. AdapterRemoval v2: rapid adapter trimming, identification, and read merging. BMC Research Notes 2016; 9.

[47] Edgar RC. Search and clustering orders of magnitude faster than BLAST. Bioinformatics. 2010; 26(19): 2460-2461.

[48] Edgar RC, Flyvbjerg H. Error filtering, pair assembly and error correction for next-generation sequencing reads. Bioinformatics. 2015; 31(21): 3476-82.

[49] Martin M. Cutadapt removes adapter sequences from high-throughput sequencing reads. EMBnet. Jjournal. 2011; 17(1): 10-12.

[50] Kiełbasa SM, Wan R, Sato K, Horton P, Frith MC. Adaptive seeds tame genomic sequence comparison. Genome Res. 2011; 21(3): 487-493.

[51] Fortes GG, Paijmans JLA. Analysis of Whole Mitogenomes from Ancient Samples. In: Kroneis T (eds). Whole Genome Amplification: Methods and Protocols. Springer New York 2015; 179-195.

[52] Ondov BD, Bergman NH, Phillippy AM. Interactive metagenomic visualization in a Web browser. BMC Bioinformatics 2011; 12: 385.

[53] Ratnasingham S, Hebert PDN. BOLD: The Barcode of Life Data System (www.barcodinglife.org). Mol Ecol Notes. 2007; 3: 355-64.

[54] Evans NT, Li Y, Renshaw MA, et al. Fish community assessment with eDNA metabarcoding: effects of sampling design and bioinformatic filtering. Can J Fish Aquat Sci. 2017; 74(9):, 1362-74.

[55] Bonin A, Taberlet P, Zinger L, Coissac E. Environmental DNA: For Biodiversity Research and Monitoring. 1st ed. Oxford University Press; 2018.

[56] Zepeda-Mendoza ML, Bohmann K, Baez A, Gilbert M., DAMe: a toolkit for the initial processing of datasets with PCR replicates of double-tagged amplicons for DNA metabarcoding analyses. BMC Res Notes 2016; 9.

[57] Racimo F, Renaud G, Slatkin M. Joint estimation of contamination, error and demography for nuclear DNA from ancient humans. PLoS Genet. 2016; 12(4): e1005972.

[58] Orlando L, Gilbert MTP, Willerslev E. Reconstructing ancient genomes and epigenomes. Nat Rev Genet 2015; 16(7): 395

[59] Laurin-Lemay S, Brinkmann H, Philippe H. Origin of land plants revisited in the light of sequence contamination and missing data. Current Biology. 2012; 22(15): R593-4.

[60] Lahoz-Monfort JJ, Guillera-Arroita G, Tingley R. Statistical approaches to account for false-positive errors in environmental DNA samples. Mol Ecol Res. 2015; 16: 673-85.

[61] Dorazio RM, Erickson RA. ednaoccupancy: An r package for multiscale occupancy modelling of environmental DNA data. Mol Ecol Res. 2018; 18: 368-80.

[62] Kwok S, Higuchi R. Avoiding false positives with PCR. Nature. 1989; 339: 237-8.

[63] Bush A, Sollmann R, Wilting A, et al.. Connecting Earth observation to high-throughput biodiversity data. Nat Ecol Evol 2017; 1(7): 0176

[64] Piñol J, Mir G, Gomez-Polo P, Agustí N. Universal and blocking primer mismatches limit the use of high-throughput DNA sequencing for the quantitative metabarcoding of arthropods., Mol Ecol Res.2014; 15: 819-30.

[65] Nichols RV, Vollmers C, Newsom L, et al.. Minimizing polymerase biases in metabarcoding. Mol Ecol Res. 2018; 18: 927-39.

Table 1: Sequence motifs that compose the 25 different target primers for the first and the second PCR. First PCR primers consist of target specific primer followed by an overhang out of sample specific *tag 1* and *read 1* and *read 2* sequencing primer, respectively. The second PCR primers consist of the *read 1* or the *read 2* sequencing primer followed by an plate specific *tag 2* and the P5 and P7 adapters, respectively (see also Fig. 2).

Name	Sequence	Reference
tag A	TGCAT	Faircloth & and Glenn 2012
tag B	TCAGC	Faircloth & and Glenn 2012
tag C	AAGCG	Faircloth & and Glenn 2012
tag D	ACAAG	Faircloth & and Glenn 2012
tag E	AGTGG	Faircloth & and Glenn 2012
tag F	TTGAC	Faircloth & and Glenn 2012
tag G	CCTAT	Faircloth & and Glenn 2012
tag H	GGATG	Faircloth & and Glenn 2012
tag I	CTAGG	Faircloth & and Glenn 2012
tag K	CACCT	Faircloth & and Glenn 2012
tag L	GTCAA	Faircloth & and Glenn 2012
tag M	GAAGT	Faircloth & and Glenn 2012
tag N	CGGTT	Faircloth & and Glenn 2012
tag O	ACCGA	Faircloth & and Glenn 2012
tag P	ACGTC	Faircloth & and Glenn 2012
tag Q	AGACT	Faircloth & and Glenn 2012
tag R	AGGAA	Faircloth & and Glenn 2012
tag S	ATTCC	Faircloth & and Glenn 2012
tag T	CAATC	Faircloth & and Glenn 2012
tag V	CATGA	Faircloth & and Glenn 2012
tag W	CCACA	Faircloth & and Glenn 2012
tag X	GCTTA	Faircloth & and Glenn 2012
tag Y	GGTAC	Faircloth & and Glenn 2012
tag Z	AACAC	Faircloth & and Glenn 2012
Tag Control	ATCTG	Faircloth & and Glenn 2012
CytB-fw	AAAAAGCTTCCATCCAACATCTCAGCATGATGAAA	Kocher et al. 1989
CytB-rv	AAACTGCAGCCCCTCAGAATGATATTTGTCCTCA	Kocher et al. 1989
16S-fw	CGGTTGGGGTGACCTCGGA	Taylor 1996
16S-rv	GCTGTTATCCCTAGGGTAACT	Taylor 1996
12S-fw	AAAAAGCTTCAAACTGGGATTAGATACCCCACTAT	Kocher et al. 1989
12S-rv	TGACTGCAGAGGGTGACGGGCGGTGTGT	Kocher et al. 1989
Read 1 sequence primer	ACACTCTTTCCCTACACGACGCTCTTCCGATCT	Illumina Document # 1000000002694 v03
Read 2 sequence primer	GTGACTGGAGTTCAGACGTGTGCTCTTCCGATCT	Illumina Document # 1000000002694 v03
P5 adapter	AATGATACGGCGACCACCGAGATCTACAC	Illumina Document # 1000000002694 v03
P7 adapter	CAAGCAGAAGACGGCATACGAGAT	Illumina Document # 1000000002694 v03

Table 2: Main steps undertaken by each module of the database curation script.

MODULE	STEPS
Module 1	Extract subset of raw Midori database for query taxon and loci.
	Remove sequences with non-binomial species names, reduce subspecies to species labels
	Add local sequences (optional)
	Check for relevant new sequences for list of query species on NCBI (GenBank and RefSeq) (optional)
	Select amplicon region and remove primers
	Remove sequences with ambiguous bases
	Align
	End of module: Optional check of alignments
Module 2	Compare sequence species labels with taxonomy
	Non-matching labels queried against Catalogue of Life to check for known synonyms
	Remaining mismatches kept if genus already exists in taxonomy, otherwise flagged for removal
	End of module: Optional check of flagged species labels
Module 3	Discard flagged sequences
	Update taxonomy key file for sequences found to be incorrectly labelled in Module 2
	Run SATIVA
	End of module: Optional check of putatively mislabelled sequences
Module 4	Discard flagged sequences
	Finalise consensus taxonomy and relabel sequences with correct species label and accession number
	Select one representative sequence per haplotype per species

Table 3: GNU core utilities and other lightweight tools used for manipulation of text and sequence files

TOOL	FUNCTION	SOURCE
awk, cut, grep, join, sed, sort, tr	Processing text files	GNU core utilities
seqbuddy	Processing FASTA/Q files	https://github.com/biologyguy/BuddySuite
seqkit	Processing FASTA/Q files	https://github.com/shenwei356/seqkit
seqtk	Processing FASTA/Q files	https://github.com/lh3/seqtk
tabtk	Processing tab-delimited text files	https://github.com/lh3/tabtk

Table 4: Number of reads per sequencing run and the numbers of reads with matching, non-matching or unidentifiable tags for seven of the eight sequencing runs*.

	total reads	matching tag 2 reads	non-matching tag 2 reads	%[1]	matching tag 1 reads	non-matching tag 1 reads	%[2]	erroneous tag 1 reads	%[2]
**SeqRun01**	18,438,517	18,102,702	282,419	1.5	17,514,515	451,028	2.5	137,159	0.8
**SeqRun02**	25,385,558	24,596,380	626,245	2.5	23,426,084	612,045	2.5	558,251	2.3
**SeqRun03**	14,875,796	14,393,884	343,528	2.3	13,766,187	426,181	3.0	201,516	1.4
**SeqRun04**	2,027,794	1,935,149	56,077	2.8	1,806,655	88,307	4.6	40,187	2.1
**SeqRun05**	18,221,504	17,500,366	421,588	2.3	16,793,851	482,365	2.8	161,458	0.9
**SeqRun06**	20,718,202	19,874,913	429,048	2.1	19,317,305	371,048	1.9	81,422	0.4
**SeqRun07**	24,604,610	23,746,938	663,730	2.7	22,446,187	497,366	2.1	803,385	3.4
**Total**	124,271,981	120,150,332	2,822,635	2.3	115,070,784	2,928,340	2,5	1,983,378	1,7
**IndexRun**	10,276,093	10,116,808	NA	NA	5,841,190	4,186,688	41.4	88,930	0.9

[1] refers to total reads

[2] refers to matching tag 2

*Sequencig run SeqRun08 run contained libraries of another project, thus we were unable to provide a number of raw reads.

Table 5: Number of accepted species assignments with two different acceptance criteria the more stringent criterion accepting only assignments occurring in both *extraction replicates* (A & B), and the more lax criterion accepting assignment two or more positives in any of the twelve *PCR replicates*.

	stringent	lax	change
Aonyx cinereus	1	1	0
Arctictis binturong	1	1	0
Bos Javanicus	9	11	+2
Echinosorex gymnura	5	6	+1
Felis catus	2	2	0
Helarctos malayanus	5	6	+1
Hemigalus derbyanus	3	3	0
Hystrix brachyura	8	8	0
Hystrix crassipinis	1	1	0
Hystrix sp.	1	2	+1
Kalophrynus pleurostigma	1	1	0
Macaca fascicularis		1	+1
Macaca nemestrina	1	2	+1
Macaca sp.		3	+3
Manis javanicus	2	2	0
Muntiacus atherodes	6	6	0
Muntiacus muntjak	2	2	0
Muntiacus sp.	10	10	0
Mydaus javanensis		1	+1
Mydaus sp.		1	+1
Pongo pygmaeus	5	5	0
Rusa unicolor	61	69	+8
Sus barbatus	17	22	+5
Tragulus javanicus	3	3	0
Tragulus napu	10	11	+1
Tragulus sp.		1	+1
Trichys fasciculata	4	4	0
Viverra tangalunga	12	12	0
**total accepted assignments**	170	197	+27

Figure 1: laboratory scheme; during DNA extraction the sample is split into two extraction replicates A & B. Our Protocol consists of two rounds of PCR that were the sample tags, the necessary sequencing primer and sequencing adapters are added to the the amplicons. For each extraction replicate we ran a low cycle PCR and a high cycle PCR for each marker that we have twelve independent PCR replicates per sample. All PCR products were sequenced and the obtained reads were taxonomically identified with PROTAX.

Figure 2: Scheme to build double 'twin-tagged' PCR libraries. The first round of PCR uses target-specific primers (*12S*, *16S*, or *CytB*, dark grey) that have both been extended with the same (i.e. 'twin') sample-identifying *tag* sequences *tag 1* (yellow) and then with the different *read 1* (dark blue) and *read 2* (light blue) sequence primers. The second round of PCR uses the priming sites of the *read 1* and *read 2* sequencing primers to add twin plate-identifying *tag* sequences *tag 2* (orange) and the P5 (dark red) and P7 (light red) Illumina adapters.

Figure 3: For the stringent acceptance criterion we only accepted taxonomic assignments that were positively detected in both *extraction replicates* A and B (green colour). The numbers (1 & 2) refer to the two PCR replicates for each extraction replicate.

Figure 4: Data availability and percentage loss at each major step in the database curation procedure for each target amplicon and class of Tetrapoda. The number of sequences decreases between steps except "Extra sequences added" where additional target sequences are included for Mammalia and there is no change for the other three classes.

Figure 5: Haplotype number by species (frequency distribution) and the total number of species with at least one haplotype, shown relative to the total number of species in the taxonomy for that category (bubbles), shown for each marker and class of Tetrapoda. The proportion of species covered by the database varies between categories but in all cases a majority of recovered species are represented by a single unique haplotype.

Figure 6: The percentage of the full taxonomy covered by the final database at each taxonomic level for each class of Tetrapoda. Includes the percentage of taxa represented by each marker and all markers combined. In all cases taking all four markers together increases the proportion of species, genera and families covered by the database, but it remains incomplete when compared with the full taxonomy.

Figure 7: The number of unique haplotypes per marker for each of the 103 mammal species expected in the study area. Bubble size is proportional to the number of haplotypes and varies between 0 and 100. Only 55 species have at least one sequence per marker and nine species are completely unrepresented in the current database.

Supplemental Figure 1: The rates of accepted false negatives (upper graph) and false positives (lower graph) for both our used acceptance criteria for varying PCR detection probabilities. The red line always denotes the stringent acceptance criterion that a positive is only accepted if it is present in at least one A and one B replicate. The lax criterion (blue)

accepted at any two positives out of the twelve replicates. The stringent criterion poses a higher risk of accepting a false negative but it reduces clearly the risk of false positives, especially with increasing detection probability due to higher risk of contamination.

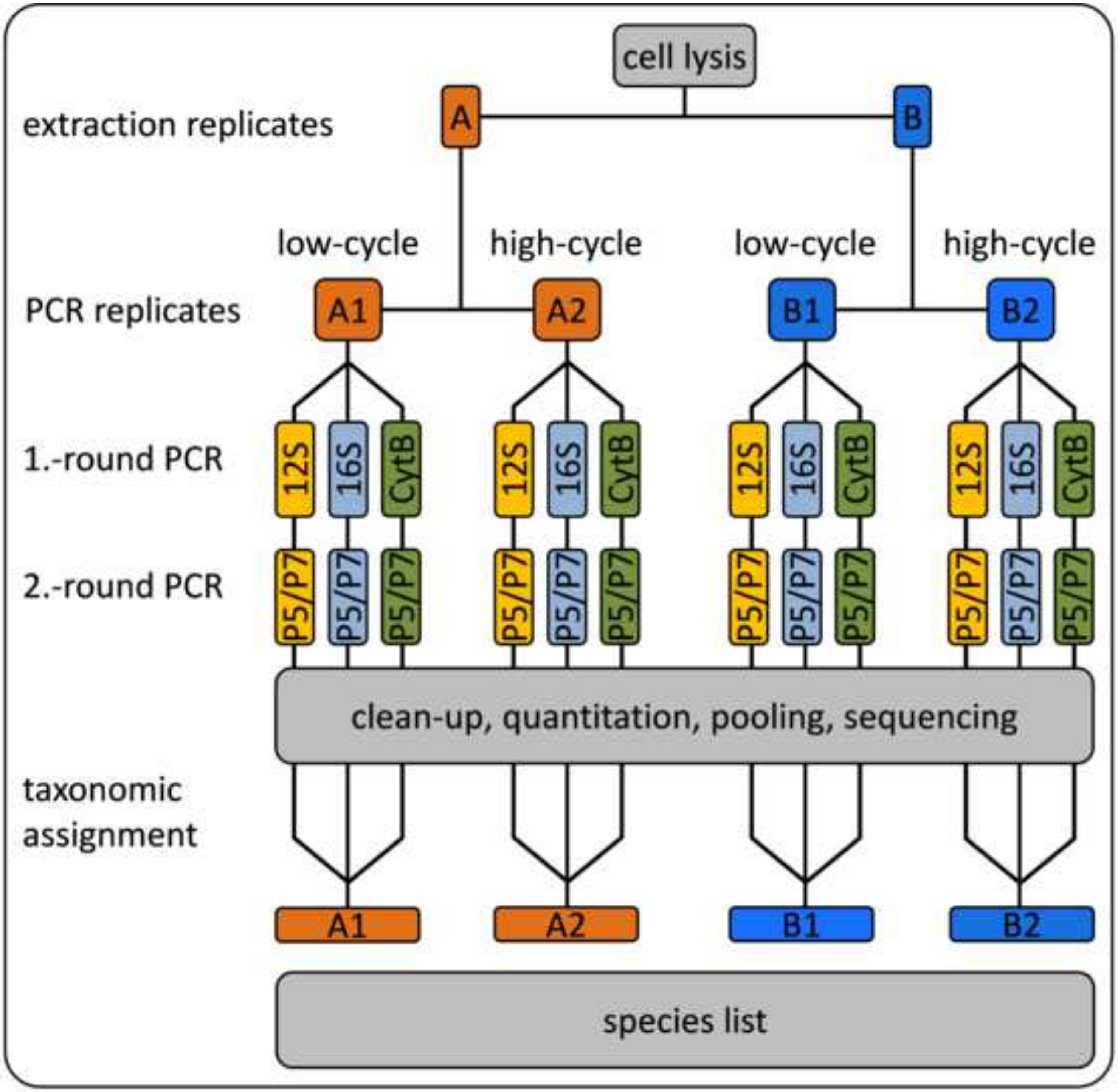
cell lysis
extraction replicates
A
B
low-cycle
high-cycle
low-cycle
high-cycle
PCR replicates
A1
A2
B1
B2
1.-round PCR
12S
16S
CytB
2.-round PCR
P5/P7
clean-up, quantitation, pooling, sequencing
taxonomic assignment
A1
A2
B1
B2
species list

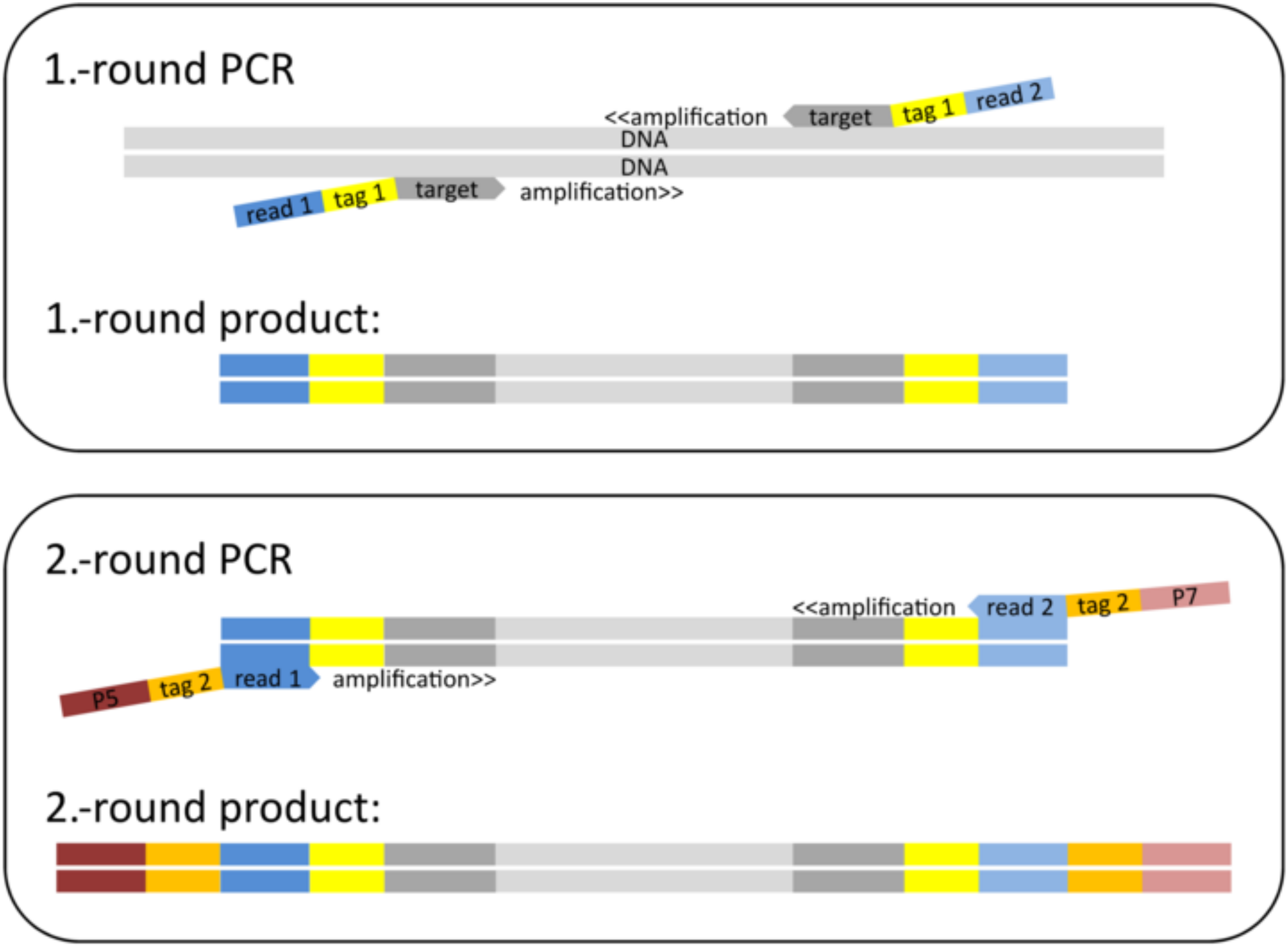
1.-round PCR
<<amplification
target
tag 1
read 2
DNA
DNA
read 1
tag 1
target
amplification>>
1.-round product:
2.-round PCR
<<amplification
read 2
tag 2
P7
P5
tag 2
read 1
amplification>>
2.-round product:

 Click here to access/download;Figure;Fig3.png

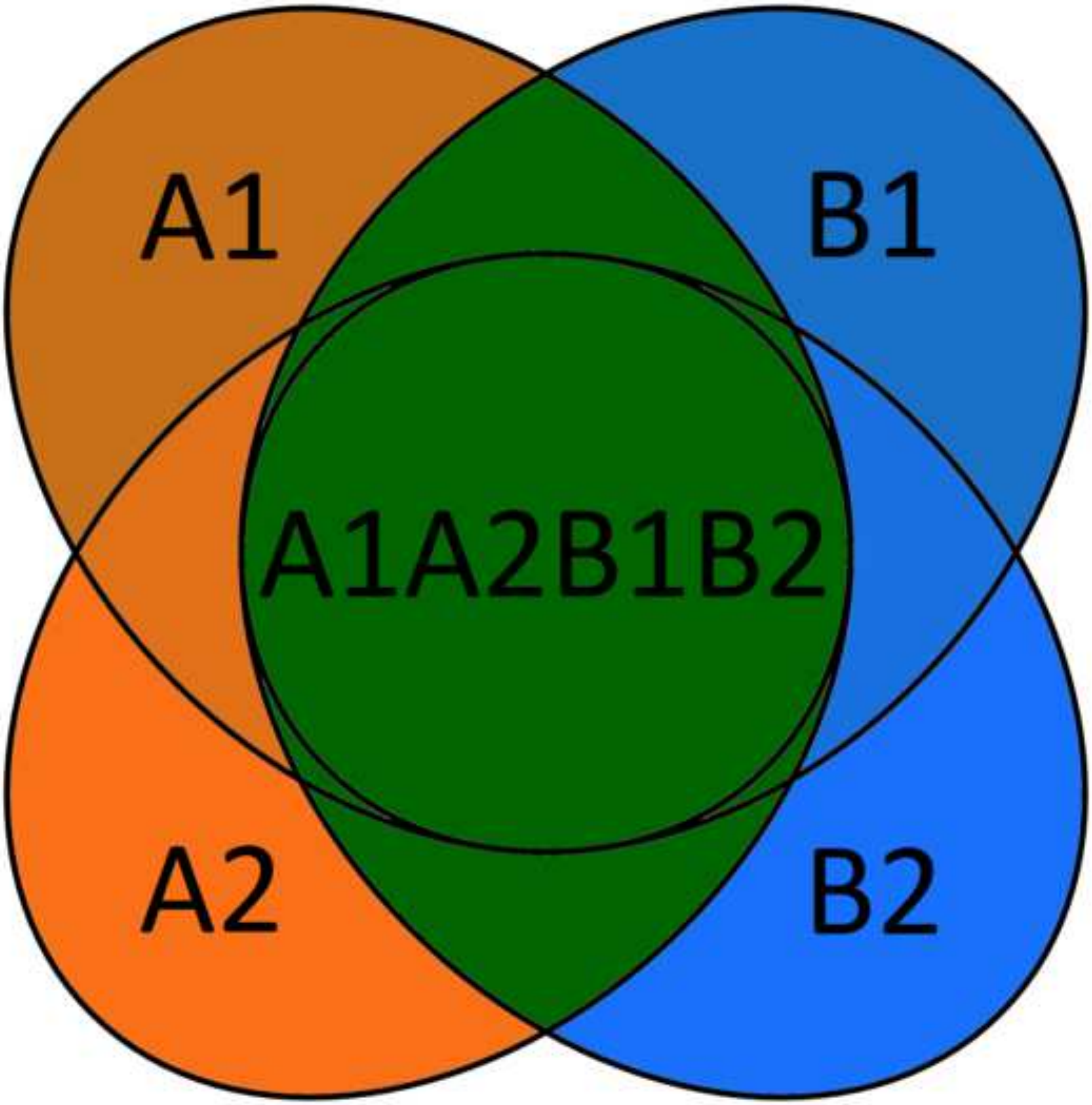

Figure

Click here to access/download;Figure;Fig4.pdf

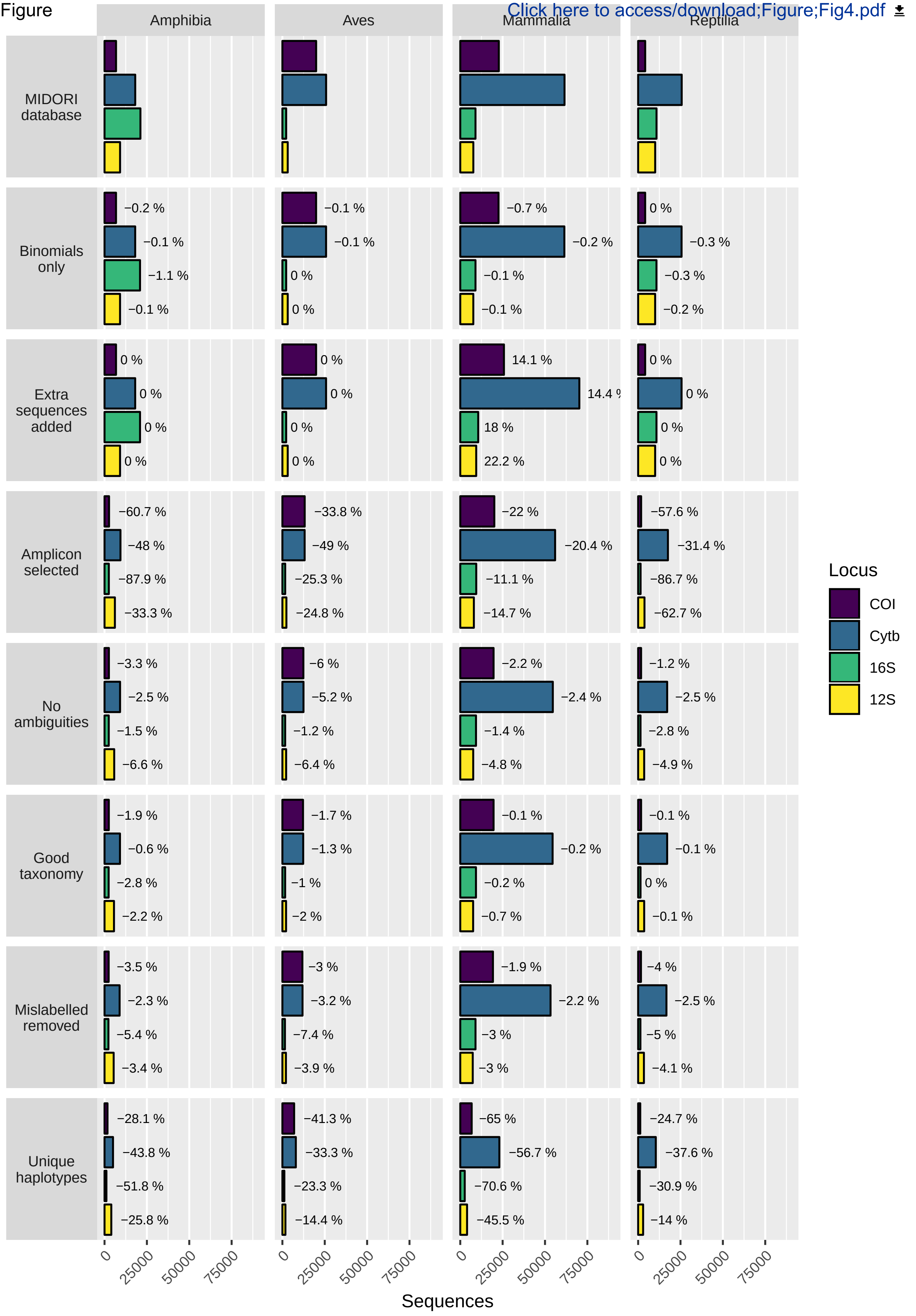

Figure

Click here to access/download;Figure;Fig5.pdf

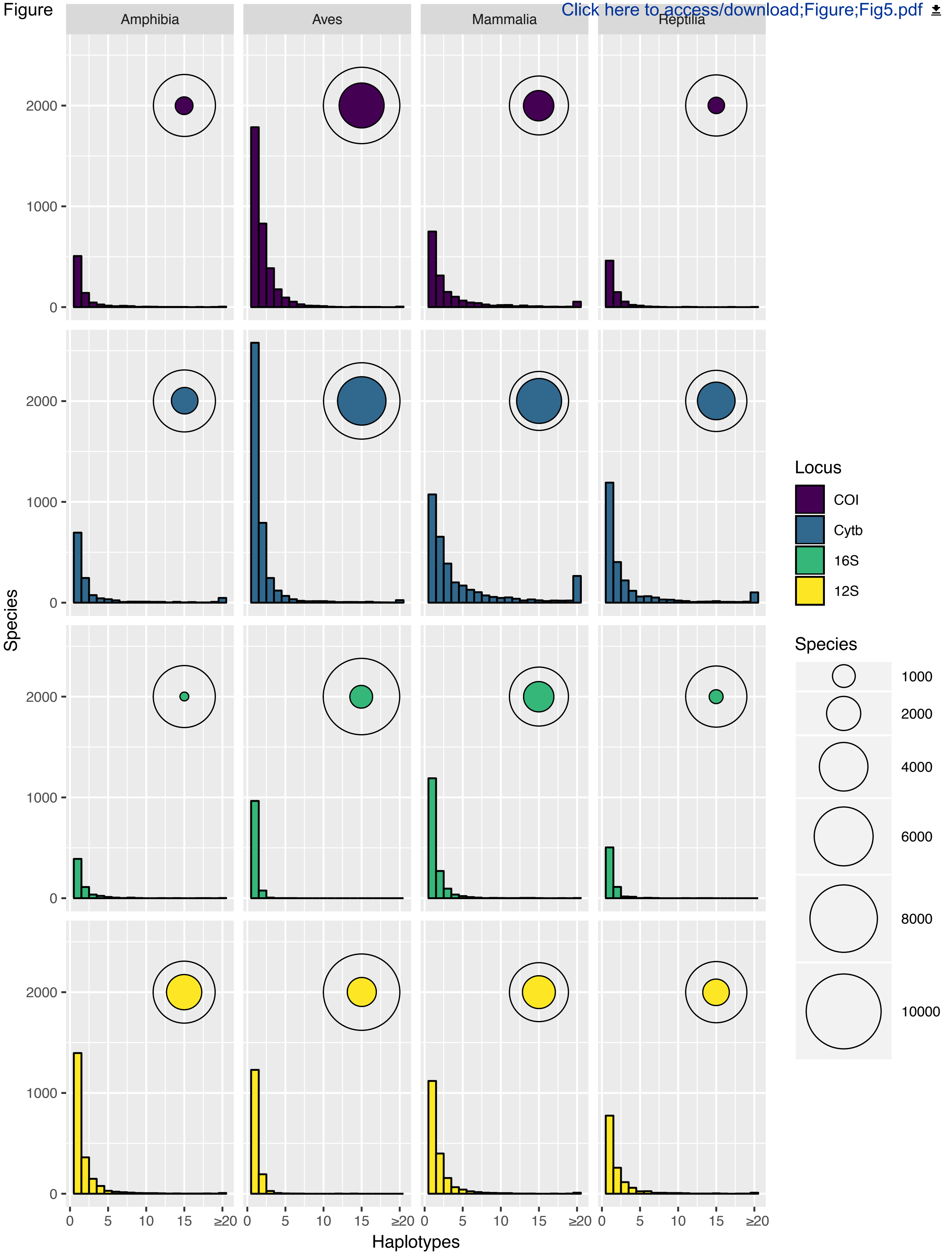

Figure

Click here to access/download;Figure;Fig6.pdf

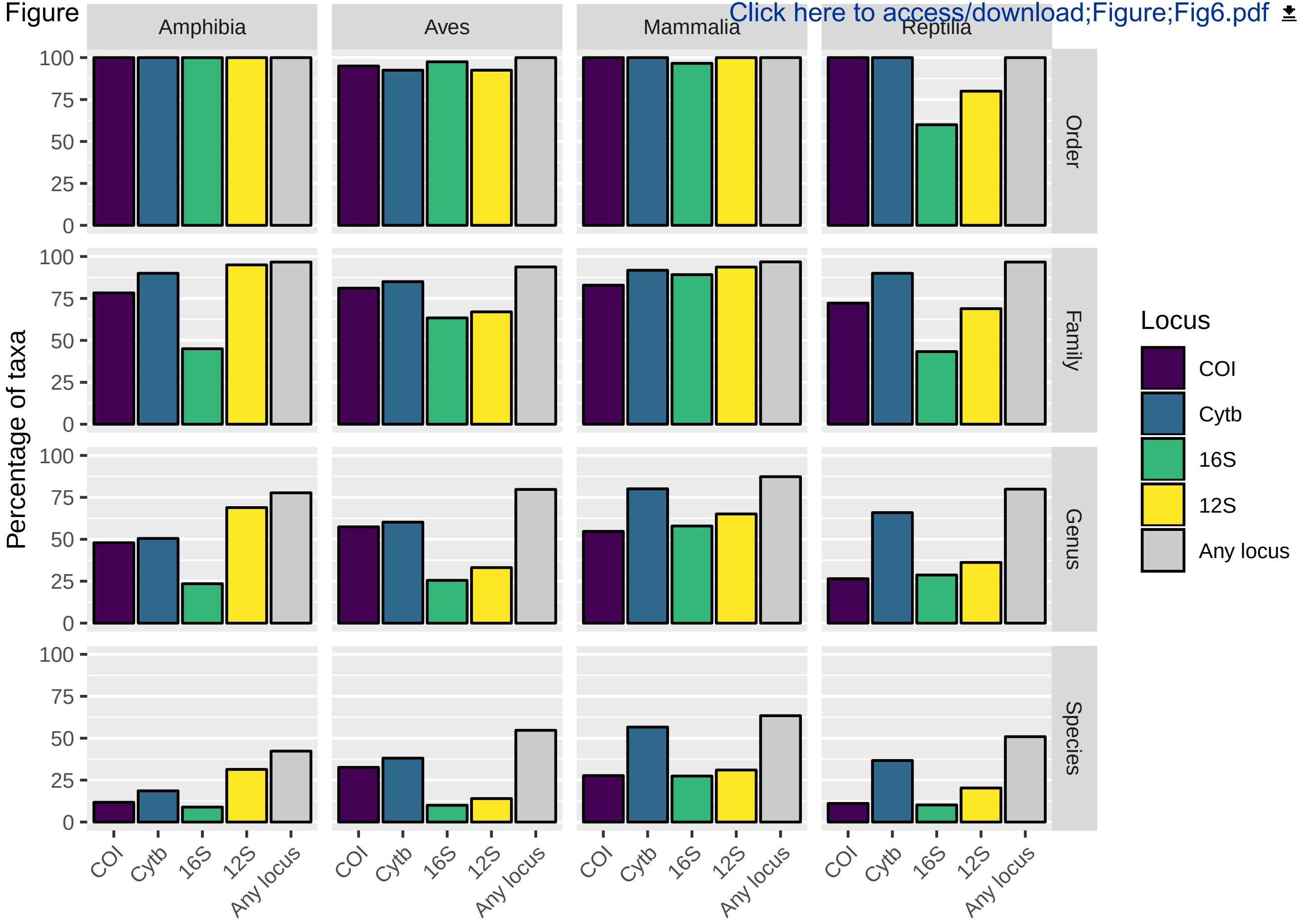

Figure

Click here to access/download;Figure;Fig7.pdf

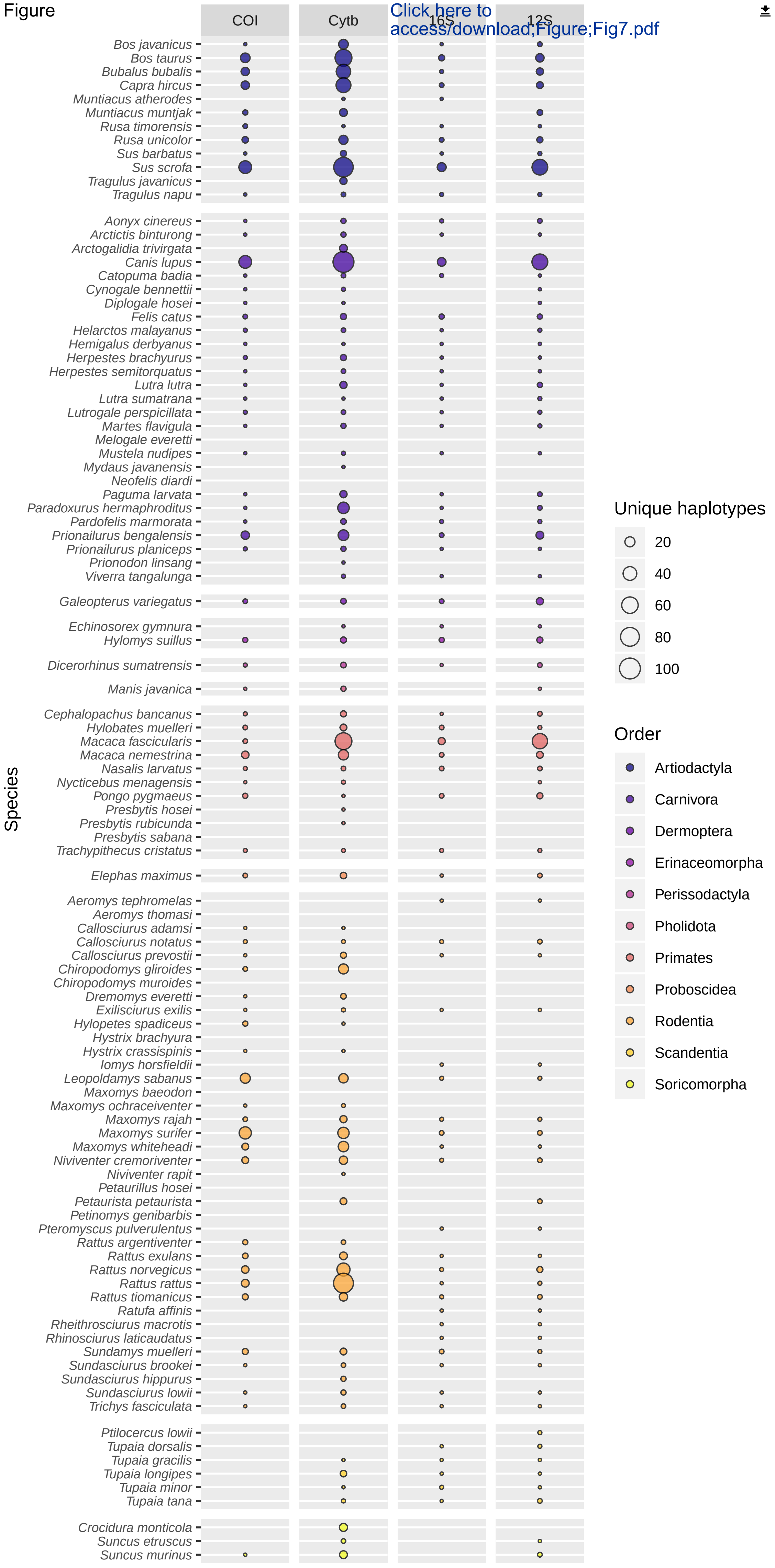

Click here to access/download
Supplementary Material
Suppl_Fig1.jpg

Click here to access/download
Supplementary Material
Supplemental table 1.pdf

Click here to access/download
Supplementary Material
Supplemental table 2.pdf

Click here to access/download
Supplementary Material
Supplemental table 3.pdf

Click here to access/download
Supplementary Material
Supplemental table 4.pdf

Personal Cover
Click here to access/download;Personal Cover;Letter_to_the_editor.docx

Leibniz Institute for Zoo and Wildlife Research
IN THE FROSCHUNGSVERBUND BERLIN E.V.

Leibniz-Gemeinschaft

Biodiversity & Biogeography of Southeast Asia

DR JAN AXTNER
TEL. +49 30 51 68-339
AXTNER@IZW-BERLIN.DE

TOTAL E-QUALITY

To
Hongling Zhou
Editorial Board of
GigaScience

Submission of the revised version of manuscript GIGA-D-18-00219

Dear Hongling Zhou,

Thank you very much for the chance to revise our manuscript. Hereby we would like to re-submit our revised version of the manuscript GIGA-D-18-00219.

The reviewers helped us a lot to streamline and focus the manuscript and we hope this brought it to a level that it could be considered for publication. According to the reviewers comments we have changed the title to “An efficient and improved laboratory workflow and tetrapod database for larger scale eDNA studies.” We do not object to change the article type and present the workflow and the database as Technical Note.

With regard to the reviewer comments we did some substantial changes in the manuscript. First, we used now two different acceptance criteria, re-analysed and discussed our data for both criteria. Furthermore we added a small simulation study to the supplements showing the differences for both acceptance criteria with respect to the acceptance of false positives and false negatives (Supplemental Figure 1). To address the concerns of reviewer 2 about sequencing depths and sequencing quality we added Supplemental Tables 3 & 4 on read losses during read processing and read numbers per sample. We provided detailed point-by-point answers to the reviewer’s comments and hope that we could address them all sufficiently. However, their comments helped us a lot to improve the manuscript in many ways.

On behalf of our co-authors,

Jan Axtner & Andreas Wilting
