# Supplementary material for: An efficient and robust laboratory workflow and tetrapod database for larger scale environmental DNA studies
Source: Gigascience. 2019 Apr 13;8(4):giz029. doi: 10.1093/gigascience/giz029 (PMC6461710; doi:10.1093/gigascience/giz029)
Supplement: GIGA-D-18-00219_Revision_2.pdf [file giz029_giga-d-18-00219_revision_2.pdf]

GigaScience

An efficient and robust laboratory workflow and tetrapod database for larger scale eDNA studies

--Manuscript Draft--

**Manuscript Number:**	GIGA-D-18-00219R2
**Full Title:**	An efficient and robust laboratory workflow and tetrapod database for larger scale eDNA studies
**Article Type:**	Technical Note
**Funding Information:**	German Federal Ministry of Education and Research (BMBF) (01LN1301A) — Dr. Andreas Wilting
**Abstract:**	Background<br>The use of environmental DNA, 'eDNA,' for species detection via metabarcoding is growing rapidly and now, even terrestrial mammals can be monitored via 'invertebrate-derived DNA' or 'iDNA' from hematophagous invertebrates. We present a co-designed lab workflow and bioinformatic pipeline to mitigate the two most important risks of e/iDNA: sample contamination and taxonomic mis-assignment. These risks arise from the need for amplification to detect the trace amounts of DNA and the necessity of using short target regions due to DNA degradation.<br>Findings<br>Here we present a high-throughput laboratory workflow that minimises these risks via a three-step strategy: (1) each sample is sequenced for two PCR replicates from each of two extraction replicates; (2) we use a 'twin-tagging,' two-step PCR protocol; (3) and a multi-marker approach targeting three mitochondrial loci: 12S, 16S and CytB. As a test, 1532 leeches were analysed from Sabah, Malaysian Borneo. Twin-tagging allowed us to detect and exclude chimeric sequences. The smallest DNA fragment (16S) amplified best for all samples but often at lower taxonomic resolution. We only accepted assignments that were found in both extraction replicates, totalling 174 assignments for 96 samples.<br>To avoid false taxonomic assignments, we also present an approach to create curated reference databases that can be used with the powerful taxonomic-assignment method PROTAX. For some taxonomic groups and some markers, curation resulted in over 50% of sequences being deleted from public reference databases, due mainly to: (1) limited overlap between our target amplicon and available reference sequences; (2) apparent mislabelling of reference sequences; (3) redundancy. A provided bioinformatics pipeline processes amplicons and conducts the PROTAX taxonomic assignment.<br>Conclusions<br>Our metabarcoding workflow should help research groups to increase the robustness of their results and therefore facilitate wider usage of e/iDNA, which is turning into a valuable source of ecological and conservation information on tetrapods.
**Corresponding Author:**	Jan Axtner<br>Leibniz Institute for Zoo and Wildlife Research<br>Berlin, Germany GERMANY
**Corresponding Author Secondary Information:**	
**Corresponding Author's Institution:**	Leibniz Institute for Zoo and Wildlife Research
**Corresponding Author's Secondary Institution:**	
**First Author:**	Jan Axtner
**First Author Secondary Information:**	
**Order of Authors:**	Jan Axtner
	Alex Crampton-Platt
	Lisa Hörig

Powered by Editorial Manager® and ProduXion Manager® from Aries Systems Corporation

	Azlan Mohamed
	Charles C.Y. Xu
	Douglas W. Yu
	Andreas Wilting
**Order of Authors Secondary Information:**	
**Response to Reviewers:**	Dear Hongling Zhou,<br><br>First we would like to thank both reviewers for their positive feedback and the editor for the potential interest to publish our paper in GigaScience. Below we provide a detailed response to the remaining comments and suggestions by the reviewers. These certainly helped to improve the manuscript further and we thank the reviewers for their valuable comments.<br>On behalf of our co-authors,<br><br>Jan Axtner & Andreas Wilting<br><br><br>Reviewer reports:<br><br>Reviewer #1: Thank you for taking the time to address all comments in detail. The corrections I think have improved the clarity of the piece, and I feel convinced where you explained where I misunderstood. One possible reference to consider (given a comment about the availability of models to account for errors at multiple levels):<br><br>Guillera-Arroita. 2017. Dealing with false-positive and false-negative errors about species occurrence at multiple levels. Methods in Ecology and Evolution. https://doi.org/10.1111/2041-210X.12743<br><br># Thank you for the positive feedback and the interesting article. So far we were not aware of it, but as it fit's so perfectly to our topic, thus we now refer to it in line 561<br><br><br><br>Reviewer #2: I am overall satisfied with the responses provided by the authors. In general, it is quite unlikely nowadays that there will be a consensus for the "right/best" way forward. It is always subject to practicality/funding. If i were to conduct my own amplicon seq project, will I follow this protocol to the dots - no. However, the bioinformatics scripts and data generated will be useful for better experimental design in the future. Furthermore, even if a method is robust, lab competency / human error (mislabeling, mixing the wrong index etc) is still going to be an issue.<br>------<br>Reviewer 1 raised the concern of similar tag1 being used repeatedly for multiple samples. I wonder if instead of using "Twin" tag, having a different tag1 combination (non-Twin tag?!) will be helpful (obviously for discussion). In other words, the forward and reverse primer combination in the 1st PCR round can be Tag1a for forward Tag1b for reverse. This is somewhat similar to dual indexing in Illumina but you're doing it at the initial stage and should will expand the 24 sample limitation for the tag1 based on my current understanding the twin-tag but happy to be proven wrong. With the increasing problem of index hoping particularly with the patterned flowcell for the Novaseq and Iseq (relevant to amplicon seq) , this should be useful and worth looking into.<br><br>See https://www.biorxiv.org/content/early/2017/10/19/205799<br><br># We agree that are other factors like lab skills or human errors that are an important issue and in fact our whole laboratory procedure is designed to minimize human-related errors. The whole workflow is designed to allow a high-throughput of samples in a maximum standardized way, i.e. sample aliquots are arranged already in eight-well stripes for the use of eight-channel pipets in order to minimize the risk of pipetting the wrong sample into the wrong well between the different replicates. That is also one of the reasons why we do not start mixing the tag1 combinations and re-use the 24 tags

Powered by Editorial Manager® and ProduXion Manager® from Aries Systems Corporation

for each PCR plate. Our forward and reverse primers are already pre-mixed in an eight-well stripe and we use the same pipetting scheme with an eight-channel pipet for every 96-well PCR plate. If we would start using different tag combinations for each PCR plate we would have a much higher risk of pipetting errors mixing the wrong indices (handling 48 tubes is much more error prone than handling just three 8-well stripes).
In addition to this rather practical lab-work related reason we highlighted (Line: 602 to 605) that it is still very unlikely that the repeated use of tags for multiple samples causes accepted false positives in the end, as the final acceptance is not based on single occurrence but on repeated occurrence in independent replicates. We fully agree that the use of non-matching tags (e.g. A/B) would increase the number of samples that could be analysed in one sequencing run. But at the same time it would make it much harder to identify contaminations or tag jumps as we discuss in line 575 to 599. Contaminations of a PCR with another differently labelled PCR product would increase the number of chimeras in your PCR which would remain undetected if you would also use non-matching tag combination. The same holds true for tag-jumps, which are an issue in Illumina sequencing (see Schnell et al. 2015) and where we could demonstrate that our PCR libraries reduce the read-losses compared to adapter-ligation techniques (lines 585-594).Particular for the last reasons we favoured to use only twin-tag combinations.

We also thank the reviewer for the interesting paper, which also used quadruple-indexed libraries. We do however not see the application of RAD sequencing to identify invertebrate-derived DNA of unknown origin. Generally RAD sequencing requires high molecular weight genomic DNA. Our samples have a mixed pools of genomic and mitochondrial DNA from different organisms and our target DNA is often highly degraded, of poor quality and of low quantities. In addition we have the presence of high amounts of leech DNA. Therefore we currently do not see an application of this sequencing method.

"Also the read losses due to trimming and quality filtering were significantly lower for the 16S sequencing runs (1.3% and 5.3% in average, Supplemental Table 3) compared to the sequencing runs for the longer fragments of 12S and CytB (65.3% and 44.3% in average, Supplemental Table 3)."

The Usearch read overlapping pipeline is sensitive to number of mismatches in alignment. The Read2 in MiSeq 600 cycles run is particularly notoriously for being low quality towards the end of the run. Try trimming both R1 and R2 to 250 bp (length trimming) and redo the overlap and read loss calculation.

Thank you, for this valuable advice. We tested it for one of our 12S runs and compared results. As you suggested we trimmed the reads to 250 base pairs adjusted the -fastq_minovlen parameter for usearch from 50bp to just 25bp as we would expect to have a smaller overlap of the trimmed reads. In fact we obtained more read after merging (13,129,505 vs. 13,388,933). However, most of those reads were lost again after filtering so that our original settings produced in fact the most reads I the end (4,694,624 vs. 4,227,346). Thus we think it is reasonable to stick to the current settings in the pipeline.
Results original pipeline:
raw reads:13,766,169
merging: 13,129,505
clipping:6,498,738
filtering:4,694,624
Trimmed reads (trimm 250bp, overlap 25bp):
raw reads:13,766,169
merging:13,388,933
clipping:6,684,766
filtering:4,227,346

"All three markers were amplified simultaneously for each batch of samples in a single

Powered by Editorial Manager® and ProduXion Manager® from Aries Systems Corporation

	PCR plate".<br><br>In different individual well?<br># Sorry for the misunderstanding, we did not do multiplex-PCR and amplified in individual wells. We added this to the sentence in lines 324-325:<br>"… All three markers were amplified simultaneously in individual wells for each batch of samples in a single PCR plate. …"<br><br><br>Because of different amplicon lengths and therefore different binding affinities to the flow cell<br>Also due to clustering efficiency . smaller fragment = easier to amplify<br># We agree, also due to DNA degradation we had higher amplification success for the shortest fragment (see lines 562 – 566). As we say in lines 337-340 "…Because of different amplicon lengths and therefore different binding affinities to the flow cell, 12S and CytB products were combined in a single library, whereas positive 16S products were always combined in a separate library. …" and these libraries were sequenced independently. To make this clearer we added a second sentence (line 340): "… 12S/CytB libraries were sequenced independently from 16S libraries…."
**Additional Information:**	
**Question**	**Response**
Are you submitting this manuscript to a special series or article collection?	No
**Experimental design and statistics**<br><br><br>Full details of the experimental design and statistical methods used should be given in the Methods section, as detailed in our Minimum Standards Reporting Checklist. Information essential to interpreting the data presented should be made available in the figure legends.<br><br><br>Have you included all the information requested in your manuscript?	Yes
**Resources**<br><br><br>A description of all resources used, including antibodies, cell lines, animals and software tools, with enough information to allow them to be uniquely identified, should be included in the Methods section. Authors are strongly encouraged to cite Research Resource Identifiers (RRIDs) for antibodies, model organisms and tools, where possible.<br><br><br>Have you included the information requested as detailed in our Minimum	Yes

Standards Reporting Checklist?	
**Availability of data and materials**<br><br>All datasets and code on which the conclusions of the paper rely must be either included in your submission or deposited in publicly available repositories (where available and ethically appropriate), referencing such data using a unique identifier in the references and in the “Availability of Data and Materials” section of your manuscript.<br><br>Have you have met the above requirement as detailed in our Minimum Standards Reporting Checklist?	Yes

Manuscript

Click here to access/download;Manuscript;Leeches_Methods_Database_dr

Click here to view linked References

An efficient and robust laboratory workflow and tetrapod database for larger scale eDNA studies

Jan Axtner[1+], Alex Crampton-Platt[1], Lisa A. Hörig[1], Azlan Mohamed[1], Charles C.Y. Xu[2,3,4], Douglas W. Yu[2,5] and Andreas Wilting[1]

Affiliations:

[1] Leibniz Institute for Zoo and Wildlife Research (*Leibniz-IZW*), Alfred-Kowalke-Str. 17, 10315 Berlin, Germany

[2] State Key Laboratory of Genetic Resources and Evolution, Kunming Institute of Zoology, Chinese Academy of Sciences, 32 Jiaochang East Road, Kunming, Yunnan 650223, China

[3] Groningen Institute for Evolutionary Life Sciences, University of Groningen, P.O. Box 11103, 9700 CC Groningen, The Netherlands

[4] Redpath Museum and Department of Biology, McGill University, Montreal, QC, Canada

[5] School of Biological Sciences, University of East Anglia, Norwich Research Park, Norwich, Norfolk NR47TJ, UK

[+] corresponding author

email addresses:

Jan Axtner: axtner@izw-berlin.de, ORCID: 0000-0003-1269-5586
Alex Crampton-Platt: alex@naturemetrics.co.uk
Lisa A. Hörig: lisa.hoerig@arcor.de
Azlan Mohamed: mohamed@izw-berlin.de, ORCID: 0000-0003-3788-4383
Charles C.Y. Xu: charles.cong.xu@gmail.com, ORCID: 0000-0001-6779-8879
Douglas W. Yu: dougwyu@mac.com
Andreas Wilting: wilting@izw-berlin.de, ORCID: 0000-0001-5073-9186

Keywords:

metabarcoding, iDNA, eDNA, leeches

Abstract

Background

The use of environmental DNA, 'eDNA,' for species detection via metabarcoding is growing rapidly. We present a co-designed lab workflow and bioinformatic pipeline to mitigate the two most important risks of eDNA: sample contamination and taxonomic mis-assignment. These risks arise from the need for PCR amplification to detect the trace amounts of DNA combined with the necessity of using short target regions due to DNA degradation.

Findings

Our high-throughput workflow minimises these risks via a four-step strategy: (1) technical replication with two *PCR replicates* and two *extraction replicates*; (2) using multi-markers (*12S*, *16S*, *CytB*); (3) a 'twin-tagging,' two-step PCR protocol;(4) use of the probabilistic taxonomic assignment method *PROTAX*, which can account for incomplete reference databases.

As annotation errors in the reference sequences can result in taxonomic mis-assignment, we supply a protocol for curating sequence datasets. For some taxonomic groups and some markers, curation resulted in over 50% of sequences being deleted from public reference databases, due to (1) limited overlap between our target amplicon and reference sequences; (2) mislabelling of reference sequences; (3) redundancy.

Finally, we provide a bioinformatic pipeline to process amplicons and conduct *PROTAX* assignment and tested it on an 'invertebrate derived DNA' (iDNA) dataset from 1532 leeches from Sabah, Malaysia. Twin-tagging allowed us to detect and exclude sequences with non-matching tags. The smallest DNA fragment (*16S*) amplified most frequently for all samples, but was less powerful for discriminating at species rank. Using a stringent and lax acceptance criteria we found 162 (stringent) and 190 (lax) vertebrate detections of 95 (stringent) and 109 (lax) leech samples.

Conclusions

Our metabarcoding workflow should help research groups increase the robustness of their results and therefore facilitate wider usage of e/iDNA, which is turning into a valuable source of ecological and conservation information on tetrapods.

Introduction

Monitoring, or even detecting, elusive or cryptic species in the wild can be challenging. In recent years there has been a rise in the availability of cost-effective DNA-based methods made possible by advances in high-throughput DNA sequencing (HTS). One such method is eDNA metabarcoding, which seeks to identify the species present in a habitat from traces of 'environmental DNA' (eDNA) in substrates such as water, soil, or faeces. A variant of eDNA metabarcoding, known as 'invertebrate-derived DNA' (iDNA) metabarcoding, targets the genetic material of prey or host species extracted from copro-, sarco- or haematophagous invertebrates. Examples include tick [1] s, blow or carrion flies [2; 3; 4; 5], mosquitoes [6; 7; 8; 9] and leeches [10; 11; 12; 13]. Many of these parasites are ubiquitous, highly abundant, and easy to collect, making them an ideal source of biodiversity data, especially for terrestrial vertebrates that are otherwise difficult to detect [10; 14; 15]. In particular, the possibility for bulk collection and sequencing in order to screen large areas and minimise costs is attractive. However, most of the recent studies on iDNA studies focus on single-specimen DNA extracts and Sanger sequencing and thus are not making use of the advances of HTS and a metabarcoding framework for carrying out larger scale biodiversity surveys.

That said, e/iDNA metabarcoding also poses several challenges, due to the low quality and low amounts of target DNA available, relative to non-target DNA (including the high-quality DNA of the live-collected, invertebrate vector). In bulk iDNA samples comprised of many invertebrate specimens, this problem is further exacerbated by the variable time since each individual has fed, if at all, leading to differences in the relative amounts and degradation of target DNA per specimen. This makes e/iDNA studies similar to ancient DNA samples, which also pose the problem of low quality and low amounts of target DNA [16; 17]. The great disparity in the ratio of target to non-target DNA and the low overall amount of the former requires an enrichment step, which is achieved via the amplification of a short target sequence (amplicon) by polymerase chain reaction (PCR) to obtain enough target material for sequencing. However, this enrichment step can result in false positive species detections, either through sample cross-contamination or through volatile short PCR amplicons in the laboratory, and in false-negative results, through primer bias and low concentrations of template DNA. Although laboratory standards to prevent and control for such false results are well established in the field of ancient DNA, there are still no best-practice guidelines for e/iDNA studies, and thus few studies sufficiently account for such problems [18].

The problem is exacerbated by the use of 'universal' primers used for the PCR, which maximise the taxonomic diversity of the amplified sequences. This makes the method a powerful biodiversity assessment tool, even where little is known *a priori* about which species might be found. However, using such primers, in combination with low quality and quantity of target DNA, which often requires a high number of PCR cycles to generate enough amplicon products for sequencing, makes metabarcoding studies particularly vulnerable to false results [13; 19; 20]. The high number of PCR cycles, combined with the

high sequencing depth of HTS, also increase the likelihood that contaminants are amplified and detected, possibly to the same or greater extent as some true-positive trace DNA. As e/iDNA have been proposed as tools to detect very rare and priority conservation species such as the Saola, *Pseudoryx nghetinhensis* [10], false detection might result in misdirected conservation activities worth several hundreds of thousands of US dollars like for the ivory-billed woodpecker where most likely false evidence of the bird's existence have been overemphasized to shore up political and financial support for saving it [21]. Therefore, similar to ancient DNA studies, great care must be taken to minimise the possibility for cross-contamination in the laboratory and to maximise the correct detection of species through proper experimental and analytical design. Replication in particular is an important tool for reducing the incidence of false negatives and detection of false positives but the trade-off is increased cost, workload, and analytical complexity [19].

An important source of false positive species detections is the incorrect assignment of taxonomies to the millions of short HTS reads generated by metabarcoding. Although there has been a proliferation of tools focused on this step, most can be categorised into just three groups depending on whether the algorithm utilises sequence similarity searches, sequence composition models, or phylogenetic methods [22; 23; 24]. The one commonality among all methods is the need for a reliable reference database of correctly identified sequences, yet there are few curated databases currently appropriate for use in e/iDNA metabarcoding. Two exceptions are SILVA [25] for the nuclear markers SSU and LSU rRNA used in microbial ecology, and BOLD (Barcode of Life Database; citation) for the COI 'DNA barcode' region. For other loci, a non-curated database downloaded from the INSDC (International Nucleotide Sequence Database Collaboration, e.g. GenBank) is generally used. However, the INSDC places the burden for metadata accuracy, including taxonomy, on the sequence submitters, with no restriction on sequence quality or veracity. For instance, specimen identification is often carried out by non-specialists, which increases error rates, and common laboratory contaminant species (e.g. human DNA sequences) are sometimes submitted in lieu of the sample itself. The rate of sequence mislabelling in fungi has been assessed for GenBank where it was up to 20% [26] and it is an issue that is often neglected [27; 28]. For several curated microbial databases (Greengenes, LTP, RDP, SILVA), mislabelling rates have been estimated at between 0.2% and 2.5% [29]. Given the lack of professional curation it is likely that the true proportion of mislabelled samples in GenBank is somewhere between these numbers. Moreover, correctly identifying such errors is labour-intensive, so most metabarcoding studies simply base their taxonomic assignments on sequence-similarity searches of the whole INSDC database (e.g. with BLAST) [3; 10; 12] and thus can only detect errors if assignments are ecologically unlikely. Furthermore, reference sequences for the species that are likely to be sampled in e/iDNA studies are often underrepresented in or absent from these databases, which increases the possibility of incorrect assignment. For instance, fewer than 50% of species occurring in a tropical megadiverse rainforest are represented in Genbank (see findings below). When species-level matches are ambiguous, it might still be possible to assign a sequence to a higher

taxonomic rank by using an appropriate algorithm such as Metagenome Analyzer's (MEGAN) Lowest Common Ancestor [30] or *PROTAX* [31].

We present here a complete laboratory workflow and complementary bioinformatics pipeline, starting from DNA extraction to taxonomic assignment of HTS reads using a curated reference database. The laboratory workflow allows for efficient screening of hundreds of e/iDNA samples. The workflow includes (1) two *extraction replicates* are separated during DNA extraction, and each is sequenced in two *PCR replicates* (Fig. 1); (2) robustness of taxonomic assignment is improved by using up to three mitochondrial markers; (3) a 'twin-tagged', two-step PCR protocol prevents cross-sample contamination as no unlabelled PCR products are produced (Fig. 2) while also allowing for hundreds of PCR products to be pooled before costly Illumina library preparation; (4) our bioinformatics pipeline includes a standardized, automated, and replicable protocol to create a curated database, which allows updating as new reference sequences become available, and to be expanded to other amplicons. We provide scripts for processing raw sequence data to quality-controlled dereplicated reads and for taxonomic assignment of these reads using *PROTAX* [31], a probabilistic method that has been shown to be robust even when reference databases are incomplete [23; 4] (all scripts are available from URL https://github.com/alexcrampton-platt/screenforbio-mbc).

Methods

Establishment of the tetrapod reference database

Reference database

A custom bash script was written to generate a tetrapod reference database for up to four mitochondrial markers – a short 93 bp fragment of *16S* rRNA (*16S*), a 389 bp fragment of *12S* rRNA (*12S*), a 302 bp fragment of cytochrome b (*CytB*), and a 250 bp mitochondrial cytochrome c oxidase subunit I amplicon (*COI*) that has previously been used in iDNA studies [2]. An important time-saving step was the use of the FASTA-formatted Midori mitochondrial database [32], which is a lightly curated subset of Genbank. Our script updated the FASTA files with a subset of target species, removed errors and redundancy, trimmed the sequences to include only the amplicon regions, and output FASTA files with species names and GenBank accessions in the headers.

The script accepts four data inputs, two of which are optional. The required inputs are: (i) the Midori sequences (December 2015 'UNIQUE', downloaded from http://www.reference-midori.info/download.php#) for the relevant genes and (ii) an initial reference taxonomy of tetrapods. This taxonomy is needed to find or generate a full taxonomic classification for each sequence because the taxonomies in Midori are from Genbank and thus include incorrect, synonymized, or incomplete taxonomies. Here we used the Integrated Taxonomic Information System (ITIS) classification for Tetrapoda, obtained with the R package *taxize* version 0.9.0 ([33], functions *downstream* and *classification*). The optional inputs are: (iii) supplementary FASTA files of reference sequences that should be added to the database,

and (iv) a list of target species to be queried on GenBank to capture any sequences published since the December 2015 Midori dataset was generated.

For this study, 72 recently published [34) and 7 unpublished partial mitochondrial mammal genomes (Accession Numbers MH464789, MH464790, MH464791, MH464792, MH464793, MH464794, MH464795, MH464796, MH464797, MH464798, MH464799, MH464800, MH464801) were added as input (iii). A list of 103 mammal species known to be present in the sampling area plus *Homo sapiens* and our positive control *Myodes glareolus* was added as input (iv).

With the above inputs, the seven curation steps are: 1) remove sequences not identified to species; 2) add extra sequences from optional inputs (iii) and (iv) above; 3) trim the sequences to leave only the target amplicon; 4) remove sequences with ambiguities; 5) compare species names from the Midori dataset to the reference taxonomy from input (ii) and replace with a consensus taxonomy; 6) identify and remove putatively mislabelled sequences; 7) dereplicate sequences, retaining one haplotype per species.

The script is split into four modules, allowing optional manual curation at three key steps. The steps covered by each of the four modules are summarized in Table 2. The main programs used are highlighted and cited in the text where relevant, but many intermediate steps used common UNIX tools and unpublished lightweight utilities freely available from GitHub (Table 3).

Module 1 - The first step is to select the tetrapod sequences from the Midori database for each of the four selected loci (input (i) above). This, and the subsequent step to discard sequences without strict binomial species names and reduce subspecies identifications to species-level, are made possible by the inclusion of the full NCBI taxonomic classification of each sequence in the FASTA header by the Midori pipeline. The headers of the retained sequences are then reformatted to include just the species name and GenBank accession separated by underscores. If desired, additional sequences from local FASTA files are now added to the Midori set (input (iii)). The headers of these FASTA files are required to be in the same format. Next, optional queries are made to the NCBI GenBank and RefSeq databases for each species in a provided list (input (iv)) for each of the four target loci, using NCBI's Entrez Direct [35]. Matching sequences are downloaded in FASTA format, sequences prefixed as "UNVERIFIED" are discarded, the headers are simplified as previously, and those sequences not already in the Midori set are added. Trimming each sequence down to the relevant target marker was carried out in a two-step process in which *usearch* (*-search_pcr*) was used to select sequences where both primers were present, and these were in turn used as a reference dataset for *blastn* to select partially matching sequences from the rest of the dataset [36; 37]. Sequences with a hit length of at least 90% of the expected marker length were retained by extracting the relevant subsequence based on the BLAST hit co-ordinates. Sequences with ambiguous bases were discarded at this stage. In the final step in module 1, a multiple-sequence alignment was generated with MAFFT (MAFFT, RRID:SCR_011811) [38; 39] for each partially curated amplicon dataset (for the SATIVA step

below). The script then breaks to allow the user to check for any obviously problematic sequences that should be discarded before continuing.

Module 2 - The species labels of the edited alignments are compared with the reference taxonomy (input (ii)). Any species not found is queried against the Catalogue of Life database (CoL) via *taxize* in case these are known synonyms, and the correct species label and classification is added to the reference taxonomy. The original species label is retained as a key to facilitate sequence renaming, and a note is added to indicate its status as a synonym. Finally, the genus name of any species not found in the CoL is searched against the consensus taxonomy, and if found, the novel species is added by taking the higher classification levels from one of the other species in the genus. Orphan species labels are printed to a text file, and the script breaks to allow the user to check this list and manually create classifications for some or all if appropriate.

Module 3 - This module begins by checking for any manually generated classification files (from the end of Module 2) and merging them with the reference taxonomy from Module 2. Any remaining sequences with unverifiable classifications are removed at this step. The next steps convert the sequences and taxonomy file to the correct formats for SATIVA [29], which detects possibly mislabelled sequences by generating a maximum likelihood phylogeny from the alignment in Module 1 and comparing each sequence's taxonomy against its phylogenetic neighbors. Sequence headers in the edited MAFFT alignments are reformatted to include only the GenBank accession, and a taxonomy key file is generated with the correct classification listed for each accession number. In cases where the original species label is found to be a synonym, the corrected label is used. Putatively mislabelled sequences in each amplicon are then detected with SATIVA, and the script breaks to allow inspection of the results. The user may choose to make appropriate edits to the taxonomy key file or list of putative mislabels at this point.

Module 4 - Any sequences that are still flagged as mislabelled at the start of the fourth module are deleted from the SATIVA input alignments, and all remaining sequences are relabelled with the correct species name and accession. A final consensus taxonomy file is generated in the format required by *PROTAX*. Alignments are subsequently unaligned prior to species-by-species selection of a single representative per unique haplotype. Sequences that are the only representative of a species are automatically added to the final database. Otherwise, all sequences for each species are extracted in turn, aligned with MAFFT, and collapsed to unique haplotypes with *collapsetypes_4.6.pl* (zero differences allowed; [40]). Representative sequences are then unaligned and added to the final database.

iDNA samples

We used 242 collections of haematophagous terrestrial leeches from Deramakot Forest Reserve in Sabah, Malaysian Borneo stored in RNA fixating saturated ammonium sulfate solution as samples. Each sample consisted of one to 77 leech specimens (median 4). In total, 1532 leeches were collected, exported under the permit (JKM/MBS.1000-2/3 JLD.2 (8)

issued by the Sabah Biodiversity Council), and analysed at the laboratories of the Leibniz-IZW.

Laboratory workflow

The laboratory workflow is designed to both minimize the risk of sample cross-contamination and to aid identification of any instances that do occur. All laboratory steps (extraction, pre and post PCR steps, sequencing) took place in separate laboratories and no samples or materials were allowed to re-enter upstream laboratories at any point in the workflow. All sample handling was carried out under specific hoods that were wiped with bleach, sterilized, and UV irradiated for 30 minutes after each use. All labs are further UV irradiated for four hours each night.

DNA extraction

DNA was extracted from each sample in bulk. Leeches were cut into small pieces with a fresh scalpel blade and incubated in lysate buffer (proteinase K and ATL buffer at a ratio of 1:10; 0.2 ml per leech) overnight at 55 °C (12 hours minimum) in an appropriately sized vessel for the number of leeches (2 or 5 ml reaction tube). For samples with more than 35 leeches, the reaction volume was split in two and recombined after lysis.

Each lysate was split into two *extraction replicates* (A and B; maximum volume 600 µl) and all further steps were applied to these independently. We followed the DNeasy 96 Blood & Tissue protocol for animal tissues (Qiagen, Hilden -Germany) on 96 plates for cleanup. DNA was eluted twice with 100 µl TE buffer. DNA concentration was measured with PicoGreen dsDNA Assay Kit (Quant-iT, ThermoFisherScientific, Waltham -USA) in 384-well plate format using an appropriate plate reader (200 PRO NanoQuant, Tecan Trading AG, Männedorf -Switzerland). Finally, all samples were diluted to a maximum concentration of 10 ng/µl.

Two-round PCR protocol

We amplified three mitochondrial markers – a short 93 bp fragment of *16S* rRNA (*16S*), a 389 bp fragment of *12S* rRNA (*12S*), and a 302 bp fragment of cytochrome b (*CytB*). For each marker, we ran a two-round PCR protocol (Figs. 1, 2). The primers were chosen on the expectation of successful DNA amplification over a large number of tetrapod species [41; 42], and we tested the fit of candidate primers on an alignment of available mitochondrial sequences of 134 Southeast-Asian mammal species. Primer sequences are in Table 1.

Primer modification. – We modified primers of the three markers to avoid the production of unlabelled PCR products, to allow the detection and deletion of tag-jumping events [43], and to reduce the cost of primers and library preparation. We used two rounds of PCR. The first round amplified the target gene and attached one of 25 different ‘twin-tag’ pairs (*tag 1*), identifying the sample within a given PCR. By ‘twin-tag,’ we mean that both the forward and reverse primers were given the *same* sample-identifying sequence (‘tags’) added as primer extensions (Fig. 2). The tags differed with a minimum pairwise distance of three nucleotides ([43]; Supplemental Table 1). These primers also contained different forward

and reverse sequences (*Read 1* & *Read 2 sequence primers*) (Supplemental Table 1) to act priming sites for the second PCR round (Fig. 2).

The second round added the Illumina adapters for sequencing and attached one of 20 twin-tag pairs (*tag 2*) identifying the PCR, with a minimum pairwise distance of three [44]. These primers also contained the Illumina P5 and P7 adapter sequences (Fig. 2). Thus no unlabelled PCR products were ever produced, and the combination of *tags 1* and *2* allowed the pooling of up to 480 (=24 X 20) samples in a single library preparation step (one *tag 1* was reserved for controls). Twin tags allowed us later to detect and delete tag jumping events [43] (Fig. 2).

Cycle number considerations. – Because we know that our target DNA is at low concentration in the samples, we are faced with a trade-off between (1) using fewer PCR cycles (e.g. 30) to minimise amplification bias (caused by some target DNA binding better to the primer sequences and thus outcompeting other target sequences that bind less well [45]) and (2) using more PCR cycles (e.g. 40) to ensure that low-concentration target DNA is sufficiently amplified in the first place. Rather than choose between these two extremes, we ran both low- and high-cycle protocols and sequenced both sets of amplicons.

Thus, each of the two *extraction replicates* A and B was split and amplified using different cycle numbers (*PCR replicates* 1 and 2) for a total of four (= 2 *extraction replicates* x 2 *PCR replicates* -> *A1*/*A2* and *B1*/*B2*) replicates per sample per marker (Fig. 1). For *PCR replicates* A1/B1, we used 30 cycles in the first PCR round to minimize the effect of amplification bias. For *PCR replicates* A2/B2, we used 40 cycles in the first PCR round to increase the likelihood of detecting species with very low input DNA (Fig. 1).

PCR protocol. – The first-round PCR reaction volume was 20 µl, including 0.1 µM primer mix, 0.2 mM dNTPs, 1.5 mM $MgCl_2$, 1x PCR buffer, 0.5 U AmpliTaq Gold™ (Invitrogen, Karlsruhe - Germany), and 2 µl of template DNA. Initial denaturation was 5 minutes at 95°C, followed by repeated cycles of 30 seconds at 95°C, 30 seconds at 54°C, and 45 seconds at 72°C. Final elongation was 5 minutes at 72°C. Samples were amplified in batches of 24 plus a negative (water) and a positive control (bank vole, *Myodes glareolus* DNA). All three markers were amplified simultaneously in individual wells for each batch of samples in a single PCR plate. Non-target by-products were removed as required from some *12S* PCRs by purification with magnetic Agencourt AMPure beads (Beckman Coulter, Krefeld -Germany).

In the second-round PCR, we used the same PCR protocol as above with 2 µl of the product of the first-round PCR and 10 PCR cycles.

Quality control and sequencing

Amplification was visually verified after the second-round PCR by gel electrophoresis on 1.5% agarose gels. Controls were additionally checked with a TapeStation 2200 (D1000 ScreenTape assay, Agilent, Waldbronn -Germany). All samples were purified with AMPure beads, using a bead-to-template ratio of 0.7:1 for *12S* and *CytB* products, and a ratio of 1:1 for *16S* products. DNA concentration was measured with PicoGreen dsDNA as described

above. Sequencing libraries were made by equimolar pooling of all positive amplifications; final concentrations were between 2 and 4 nmol. Because of different amplicon lengths and therefore different binding affinities to the flow cell, *12S* and *CytB* products were combined in a single library, whereas positive *16S* products were always combined in a separate library. 12S/CytB libraries were sequenced independently from 16S libraries. Apart from our negative controls, we did not include samples that did not amplify, as this would have resulted in highly diluted libraries. Up to 11 libraries were sequenced on each run of Illumina MiSeq, following standard protocols. Libraries were sequenced with MiSeq Reagent Kit V3 (600 cycles, 300 bp paired-end reads) and had a final concentration of 11 pM spiked with 20 to 30% of PhiX control.

Bioinformatics workflow

Read processing

Although the curation of the reference databases is our main focus, it is just one part of the bioinformatics workflow for e/iDNA metabarcoding. A custom bash script was used to process raw basecall files into demultiplexed, cleaned, and dereplicated reads in FASTQ format on a run-by-run basis. All runs and amplicons were processed with the same settings unless otherwise indicated. *bcl2fastq* (Illumina) was used to convert the basecall file from each library to paired-end FASTQ files, demultiplexed into the separate PCRs via the *tag 2* pairs, allowing up to 1 mismatch in each *tag 2.* Each FASTQ file was further demultiplexed into samples via the *tag 1* pairs using *AdapterRemoval* (AdapterRemoval, RRID:SCR_011834) [46], again allowing up to 1 mismatch in each tag. These steps allowed reads to be assigned to the correct samples.

In all cases, amplicons were short enough to expect paired reads to overlap. For libraries with more than 1000 reads pairs were merged with *usearch* (*-fastq_mergepairs*; [47; 48]), and only successfully merged pairs were retained. For libraries with more than 500 merged pairs the primer sequences were trimmed away with *cutadapt* (cutadapt, RRID:SCR_011841) [49], and only successfully trimmed reads at least 90% of expected amplicon length were passed to a quality filtering step with *usearch* (*-fastq_filter*). Lastly, reads were dereplicated with *usearch* (*-derep_fulllength*), and singletons were discarded. The number of replicates that each unique sequence represented was also added to the read header at this step (option *-sizeout*). The number of reads processed at each step for each sample are reported in a standard tab delimited txt-file.

Taxonomic assignment

The curated reference sequences and associated taxonomy were used for *PROTAX* taxonomic assignment of the dereplicated reads [24; 31]. *PROTAX* gives unbiased estimates of placement probability for each read at each taxonomic rank, allowing assignments to be made to a higher rank even when there is uncertainty at the species level. In other words, and unlike other taxonomic assignment methods, *PROTAX* can estimate the probability that a sequence belongs to a taxon that is not present in the reference database. This was considered an important feature due to the known incompleteness of the reference

databases for tetrapods in the sampled location. As other studies have compared *PROTAX* with more established methods, e.g. MEGAN [30] (see [4; 24]), it was beyond the scope of this study to evaluate the performance of *PROTAX.*

Classification with *PROTAX* is a two-step process. Firstly, PROTAX selected a subset of the reference database that was used as training data to parameterise a *PROTAX* model for each marker, and secondly, the fitted models were used to assign four taxonomic ranks (species, genus, family, order) to each of the dereplicated reads, along with a probability estimate at each level. We also included the best similarity score of the assigned species or genus, mined from the LAST results (see below) for each read. This was helpful for flagging problematic assignments for downstream manual inspection, i.e. high probability assignments based on low similarity scores (implying that there are no better matches available) and low probability assignments based on high similarity scores (indicates conflicting database signal from several species with highly similar sequences).

Fitting the *PROTAX* model followed Somervuo et al. [31] except that 5000 training sequences were randomly selected for each target marker due to the large size of the reference database. In each case, 4500 training sequences represented a mix of known species with reference sequences (conspecific sequences retained in the database) and known species without reference sequences (conspecific sequences omitted, simulating species missing from the database), and 500 sequences represented previously unknown lineages distributed evenly across the four taxonomic levels (i.e. mimicked a mix of completely novel species, genera, families and orders). Pairwise sequence similarities of queries and references were calculated with LAST [50] following the approach of Somervuo et al. [31]. The models were weighted towards the Bornean mammals expected in the sampled area by assigning a prior probability of 90% to these 103 species and a 10% probability to all others ([31]; Supplemental Table 2). In cases of missing interspecific variation, this helped to avoid assignments to geographically impossible taxa, especially in case of the very short 93 bp fragment of *16S*. Maximum *a posteriori* (MAP) parameter estimates were obtained following the approach of Somervuo et al. [24], but the models were parameterised for each of the four taxonomic levels independently, with a total of five parameters at each level (four regression coefficients and the probability of mislabelling).

Dereplicated reads for each sample were then classified using a custom bash script on a run-by-run basis. For each sample, reads in FASTQ format were converted to FASTA, and pairwise similarities were calculated against the full reference sequence database for the applicable marker with LAST (LAST, RRID:SCR_006119). Assignments of each read to a taxonomic node based on these sequence similarities were made using a Perl script and the trained model for that level. The taxonomy of each node assignment was added with a second Perl script for a final table including the node assignment, probability, taxonomic level, and taxonomic path for each read. Read count information was included directly in the classification output via the size annotation added to the read headers during dereplication. All Perl scripts to convert input files into the formats expected by *PROTAX*, *R*

code for training the model following Somervuo et al. [31], and Perl scripts for taxonomic assignment were provided by P. Somervuo (personal communication).

Acceptance criteria

In total we had twelve PCR reactions per sample: two *extraction replicates A* and *B* X two *PCR replicates* 1 and 2 per extraction replication X the three markers (Fig. 1). We applied two different acceptance criteria to the data with different stringency regimes. One more naive one that accepted any two positives out of the twelve *PCR replicates* (from now on referred to as lax), and one stringent one that only accepted taxonomic assignments that were positively detected in both *extraction replicates* (*A* & *B*, Fig. 3). Our lax approach refers to one of the approaches of Ficetola et al. [19] where they evaluated different statistical approaches developed to estimate occupancy in the presence of observational errors and has been applied in other studies (e.g. [13]). The reason for conservatively omitting assignments that appeared in only one *extraction replicate* was to rule out sample cross-contamination during DNA extraction. In addition, we only accepted assignments with ten or more reads per marker, if only one marker was sequenced. If a species was assigned in more than one marker (e.g. *12S* and *16S*), we accepted the assignment even if in one sequencing run the number of reads was below ten.

Due to the imperfect PCR amplification of markers (the small *16S* fragment amplified better than the longer *CytB* fragment) and missing reference sequences in the database or shared sequence motifs between species, reads sometimes were assigned to species level for one marker but only to genus level for another marker. Thus, the final identification of species could not be automated, and manual inspection and curation was needed. For each assignment, three parameters were taken into consideration: number of sequencing reads, the mean probability estimate derived from *PROTAX*, and the mean sequence similarity to the reference sequences based on LAST.

Shot-gun sequencing to quantify mammalian DNA content

As the success of the metabarcoding largely depends on the mammal DNA quantity in our leech bulk samples we quantified the mammalian DNA content in a subset of 58 of our leech samples using shotgun sequencing. Extracted DNA was sheared with a Covaris M220 focused-ultra-sonicator to a peak target size of 100-200 bp, and re-checked for size distribution. Double-stranded Illumina sequencing libraries were prepared according to a ligation protocol designed by Fortes and Paijmans [51] with single 8 nt indices. All libraries were pooled equimolarly and sequenced on the MiSeq using the v3 150-cycle kit. We demultiplexed reads using bcl2fastq and cutadapt for trimming the adapters. We used BLAST (NCBI BLAST, RRID:SCR_004870) search to identify reads and applied Metagenome Analyzer MEGAN (MEGAN, RRID:SCR_011942) [30] to explore the taxonomic content of the data based on the NCBI taxonomy. Finally we used KRONA (Krona, RRID:SCR_012785) [52] for visualisation of the results.

Findings & Discussion

Database curation

The Midori UNIQUE database (December 2015 version) contains 1,019,391 sequences across the four mitochondrial loci of interest (*12S*: 66,937; *16S*: 146,164; *CytB*: 223,247; *COI*: 583,043), covering all Metazoa. Of these, 258,225 (25.3%) derive from the four tetrapod classes (Amphibia: 55,254; Aves: 51,096; Mammalia: 101,106; Reptilia: 50,769). The distribution of these sequences between classes and loci, and the losses at each curation step are shown in Figure 4. In three of the four classes, there is a clear bias towards *CytB* sequences, with over 50% of sequences derived from this locus. In both Aves and Mammalia, the *16S* and *12S* loci are severely underrepresented at less than 10% each, while for Reptilia, *COI* is the least sequenced locus in the database.

The numbers of sequences and rates of loss due to our curation steps varied among taxonomic classes and the four loci, although losses were observed between steps in almost all instances. The most significant losses followed amplicon trimming and removal of non-unique sequences. Amplicon trimming led to especially high losses in Amphibia and *16S*, indicating that data published on GenBank for this class and marker do not generally overlap with our amplicons. Meanwhile, the high level of redundancy in public databases was highlighted by the significant reduction in the number of sequences during the final step of removing redundant sequences – in all cases over 10% of sequences was discarded, with some losses exceeding 50% (Mammalia: *COI*, *CytB*, *16S*; Amphibia: *16S*).

Data loss due to apparent mislabelling ranged between 1.9% and 7.4% and was thus generally higher than similar estimates for curated microbial databases [29]. SATIVA flags potential mislabels and suggests an alternative label supported by the phylogenetic placement of the sequences, allowing the user to make an appropriate decision on a case by case basis. The pipeline pauses after this step to allow such manual inspection to take place. However, for the current database, the number of sequences flagged was large (4378 in total), and the required taxonomic expertise was lacking, so all flagged sequences from non-target species were discarded to be conservative. The majority of mislabels were identified at species level (3053), but there were also significant numbers at genus (788), family (364) and order (102) level. Two to three sequences from Bornean mammal species were unflagged in each amplicon to retain the sequences in the database. This was important as in each case these were the only reference sequences available for the species. Additionally, *Muntiacus vaginalis* sequences that were automatically synonymised to *M. muntjak* based on the available information in the Catalogue of Life were revised back to their original identifications to reflect current taxonomic knowledge.

Database composition

The final database was skewed even more strongly towards *CytB* than was the raw database. It was the most abundant locus for each class and represented over 60% of sequences for both Mammalia and Reptilia. In all classes, *16S* made up less than 10% of the final database, with Reptilia *COI* also at less than 10%.

Figure 5 shows that most species represented in the curated database for any locus have just one unique haplotype against which HTS reads can be compared; only a few species have many haplotypes. The prevalence of species with 20 or more haplotypes is particularly notable in *CytB* where the four classes have between 25 (Aves) and 265 (Mammalia) species in this category. The coloured circles in Figure 5 also show that the species of the taxonomy are incompletely represented across all loci, and that coverage varies significantly between taxonomic groups. In spite of global initiatives to generate *COI* sequences [53], this marker does not offer the best species-level coverage in any class and is a poor choice for Amphibia and Reptilia (<15% of species included). Even the best performing marker, *CytB*, is not a universally appropriate choice, as Amphibia is better covered by *12S*. These differences in underlying database composition will impact the likelihood of obtaining accurate taxonomic assignment for any one species from any single marker. Further barcoding campaigns are clearly needed to fill gaps in the reference databases for all markers and all classes to increase the power of future e/iDNA studies. As the costs of HTS decrease, we expect that such gap-filling will increasingly shift towards sequencing of whole mitochondrial genomes of specimen obtained from museum collections, trapping campaigns etc. [34], reducing the effect of marker choice on detection likelihood. In the meantime, however, the total number of species covered by the database can be increased by combining multiple loci (here, up to four) and thus the impacts of database gaps on correctly detecting species can be minimized ([54]; Fig. 6).

In the present study, the primary target for iDNA sampling was the mammal fauna of Malaysian Borneo, and the 103 species expected in the sampling area represent an informative case study highlighting the deficiencies in existing databases (Fig. 7). Nine species are completely unrepresented while only slightly over half (55 species) have at least one sequence for all of the loci. Individually, each marker covers over half of the target species, but none achieves more than 85% coverage (*12S*: 75 species; *16S*: 68; *CytB*: 88; *COI*: 66). Equally striking is the lack of within-species diversity, as most of the incorporated species are represented by only a single haplotype per locus. Some of the species have large distribution ranges, so it is likely that in some cases the populations on Borneo differ genetically from the available reference sequences, possibly limiting assignment success. Only a few expected species have been sequenced extensively, and most are of economic importance to humans (e.g. *Bos taurus*, *Bubalus bubalis*, *Macaca* spp, *Paradoxurus hermaphroditus*, *Rattus* spp., *Sus scrofa*), with as many as 100 haplotypes available (*Canis lupus*). Other well-represented species (≥20 haplotypes) present in the sampling area include several Muridae (*Chiropodomys gliroides*, *Leopoldamys sabanus*, *Maxomys surifer*, *Maxomys whiteheadi*) and the leopard cat (*Prionailurus bengalensis*).

Laboratory workflow

Shotgun sequencing of a subset of our samples revealed that the median mammalian DNA content was only 0.9%, ranging from 0% to 98%. These estimates are approximate, but with more than 75% of the samples being below 5%, this shows clearly the scarcity of target DNA

in bulk iDNA samples. The generally low DNA content and the fact that the target DNA is often degraded make enrichment of the target barcoding loci necessary. We used PCR with high cycle numbers to obtain enough DNA for sequencing. However, this second step increases the risk of PCR error: artificial sequence variation, non-target amplification, and/or raising contaminations up to a detectable level.

We addressed these problems by running two *extraction replicates*, two *PCR replicates*, and a multi-marker approach. The need for *PCR replicates* has been acknowledged and addressed extensively in ancient DNA studies [16] and has also been highlighted for metabarcoding studies [19; 20; 55; 56]. Despite this, many e/iDNA studies do not carry out multiple *PCR replicates* to detect and omit potential false sequences. In addition, *extraction replicates* are seldom applied, despite the evidence that cross-sample DNA contamination can occur during DNA extraction [57; 58; 59]. We only accepted sequences that appeared in a minimum of two independent PCRs for the lax and for the stringent criterion, where it has to occur in each *extraction replicate A* and *B* (Fig. 1). The latter acceptance criterion is quite conservative and produces higher false negative rates than e.g. accepting occurrence of at least two positives. However, it also reduces the risk of accepting a false positives compared to it (see Supplemental Fig. 1. for a simulation of false positive and false negatives rates within a PCR), especially with increasing risk of false positive occurrence in a PCR for e.g. example due to higher risk of contamination etc.. Metabarcoding studies are very prone to false negatives, and downstream analyses like occupancy models for species distributions can account for imperfect detection and false negatives. However, methods for discounting false positive detections are not well developed [60]. Thus we think it is more important to avoid false positives, especially if the results will be used to make management decisions regarding rare or endangered species. In contrast, it might be acceptable to use a relaxed acceptance criterion for more common species, as long as the rate false-positives/true-positives is small and does not affect species distribution estimates. Employing both of our tested criteria researchers could flag unreliable assignments and management decisions can still use this information, but now in a forewarned way. An alternative to our acceptance criteria could be use the PCR replicates itself to model the detection probability within a sample using an occupancy framework [20; 60; 61; 62].

We used three different loci to correct for potential PCR-amplification biases. We were, however, unable to quantify this bias in this study due to the high degradation of the target mammalian DNA, which resulted in much higher overall amplification rates for *16S*, the shortest of our PCR amplicons. For *16S*, 85% of the samples amplified, whereas for *CytB* and *12S*, only 57% and 44% amplified, respectively. Also the read losses due to trimming and quality filtering were significantly lower for the *16S* sequencing runs (1.3% and 5.3% in average, Supplemental Table 3) compared to the sequencing runs for the longer fragments of *12S* and *CytB* (65.3% and 44.3% in average, Supplemental Table 3). Despite the greater taxonomic resolution of the longer *12S* and *CytB* fragments, our poorer amplification and sequencing results for these longer fragments emphasize that e/iDNA studies should

generally focus on short PCR fragments to increase the likelihood of positive amplifications of the degraded target DNA. In the case of mammal-focussed e/iDNA studies, developing a shorter (100 bp) *CytB* fragment would likely be very useful.

Another major precaution was the use of twin-tagging for both PCRs (Fig. 2). This ensures that unlabelled PCR products are never produced and allows us to multiplex a large number of samples on a single run of Illumina MiSeq run. Just 24 sample *tags 1* and 20 plate *tags 2* allow the differentiation of up to 480 samples with matching tags on both ends. The same number of individual primers would have needed longer tags to maintain enough distance between them and would have resulted in an even longer adapter-tag overhang compared to primer length. This would have most likely resulted in lower binding efficiencies due to steric hindrances of the primers. Furthermore, this would have resulted in increased primer costs. Thus our approach reduced sequencing and primer purchase costs while at the same time largely eliminating sample mis-assignment via tag jumping, because tag-jump sequences have non-matching forward and reverse *tag 1* sequences [43]. We estimated the rate of tag jumps producing non-matching *tag 1* sequences to be 1 to 5%, and these were removed from the dataset (Table 4). For our sequenced PCR plates, the rate of non-matching *tag 2* tags was 2%. These numbers are smaller than data from Zepeda-Mendoza et al. [56] who reported on sequence losses of 19% to 23% due to unused tag combinations when they tested their DAMe pipeline to different datasets built using standard blunt-end ligation technique. Although their numbers might not be one-to-one comparable to our results as they counted unique sequences, and we report on read numbers, our PCR libraries with matching barcodes seem reduce the risk of tag jumping compared to blunt-end ligation techniques. For the second PCR round, we used the same tag pair *tag 2* for all 24 samples of a PCR plate. In order to reduce cost we tested pooling these 24 samples prior to the second PCR round, but we detected a very high tag jumping rate of over 40% (Table 4), which ultimately would increase cost through reduced sequencing efficiency. Twin-tagging increases costs because of the need to purchase a larger number of primer pairs but at the same time it increases confidence in the results.

Tagging primers in the first PCR reduces the risk of cross-contamination via aerosolised PCR products. However, we would not be able to detect a contamination prior the second PCR from one plate to another, as we used the same 24 tags (*tag 1*) for all plates. Nevertheless such a contamination is very unlikely to result in any accepted false positive as it would be improbable to contaminate both the A and B replicates, given the exchange of all reagents and the time gap between the PCRs. Previous studies have shown that unlabelled volatile PCR products pose a great risk of false detections [63], a risk that is greatly increased if a high number of samples are analysed in the laboratories [13]. Also, in laboratories where other research projects are conducted, this approach allows the detection of cross-experiment contamination. Therefore, we see a clear advantage of our approach over ligation techniques when it comes to producing sequencing libraries, as the Illumina tags are

only added after the first PCR, and thus the risk of cross contamination with unlabelled PCR amplicons is very low.

Assignment results

A robust assignment of species is an important factor in metabarcoding as an incorrect identification might result incorrect management interventions. The reliability of taxonomic assignments is expected to vary with respect to both marker information content and database completeness, and this is reflected in the probability estimates provided by *PROTAX*. In a recent study, less than 10% of the mammal assignments made at species level against a worldwide reference database were considered reliable with the short *16S* amplicon, but this increased to 46% with full-length *16S* sequences [31]. In contrast, in the same study over 80% of insect assignments at species level were considered reliable with a more complete, geographically restricted database of full-length COI barcodes. A similar pattern was observed in our data during manual curation of the assignment results – there was more ambiguity in the results for the short *16S* amplicon than for other markers. However, due to the limited amount of often degraded target DNA in e/iDNA samples, short amplicons amplify much better. In our case, this had the drawback that some species lacked any interspecific variation, and thus sequencing reads shared 99%-100% identity for several species. For example, our only *16S* reference of *Sus barbatus* was 100% identical to *S. scrofa*. But as latter species does not occur in the studied area we could assign all reads manually to *S. barbatus*. In several cases we were able to confirm *S. barbatus* by additional *CytB* results, highlighting the usefulness of multiple markers.

Another advantage of multiple markers is the opportunity to fill gaps in the reference database. For example, we lacked *16S* reference sequences for *Hystrix brachyura*, and reads were assigned by *PROTAX* only to the unknown species *Hystrix* sp.. In one sample, however, almost 5000 *CytB* reads could be confidently assigned to *Hystrix brachyura*, and thus we used the *Hystrix* sp. 16S sequences in the same sample to build a consensus *16S* reference sequence for *Hystrix brachyura* for future analyses. In another example we had *CytB* reads assigned to *Mydaus javanicus*, the Sunda stink-badger in one sample but *12S* reads assigned to *Mydaus* sp. in another one. As we lacked a *12S Mydaus* reference and as there is only one *Mydaus* species on Borneo we could assume that this second sample is most likely also *Mydaus javanicus*.

We also inferred that PCR and sequencing errors resulted in reads being assigned to sister taxa. We observed that a high number of reads of a true sequence were assigned to a species and a lower number of noise sequences were assigned to a sister taxon. Such a pattern was observed for ungulates, especially deer that showed little variance in *16S*. It is hard to identify and control for such pattern automatically, and it highlights the importance of visual inspection of the results.

For the more lax criterion (two positive *PCR replicates*) we accepted 190 species assignments out of 109 leech samples. Under the stringent criterion (i.e. having positive detections in both *extraction replicates A* and *B*) we accepted about 14% assignments less;

in total 162 vertebrate detections within 95 bulk samples (Table 5). For 48% of the species frequencies did not change and almost half of the not accepted assignments were from the most frequent species *Rusa unicolor* and *Sus barbatus*. However, with the more stringent criterion we did not accept two species (1x *Macaca fascicularis* & 2x *Mydaus javanensis*). In three cases the stringent criterion would not accept assignments that could be made only to unknown species (*Macaca* sp.) (Table 5). For this genus we have two occurring species in the area. As the true occurrence of species within our leeches was unknown we cannot evaluate how many of the additional 27 detections in the lax criterion are false positives and how many might be false negatives for the stricter criterion. However, by accepting only positive *AB* assignment results, we increase the confidence of species detection, even if the total number of reads for that species was low. When it comes to rare or threated species this outweighs the risk of reporting false positives to our opinion. 48% of the assignments with the stringent criterion were present in all four *A1*, *A2*, *B1* and *B2*. 35% were present in at least three replicates (e.g. A1, A2, B1).

The mean number of reads per sample used for the taxomomic assignment varied from 162,487 *16S* reads for SeqRun01 to 7,638 *CytB* reads for SeqRun05 (Supplemental Table 4). In almost all cases, however, the number of reads of an accepted assignment was high (median= 52,386; mean= 300,996; SD= 326,883). PCR stochasticity, primer biases, multiple species in individual samples, and pooling of samples exert too many uncertainties that could bias the sequencing results [64; 65]. Thus we do not believe that raw read numbers are the most reliable indicators of tetrapod DNA quantity in iDNA samples. Replication of detection is inherently more reliable. In contrast to our expectation that higher cycle number might be necessary to amplify even the lowest amounts of target DNA, our data do not support this hypothesis. Although we observed an increase in positive PCRs for A2/B2 (the 40-cycle PCR replicates), the total number of accepted assignments in *A1/B1* and *A2/B2* samples did not differ. This indicates first that high PCR cycle numbers mainly increased the risk of false positives and second that our multiple precautions successfully minimized the acceptance of false detections.

Conclusion

Metabarcoding of e/iDNA samples will certainly become a very valuable tool in assessing biodiversity, as it allows to detect species non-invasively without the need to capture and handle the animals [66] and because sampling effort can often be greatly reduced. However, the technical and analytical challenges linked to sample types (low quantity and quality DNA) and poor reference databases have so far been insufficiently recognized. In contrast to ancient DNA studies where standardized laboratory procedures and specialized bioinformatics pipelines have been established and are followed in most cases, there is limited methodological consensus in e/iDNA studies, which reduces rigour. In this study, we present a robust metabarcoding workflow for e/iDNA studies. We hope that the provided scripts and protocols facilitate further technical and analytical developments. The use of e/iDNA metabarcoding to study the rarest and most endangered species such as the Saola is

exciting, but geneticists bear the heavy responsibility of providing correct answers to conservationists.

Availability of supporting data

Sequencing data is available in the EBI via bioproject number: PRJEB27367. All other supporting data are also available via the *GigaScience* GigaDB repository [67].

List of abbreviations

Amplicon: amplification of a short target sequence; BOLD: Barcode of Life Database; CoL: Catalogue of Life database; CytB: cytochrome b; eDNA: environmental DNA; iDNA: invertebrate-derived DNA; INSDC: International Nucleotide Sequence Database Collaboration; ITIS: Integrated Taxonomic Information System; MEGAN: Metagenome Analyzer; PCR: polymerase chain reaction; 12S: 12S rRNA; 16S: 16S rRNA.

Competing interests

None.

Acknowledgements

All authors thank the German Federal Ministry of Education and Research (BMBF FKZ: 01LN1301A) and the Leibniz-IZW for funding this study. We also thank the Sabah Forestry Department, especially Johnny Kissing, Peter Lagan and Datuk Sam Mannan for supporting the fieldwork and the Sabah Biodiversity Council for providing research, collection and export permits for this work. We are grateful to John Mathai, Seth Timothy Wong for conducting the field work and collecting the leeches. We are also grateful to Jörns Fickel, head of the Department Evolutionary Genetics of the Leibniz-IZW for continuous support and collaboration. Furthermore we would like to thank Sebastian Wieser for lab-support, Dorina Lenz and Anke Schmidt for their help and fruitful discussions. C.C.Y. Xu was also supported by the MEME Erasmus Mundus Programme in Evolutionary Biology, and by the Groningen University Fund and the Marco Polo Fund from the University of Groningen. D.W. Yu and C.C.Y. Xu were supported by the National Natural Science Foundation of China (41661144002, 31670536, 31400470, 31500305), the Key Research Program of Frontier Sciences, CAS (QYZDY-SSW-SMC024), the Bureau of International Cooperation project (GJHZ1754), the Strategic Priority Research Program of the Chinese Academy of Sciences (XDA20050202, XDB31000000), the Ministry of Science and Technology of China (2012FY110800), and the State Key Laboratory of Genetic Resources and Evolution at the Kunming Institute of Zoology.

References

[1] Gariepy TD, Lindsay R, Odgen N, Greory TR. Identifying the last supper: utility of the DNA barcode library for bloodmeal identification in ticks. Mol Ecol Res. 2012; 12: 646-52.

[2] Lee P-S, Gan HM, Clements GR, Wilson J-J. Field calibration of blowfly-derived DNA against traditional methods for assessing mammal diversity in tropical forests. Genome 2016;59: 1008-22.

[3] Calvignac-Spencer S, Merkel K, Kutzner N, et al.. Carrion fly-derived DNA as a tool for comprehensive and cost-effective assessment of mammalian biodiversity. Mol Ecol 2013; 22: 915-24.

[4] Rodgers TW, Xu CCY, Giacalcone J, et al.. Carrion fly-derived DNA metabarcoding is an effective tool for mammal surveys: Evidence from a known tropical mammal community. Mol Ecol Res. 2017; 17(6):1-13

[5] Hoffmann C, Merkel K, Sachse A, et al.. Blow flies as urban wildlife sensors. Mol Ecol Res. 2018; 18(3):502-10

[6] Schönberger AC, Wagner S, Tuten HC, et al.. Host preferences in host-seeking and 632 blood-fed mosquitoes in Switzerland. Med Vet Entomol. 2015; 30(1): 39-52.

[7] Townzen JS, Brower AVZ, Judd DD. Identification of mosquito bloodmeals using mitochondrial cytochrome oxidase subunit I and cytochrome b gene sequences. Med Vet Entomol. 2008; 22. 386-93.

[8] Kocher A, Thoisy B, Catzeflies F, et al.. iDNA screening: Disease vectors as vertebrate samplers. Mol Ecol. 2017; 26(22): 6478-86.

[9] Taylor L, Cummings RF, Velten R, et al.. Host (Avian) Biting Preference of Southern California Culex Mosquitoes (Diptera: Culicidae). J Med Entomol. 2012; 49(3): 687-96.

[10] Schnell IB, Thomsen PF, Wilkinson N, et al.. Screening mammal biodiversity using DNA from leeches. Curr Biol. 2012, 22(8): R262—3.

[11] Tessler M, Weiskopf SR, Berniker L, et al.. Bloodlines: mammals, leeches, and conservation in southern Asia. Syst Biodivers. 2018; 1-9.

[12] Weiskopf SR, McCarthy KP, Tessler M, et al.. Using terrestrial haematophagous leeches to enhance tropical biodiversity monitoring programmes in Bangladesh. J Appl Ecol. 2018: 1-11.

[13] Schnell IB, Bohmann K, Schultze SE, et al.. Debugging diversity - a pan-continental exploration of the potential of terrestrial blood-feeding leeches as a vertebrate monitoring tool. Mol Ecol Res. 2018.

[14] Calvignac-Spencer S, Leendertz FH, Gilbert MT, Schubert G. An invertebrate stomach's view on vertebrate ecology: certain invertebrates could be used as "vertebrate samplers" and deliver DNA-based information on many aspects of vertebrate ecology. BioEssays. 2013; 35(11): 1004-13.

[15] Schnell IB, Sollmann R, Calvignac-Spencer S, et al.. iDNA from terrestrial haematophagous leeches as a wildlife surveying and monitoring tool – prospects, pitfalls and avenues to be developed. Front Zool. 2015; 12:24.

[16] Pääbo S, Poinar H, Serre D, et al.. Genetic analyses from ancient DNA. Annu Rev Genet. 2004; 38: 645-79.

[17] Hofreiter M, Paijmans JL, Goodchild H, et al. The future of ancient DNA: Technical advances and conceptual shifts. BioEssays. 2015; 37(3): 284-93.

[18] Cristescu ME, Hebert , PDN. Uses and Misuses of Environmental DNA in Biodiversity Science and Conservation. Cristescu, Melania E und Hebert, Paul D N. 1, 2018, Annu Rev Ecol Evol Syst 2018; 49.

[19] Ficetola GF, Pansu J, Bonin A, et al.. Replication levels, false presences and the estimation of the presence/absence from eDNA metabarcoding data. Mol Ecol Res. 2014; 15(3): 543-56.

[20] Ficetola GF, Taberlet P., Coissac E. How to limit false positives in environmental DNA and metabarcoding? Mol Ecol Res. 2016; 16(3): 604-7.

[21] Dalton R. Still looking for that woodpecker. Nature 2010; 463: 718-9.

[22] Bazinet AL, Cummings MP. A comparative evaluation of sequence classification programs. BMC bioinformatics. 2012; 13(1): 92.

[23] Richardson RT, Bengtsson-Palme J, Johnson RM. Evaluating and optimizing the performance of software commonly used for the taxonomic classification of DNA metabarcoding sequence data. Mol Ecol Res. 2017; 17(4): 760-9.

[24] Somervuo P, Koskela S, Pennanen J, Henrik Nilsson R, Ovaskainen O. Unbiased probabilistic taxonomic classification for DNA barcoding. Bioinformatics. 2016; 32(19): 2920-7.

[25] Quast C, Pruesse E, Gerken J, et al.. SILVA Databases. In: Nelson KE (eds) Encyclopedia of Metagenomics. Springer, Boston 2015; 626-35.

[26] Nilsson RH, Ryberg M. Taxonomic Reliability of DNA Sequences in Public Sequence Databases: A Fungal Persepctive. PLoS ONE. 1, 2006; 1.

[27] Forster P. To Err is Human. Ann Hum Gen 2003; 67: 2-4.

[28] Harris JD. Can you bank on GenBank? Trends Ecol Evol. 2003; 18: 317-9. doi:10.1016/S0169-5347(03)00150-2.

[29] Kozlov AM, Zhang J, Yilmaz P, Glöckner FO, Stamatakis A. Phylogeny-aware identification and correction of taxonomically mislabeled sequences. Nucleic Acids Res. 2016; 44(11): 5022-33.

[30] Huson DH, Auch AF, Qi J, Schuster SC. MEGAN analysis of metagenomic data. Genome Res. 2007; 17(3): 377-86.

[31] Somervuo P, Yu DW, Xu CC, Ji Y, et al.. Quantifying uncertainty of taxonomic placement in DNA barcoding and metabarcoding. Methods Ecol Evol. 2017; 8(4): 398-407.

[32] Machida RJ, Leray M, Ho SL, Knowlton N. Metazoan mitochondrial gene sequence reference datasets for taxonomic assignment of environmental samples. Sci Data. 2017; 4: 170027.

[33] Chamberlain SA, Szöcs E. taxize: taxonomic search and retrieval in R. Version 2. F1000Res. 2013; 2: 191.

[34] Salleh FM, Ramos-Madrigal J, Peñaloza F, et al.. An expanded mammal mitogenome dataset from Southeast Asia. GigaScience. 2017; 6(8): 1-8

[35] Kans, Jonathan. Entrez Direct: E-utilities on the UNIX Command Line. In: Entrez Programming Utilities Help [Internet]. Bethesda (MD): National Center for Biotechnology Information (US). 2010.

[36] Altschul SF, Gish W, Miller W, Myers EW, Lipman DJ. Basic local alignment search tool. Journal of molecular biology. 1990; 215(3):, 403-10.

[37] Camacho C, Coulouris G, Avagyan V, et al. BLAST+: architecture and applications. BMC bioinformatics. 2009; 10(1): 421.

[38] Katoh K, Standley DM. (2013). MAFFT multiple sequence alignment software version 7: improvements in performance and usability. Mol Biol Evol. 2013; 30(4): 772-80.

[39] Katoh K, Misawa K, Kuma KI, Miyata T. MAFFT: a novel method for rapid multiple sequence alignment based on fast Fourier transform. Nucleic Acids Res. 2002; 30(14): 3059-66.

[40] Chesters D. (2013) collapsetypes.pl http://sourceforge.net/projects/collapsetypes/ Accessed 1st Feb 2019

[41] Kocher TD, Thomas WK, Meyer A, et al.. Dynamics of mitochondrial DNA evolution in animals: amplification and sequencing with conserved primers. Proc. Natl. Acad. Sci. U.S.A.. 1989; 86(16): 6196-6200.

[42] Taylor PG. Reproducibility of ancient DNA sequences from extinct Pleistocene fauna. Mol Biol Evol. 2996; 13(1): 283-5.

[43] Schnell IB, Bohmann K, Gilbert MTP. Tag jumps illuminated–reducing sequence-to-sample misidentifications in metabarcoding studies. Mol Ecol Res. 2015; 15(6): 1289-1303.

[44] Faircloth BC, Glenn TC. Not all sequence tags are created equal: designing and validating sequence identification tags robust to indels. PloS One. 2012; 7(8): e42543

[45] Murray DC, Coghlan ML, Bunce M. From benchtop to desktop: important considerations when designing amplicon sequencing workflows. PLoS One. 2015; 10(4): e0124671.

[46] Schubert M, Lindgreen S, Orlando L. AdapterRemoval v2: rapid adapter trimming, identification, and read merging. BMC Research Notes 2016; 9.

[47] Edgar RC. Search and clustering orders of magnitude faster than BLAST. Bioinformatics. 2010; 26(19): 2460-2461.

[48] Edgar RC, Flyvbjerg H. Error filtering, pair assembly and error correction for next-generation sequencing reads. Bioinformatics. 2015; 31(21): 3476-82.

[49] Martin M. Cutadapt removes adapter sequences from high-throughput sequencing reads. EMBnet. Journal. 2011; 17(1): 10-12.

[50] Kiełbasa SM, Wan R, Sato K, Horton P, Frith MC. Adaptive seeds tame genomic sequence comparison. Genome Res. 2011; 21(3): 487-493.

[51] Fortes GG, Paijmans JLA. Analysis of Whole Mitogenomes from Ancient Samples. In: Kroneis T (eds). Whole Genome Amplification: Methods and Protocols. Springer New York 2015; 179-195.

[52] Ondov BD, Bergman NH, Phillippy AM. Interactive metagenomic visualization in a Web browser. BMC Bioinformatics 2011; 12: 385.

[53] Ratnasingham S, Hebert PDN. BOLD: The Barcode of Life Data System (www.barcodinglife.org). Mol Ecol Notes. 2007; 3: 355-64.

[54] Evans NT, Li Y, Renshaw MA, et al. Fish community assessment with eDNA metabarcoding: effects of sampling design and bioinformatic filtering. Can J Fish Aquat Sci. 2017; 74(9):, 1362-74.

[55] Bonin A, Taberlet P, Zinger L, Coissac E. Environmental DNA: For Biodiversity Research and Monitoring. 1st ed. Oxford University Press; 2018.

[56] Zepeda-Mendoza ML, Bohmann K, Baez A, Gilbert M., DAMe: a toolkit for the initial processing of datasets with PCR replicates of double-tagged amplicons for DNA metabarcoding analyses. BMC Res Notes 2016; 9.

[57] Racimo F, Renaud G, Slatkin M. Joint estimation of contamination, error and demography for nuclear DNA from ancient humans. PLoS Genet. 2016; 12(4): e1005972.

[58] Orlando L, Gilbert MTP, Willerslev E. Reconstructing ancient genomes and epigenomes. Nat Rev Genet 2015; 16(7): 395

[59] Laurin-Lemay S, Brinkmann H, Philippe H. Origin of land plants revisited in the light of sequence contamination and missing data. Current Biology. 2012; 22(15): R593-4.

[60] Lahoz-Monfort JJ, Guillera-Arroita G, Tingley R. Statistical approaches to account for false-positive errors in environmental DNA samples. Mol Ecol Res. 2015; 16: 673-85.

[61] Dorazio RM, Erickson RA. ednaoccupancy: An r package for multiscale occupancy modelling of environmental DNA data. Mol Ecol Res. 2018; 18: 368-80.

[62] Guillera-Arriota G, Lahoz-Monfort JJ, van Rooyen AR, et al.. Dealing with false-positives and false-negative errors about species occurrence at multiple levels. Methods Ecol Evol. 2017; 8: 1081-91

[63] Kwok S, Higuchi R. Avoiding false positives with PCR. Nature. 1989; 339: 237-8.

[64] Bush A, Sollmann R, Wilting A, et al. Connecting Earth observation to high-throughput biodiversity data. Nat Ecol Evol 2017; 1(7): 0176

[65] Piñol J, Mir G, Gomez-Polo P, Agustí N. Universal and blocking primer mismatches limit the use of high-throughput DNA sequencing for the quantitative metabarcoding of arthropods., Mol Ecol Res.2014; 15: 819-30.

[66] Nichols RV, Vollmers C, Newsom L, et al.. Minimizing polymerase biases in metabarcoding. Mol Ecol Res. 2018; 18: 927-39.
[67] Axtner J; Crampton-Platt A; Hörig L; Mohamed A; Xu CCY; Yu DW; Wilting A (2019): Supporting data for "An efficient and improved laboratory workflow and tetrapod database for larger scale eDNA studies" GigaScience Database. http://dx.doi.org/10.5524/100570

Table 1: Sequence motifs that compose the 25 different target primers for the first and the second PCR. First PCR primers consist of target specific primer followed by an overhang out of sample specific *tag 1* and *read 1* and *read 2* sequencing primer, respectively. The second PCR primers consist of the *read 1* or the *read 2* sequencing primer followed by an plate specific *tag 2* and the P5 and P7 adapters, respectively (see also Fig. 2).

Name	Sequence	Reference
tag A	TGCAT	Faircloth & and Glenn 2012
tag B	TCAGC	Faircloth & and Glenn 2012
tag C	AAGCG	Faircloth & and Glenn 2012
tag D	ACAAG	Faircloth & and Glenn 2012
tag E	AGTGG	Faircloth & and Glenn 2012
tag F	TTGAC	Faircloth & and Glenn 2012
tag G	CCTAT	Faircloth & and Glenn 2012
tag H	GGATG	Faircloth & and Glenn 2012
tag I	CTAGG	Faircloth & and Glenn 2012
tag K	CACCT	Faircloth & and Glenn 2012
tag L	GTCAA	Faircloth & and Glenn 2012
tag M	GAAGT	Faircloth & and Glenn 2012
tag N	CGGTT	Faircloth & and Glenn 2012
tag O	ACCGA	Faircloth & and Glenn 2012
tag P	ACGTC	Faircloth & and Glenn 2012
tag Q	AGACT	Faircloth & and Glenn 2012
tag R	AGGAA	Faircloth & and Glenn 2012
tag S	ATTCC	Faircloth & and Glenn 2012
tag T	CAATC	Faircloth & and Glenn 2012
tag V	CATGA	Faircloth & and Glenn 2012
tag W	CCACA	Faircloth & and Glenn 2012
tag X	GCTTA	Faircloth & and Glenn 2012
tag Y	GGTAC	Faircloth & and Glenn 2012
tag Z	AACAC	Faircloth & and Glenn 2012
Tag Control	ATCTG	Faircloth & and Glenn 2012
CytB-fw	AAAAAGCTTCCATCCAACATCTCAGCATGATGAAA	Kocher et al. 1989
CytB-rv	AAACTGCAGCCCCTCAGAATGATATTTGTCCTCA	Kocher et al. 1989
16S-fw	CGGTTGGGGTGACCTCGGA	Taylor 1996
16S-rv	GCTGTTATCCCTAGGGTAACT	Taylor 1996
12S-fw	AAAAAGCTTCAAACTGGGATTAGATACCCCACTAT	Kocher et al. 1989
12S-rv	TGACTGCAGAGGGTGACGGGCGGTGTGT	Kocher et al. 1989
Read 1 sequence primer	ACACTCTTTCCCTACACGACGCTCTTCCGATCT	Illumina Document # 1000000002694 v03
Read 2 sequence primer	GTGACTGGAGTTCAGACGTGTGCTCTTCCGATCT	Illumina Document # 1000000002694 v03
P5 adapter	AATGATACGGCGACCACCGAGATCTACAC	Illumina Document # 1000000002694 v03
P7 adapter	CAAGCAGAAGACGGCATACGAGAT	Illumina Document # 1000000002694 v03

898 **Table 2:** Main steps undertaken by each module of the database curation script.

MODULE	STEPS
Module 1	Extract subset of raw Midori database for query taxon and loci.
	Remove sequences with non-binomial species names, reduce subspecies to species labels
	Add local sequences (optional)
	Check for relevant new sequences for list of query species on NCBI (GenBank and RefSeq) (optional)
	Select amplicon region and remove primers
	Remove sequences with ambiguous bases
	Align
	End of module: Optional check of alignments
Module 2	Compare sequence species labels with taxonomy
	Non-matching labels queried against Catalogue of Life to check for known synonyms
	Remaining mismatches kept if genus already exists in taxonomy, otherwise flagged for removal
	End of module: Optional check of flagged species labels
Module 3	Discard flagged sequences
	Update taxonomy key file for sequences found to be incorrectly labelled in Module 2
	Run SATIVA
	End of module: Optional check of putatively mislabelled sequences
Module 4	Discard flagged sequences
	Finalise consensus taxonomy and relabel sequences with correct species label and accession number
	Select one representative sequence per haplotype per species

Table 3: GNU core utilities and other lightweight tools used for manipulation of text and sequence files

TOOL	FUNCTION	SOURCE
awk, cut, grep, join, sed, sort, tr	Processing text files	GNU core utilities
seqbuddy	Processing FASTA/Q files	https://github.com/biologyguy/BuddySuite
seqkit	Processing FASTA/Q files	https://github.com/shenwei356/seqkit
seqtk	Processing FASTA/Q files	https://github.com/lh3/seqtk
tabtk	Processing tab-delimited text files	https://github.com/lh3/tabtk

902 **Table 4:** Number of reads per sequencing run and the numbers of reads with matching, non-matching or unidentifiable tags for seven of the
903 eight sequencing runs*.

	total reads	matching tag 2 reads	non-matching tag 2 reads	%[1]	matching tag 1 reads	non-matching tag 1 reads	%[2]	erroneous tag 1 reads	%[2]
**SeqRun01**	18,438,517	18,102,702	282,419	1.5	17,514,515	451,028	2.5	137,159	0.8
**SeqRun02**	25,385,558	24,596,380	626,245	2.5	23,426,084	612,045	2.5	558,251	2.3
**SeqRun03**	14,875,796	14,393,884	343,528	2.3	13,766,187	426,181	3.0	201,516	1.4
**SeqRun04**	2,027,794	1,935,149	56,077	2.8	1,806,655	88,307	4.6	40,187	2.1
**SeqRun05**	18,221,504	17,500,366	421,588	2.3	16,793,851	482,365	2.8	161,458	0.9
**SeqRun06**	20,718,202	19,874,913	429,048	2.1	19,317,305	371,048	1.9	81,422	0.4
**SeqRun07**	24,604,610	23,746,938	663,730	2.7	22,446,187	497,366	2.1	803,385	3.4
**Total**	124,271,981	120,150,332	2,822,635	2.3	115,070,784	2,928,340	2,5	1,983,378	1,7
**IndexRun**	10,276,093	10,116,808	NA	NA	5,841,190	4,186,688	41.4	88,930	0.9

[1] refers to total reads

[2] refers to matching tag 2

904 *Sequencing run SeqRun08 run contained libraries of another project, thus we were unable to provide a number of raw reads.

Table 5: Number of accepted species assignments with two different acceptance criteria the more stringent criterion accepting only assignments occurring in both *extraction replicates* (A & B), and the more lax criterion accepting assignment two or more positives in any of the twelve *PCR replicates*.

	**stringent**	**lax**	**change**
Aonyx cinereus	1	1	0
Arctictis binturong	1	1	0
Bos Javanicus	9	11	+2
Echinosorex gymnura	5	6	+1
Felis catus	2	2	0
Helarctos malayanus	5	6	+1
Hemigalus derbyanus	3	3	0
Hystrix brachyura	4	5	+1
Kalophrynus pleurostigma	1	1	0
Macaca fascicularis		1	+1
Macaca nemestrina	1	2	+1
Macaca sp.		3	+3
Manis javanicus	2	2	0
Muntiacus atherodes	6	6	0
Muntiacus muntjak	2	2	0
Muntiacus sp.	10	10	0
Mydaus javanensis		2	+2
Pongo pygmaeus	5	5	0
Rusa unicolor	59	67	+8
Sus barbatus	17	22	+5
Tragulus javanicus	4	6	+2
Tragulus napu	10	11	+1
Trichys fasciculata	5	5	0
Viverra tangalunga	11	11	0
**total accepted assignments**	162	190	+28

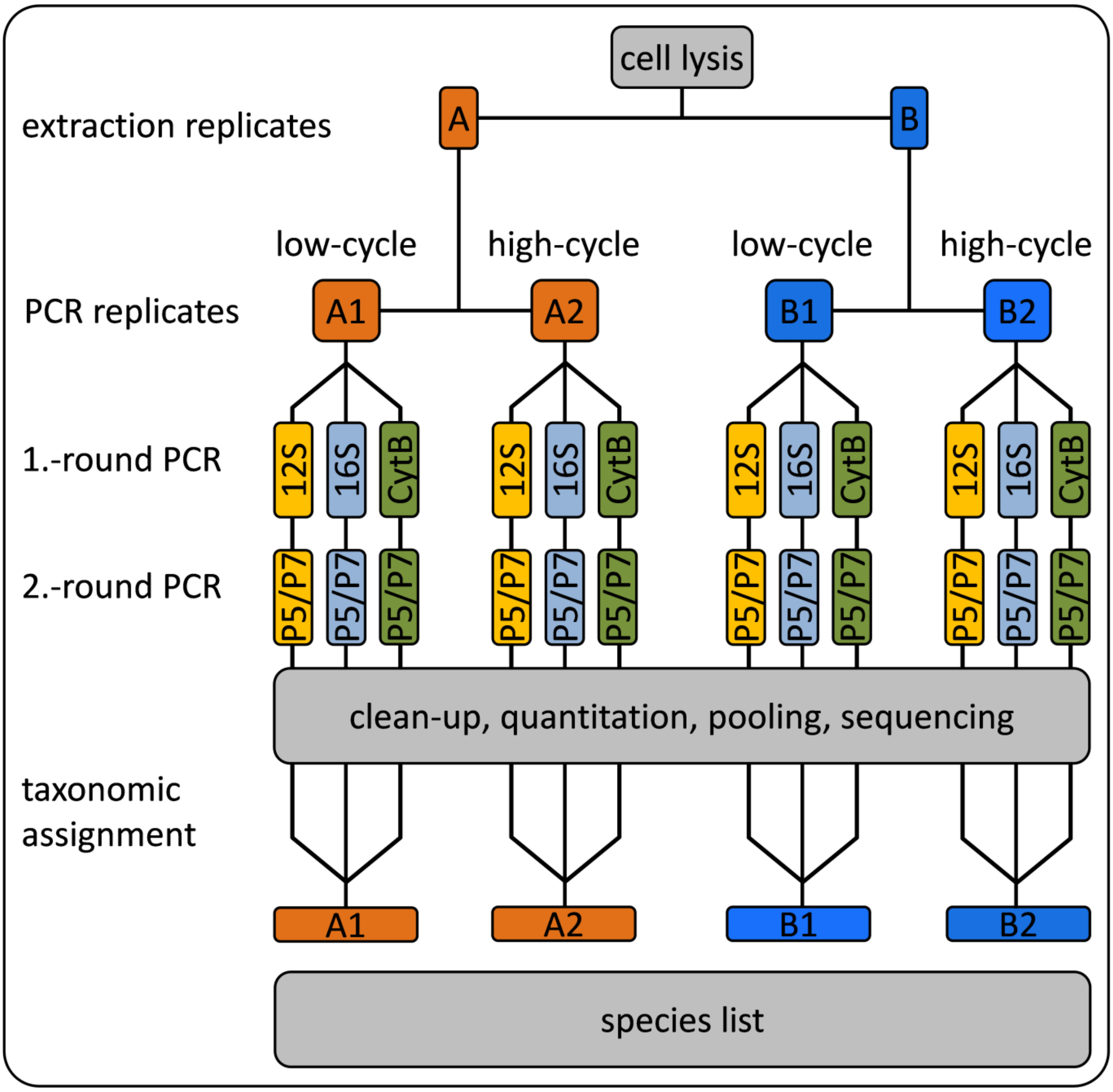

Figure 1: laboratory scheme; during DNA extraction the sample is split into two extraction replicates A & B. Our Protocol consists of two rounds of PCR that were the sample tags, the necessary sequencing primer and sequencing adapters are added to the the amplicons. For each extraction replicate we ran a low cycle PCR and a high cycle PCR for each marker that we have twelve independent PCR replicates per sample. All PCR products were sequenced and the obtained reads were taxonomically identified with PROTAX.

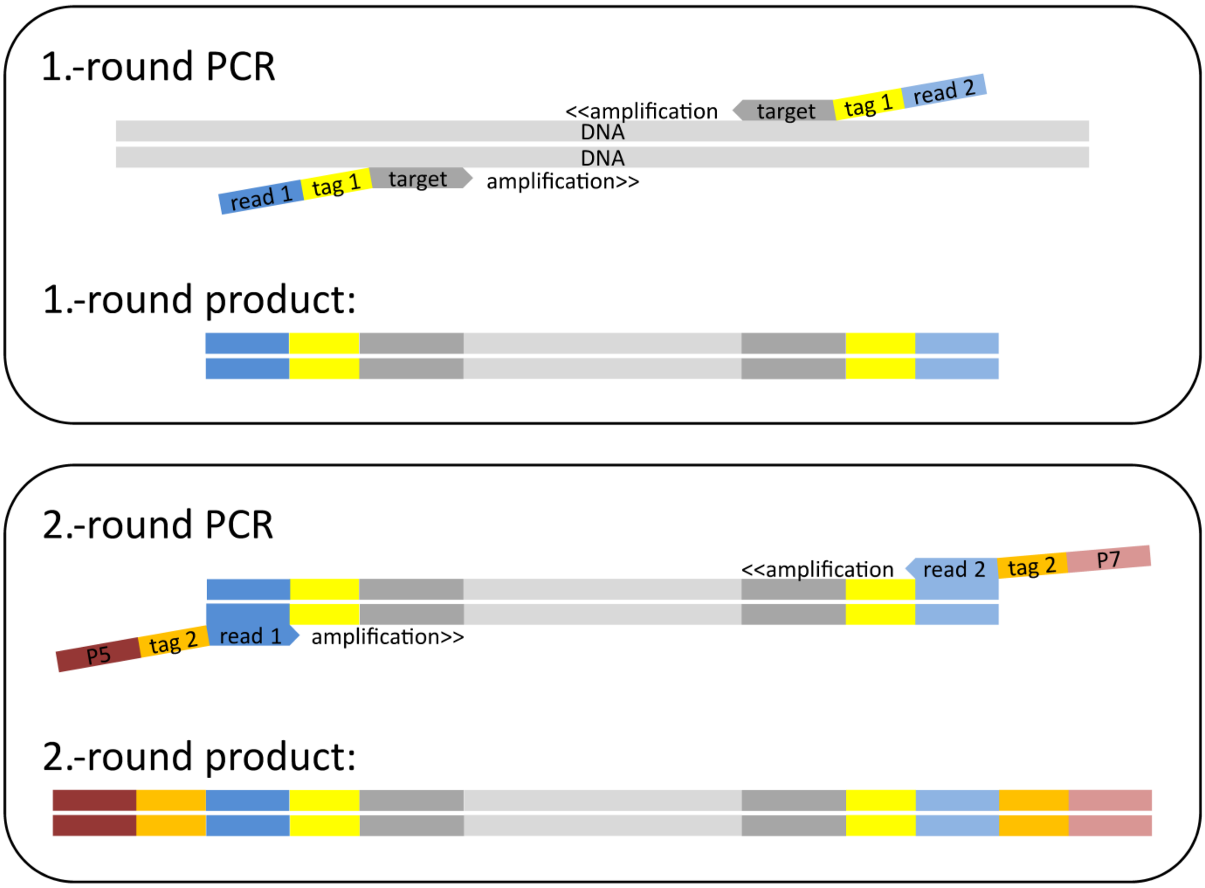

Figure 2: Scheme to build double 'twin-tagged' PCR libraries. The first round of PCR uses target-specific primers (*12S*, *16S*, or *CytB*, dark grey) that have both been extended with the same (i.e. 'twin') sample-identifying *tag* sequences *tag 1* (yellow) and then with the different *read 1* (dark blue) and *read 2* (light blue) sequence primers. The second round of PCR uses the priming sites of the *read 1* and *read 2* sequencing primers to add twin plate-identifying *tag* sequences *tag 2* (orange) and the P5 (dark red) and P7 (light red) Illumina adapters.

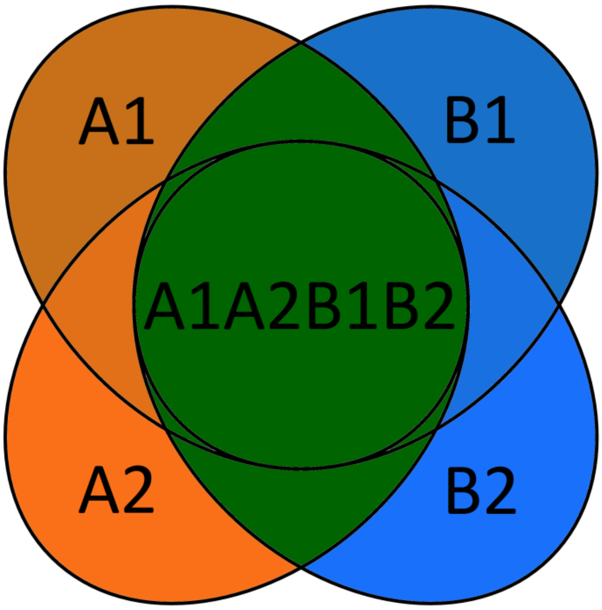

Figure 3: For the stringent acceptance criterion we only accepted taxonomic assignments that were positively detected in both *extraction replicates* A and B (green colour). The numbers (1 & 2) refer to the two PCR replicates for each extraction replicate.

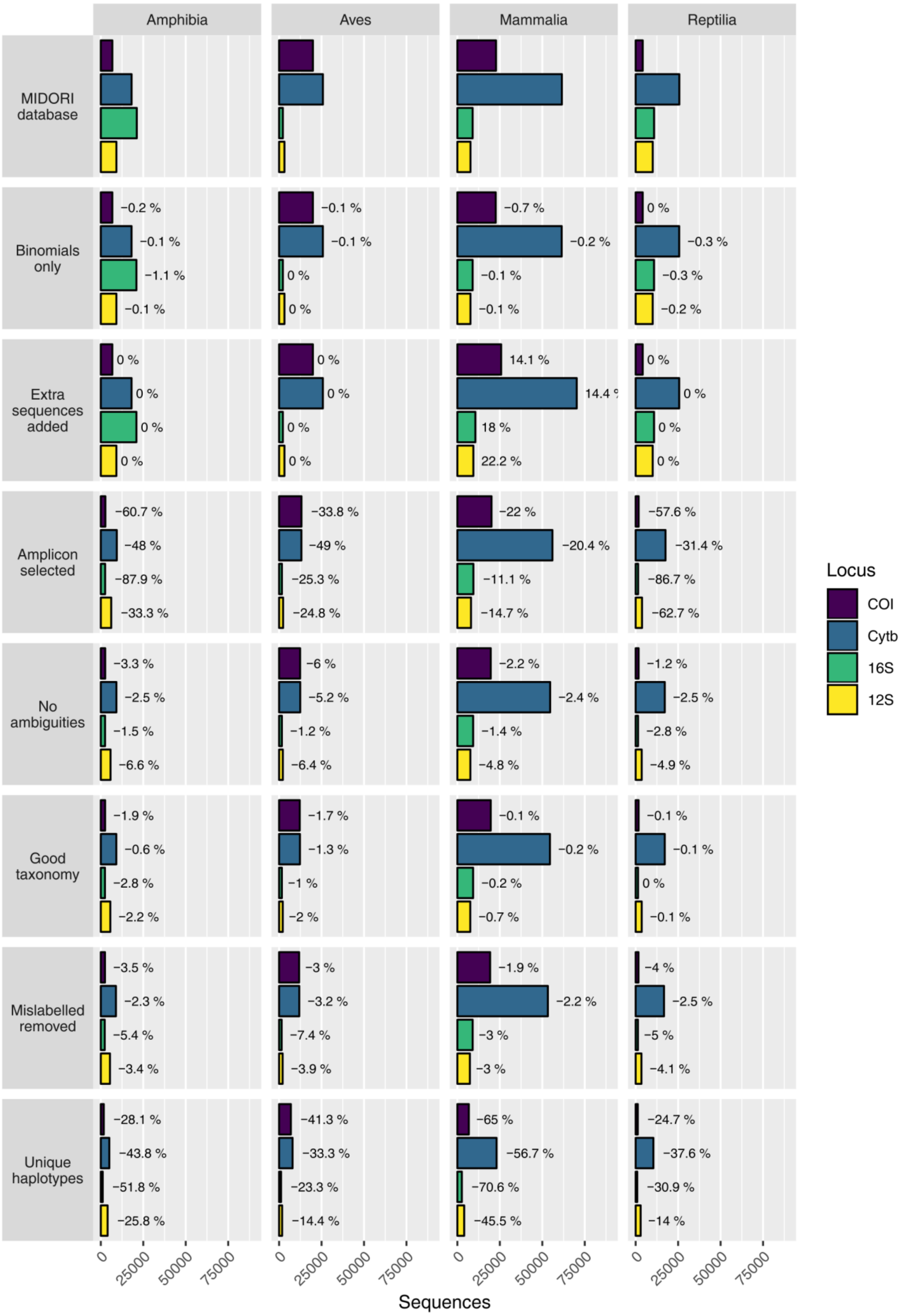

Figure 4: Data availability and percentage loss at each major step in the database curation procedure for each target amplicon and class of Tetrapoda. The number of sequences decreases between steps except "Extra sequences added" where additional target sequences are included for Mammalia and there is no change for the other three classes.

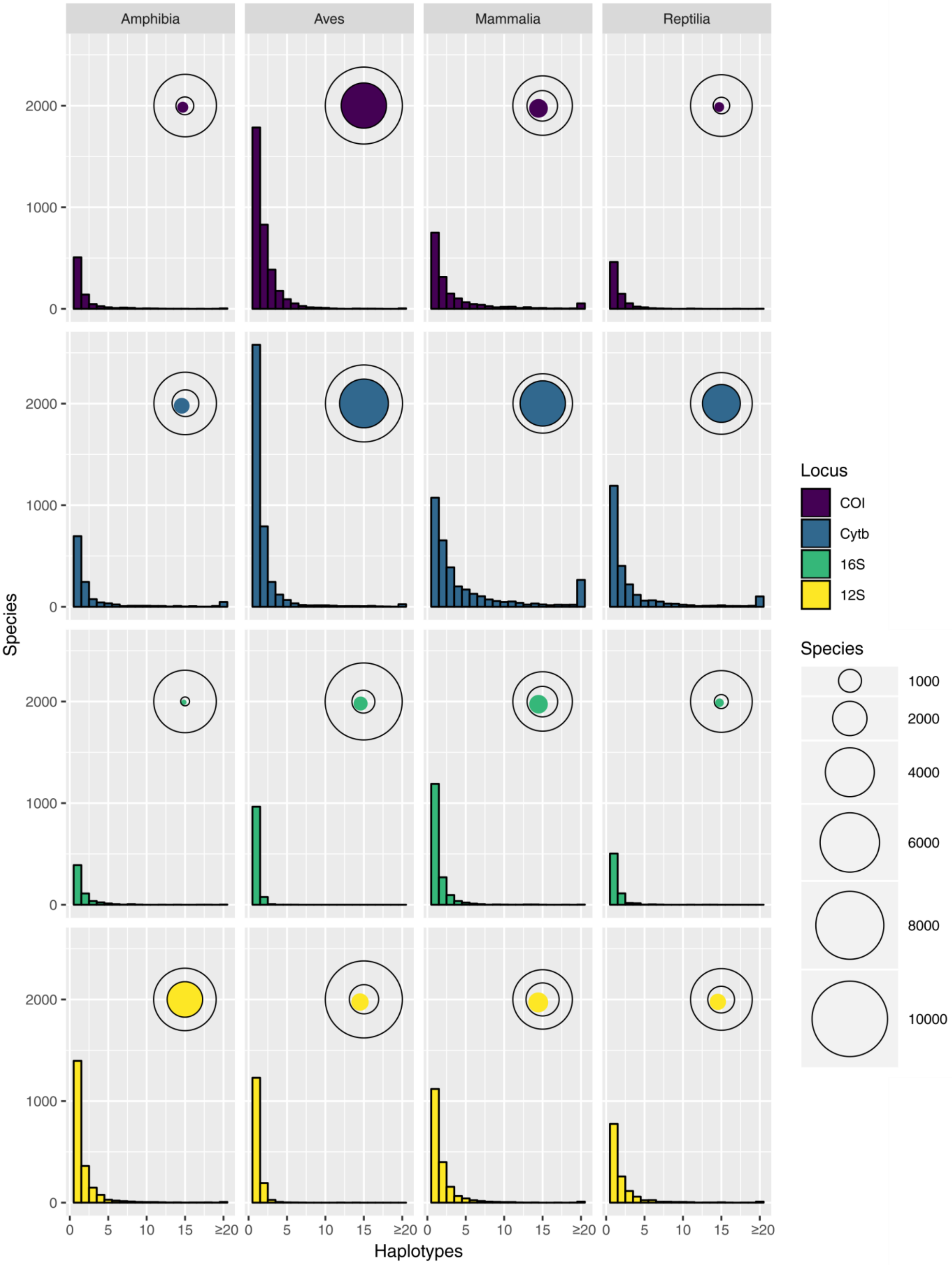

Figure 5: Haplotype number by species (frequency distribution) and the total number of species with at least one haplotype, shown relative to the total number of species in the taxonomy for that category (bubbles), shown for each marker and class of Tetrapoda. The proportion of species covered by the database varies between categories but in all cases a majority of recovered species are represented by a single unique haplotype.

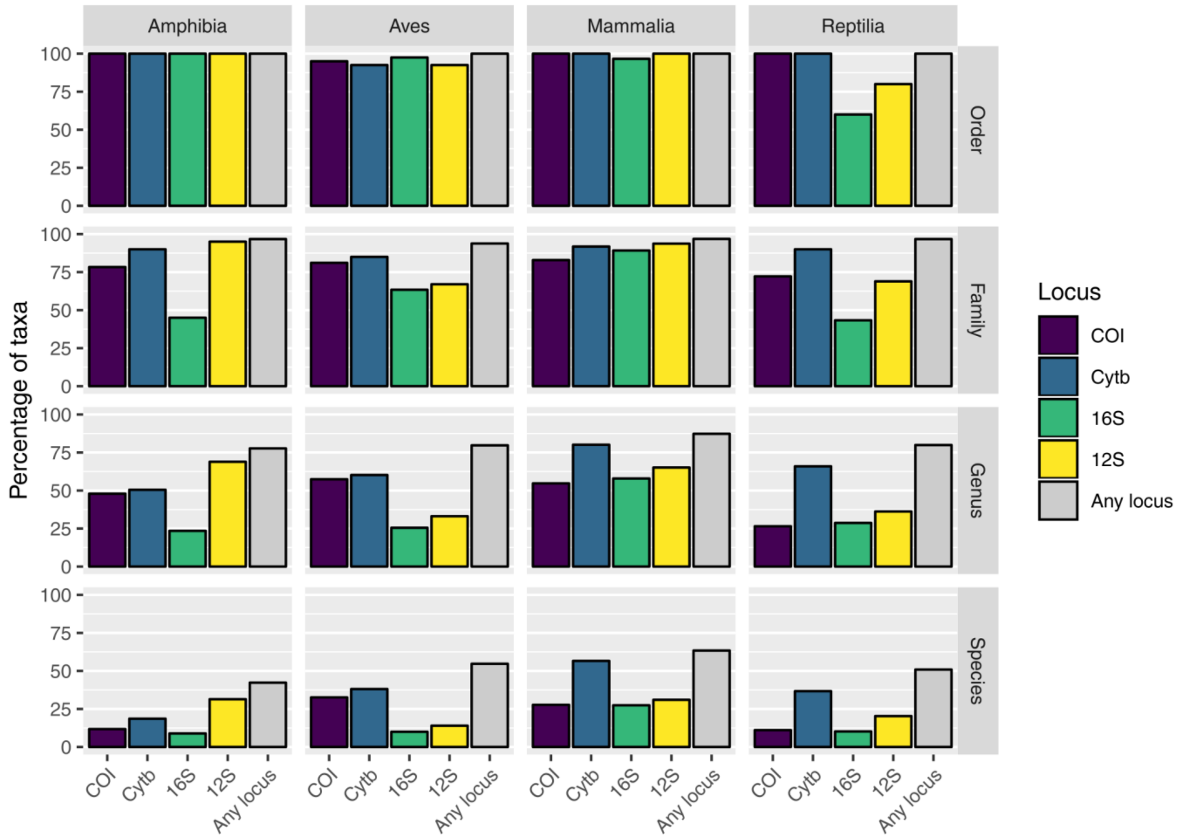

Figure 6: The percentage of the full taxonomy covered by the final database at each taxonomic level for each class of Tetrapoda. Includes the percentage of taxa represented by each marker and all markers combined. In all cases taking all four markers together increases the proportion of species, genera and families covered by the database, but it remains incomplete when compared with the full taxonomy.

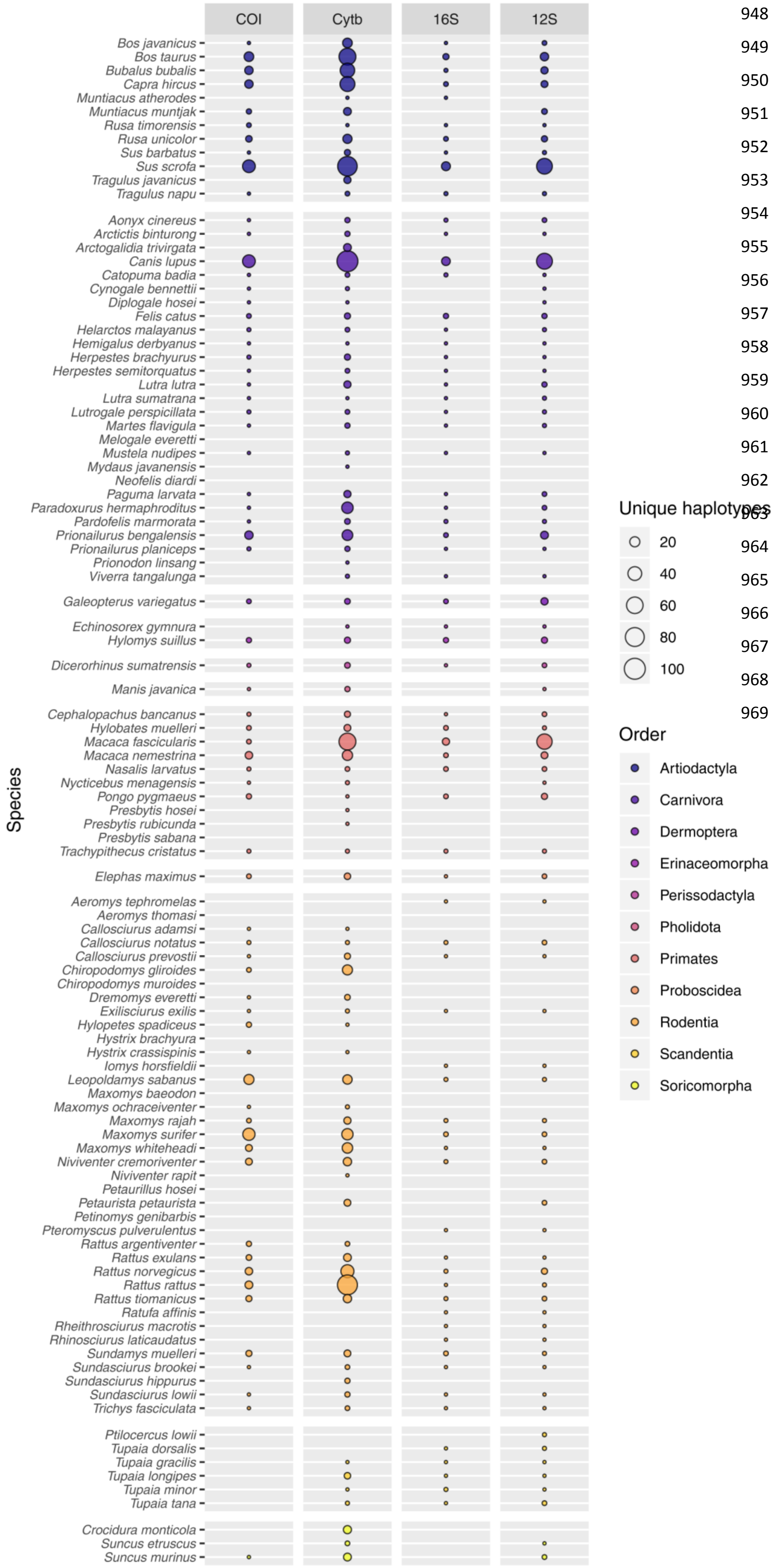

Figure 7: The number of unique haplotypes per marker for each of the 103 mammal species expected in the study area. Bubble size is proportional to the number of haplotypes and varies between 0 and 100. Only 55 species have at least one sequence per marker and nine species are completely unrepresented in the current database.

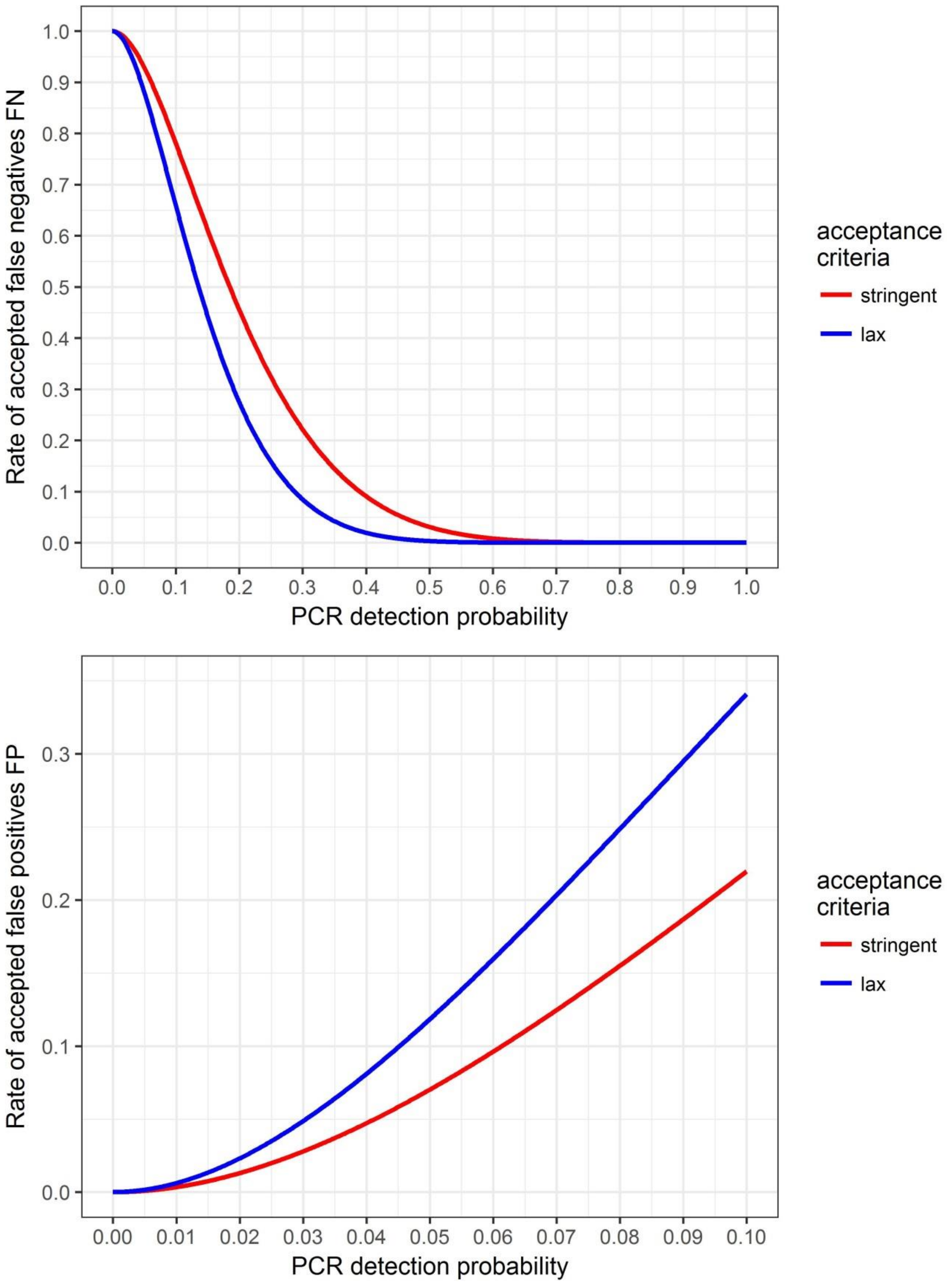

Supplemental Figure 1: The rates of accepted false negatives (upper graph) and false positives (lower graph) for both our used acceptance criteria for varying PCR detection probabilities. The red line always denotes the stringent acceptance criterion that a positive is only accepted if it is present in at least one A and one B replicate. The lax criterion (blue) accepted at any two positives out of the twelve replicates. The stringent criterion poses a higher risk of accepting a false negative but it reduces clearly the risk of false positives, especially with increasing detection probability due to higher risk of contamination.

Supplemental table 1: Complete list of all used primer sequences in 5'-3' direction.

primer name	primer sequence	direction	primer length [bp]
12SfA	ACACTCTTTCCCTACACGACGCTCTTCCGATCTTGCATAAAAAGCTTCAAACTGGGATTAGATACCCCACTAT	forward	73
12SfB	ACACTCTTTCCCTACACGACGCTCTTCCGATCTTCAGCAAAAAGCTTCAAACTGGGATTAGATACCCCACTAT	forward	73
12SfC	ACACTCTTTCCCTACACGACGCTCTTCCGATCTAAGCGAAAAAGCTTCAAACTGGGATTAGATACCCCACTAT	forward	73
12SfD	ACACTCTTTCCCTACACGACGCTCTTCCGATCTACAAGAAAAAGCTTCAAACTGGGATTAGATACCCCACTAT	forward	73
12SfE	ACACTCTTTCCCTACACGACGCTCTTCCGATCTAGTGGAAAAAGCTTCAAACTGGGATTAGATACCCCACTAT	forward	73
12SfF	ACACTCTTTCCCTACACGACGCTCTTCCGATCTTTGACAAAAAGCTTCAAACTGGGATTAGATACCCCACTAT	forward	73
12SfG	ACACTCTTTCCCTACACGACGCTCTTCCGATCTCCTATAAAAAGCTTCAAACTGGGATTAGATACCCCACTAT	forward	73
12SfH	ACACTCTTTCCCTACACGACGCTCTTCCGATCTGGATGAAAAAGCTTCAAACTGGGATTAGATACCCCACTAT	forward	73
12SfI	ACACTCTTTCCCTACACGACGCTCTTCCGATCTCTAGGAAAAAGCTTCAAACTGGGATTAGATACCCCACTAT	forward	73
12SfK	ACACTCTTTCCCTACACGACGCTCTTCCGATCTCACCTAAAAAGCTTCAAACTGGGATTAGATACCCCACTAT	forward	73
12SfL	ACACTCTTTCCCTACACGACGCTCTTCCGATCTGTCAAAAAAAGCTTCAAACTGGGATTAGATACCCCACTAT	forward	73
12SfM	ACACTCTTTCCCTACACGACGCTCTTCCGATCTGAAGTAAAAAGCTTCAAACTGGGATTAGATACCCCACTAT	forward	73
12SfN	ACACTCTTTCCCTACACGACGCTCTTCCGATCTCGGTTAAAAAGCTTCAAACTGGGATTAGATACCCCACTAT	forward	73
12SfO	ACACTCTTTCCCTACACGACGCTCTTCCGATCTACCGAAAAAAGCTTCAAACTGGGATTAGATACCCCACTAT	forward	73
12SfP	ACACTCTTTCCCTACACGACGCTCTTCCGATCTACGTCAAAAAGCTTCAAACTGGGATTAGATACCCCACTAT	forward	73
12SfQ	ACACTCTTTCCCTACACGACGCTCTTCCGATCTAGACTAAAAAGCTTCAAACTGGGATTAGATACCCCACTAT	forward	73
12SfR	ACACTCTTTCCCTACACGACGCTCTTCCGATCTAGGAAAAAAAGCTTCAAACTGGGATTAGATACCCCACTAT	forward	73
12SfS	ACACTCTTTCCCTACACGACGCTCTTCCGATCTATTCCAAAAAGCTTCAAACTGGGATTAGATACCCCACTAT	forward	73
12SfT	ACACTCTTTCCCTACACGACGCTCTTCCGATCTCAATCAAAAAGCTTCAAACTGGGATTAGATACCCCACTAT	forward	73
12SfW	ACACTCTTTCCCTACACGACGCTCTTCCGATCTCCACAAAAAAGCTTCAAACTGGGATTAGATACCCCACTAT	forward	73
12SfX	ACACTCTTTCCCTACACGACGCTCTTCCGATCTGCTTAAAAAAGCTTCAAACTGGGATTAGATACCCCACTAT	forward	73
12SfY	ACACTCTTTCCCTACACGACGCTCTTCCGATCTGGTACAAAAAGCTTCAAACTGGGATTAGATACCCCACTAT	forward	73

primer name	primer sequence	direction	primer length [bp]
12SfZ	ACACTCTTTCCCTACACGACGCTCTTCCGATCTAACACAAAAAGCTT CAAACTGGGATTAGATACCCCACTAT	forward	73
12Sfctr	ACACTCTTTCCCTACACGACGCTCTTCCGATCTATCTGAAAAAGCTT CAAACTGGGATTAGATACCCCACTAT	forward	73
12SrA	GTGACTGGAGTTCAGACGTGTGCTCTTCCGATCTTGCATTGACTGCA GAGGGTGACGGGCGGTGTGT	reverse	67
12SrB	GTGACTGGAGTTCAGACGTGTGCTCTTCCGATCTTCAGCTGACTGCA GAGGGTGACGGGCGGTGTGT	reverse	67
12SrC	GTGACTGGAGTTCAGACGTGTGCTCTTCCGATCTAAGCGTGACTGCA GAGGGTGACGGGCGGTGTGT	reverse	67
12SrD	GTGACTGGAGTTCAGACGTGTGCTCTTCCGATCTACAAGTGACTGCA GAGGGTGACGGGCGGTGTGT	reverse	67
12SrE	GTGACTGGAGTTCAGACGTGTGCTCTTCCGATCTAGTGGTGACTGCA GAGGGTGACGGGCGGTGTGT	reverse	67
12SrF	GTGACTGGAGTTCAGACGTGTGCTCTTCCGATCTTTGACTGACTGCA GAGGGTGACGGGCGGTGTGT	reverse	67
12SrG	GTGACTGGAGTTCAGACGTGTGCTCTTCCGATCTCCTATTGACTGCA GAGGGTGACGGGCGGTGTGT	reverse	67
12SrH	GTGACTGGAGTTCAGACGTGTGCTCTTCCGATCTGGATGTGACTGCA GAGGGTGACGGGCGGTGTGT	reverse	67
12SrI	GTGACTGGAGTTCAGACGTGTGCTCTTCCGATCTCTAGGTGACTGCA GAGGGTGACGGGCGGTGTGT	reverse	67
12SrK	GTGACTGGAGTTCAGACGTGTGCTCTTCCGATCTCACCTTGACTGCA GAGGGTGACGGGCGGTGTGT	reverse	67
12SrL	GTGACTGGAGTTCAGACGTGTGCTCTTCCGATCTGTCAATGACTGCA GAGGGTGACGGGCGGTGTGT	reverse	67
12SrM	GTGACTGGAGTTCAGACGTGTGCTCTTCCGATCTGAAGTTGACTGCA GAGGGTGACGGGCGGTGTGT	reverse	67
12SrN	GTGACTGGAGTTCAGACGTGTGCTCTTCCGATCTCGGTTTGACTGCA GAGGGTGACGGGCGGTGTGT	reverse	67
12SrO	GTGACTGGAGTTCAGACGTGTGCTCTTCCGATCTACCGATGACTGCA GAGGGTGACGGGCGGTGTGT	reverse	67
12SrP	GTGACTGGAGTTCAGACGTGTGCTCTTCCGATCTACGTCTGACTGCA GAGGGTGACGGGCGGTGTGT	reverse	67
12SrR	GTGACTGGAGTTCAGACGTGTGCTCTTCCGATCTAGGAATGACTGCA GAGGGTGACGGGCGGTGTGT	reverse	67
12SrS	GTGACTGGAGTTCAGACGTGTGCTCTTCCGATCTATTCCTGACTGCA GAGGGTGACGGGCGGTGTGT	reverse	67
12SrT	GTGACTGGAGTTCAGACGTGTGCTCTTCCGATCTCAATCTGACTGCA GAGGGTGACGGGCGGTGTGT	reverse	67
12SrV	GTGACTGGAGTTCAGACGTGTGCTCTTCCGATCTCATGATGACTGCA GAGGGTGACGGGCGGTGTGT	reverse	67
12SrW	GTGACTGGAGTTCAGACGTGTGCTCTTCCGATCTCCACATGACTGCA GAGGGTGACGGGCGGTGTGT	reverse	67

**primer name**	**primer sequence**	**direction**	**primer length [bp]**
12SrX	GTGACTGGAGTTCAGACGTGTGCTCTTCCGATCTGCTTATGACTGCA GAGGGTGACGGGCGGTGTGT	reverse	67
12SrY	GTGACTGGAGTTCAGACGTGTGCTCTTCCGATCTGGTACTGACTGCA GAGGGTGACGGGCGGTGTGT	reverse	67
12SrZ	GTGACTGGAGTTCAGACGTGTGCTCTTCCGATCTAACACTGACTGCA GAGGGTGACGGGCGGTGTGT	reverse	67
12Srctr	GTGACTGGAGTTCAGACGTGTGCTCTTCCGATCTATCTGTGACTGCA GAGGGTGACGGGCGGTGTGT	reverse	67
16SfA	ACACTCTTTCCCTACACGACGCTCTTCCGATCTTGCATCGGTTGGGG TGACCTCGGA	forward	57
16SfB	ACACTCTTTCCCTACACGACGCTCTTCCGATCTTCAGCCGGTTGGGG TGACCTCGGA	forward	57
16SfC	ACACTCTTTCCCTACACGACGCTCTTCCGATCTAAGCGCGGTTGGGG TGACCTCGGA	forward	57
16SfD	ACACTCTTTCCCTACACGACGCTCTTCCGATCTACAAGCGGTTGGGG TGACCTCGGA	forward	57
16SfE	ACACTCTTTCCCTACACGACGCTCTTCCGATCTAGTGGCGGTTGGGG TGACCTCGGA	forward	57
16SfF	ACACTCTTTCCCTACACGACGCTCTTCCGATCTTTGACCGGTTGGGG TGACCTCGGA	forward	57
16SfG	ACACTCTTTCCCTACACGACGCTCTTCCGATCTCCTATCGGTTGGGG TGACCTCGGA	forward	57
16SfH	ACACTCTTTCCCTACACGACGCTCTTCCGATCTGGATGCGGTTGGGG TGACCTCGGA	forward	57
16SfI	ACACTCTTTCCCTACACGACGCTCTTCCGATCTCTAGGCGGTTGGGG TGACCTCGGA	forward	57
16SfK	ACACTCTTTCCCTACACGACGCTCTTCCGATCTCACCTCGGTTGGGG TGACCTCGGA	forward	57
16SfL	ACACTCTTTCCCTACACGACGCTCTTCCGATCTGTCAACGGTTGGGG TGACCTCGGA	forward	57
16SfN	ACACTCTTTCCCTACACGACGCTCTTCCGATCTCGGTTCGGTTGGGG TGACCTCGGA	forward	57
16SfO	ACACTCTTTCCCTACACGACGCTCTTCCGATCTACCGACGGTTGGGG TGACCTCGGA	forward	57
16SfP	ACACTCTTTCCCTACACGACGCTCTTCCGATCTACGTCCGGTTGGGG TGACCTCGGA	forward	57
16SfQ	ACACTCTTTCCCTACACGACGCTCTTCCGATCTAGACTCGGTTGGGG TGACCTCGGA	forward	57
16SfR	ACACTCTTTCCCTACACGACGCTCTTCCGATCTAGGAACGGTTGGGG TGACCTCGGA	forward	57
16SfS	ACACTCTTTCCCTACACGACGCTCTTCCGATCTATTCCCGGTTGGGG TGACCTCGGA	forward	57
16SfT	ACACTCTTTCCCTACACGACGCTCTTCCGATCTCAATCCGGTTGGGG TGACCTCGGA	forward	57

primer name	primer sequence	direction	primer length [bp]
16SfV	ACACTCTTTCCCTACACGACGCTCTTCCGATCTCATGACGGTTGGGGTGACCTCGGA	forward	57
16SfW	ACACTCTTTCCCTACACGACGCTCTTCCGATCTCCACACGGTTGGGGTGACCTCGGA	forward	57
16SfX	ACACTCTTTCCCTACACGACGCTCTTCCGATCTGCTTACGGTTGGGGTGACCTCGGA	forward	57
16SfY	ACACTCTTTCCCTACACGACGCTCTTCCGATCTGGTACCGGTTGGGGTGACCTCGGA	forward	57
16SfZ	ACACTCTTTCCCTACACGACGCTCTTCCGATCTAACACCGGTTGGGGTGACCTCGGA	forward	57
16Sfcrt	ACACTCTTTCCCTACACGACGCTCTTCCGATCTATCTGCGGTTGGGGTGACCTCGGA	forward	57
16SrA	GTGACTGGAGTTCAGACGTGTGCTCTTCCGATCTTGCATGCTGTTATCCCTAGGGTAACT	reverse	60
16SrB	GTGACTGGAGTTCAGACGTGTGCTCTTCCGATCTTCAGCGCTGTTATCCCTAGGGTAACT	reverse	60
16SrC	GTGACTGGAGTTCAGACGTGTGCTCTTCCGATCTAAGCGGCTGTTATCCCTAGGGTAACT	reverse	60
16SrD	GTGACTGGAGTTCAGACGTGTGCTCTTCCGATCTACAAGGCTGTTATCCCTAGGGTAACT	reverse	60
16SrE	GTGACTGGAGTTCAGACGTGTGCTCTTCCGATCTAGTGGGCTGTTATCCCTAGGGTAACT	reverse	60
16SrF	GTGACTGGAGTTCAGACGTGTGCTCTTCCGATCTTTGACGCTGTTATCCCTAGGGTAACT	reverse	60
16SrG	GTGACTGGAGTTCAGACGTGTGCTCTTCCGATCTCCTATGCTGTTATCCCTAGGGTAACT	reverse	60
16SrI	GTGACTGGAGTTCAGACGTGTGCTCTTCCGATCTCTAGGGCTGTTATCCCTAGGGTAACT	reverse	60
16SrK	GTGACTGGAGTTCAGACGTGTGCTCTTCCGATCTCACCTGCTGTTATCCCTAGGGTAACT	reverse	60
16SrL	GTGACTGGAGTTCAGACGTGTGCTCTTCCGATCTGTCAAGCTGTTATCCCTAGGGTAACT	reverse	60
16SrM	GTGACTGGAGTTCAGACGTGTGCTCTTCCGATCTGAAGTGCTGTTATCCCTAGGGTAACT	reverse	60
16SrN	GTGACTGGAGTTCAGACGTGTGCTCTTCCGATCTCGGTTGCTGTTATCCCTAGGGTAACT	reverse	60
16SrO	GTGACTGGAGTTCAGACGTGTGCTCTTCCGATCTACCGAGCTGTTATCCCTAGGGTAACT	reverse	60
16SrP	GTGACTGGAGTTCAGACGTGTGCTCTTCCGATCTACGTCGCTGTTATCCCTAGGGTAACT	reverse	60
16SrQ	GTGACTGGAGTTCAGACGTGTGCTCTTCCGATCTAGACTGCTGTTATCCCTAGGGTAACT	reverse	60
16SrR	GTGACTGGAGTTCAGACGTGTGCTCTTCCGATCTAGGAAGCTGTTATCCCTAGGGTAACT	reverse	60

primer name	primer sequence	direction	primer length [bp]
16SrS	GTGACTGGAGTTCAGACGTGTGCTCTTCCGATCTATTCCGCTGTTAT CCCTAGGGTAACT	reverse	60
16SrT	GTGACTGGAGTTCAGACGTGTGCTCTTCCGATCTCAATCGCTGTTAT CCCTAGGGTAACT	reverse	60
16SrV	GTGACTGGAGTTCAGACGTGTGCTCTTCCGATCTCATGAGCTGTTAT CCCTAGGGTAACT	reverse	60
16SrW	GTGACTGGAGTTCAGACGTGTGCTCTTCCGATCTCCACAGCTGTTAT CCCTAGGGTAACT	reverse	60
16SrX	GTGACTGGAGTTCAGACGTGTGCTCTTCCGATCTGCTTAGCTGTTAT CCCTAGGGTAACT	reverse	60
16SrY	GTGACTGGAGTTCAGACGTGTGCTCTTCCGATCTGGTACGCTGTTAT CCCTAGGGTAACT	reverse	60
16SrZ	GTGACTGGAGTTCAGACGTGTGCTCTTCCGATCTAACACGCTGTTAT CCCTAGGGTAACT	reverse	60
16Srctr	GTGACTGGAGTTCAGACGTGTGCTCTTCCGATCTATCTGGCTGTTAT CCCTAGGGTAACT	reverse	60
CytBfA	ACACTCTTTCCCTACACGACGCTCTTCCGATCTTGCATAAAAAGCTT CCATCCAACATCTCAGCATGATGAAA	forward	73
CytBfB	ACACTCTTTCCCTACACGACGCTCTTCCGATCTTCAGCAAAAAGCTT CCATCCAACATCTCAGCATGATGAAA	forward	73
CytBfC	ACACTCTTTCCCTACACGACGCTCTTCCGATCTAAGCGAAAAAGCTT CCATCCAACATCTCAGCATGATGAAA	forward	73
CytBfE	ACACTCTTTCCCTACACGACGCTCTTCCGATCTAGTGGAAAAAGCTT CCATCCAACATCTCAGCATGATGAAA	forward	73
CytBfF	ACACTCTTTCCCTACACGACGCTCTTCCGATCTTTGACAAAAAGCTT CCATCCAACATCTCAGCATGATGAAA	forward	73
CytBfG	ACACTCTTTCCCTACACGACGCTCTTCCGATCTCCTATAAAAAGCTT CCATCCAACATCTCAGCATGATGAAA	forward	73
CytBfH	ACACTCTTTCCCTACACGACGCTCTTCCGATCTGGATGAAAAAGCTT CCATCCAACATCTCAGCATGATGAAA	forward	73
CytBfI	ACACTCTTTCCCTACACGACGCTCTTCCGATCTCTAGGAAAAAGCTT CCATCCAACATCTCAGCATGATGAAA	forward	73
CytBfK	ACACTCTTTCCCTACACGACGCTCTTCCGATCTCACCTAAAAAGCTT CCATCCAACATCTCAGCATGATGAAA	forward	73
CytBfL	ACACTCTTTCCCTACACGACGCTCTTCCGATCTGTCAAAAAAAGCTT CCATCCAACATCTCAGCATGATGAAA	forward	73
CytBfM	ACACTCTTTCCCTACACGACGCTCTTCCGATCTGAAGTAAAAAGCTT CCATCCAACATCTCAGCATGATGAAA	forward	73
CytBfN	ACACTCTTTCCCTACACGACGCTCTTCCGATCTCGGTTAAAAAGCTT CCATCCAACATCTCAGCATGATGAAA	forward	73
CytBfO	ACACTCTTTCCCTACACGACGCTCTTCCGATCTACCGAAAAAAGCTT CCATCCAACATCTCAGCATGATGAAA	forward	73
CytBfP	ACACTCTTTCCCTACACGACGCTCTTCCGATCTACGTCAAAAAGCTT CCATCCAACATCTCAGCATGATGAAA	forward	73

primer name	primer sequence	direction	primer length [bp]
CytBfQ	ACACTCTTTCCCTACACGACGCTCTTCCGATCTAGACTAAAAAGCTTCCATCCAACATCTCAGCATGATGAAA	forward	73
CytBfR	ACACTCTTTCCCTACACGACGCTCTTCCGATCTAGGAAAAAAAGCTTCCATCCAACATCTCAGCATGATGAAA	forward	73
CytBfS	ACACTCTTTCCCTACACGACGCTCTTCCGATCTATTCCAAAAAGCTTCCATCCAACATCTCAGCATGATGAAA	forward	73
CytBfT	ACACTCTTTCCCTACACGACGCTCTTCCGATCTCAATCAAAAAGCTTCCATCCAACATCTCAGCATGATGAAA	forward	73
CytBfV	ACACTCTTTCCCTACACGACGCTCTTCCGATCTCATGAAAAAAGCTTCCATCCAACATCTCAGCATGATGAAA	forward	73
CytBfW	ACACTCTTTCCCTACACGACGCTCTTCCGATCTCCACAAAAAAGCTTCCATCCAACATCTCAGCATGATGAAA	forward	73
CytBfX	ACACTCTTTCCCTACACGACGCTCTTCCGATCTGCTTAAAAAAGCTTCCATCCAACATCTCAGCATGATGAAA	forward	73
CytBfY	ACACTCTTTCCCTACACGACGCTCTTCCGATCTGGTACAAAAAGCTTCCATCCAACATCTCAGCATGATGAAA	forward	73
CytBfZ	ACACTCTTTCCCTACACGACGCTCTTCCGATCTAACACAAAAAGCTTCCATCCAACATCTCAGCATGATGAAA	forward	73
CytBrA	GTGACTGGAGTTCAGACGTGTGCTCTTCCGATCTTGCATAAACTGCAGCCCCTCAGAATGATATTTGTCCTCA	reverse	73
CytBrB	GTGACTGGAGTTCAGACGTGTGCTCTTCCGATCTTCAGCAAACTGCAGCCCCTCAGAATGATATTTGTCCTCA	reverse	73
CytBrC	GTGACTGGAGTTCAGACGTGTGCTCTTCCGATCTAAGCGAAACTGCAGCCCCTCAGAATGATATTTGTCCTCA	reverse	73
CytBrD	GTGACTGGAGTTCAGACGTGTGCTCTTCCGATCTACAAGAAACTGCAGCCCCTCAGAATGATATTTGTCCTCA	reverse	73
CytBrE	GTGACTGGAGTTCAGACGTGTGCTCTTCCGATCTAGTGGAAACTGCAGCCCCTCAGAATGATATTTGTCCTCA	reverse	73
CytBrF	GTGACTGGAGTTCAGACGTGTGCTCTTCCGATCTTTGACAAACTGCAGCCCCTCAGAATGATATTTGTCCTCA	reverse	73
CytBrG	GTGACTGGAGTTCAGACGTGTGCTCTTCCGATCTCCTATAAACTGCAGCCCCTCAGAATGATATTTGTCCTCA	reverse	73
CytBrH	GTGACTGGAGTTCAGACGTGTGCTCTTCCGATCTGGATGAAACTGCAGCCCCTCAGAATGATATTTGTCCTCA	reverse	73
CytBrI	GTGACTGGAGTTCAGACGTGTGCTCTTCCGATCTCTAGGAAACTGCAGCCCCTCAGAATGATATTTGTCCTCA	reverse	73
CytBrK	GTGACTGGAGTTCAGACGTGTGCTCTTCCGATCTCACCTAAACTGCAGCCCCTCAGAATGATATTTGTCCTCA	reverse	73
CytBrL	GTGACTGGAGTTCAGACGTGTGCTCTTCCGATCTGTCAAAAACTGCAGCCCCTCAGAATGATATTTGTCCTCA	reverse	73
CytBrM	GTGACTGGAGTTCAGACGTGTGCTCTTCCGATCTGAAGTAAACTGCAGCCCCTCAGAATGATATTTGTCCTCA	reverse	73
CytBrN	GTGACTGGAGTTCAGACGTGTGCTCTTCCGATCTCGGTTAAACTGCAGCCCCTCAGAATGATATTTGTCCTCA	reverse	73

primer name	primer sequence	direction	primer length [bp]
CytBrO	GTGACTGGAGTTCAGACGTGTGCTCTTCCGATCTACCGAAAACTGCAGCCCCTCAGAATGATATTTGTCCTCA	reverse	73
CytBrP	GTGACTGGAGTTCAGACGTGTGCTCTTCCGATCTACGTCAAACTGCAGCCCCTCAGAATGATATTTGTCCTCA	reverse	73
CytBrQ	GTGACTGGAGTTCAGACGTGTGCTCTTCCGATCTAGACTAAACTGCAGCCCCTCAGAATGATATTTGTCCTCA	reverse	73
CytBrR	GTGACTGGAGTTCAGACGTGTGCTCTTCCGATCTAGGAAAAACTGCAGCCCCTCAGAATGATATTTGTCCTCA	reverse	73
CytBrS	GTGACTGGAGTTCAGACGTGTGCTCTTCCGATCTATTCCAAACTGCAGCCCCTCAGAATGATATTTGTCCTCA	reverse	73
CytBrT	GTGACTGGAGTTCAGACGTGTGCTCTTCCGATCTCAATCAAACTGCAGCCCCTCAGAATGATATTTGTCCTCA	reverse	73
CytBrV	GTGACTGGAGTTCAGACGTGTGCTCTTCCGATCTCATGAAAACTGCAGCCCCTCAGAATGATATTTGTCCTCA	reverse	73
CytBrX	GTGACTGGAGTTCAGACGTGTGCTCTTCCGATCTGCTTAAAACTGCAGCCCCTCAGAATGATATTTGTCCTCA	reverse	73
CytBrY	GTGACTGGAGTTCAGACGTGTGCTCTTCCGATCTGGTACAAACTGCAGCCCCTCAGAATGATATTTGTCCTCA	reverse	73
CytBrZ	GTGACTGGAGTTCAGACGTGTGCTCTTCCGATCTAACACAAACTGCAGCCCCTCAGAATGATATTTGTCCTCA	reverse	73
CytBrctr	GTGACTGGAGTTCAGACGTGTGCTCTTCCGATCTATCTGAAACTGCAGCCCCTCAGAATGATATTTGTCCTCA	reverse	73
P5-A	AATGATACGGCGACCACCGAGATCTACACTGCATACACTCTTTCCCTACACGACGCTCTTCCGATCT	forward	67
P5-B	AATGATACGGCGACCACCGAGATCTACACTCAGCACACTCTTTCCCTACACGACGCTCTTCCGATCT	forward	67
P5-C	AATGATACGGCGACCACCGAGATCTACACAAGCGACACTCTTTCCCTACACGACGCTCTTCCGATCT	forward	67
P5-D	AATGATACGGCGACCACCGAGATCTACACACAAGACACTCTTTCCCTACACGACGCTCTTCCGATCT	forward	67
P5-E	AATGATACGGCGACCACCGAGATCTACACAGTGGACACTCTTTCCCTACACGACGCTCTTCCGATCT	forward	67
P5-F	AATGATACGGCGACCACCGAGATCTACACTTGACACACTCTTTCCCTACACGACGCTCTTCCGATCT	forward	67
P5-G	AATGATACGGCGACCACCGAGATCTACACCCTATACACTCTTTCCCTACACGACGCTCTTCCGATCT	forward	67
P5-H	AATGATACGGCGACCACCGAGATCTACACGGATGACACTCTTTCCCTACACGACGCTCTTCCGATCT	forward	67
P5-I	AATGATACGGCGACCACCGAGATCTACACCTAGGACACTCTTTCCCTACACGACGCTCTTCCGATCT	forward	67
P5-K	AATGATACGGCGACCACCGAGATCTACACCACCTACACTCTTTCCCTACACGACGCTCTTCCGATCT	forward	67
P5-L	AATGATACGGCGACCACCGAGATCTACACGTCAAACACTCTTTCCCTACACGACGCTCTTCCGATCT	forward	67

primer name	primer sequence	direction	primer length [bp]
P5-M	AATGATACGGCGACCACCGAGATCTACACGAAGTACACTCTTTCCCTACACGACGCTCTTCCGATCT	forward	67
P5-N	AATGATACGGCGACCACCGAGATCTACACCGGTTACACTCTTTCCCTACACGACGCTCTTCCGATCT	forward	67
P5-O	AATGATACGGCGACCACCGAGATCTACACACCGAACACTCTTTCCCTACACGACGCTCTTCCGATCT	forward	67
P5-P	AATGATACGGCGACCACCGAGATCTACACACGTCACACTCTTTCCCTACACGACGCTCTTCCGATCT	forward	67
P5-Q	AATGATACGGCGACCACCGAGATCTACACAGACTACACTCTTTCCCTACACGACGCTCTTCCGATCT	forward	67
P5-S	AATGATACGGCGACCACCGAGATCTACACATTCCACACTCTTTCCCTACACGACGCTCTTCCGATCT	forward	67
P5-T	AATGATACGGCGACCACCGAGATCTACACCAATCACACTCTTTCCCTACACGACGCTCTTCCGATCT	forward	67
P5-V	AATGATACGGCGACCACCGAGATCTACACCATGAACACTCTTTCCCTACACGACGCTCTTCCGATCT	forward	67
P7-A	CAAGCAGAAGACGGCATACGAGATTGCATGTGACTGGAGTTCAGACGTGTGCTCTTCCGATCT	reverse	65
P7-B	CAAGCAGAAGACGGCATACGAGATTCAGCGTGACTGGAGTTCAGACGTGTGCTCTTCCGATCT	reverse	63
P7-C	CAAGCAGAAGACGGCATACGAGATAAGCGGTGACTGGAGTTCAGACGTGTGCTCTTCCGATCT	reverse	63
P7-D	CAAGCAGAAGACGGCATACGAGATACAAGGTGACTGGAGTTCAGACGTGTGCTCTTCCGATCT	reverse	63
P7-E	CAAGCAGAAGACGGCATACGAGATAGTGGGTGACTGGAGTTCAGACGTGTGCTCTTCCGATCT	reverse	63
P7-F	CAAGCAGAAGACGGCATACGAGATTTGACGTGACTGGAGTTCAGACGTGTGCTCTTCCGATCT	reverse	63
P7-G	CAAGCAGAAGACGGCATACGAGATCCTATGTGACTGGAGTTCAGACGTGTGCTCTTCCGATCT	reverse	63
P7-H	CAAGCAGAAGACGGCATACGAGATGGATGGTGACTGGAGTTCAGACGTGTGCTCTTCCGATCT	reverse	63
P7-I	CAAGCAGAAGACGGCATACGAGATCTAGGGTGACTGGAGTTCAGACGTGTGCTCTTCCGATCT	reverse	63
P7-K	CAAGCAGAAGACGGCATACGAGATCACCTGTGACTGGAGTTCAGACGTGTGCTCTTCCGATCT	reverse	63
P7-L	CAAGCAGAAGACGGCATACGAGATGTCAAGTGACTGGAGTTCAGACGTGTGCTCTTCCGATCT	reverse	63
P7-M	CAAGCAGAAGACGGCATACGAGATGAAGTGTGACTGGAGTTCAGACGTGTGCTCTTCCGATCT	reverse	63
P7-N	CAAGCAGAAGACGGCATACGAGATCGGTTGTGACTGGAGTTCAGACGTGTGCTCTTCCGATCT	reverse	63
P7-O	CAAGCAGAAGACGGCATACGAGATACCGAGTGACTGGAGTTCAGACGTGTGCTCTTCCGATCT	reverse	63

primer name	primer sequence	direction	primer length [bp]
P7-P	CAAGCAGAAGACGGCATACGAGATACGTCGTGACTGGAGTTCAGACG TGTGCTCTTCCGATCT	reverse	63
P7-Q	CAAGCAGAAGACGGCATACGAGATAGACTGTGACTGGAGTTCAGACG TGTGCTCTTCCGATCT	reverse	63
P7-R	CAAGCAGAAGACGGCATACGAGATAGGAAGTGACTGGAGTTCAGACG TGTGCTCTTCCGATCT	reverse	63
P7-T	CAAGCAGAAGACGGCATACGAGATCAATCGTGACTGGAGTTCAGACG TGTGCTCTTCCGATCT	reverse	63
P7-V	CAAGCAGAAGACGGCATACGAGATCATGAGTGACTGGAGTTCAGACG TGTGCTCTTCCGATCT	reverse	63
P7-W	CAAGCAGAAGACGGCATACGAGATCCACAGTGACTGGAGTTCAGACG TGTGCTCTTCCGATCT	reverse	63
P7-X	CAAGCAGAAGACGGCATACGAGATGCTTAGTGACTGGAGTTCAGACG TGTGCTCTTCCGATCT	reverse	63
P7-Y	CAAGCAGAAGACGGCATACGAGATGGTACGTGACTGGAGTTCAGACG TGTGCTCTTCCGATCT	reverse	63
P7-Z	CAAGCAGAAGACGGCATACGAGATAACACGTGACTGGAGTTCAGACG TGTGCTCTTCCGATCT	reverse	6

Supplemental table 2: List of Bornean species that were weighted in the PROTAX assignment.

**Species**	**Species**	**Species**
Bos,javanicus	*Arctictis,binturong*	*Chiropodomys,muroides*
Bos,taurus	*Arctogalidia,trivirgata*	*Leopoldamys,sabanus*
Bubalus,bubalis	*Cynogale,bennettii*	*Maxomys,baeodon*
Capra,hircus	*Diplogale,hosei*	*Maxomys,ochraceiventer*
Muntiacus,atherodes	*Hemigalus,derbyanus*	*Maxomys,rajah*
Muntiacus,muntjak	*Paguma,larvata*	*Maxomys,surifer*
Rusa,timorensis	*Paradoxurus,hermaphroditus*	*Maxomys,whiteheadi*
Rusa,unicolor	*Prionodon,linsang*	*Niviventer,cremoriventer*
Sus,barbatus	*Viverra,tangalunga*	*Niviventer,rapit*
Sus,scrofa	*Galeopterus,variegatus*	*Rattus,argentiventer*
Tragulus,javanicus	*Echinosorex,gymnura*	*Rattus,exulans*
Tragulus,napu	*Hylomys,suillus*	*Rattus,norvegicus*
Canis,lupus	*Dicerorhinus,sumatrensis*	*Rattus,rattus*
Catopuma,badia	*Manis,javanica*	*Rattus,tiomanicus*
Felis,catus	*Macaca,fascicularis*	*Sundamys,muelleri*
Neofelis,diardi	*Macaca,nemestrina*	*Aeromys,tephromelas*
Pardofelis,marmorata	*Nasalis,larvatus*	*Aeromys,thomasi*
Prionailurus,bengalensis	*Presbytis,hosei*	*Callosciurus,adamsi*
Prionailurus,planiceps	*Presbytis,rubicunda*	*Callosciurus,notatus*
Herpestes,brachyurus	*Presbytis,sabana*	*Callosciurus,prevostii*
Herpestes,semitorquatus	*Trachypithecus,cristatus*	*Dremomys,everetti*
Mydaus,javanensis	*Pongo,pygmaeus*	*Exilisciurus,exilis*
Aonyx,cinereus	*Hylobates,muelleri*	*Hylopetes,spadiceus*
Lutra,lutra	*Nycticebus,menagensis*	*Iomys,horsfieldii*
Lutra,sumatrana	*Cephalopachus,bancanus*	*Petaurillus,hosei*
Lutrogale,perspicillata	*Elephas,maximus*	*Petaurista,petaurista*
Martes,flavigula	*Hystrix,brachyura*	*Petinomys,genibarbis*
Melogale,everetti	*Hystrix,crassispinis*	*Pteromyscus,pulverulentus*
Mustela,nudipes	*Trichys,fasciculata*	*Ratufa,affinis*
Helarctos,malayanus	*Chiropodomys,gliroides*	*Rheithrosciurus,macrotis*
Rhinosciurus,laticaudatus	*Tupaia,dorsalis*	*Crocidura,monticola*
Sundasciurus,brookei	*Tupaia,gracilis*	*Suncus,etruscus*

**Species**	**Species**	**Species**
Sundasciurus,hippurus	*Tupaia,longipes*	*Suncus,murinus*
Sundasciurus,lowii	*Tupaia,minor*	
Ptilocercus,lowii	*Tupaia,tana*	

Supplemental table 3: Summary of the read losses of each sample during the read processing steps for each sequencing run seperately. The first line gives the raw read number per sample. The losses are given as percentage of each step; 1. merging of the R1/R2 reads of the Illumina sequencing done by *usearch* [43; 44], 2. clipping of primers and trimming of reads using *cutadapt* [45], 3. quality filtering and 4. dereplication, both using usearch.

	Step	Mean	SD	Median	Min	Max
**SeqRun01**	raw	72977	96466	74	1	422271
	merging	7%	11%	2%	1%	50%
	clipping & trimming	2%	14%	0%	0%	100%
	filtering	4%	11%	2%	1%	100%
**SeqRun02**	raw	97372	83870	117626	1	409999
	merging	22%	23%	13%	2%	98%
	clipping & trimming	2%	13%	0%	0%	100%
	filtering	6%	3%	6%	5%	43%
**SeqRun03**	raw	57359	123971	48	1	1105978
	merging	5%	3%	5%	1%	11%
	clipping & trimming	43%	40%	28%	0%	100%
	filtering	37%	20%	29%	24%	100%
**SeqRun04**	raw	8629	10184	2075	1	37592
	merging	8%	2%	8%	6%	14%
	clipping & trimming	79%	34%	100%	0%	100%
	filtering	38%	18%	34%	0%	92%
**SeqRun05**	raw	77936	193818	36	1	1081947
	merging	34%	17%	36%	4%	89%
	clipping & trimming	50%	41%	59%	0%	100%
	filtering	53%	19%	51%	0%	100%
**SeqRun06**	raw	80816	80656	87013	1	407872
	merging	10%	15%	3%	1%	69%
	clipping & trimming	0%	0%	0%	0%	1%
	filtering	5%	1%	4%	4%	7%
**SeqRun07**	raw	90040	91022	81026	1	383072
	merging	23%	25%	10%	2%	99%
	clipping & trimming	1%	8%	0%	0%	100%
	filtering	6%	1%	6%	4%	10%
**SeqRun08**	raw	52951	132500	64	1	993255
	merging	14%	8%	17%	1%	26%
	clipping & trimming	89%	24%	100%	1%	100%
	filtering	49%	37%	28%	0%	100%

Supplemental table 4: Number of merged R1/R2 reads per sample that were used for the taxonomic assignment for each of the eight sequencing runs. Displayed are the median, minimum, maximum read numbers per PCR replicate, the mean and its standard deviation as well as the number of PCR replicates with less than 500 reads.

		SeqRun01	SeqRun02	SeqRun03	SeqRun04	SeqRun05	SeqRun06	SeqRun07	SeqRun08
median	16S	172,566	122,890				132,313	138,584	
min	16S	15	106				14,343	422	
max	16S	408,924	293,765				385,649	309,591	
mean	16S	162,487	110,274				126,365	120,850	
sd	16S	65,214	62,835				54,000	68,996	
< 500	16S	1	1				0	1	
median	12S			46,597	9,628	9,383			52,260
min	12S			2	3	3			1,164
max	12S			380,936	19,961	19,621			516,686
mean	12S			64,377	8,747	8,551			70,999
sd	12S			66,703	4,824	4,736			97,161
< 500	12S			9	62	62			49
median	CytB				8,428	8,218			53,104
min	CytB				3	3			2
max	CytB				19,961	19,621			608,948
mean	CytB				7,815	7,638			79,434
sd	CytB				5,473	5,365			120,055
< 500	CytB				21	21			13

Figure

Click here to access/download;Figure;Fig1.png

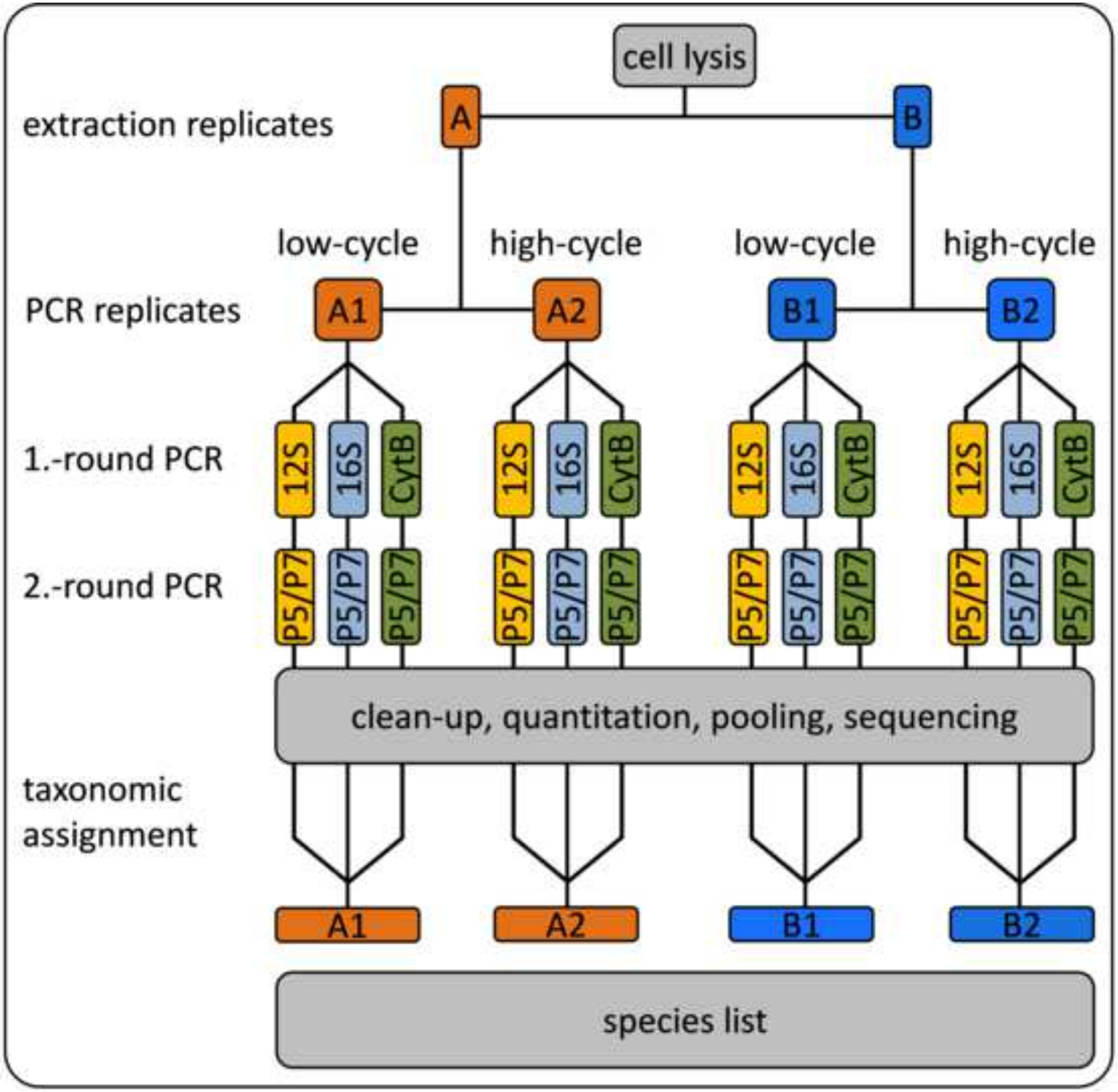

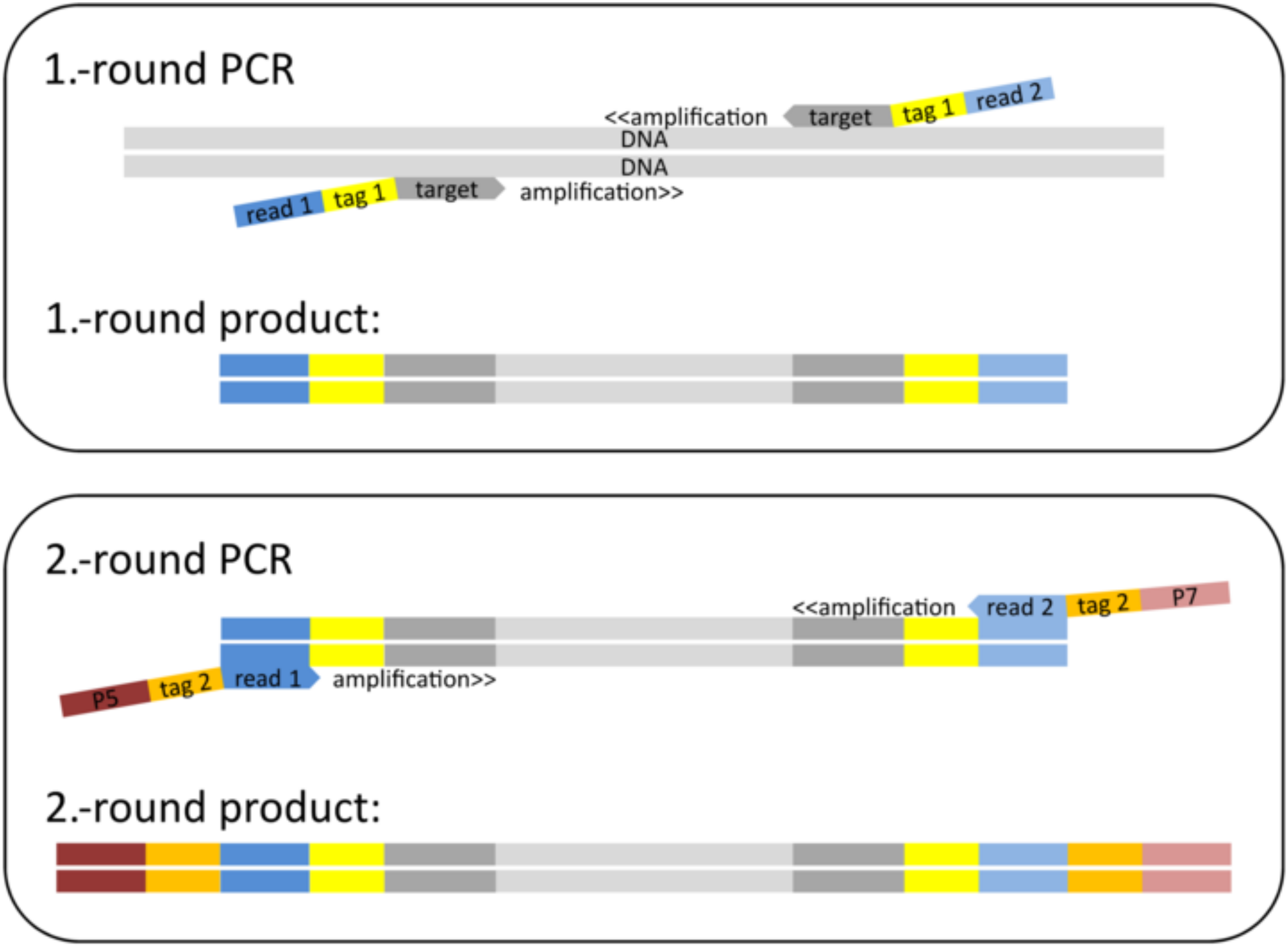
1.-round PCR
<<amplification
target
tag 1
read 2
DNA
DNA
read 1
tag 1
target
amplification>>
1.-round product:
2.-round PCR
<<amplification
read 2
tag 2
P7
P5
tag 2
read 1
amplification>>
2.-round product:

 Click here to access/download;Figure;Fig3.png

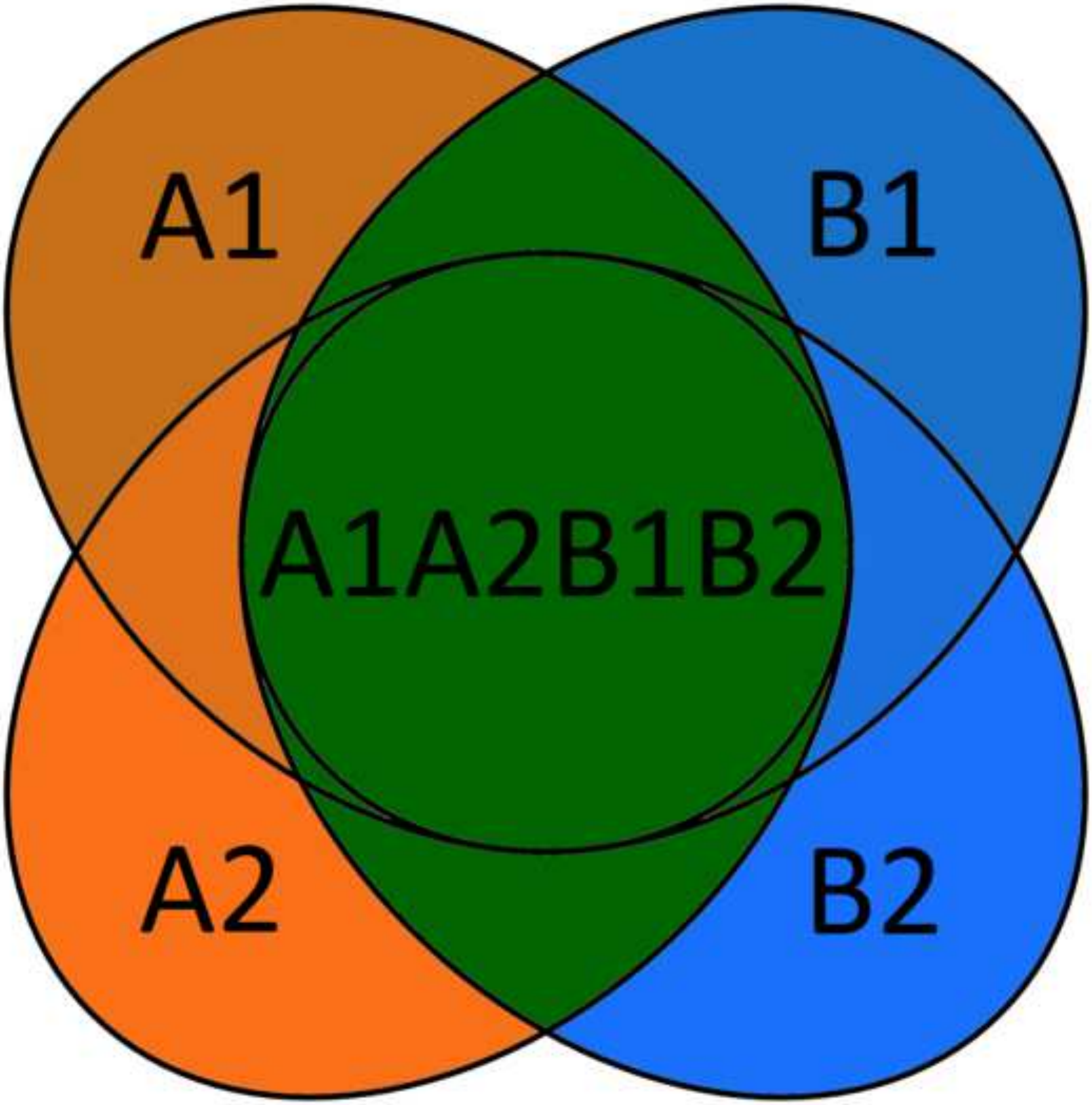

Figure

Click here to access/download;Figure;Fig4.pdf

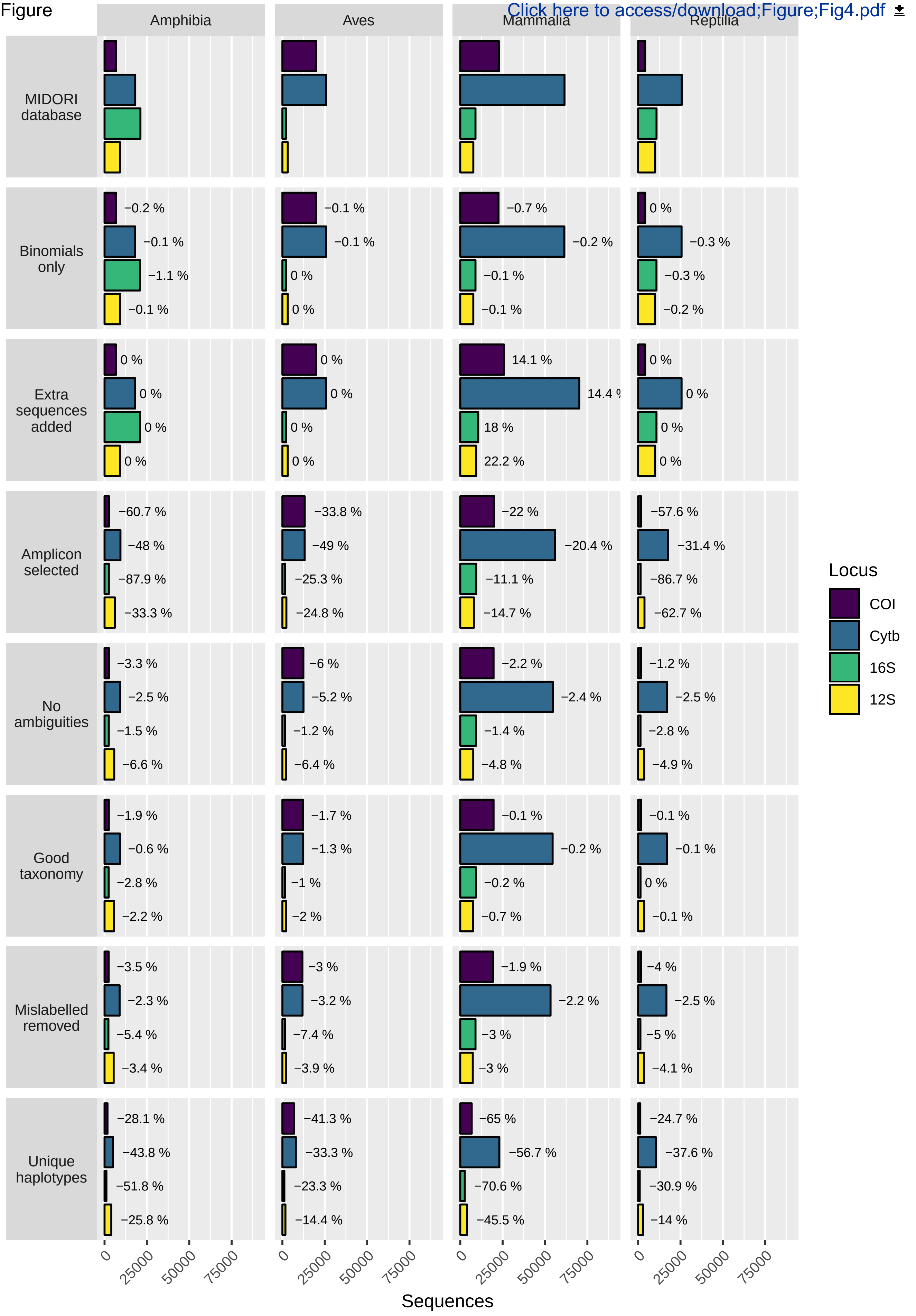

Figure

Click here to access/download;Figure;Fig5.pdf

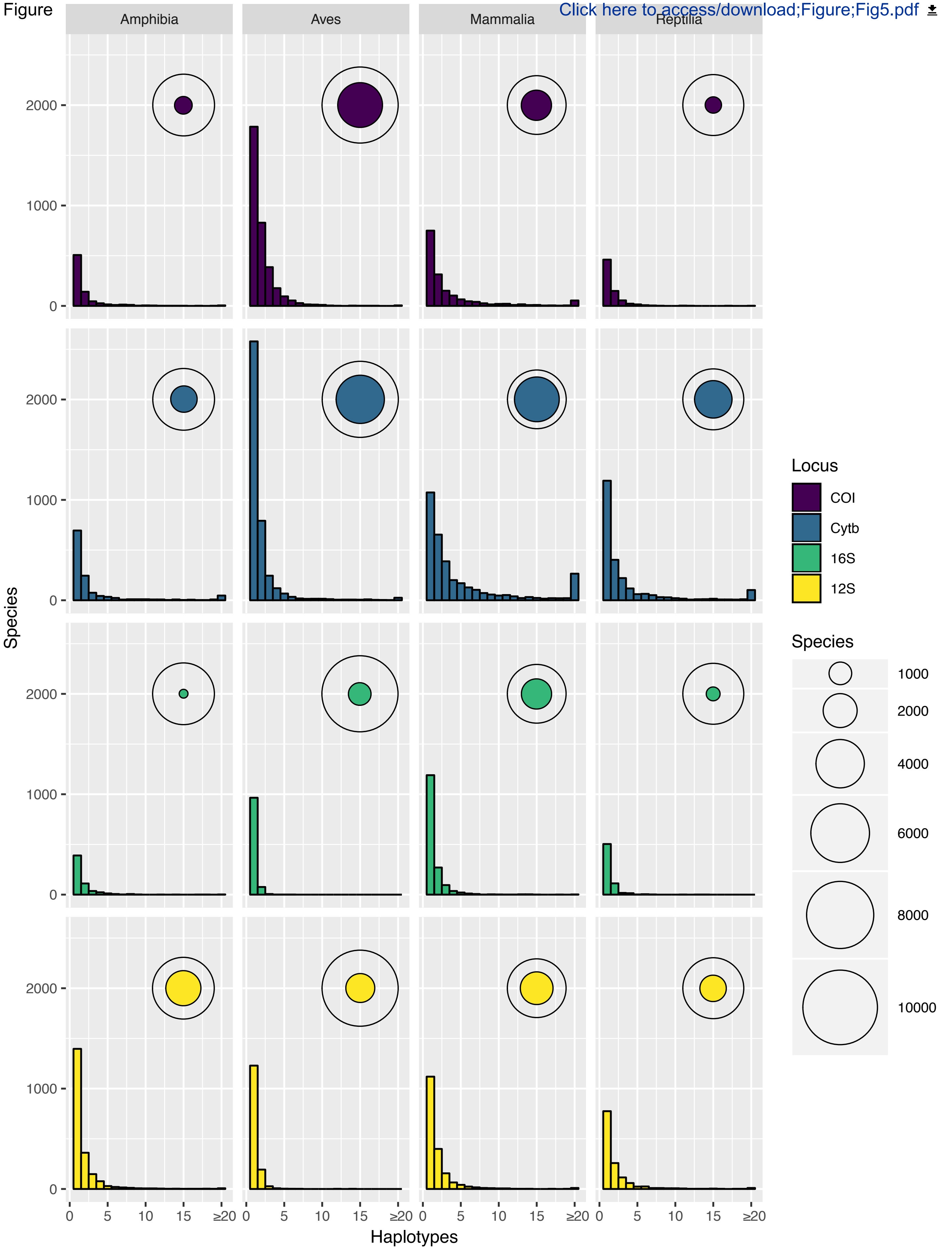

Figure

Click here to access/download;Figure;Fig6.pdf

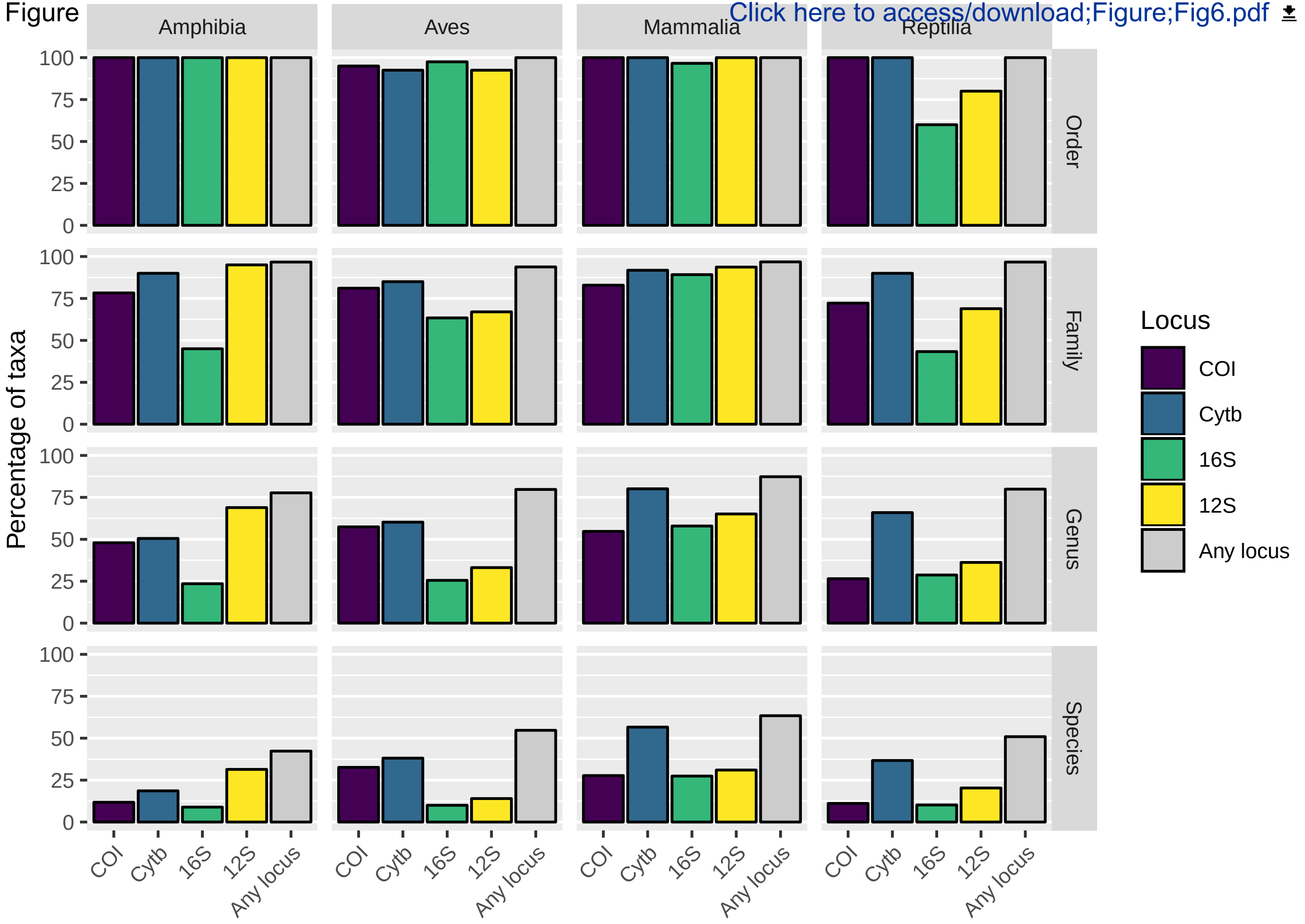

Figure

Click here to access/download;Figure;Fig7.pdf

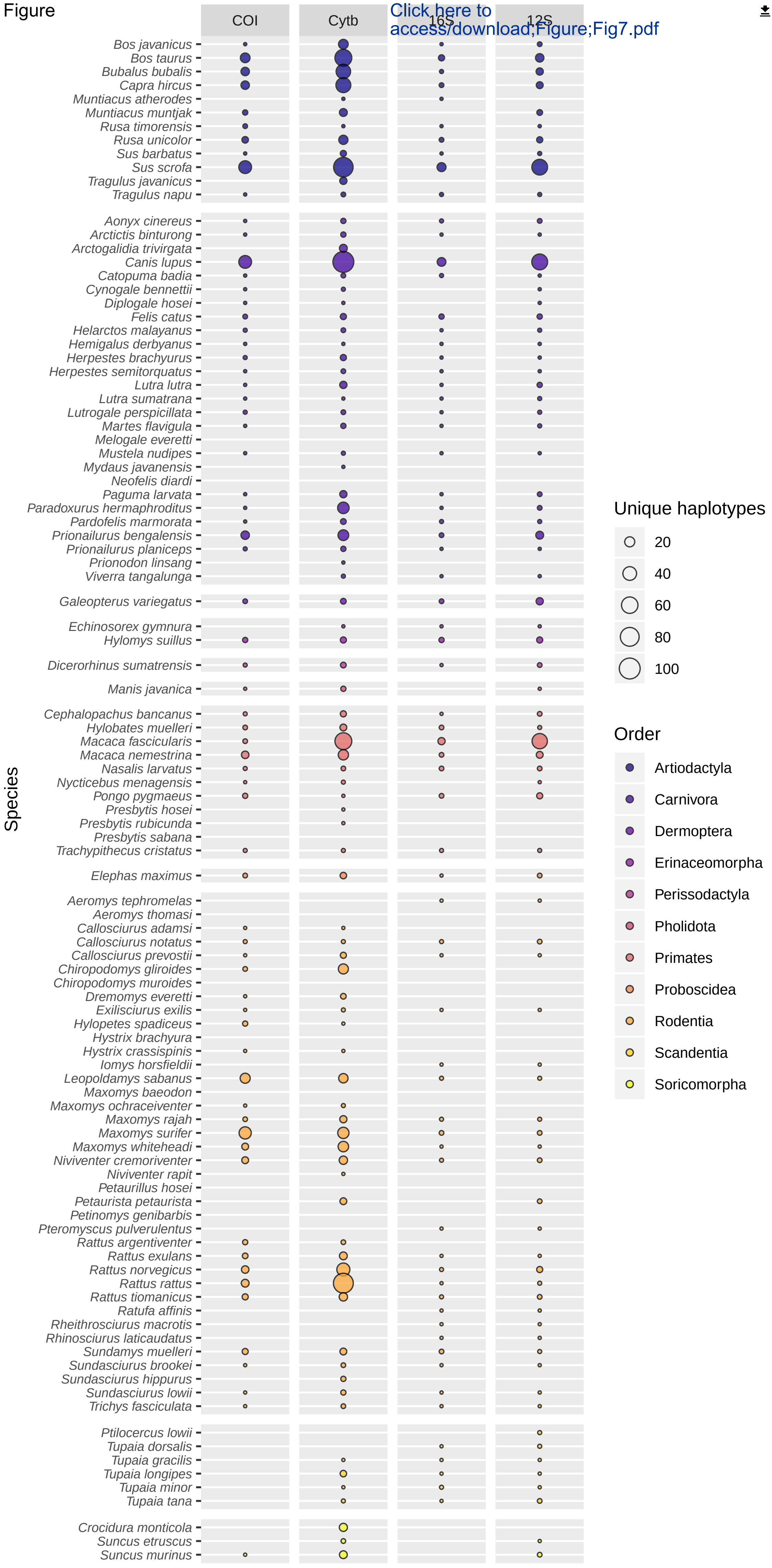

Click here to access/download
Supplementary Material
Suppl_Fig1.jpg

Click here to access/download
Supplementary Material
Supplemental table 1.pdf

Click here to access/download
Supplementary Material
Supplemental table 2.pdf

Click here to access/download
Supplementary Material
Supplemental table 3.pdf

Click here to access/download
Supplementary Material
Supplemental table 4.pdf

Dear Hongling Zhou,

First we would like to thank both reviewers for their positive feedback and the editor for the potential interest to publish our paper in GigaScience. Below we provide a detailed response to the remaining comments and suggestions by the reviewers. These certainly helped to improve the manuscript further and we thank the reviewers for their valuable comments.

On behalf of our co-authors,

Jan Axtner & Andreas Wilting

Reviewer reports:
Reviewer #1: Thank you for taking the time to address all comments in detail. The corrections I think have improved the clarity of the piece, and I feel convinced where you explained where I misunderstood. One possible reference to consider (given a comment about the availability of models to account for errors at multiple levels):

Guillera-Arroita. 2017. Dealing with false-positive and false-negative errors about species occurrence at multiple levels. Methods in Ecology and Evolution. https://doi.org/10.1111/2041-210X.12743
→ Thank you for the positive feedback and the interesting article. So far we were not aware of it, but as it fit's so perfectly to our topic, thus we now refer to it in line 561.

Reviewer #2: I am overall satisfied with the responses provided by the authors. In general, it is quite unlikely nowadays that there will be a consensus for the "right/best" way forward. It is always subject to practicality/funding. If i were to conduct my own amplicon seq project, will I follow this protocol to the dots - no. However, the bioinformatics scripts and data generated will be useful for better experimental design in the future. Furthermore, even if a method is robust, lab competency / human error (mislabeling, mixing the wrong index etc) is still going to be an issue.

Reviewer 1 raised the concern of similar tag1 being used repeatedly for multiple samples. I wonder if instead of using "Twin" tag, having a different tag1 combination (non-Twin tag?!) will be helpful (obviously for discussion). In other words, the forward and reverse primer combination in the 1st PCR round can be Tag1a for forward Tag1b for reverse. This is somewhat similar to dual indexing in Illumina but you're doing it at the initial stage and should will expand the 24 sample limitation for the tag1 based on my current understanding the twin-tag but happy to be proven wrong. With the increasing problem of index hoping particularly with the patterned flowcell for the Novaseq and Iseq (relevant to amplicon seq) , this should be useful and worth looking into.

See https://www.biorxiv.org/content/early/2017/10/19/205799

→ We agree that are other factors like lab skills or human errors that are an important issue and in fact our whole laboratory procedure is designed to minimize human-related errors. The whole workflow is designed to allow a high-throughput of samples in a maximum standardized way, i.e. sample aliquots are arranged already in eight-well stripes for the use of eight-channel pipets in order to minimize the risk of pipetting the wrong sample into the wrong well between the different replicates. That is also one of the reasons why we do not start mixing the tag1 combinations and re-use the 24 tags for each PCR plate. Our forward and reverse primers are already pre-mixed in an eight-well stripe and we use the same pipetting scheme with an eight-channel pipet for every 96-well PCR plate. If we would start using different tag combinations for each PCR plate we would have a much higher risk of pipetting errors mixing the wrong indices (handling 48 tubes is much more error prone than handling just three 8-well stripes).
In addition to this rather practical lab-work related reason we highlighted (Line: 602 to 605) that it is still very unlikely that the repeated use of tags for multiple samples causes accepted false positives in the end, as the final acceptance is not based on single occurrence but on repeated occurrence in independent replicates. We fully agree that the use of non-matching tags (e.g. A/B) would increase the number of samples that could be analysed in one sequencing run. But at the same time it would make it much harder to identify contaminations or tag jumps as we discuss in line 575 to 599. Contaminations of a PCR with another differently labelled PCR product would increase the number of chimeras in your PCR which would remain undetected if you would also use non-matching tag combination. The same holds true for tag-jumps, which are an issue in Illumina sequencing (see Schnell et al. 2015) and where we could demonstrate that our PCR libraries reduce the read-losses compared to adapter-ligation techniques (lines 585-594).Particular for the last reasons we favoured to use only twin-tag combinations.

→ We also thank the reviewer for the interesting paper, which also used quadruple-indexed libraries. We do however not see the application of RAD sequencing to identify invertebrate-derived DNA of unknown origin. Generally RAD sequencing requires high molecular weight genomic DNA. Our samples have a mixed pools of genomic and mitochondrial DNA from different organisms and our target DNA is often highly degraded, of poor quality and of low quantities. In addition we have the presence of high amounts of leech DNA. Therefore we currently do not see an application of this sequencing method.

"Also the read losses due to trimming and quality filtering were significantly lower for the 16S sequencing runs (1.3% and 5.3% in average, Supplemental Table 3) compared to the sequencing runs for the longer fragments of 12S and CytB (65.3% and 44.3% in average, Supplemental Table 3)."

The Usearch read overlapping pipeline is sensitive to number of mismatches in alignment. The Read2 in MiSeq 600 cycles run is particularly notoriously for being low quality towards the end of the run. Try trimming both R1 and R2 to 250 bp (length trimming) and redo the overlap and read loss calculation.

→ Thank you, for this valuable advice. We tested it for one of our 12S runs and compared results. As you suggested we trimmed the reads to 250 base pairs adjusted the -fastq_minovlen parameter for usearch from 50bp to just 25bp as we would expect to have a smaller overlap of the trimmed reads. In fact we obtained more read after merging (13,129,505 vs. 13,388,933). However, most of those reads were lost again after filtering so that our original settings produced in fact the most reads I the end (4,694,624 vs. 4,227,346). Thus we think it is reasonable to stick to the current settings in the pipeline.

Results original pipeline:

raw reads:	13,766,169
merging:	13,129,505
clipping:	6,498,738
filtering:	4,694,624

Trimmed reads (trimm 250bp, overlap 25bp):

raw reads:	13,766,169
merging:	13,388,933
clipping:	6,684,766
filtering:	4,227,346

"All three markers were amplified simultaneously for each batch of samples in a single PCR plate".

In different individual well?

→ Sorry for the misunderstanding, we did not do multiplex-PCR and amplified in individual wells. We added this to the sentence in lines 324-325:

"… All three markers were amplified simultaneously in individual wells for each batch of samples in a single PCR plate. …"

Because of different amplicon lengths and therefore different binding affinities to the flow cell
Also due to clustering efficiency . smaller fragment = easier to amplify

→ We agree, also due to DNA degradation we had higher amplification success for the shortest fragment (see lines 562 – 566). As we say in lines 337-340 "…Because of different amplicon lengths and therefore different binding affinities to the flow cell, 12S and CytB products were combined in a single library, whereas positive 16S products were always combined in a separate library. …" and these libraries were sequenced independently. To make this clearer we added a second sentence (line 340): "… 12S/CytB libraries were sequenced independently from 16S libraries…."
