# Supplementary material for: An efficient and robust laboratory workflow and tetrapod database for larger scale environmental DNA studies
Source: Gigascience. 2019 Apr 13;8(4):giz029. doi: 10.1093/gigascience/giz029 (PMC6461710; doi:10.1093/gigascience/giz029)
Supplement: Supplemental Files [file giz029_supplemental_files.zip › Supplemental table 1.pdf]

Supplemental table 1: Complete list of all used primer sequences in 5'-3' direction.

primer name	primer sequence	direction	primer length [bp]
12SfA	ACACTCTTTCCCTACACGACGCTCTTCCGATCTTGCATAAAAAGCTTCAAACTGGGATTAGATACCCCACTAT	forward	73
12SfB	ACACTCTTTCCCTACACGACGCTCTTCCGATCTTCAGCAAAAAGCTTCAAACTGGGATTAGATACCCCACTAT	forward	73
12SfC	ACACTCTTTCCCTACACGACGCTCTTCCGATCTAAGCGAAAAAGCTTCAAACTGGGATTAGATACCCCACTAT	forward	73
12SfD	ACACTCTTTCCCTACACGACGCTCTTCCGATCTACAAGAAAAAGCTTCAAACTGGGATTAGATACCCCACTAT	forward	73
12SfE	ACACTCTTTCCCTACACGACGCTCTTCCGATCTAGTGGAAAAAGCTTCAAACTGGGATTAGATACCCCACTAT	forward	73
12SfF	ACACTCTTTCCCTACACGACGCTCTTCCGATCTTTGACAAAAAGCTTCAAACTGGGATTAGATACCCCACTAT	forward	73
12SfG	ACACTCTTTCCCTACACGACGCTCTTCCGATCTCCTATAAAAAGCTTCAAACTGGGATTAGATACCCCACTAT	forward	73
12SfH	ACACTCTTTCCCTACACGACGCTCTTCCGATCTGGATGAAAAAGCTTCAAACTGGGATTAGATACCCCACTAT	forward	73
12SfI	ACACTCTTTCCCTACACGACGCTCTTCCGATCTCTAGGAAAAAGCTTCAAACTGGGATTAGATACCCCACTAT	forward	73
12SfK	ACACTCTTTCCCTACACGACGCTCTTCCGATCTCACCTAAAAAGCTTCAAACTGGGATTAGATACCCCACTAT	forward	73
12SfL	ACACTCTTTCCCTACACGACGCTCTTCCGATCTGTCAAAAAAAGCTTCAAACTGGGATTAGATACCCCACTAT	forward	73
12SfM	ACACTCTTTCCCTACACGACGCTCTTCCGATCTGAAGTAAAAAGCTTCAAACTGGGATTAGATACCCCACTAT	forward	73
12SfN	ACACTCTTTCCCTACACGACGCTCTTCCGATCTCGGTTAAAAAGCTTCAAACTGGGATTAGATACCCCACTAT	forward	73
12SfO	ACACTCTTTCCCTACACGACGCTCTTCCGATCTACCGAAAAAAGCTTCAAACTGGGATTAGATACCCCACTAT	forward	73
12SfP	ACACTCTTTCCCTACACGACGCTCTTCCGATCTACGTCAAAAAGCTTCAAACTGGGATTAGATACCCCACTAT	forward	73
12SfQ	ACACTCTTTCCCTACACGACGCTCTTCCGATCTAGACTAAAAAGCTTCAAACTGGGATTAGATACCCCACTAT	forward	73
12SfR	ACACTCTTTCCCTACACGACGCTCTTCCGATCTAGGAAAAAAAGCTTCAAACTGGGATTAGATACCCCACTAT	forward	73
12SfS	ACACTCTTTCCCTACACGACGCTCTTCCGATCTATTCCAAAAAGCTTCAAACTGGGATTAGATACCCCACTAT	forward	73
12SfT	ACACTCTTTCCCTACACGACGCTCTTCCGATCTCAATCAAAAAGCTTCAAACTGGGATTAGATACCCCACTAT	forward	73
12SfW	ACACTCTTTCCCTACACGACGCTCTTCCGATCTCCACAAAAAAGCTTCAAACTGGGATTAGATACCCCACTAT	forward	73
12SfX	ACACTCTTTCCCTACACGACGCTCTTCCGATCTGCTTAAAAAAGCTTCAAACTGGGATTAGATACCCCACTAT	forward	73
12SfY	ACACTCTTTCCCTACACGACGCTCTTCCGATCTGGTACAAAAAGCTTCAAACTGGGATTAGATACCCCACTAT	forward	73

primer name	primer sequence	direction	primer length [bp]
12SfZ	ACACTCTTTCCCTACACGACGCTCTTCCGATCTAACACAAAAAGCTT CAAACTGGGATTAGATACCCCACTAT	forward	73
12Sfctr	ACACTCTTTCCCTACACGACGCTCTTCCGATCTATCTGAAAAAGCTT CAAACTGGGATTAGATACCCCACTAT	forward	73
12SrA	GTGACTGGAGTTCAGACGTGTGCTCTTCCGATCTTGCATTGACTGCA GAGGGTGACGGGCGGTGTGT	reverse	67
12SrB	GTGACTGGAGTTCAGACGTGTGCTCTTCCGATCTTCAGCTGACTGCA GAGGGTGACGGGCGGTGTGT	reverse	67
12SrC	GTGACTGGAGTTCAGACGTGTGCTCTTCCGATCTAAGCGTGACTGCA GAGGGTGACGGGCGGTGTGT	reverse	67
12SrD	GTGACTGGAGTTCAGACGTGTGCTCTTCCGATCTACAAGTGACTGCA GAGGGTGACGGGCGGTGTGT	reverse	67
12SrE	GTGACTGGAGTTCAGACGTGTGCTCTTCCGATCTAGTGGTGACTGCA GAGGGTGACGGGCGGTGTGT	reverse	67
12SrF	GTGACTGGAGTTCAGACGTGTGCTCTTCCGATCTTTGACTGACTGCA GAGGGTGACGGGCGGTGTGT	reverse	67
12SrG	GTGACTGGAGTTCAGACGTGTGCTCTTCCGATCTCCTATTGACTGCA GAGGGTGACGGGCGGTGTGT	reverse	67
12SrH	GTGACTGGAGTTCAGACGTGTGCTCTTCCGATCTGGATGTGACTGCA GAGGGTGACGGGCGGTGTGT	reverse	67
12SrI	GTGACTGGAGTTCAGACGTGTGCTCTTCCGATCTCTAGGTGACTGCA GAGGGTGACGGGCGGTGTGT	reverse	67
12SrK	GTGACTGGAGTTCAGACGTGTGCTCTTCCGATCTCACCTTGACTGCA GAGGGTGACGGGCGGTGTGT	reverse	67
12SrL	GTGACTGGAGTTCAGACGTGTGCTCTTCCGATCTGTCAATGACTGCA GAGGGTGACGGGCGGTGTGT	reverse	67
12SrM	GTGACTGGAGTTCAGACGTGTGCTCTTCCGATCTGAAGTTGACTGCA GAGGGTGACGGGCGGTGTGT	reverse	67
12SrN	GTGACTGGAGTTCAGACGTGTGCTCTTCCGATCTCGGTTTGACTGCA GAGGGTGACGGGCGGTGTGT	reverse	67
12SrO	GTGACTGGAGTTCAGACGTGTGCTCTTCCGATCTACCGATGACTGCA GAGGGTGACGGGCGGTGTGT	reverse	67
12SrP	GTGACTGGAGTTCAGACGTGTGCTCTTCCGATCTACGTCTGACTGCA GAGGGTGACGGGCGGTGTGT	reverse	67
12SrR	GTGACTGGAGTTCAGACGTGTGCTCTTCCGATCTAGGAATGACTGCA GAGGGTGACGGGCGGTGTGT	reverse	67
12SrS	GTGACTGGAGTTCAGACGTGTGCTCTTCCGATCTATTCCTGACTGCA GAGGGTGACGGGCGGTGTGT	reverse	67
12SrT	GTGACTGGAGTTCAGACGTGTGCTCTTCCGATCTCAATCTGACTGCA GAGGGTGACGGGCGGTGTGT	reverse	67
12SrV	GTGACTGGAGTTCAGACGTGTGCTCTTCCGATCTCATGATGACTGCA GAGGGTGACGGGCGGTGTGT	reverse	67
12SrW	GTGACTGGAGTTCAGACGTGTGCTCTTCCGATCTCCACATGACTGCA GAGGGTGACGGGCGGTGTGT	reverse	67
12SrX	GTGACTGGAGTTCAGACGTGTGCTCTTCCGATCTGCTTATGACTGCA GAGGGTGACGGGCGGTGTGT	reverse	67

primer name	primer sequence	direction	primer length [bp]
12SrY	GTGACTGGAGTTCAGACGTGTGCTCTTCCGATCTGGTACTGACTGCA GAGGGTGACGGGCGGTGTGT	reverse	67
12SrZ	GTGACTGGAGTTCAGACGTGTGCTCTTCCGATCTAACACTGACTGCA GAGGGTGACGGGCGGTGTGT	reverse	67
12Srctr	GTGACTGGAGTTCAGACGTGTGCTCTTCCGATCTATCTGTGACTGCA GAGGGTGACGGGCGGTGTGT	reverse	67
16SfA	ACACTCTTTCCCTACACGACGCTCTTCCGATCTTGCATCGGTTGGGG TGACCTCGGA	forward	57
16SfB	ACACTCTTTCCCTACACGACGCTCTTCCGATCTTCAGCCGGTTGGGG TGACCTCGGA	forward	57
16SfC	ACACTCTTTCCCTACACGACGCTCTTCCGATCTAAGCGCGGTTGGGG TGACCTCGGA	forward	57
16SfD	ACACTCTTTCCCTACACGACGCTCTTCCGATCTACAAGCGGTTGGGG TGACCTCGGA	forward	57
16SfE	ACACTCTTTCCCTACACGACGCTCTTCCGATCTAGTGGCGGTTGGGG TGACCTCGGA	forward	57
16SfF	ACACTCTTTCCCTACACGACGCTCTTCCGATCTTTGACCGGTTGGGG TGACCTCGGA	forward	57
16SfG	ACACTCTTTCCCTACACGACGCTCTTCCGATCTCCTATCGGTTGGGG TGACCTCGGA	forward	57
16SfH	ACACTCTTTCCCTACACGACGCTCTTCCGATCTGGATGCGGTTGGGG TGACCTCGGA	forward	57
16SfI	ACACTCTTTCCCTACACGACGCTCTTCCGATCTCTAGGCGGTTGGGG TGACCTCGGA	forward	57
16SfK	ACACTCTTTCCCTACACGACGCTCTTCCGATCTCACCTCGGTTGGGG TGACCTCGGA	forward	57
16SfL	ACACTCTTTCCCTACACGACGCTCTTCCGATCTGTCAACGGTTGGGG TGACCTCGGA	forward	57
16SfN	ACACTCTTTCCCTACACGACGCTCTTCCGATCTCGGTTCGGTTGGGG TGACCTCGGA	forward	57
16SfO	ACACTCTTTCCCTACACGACGCTCTTCCGATCTACCGACGGTTGGGG TGACCTCGGA	forward	57
16SfP	ACACTCTTTCCCTACACGACGCTCTTCCGATCTACGTCCGGTTGGGG TGACCTCGGA	forward	57
16SfQ	ACACTCTTTCCCTACACGACGCTCTTCCGATCTAGACTCGGTTGGGG TGACCTCGGA	forward	57
16SfR	ACACTCTTTCCCTACACGACGCTCTTCCGATCTAGGAACGGTTGGGG TGACCTCGGA	forward	57
16SfS	ACACTCTTTCCCTACACGACGCTCTTCCGATCTATTCCCGGTTGGGG TGACCTCGGA	forward	57
16SfT	ACACTCTTTCCCTACACGACGCTCTTCCGATCTCAATCCGGTTGGGG TGACCTCGGA	forward	57
16SfV	ACACTCTTTCCCTACACGACGCTCTTCCGATCTCATGACGGTTGGGG TGACCTCGGA	forward	57
16SfW	ACACTCTTTCCCTACACGACGCTCTTCCGATCTCCACACGGTTGGGG TGACCTCGGA	forward	57

primer name	primer sequence	direction	primer length [bp]
16SfX	ACACTCTTTCCCTACACGACGCTCTTCCGATCTGCTTACGGTTGGGG TGACCTCGGA	forward	57
16SfY	ACACTCTTTCCCTACACGACGCTCTTCCGATCTGGTACCGGTTGGGG TGACCTCGGA	forward	57
16SfZ	ACACTCTTTCCCTACACGACGCTCTTCCGATCTAACACCGGTTGGGG TGACCTCGGA	forward	57
16Sfcrt	ACACTCTTTCCCTACACGACGCTCTTCCGATCTATCTGCGGTTGGGG TGACCTCGGA	forward	57
16SrA	GTGACTGGAGTTCAGACGTGTGCTCTTCCGATCTTGCATGCTGTTAT CCCTAGGGTAACT	reverse	60
16SrB	GTGACTGGAGTTCAGACGTGTGCTCTTCCGATCTTCAGCGCTGTTAT CCCTAGGGTAACT	reverse	60
16SrC	GTGACTGGAGTTCAGACGTGTGCTCTTCCGATCTAAGCGGCTGTTAT CCCTAGGGTAACT	reverse	60
16SrD	GTGACTGGAGTTCAGACGTGTGCTCTTCCGATCTACAAGGCTGTTAT CCCTAGGGTAACT	reverse	60
16SrE	GTGACTGGAGTTCAGACGTGTGCTCTTCCGATCTAGTGGGCTGTTAT CCCTAGGGTAACT	reverse	60
16SrF	GTGACTGGAGTTCAGACGTGTGCTCTTCCGATCTTTGACGCTGTTAT CCCTAGGGTAACT	reverse	60
16SrG	GTGACTGGAGTTCAGACGTGTGCTCTTCCGATCTCCTATGCTGTTAT CCCTAGGGTAACT	reverse	60
16SrI	GTGACTGGAGTTCAGACGTGTGCTCTTCCGATCTCTAGGGCTGTTAT CCCTAGGGTAACT	reverse	60
16SrK	GTGACTGGAGTTCAGACGTGTGCTCTTCCGATCTCACCTGCTGTTAT CCCTAGGGTAACT	reverse	60
16SrL	GTGACTGGAGTTCAGACGTGTGCTCTTCCGATCTGTCAAGCTGTTAT CCCTAGGGTAACT	reverse	60
16SrM	GTGACTGGAGTTCAGACGTGTGCTCTTCCGATCTGAAGTGCTGTTAT CCCTAGGGTAACT	reverse	60
16SrN	GTGACTGGAGTTCAGACGTGTGCTCTTCCGATCTCGGTTGCTGTTAT CCCTAGGGTAACT	reverse	60
16SrO	GTGACTGGAGTTCAGACGTGTGCTCTTCCGATCTACCGAGCTGTTAT CCCTAGGGTAACT	reverse	60
16SrP	GTGACTGGAGTTCAGACGTGTGCTCTTCCGATCTACGTCGCTGTTAT CCCTAGGGTAACT	reverse	60
16SrQ	GTGACTGGAGTTCAGACGTGTGCTCTTCCGATCTAGACTGCTGTTAT CCCTAGGGTAACT	reverse	60
16SrR	GTGACTGGAGTTCAGACGTGTGCTCTTCCGATCTAGGAAGCTGTTAT CCCTAGGGTAACT	reverse	60
16SrS	GTGACTGGAGTTCAGACGTGTGCTCTTCCGATCTATTCCGCTGTTAT CCCTAGGGTAACT	reverse	60
16SrT	GTGACTGGAGTTCAGACGTGTGCTCTTCCGATCTCAATCGCTGTTAT CCCTAGGGTAACT	reverse	60
16SrV	GTGACTGGAGTTCAGACGTGTGCTCTTCCGATCTCATGAGCTGTTAT CCCTAGGGTAACT	reverse	60

primer name	primer sequence	direction	primer length [bp]
16SrW	GTGACTGGAGTTCAGACGTGTGCTCTTCCGATCTCCACAGCTGTTATCCCTAGGGTAACT	reverse	60
16SrX	GTGACTGGAGTTCAGACGTGTGCTCTTCCGATCTGCTTAGCTGTTATCCCTAGGGTAACT	reverse	60
16SrY	GTGACTGGAGTTCAGACGTGTGCTCTTCCGATCTGGTACGCTGTTATCCCTAGGGTAACT	reverse	60
16SrZ	GTGACTGGAGTTCAGACGTGTGCTCTTCCGATCTAACACGCTGTTATCCCTAGGGTAACT	reverse	60
16Srctr	GTGACTGGAGTTCAGACGTGTGCTCTTCCGATCTATCTGGCTGTTATCCCTAGGGTAACT	reverse	60
CytBfA	ACACTCTTTCCCTACACGACGCTCTTCCGATCTTGCATAAAAAGCTTCCATCCAACATCTCAGCATGATGAAA	forward	73
CytBfB	ACACTCTTTCCCTACACGACGCTCTTCCGATCTTCAGCAAAAAGCTTCCATCCAACATCTCAGCATGATGAAA	forward	73
CytBfC	ACACTCTTTCCCTACACGACGCTCTTCCGATCTAAGCGAAAAAGCTTCCATCCAACATCTCAGCATGATGAAA	forward	73
CytBfE	ACACTCTTTCCCTACACGACGCTCTTCCGATCTAGTGGAAAAAGCTTCCATCCAACATCTCAGCATGATGAAA	forward	73
CytBfF	ACACTCTTTCCCTACACGACGCTCTTCCGATCTTTGACAAAAAGCTTCCATCCAACATCTCAGCATGATGAAA	forward	73
CytBfG	ACACTCTTTCCCTACACGACGCTCTTCCGATCTCCTATAAAAAGCTTCCATCCAACATCTCAGCATGATGAAA	forward	73
CytBfH	ACACTCTTTCCCTACACGACGCTCTTCCGATCTGGATGAAAAAGCTTCCATCCAACATCTCAGCATGATGAAA	forward	73
CytBfI	ACACTCTTTCCCTACACGACGCTCTTCCGATCTCTAGGAAAAAGCTTCCATCCAACATCTCAGCATGATGAAA	forward	73
CytBfK	ACACTCTTTCCCTACACGACGCTCTTCCGATCTCACCTAAAAAGCTTCCATCCAACATCTCAGCATGATGAAA	forward	73
CytBfL	ACACTCTTTCCCTACACGACGCTCTTCCGATCTGTCAAAAAAAGCTTCCATCCAACATCTCAGCATGATGAAA	forward	73
CytBfM	ACACTCTTTCCCTACACGACGCTCTTCCGATCTGAAGTAAAAAGCTTCCATCCAACATCTCAGCATGATGAAA	forward	73
CytBfN	ACACTCTTTCCCTACACGACGCTCTTCCGATCTCGGTTAAAAAGCTTCCATCCAACATCTCAGCATGATGAAA	forward	73
CytBfO	ACACTCTTTCCCTACACGACGCTCTTCCGATCTACCGAAAAAAGCTTCCATCCAACATCTCAGCATGATGAAA	forward	73
CytBfP	ACACTCTTTCCCTACACGACGCTCTTCCGATCTACGTCAAAAAGCTTCCATCCAACATCTCAGCATGATGAAA	forward	73
CytBfQ	ACACTCTTTCCCTACACGACGCTCTTCCGATCTAGACTAAAAAGCTTCCATCCAACATCTCAGCATGATGAAA	forward	73
CytBfR	ACACTCTTTCCCTACACGACGCTCTTCCGATCTAGGAAAAAAAGCTTCCATCCAACATCTCAGCATGATGAAA	forward	73
CytBfS	ACACTCTTTCCCTACACGACGCTCTTCCGATCTATTCCAAAAAGCTTCCATCCAACATCTCAGCATGATGAAA	forward	73
CytBfT	ACACTCTTTCCCTACACGACGCTCTTCCGATCTCAATCAAAAAGCTTCCATCCAACATCTCAGCATGATGAAA	forward	73

primer name	primer sequence	direction	primer length [bp]
CytBfV	ACACTCTTTCCCTACACGACGCTCTTCCGATCTCATGAAAAAAGCTT CCATCCAACATCTCAGCATGATGAAA	forward	73
CytBfW	ACACTCTTTCCCTACACGACGCTCTTCCGATCTCCACAAAAAAGCTT CCATCCAACATCTCAGCATGATGAAA	forward	73
CytBfX	ACACTCTTTCCCTACACGACGCTCTTCCGATCTGCTTAAAAAAGCTT CCATCCAACATCTCAGCATGATGAAA	forward	73
CytBfY	ACACTCTTTCCCTACACGACGCTCTTCCGATCTGGTACAAAAAGCTT CCATCCAACATCTCAGCATGATGAAA	forward	73
CytBfZ	ACACTCTTTCCCTACACGACGCTCTTCCGATCTAACACAAAAAGCTT CCATCCAACATCTCAGCATGATGAAA	forward	73
CytBrA	GTGACTGGAGTTCAGACGTGTGCTCTTCCGATCTTGCATAAACTGCA GCCCCTCAGAATGATATTTGTCCTCA	reverse	73
CytBrB	GTGACTGGAGTTCAGACGTGTGCTCTTCCGATCTTCAGCAAACTGCA GCCCCTCAGAATGATATTTGTCCTCA	reverse	73
CytBrC	GTGACTGGAGTTCAGACGTGTGCTCTTCCGATCTAAGCGAAACTGCA GCCCCTCAGAATGATATTTGTCCTCA	reverse	73
CytBrD	GTGACTGGAGTTCAGACGTGTGCTCTTCCGATCTACAAGAAACTGCA GCCCCTCAGAATGATATTTGTCCTCA	reverse	73
CytBrE	GTGACTGGAGTTCAGACGTGTGCTCTTCCGATCTAGTGGAAACTGCA GCCCCTCAGAATGATATTTGTCCTCA	reverse	73
CytBrF	GTGACTGGAGTTCAGACGTGTGCTCTTCCGATCTTTGACAAACTGCA GCCCCTCAGAATGATATTTGTCCTCA	reverse	73
CytBrG	GTGACTGGAGTTCAGACGTGTGCTCTTCCGATCTCCTATAAACTGCA GCCCCTCAGAATGATATTTGTCCTCA	reverse	73
CytBrH	GTGACTGGAGTTCAGACGTGTGCTCTTCCGATCTGGATGAAACTGCA GCCCCTCAGAATGATATTTGTCCTCA	reverse	73
CytBrI	GTGACTGGAGTTCAGACGTGTGCTCTTCCGATCTCTAGGAAACTGCA GCCCCTCAGAATGATATTTGTCCTCA	reverse	73
CytBrK	GTGACTGGAGTTCAGACGTGTGCTCTTCCGATCTCACCTAAACTGCA GCCCCTCAGAATGATATTTGTCCTCA	reverse	73
CytBrL	GTGACTGGAGTTCAGACGTGTGCTCTTCCGATCTGTCAAAAACTGCA GCCCCTCAGAATGATATTTGTCCTCA	reverse	73
CytBrM	GTGACTGGAGTTCAGACGTGTGCTCTTCCGATCTGAAGTAAACTGCA GCCCCTCAGAATGATATTTGTCCTCA	reverse	73
CytBrN	GTGACTGGAGTTCAGACGTGTGCTCTTCCGATCTCGGTTAAACTGCA GCCCCTCAGAATGATATTTGTCCTCA	reverse	73
CytBrO	GTGACTGGAGTTCAGACGTGTGCTCTTCCGATCTACCGAAAACTGCA GCCCCTCAGAATGATATTTGTCCTCA	reverse	73
CytBrP	GTGACTGGAGTTCAGACGTGTGCTCTTCCGATCTACGTCAAACTGCA GCCCCTCAGAATGATATTTGTCCTCA	reverse	73
CytBrQ	GTGACTGGAGTTCAGACGTGTGCTCTTCCGATCTAGACTAAACTGCA GCCCCTCAGAATGATATTTGTCCTCA	reverse	73
CytBrR	GTGACTGGAGTTCAGACGTGTGCTCTTCCGATCTAGGAAAAACTGCA GCCCCTCAGAATGATATTTGTCCTCA	reverse	73
CytBrS	GTGACTGGAGTTCAGACGTGTGCTCTTCCGATCTATTCCAAACTGCA GCCCCTCAGAATGATATTTGTCCTCA	reverse	73

primer name	primer sequence	direction	primer length [bp]
CytBrT	GTGACTGGAGTTCAGACGTGTGCTCTTCCGATCTCAATCAAACTGCAGCCCCTCAGAATGATATTTGTCCTCA	reverse	73
CytBrV	GTGACTGGAGTTCAGACGTGTGCTCTTCCGATCTCATGAAAACTGCAGCCCCTCAGAATGATATTTGTCCTCA	reverse	73
CytBrX	GTGACTGGAGTTCAGACGTGTGCTCTTCCGATCTGCTTAAAACTGCAGCCCCTCAGAATGATATTTGTCCTCA	reverse	73
CytBrY	GTGACTGGAGTTCAGACGTGTGCTCTTCCGATCTGGTACAAACTGCAGCCCCTCAGAATGATATTTGTCCTCA	reverse	73
CytBrZ	GTGACTGGAGTTCAGACGTGTGCTCTTCCGATCTAACACAAACTGCAGCCCCTCAGAATGATATTTGTCCTCA	reverse	73
CytBrctr	GTGACTGGAGTTCAGACGTGTGCTCTTCCGATCTATCTGAAACTGCAGCCCCTCAGAATGATATTTGTCCTCA	reverse	73
P5-A	AATGATACGGCGACCACCGAGATCTACACTGCATACACTCTTTCCCTACACGACGCTCTTCCGATCT	forward	67
P5-B	AATGATACGGCGACCACCGAGATCTACACTCAGCACACTCTTTCCCTACACGACGCTCTTCCGATCT	forward	67
P5-C	AATGATACGGCGACCACCGAGATCTACACAAGCGACACTCTTTCCCTACACGACGCTCTTCCGATCT	forward	67
P5-D	AATGATACGGCGACCACCGAGATCTACACACAAGACACTCTTTCCCTACACGACGCTCTTCCGATCT	forward	67
P5-E	AATGATACGGCGACCACCGAGATCTACACAGTGGACACTCTTTCCCTACACGACGCTCTTCCGATCT	forward	67
P5-F	AATGATACGGCGACCACCGAGATCTACACTTGACACACTCTTTCCCTACACGACGCTCTTCCGATCT	forward	67
P5-G	AATGATACGGCGACCACCGAGATCTACACCCTATACACTCTTTCCCTACACGACGCTCTTCCGATCT	forward	67
P5-H	AATGATACGGCGACCACCGAGATCTACACGGATGACACTCTTTCCCTACACGACGCTCTTCCGATCT	forward	67
P5-I	AATGATACGGCGACCACCGAGATCTACACCTAGGACACTCTTTCCCTACACGACGCTCTTCCGATCT	forward	67
P5-K	AATGATACGGCGACCACCGAGATCTACACCACCTACACTCTTTCCCTACACGACGCTCTTCCGATCT	forward	67
P5-L	AATGATACGGCGACCACCGAGATCTACACGTCAAACACTCTTTCCCTACACGACGCTCTTCCGATCT	forward	67
P5-M	AATGATACGGCGACCACCGAGATCTACACGAAGTACACTCTTTCCCTACACGACGCTCTTCCGATCT	forward	67
P5-N	AATGATACGGCGACCACCGAGATCTACACCGGTTACACTCTTTCCCTACACGACGCTCTTCCGATCT	forward	67
P5-O	AATGATACGGCGACCACCGAGATCTACACACCGAACACTCTTTCCCTACACGACGCTCTTCCGATCT	forward	67
P5-P	AATGATACGGCGACCACCGAGATCTACACACGTCACACTCTTTCCCTACACGACGCTCTTCCGATCT	forward	67
P5-Q	AATGATACGGCGACCACCGAGATCTACACAGACTACACTCTTTCCCTACACGACGCTCTTCCGATCT	forward	67
P5-S	AATGATACGGCGACCACCGAGATCTACACATTCCACACTCTTTCCCTACACGACGCTCTTCCGATCT	forward	67

primer name	primer sequence	direction	primer length [bp]
P5-T	AATGATACGGCGACCACCGAGATCTACACCAATCACACTCTTTCCCT ACACGACGCTCTTCCGATCT	forward	67
P5-V	AATGATACGGCGACCACCGAGATCTACACCATGAACACTCTTTCCCT ACACGACGCTCTTCCGATCT	forward	67
P7-A	CAAGCAGAAGACGGCATACGAGATTGCATGTGACTGGAGTTCAGACG TGTGCTCTTCCGATCT	reverse	65
P7-B	CAAGCAGAAGACGGCATACGAGATTCAGCGTGACTGGAGTTCAGACG TGTGCTCTTCCGATCT	reverse	63
P7-C	CAAGCAGAAGACGGCATACGAGATAAGCGGTGACTGGAGTTCAGACG TGTGCTCTTCCGATCT	reverse	63
P7-D	CAAGCAGAAGACGGCATACGAGATACAAGGTGACTGGAGTTCAGACG TGTGCTCTTCCGATCT	reverse	63
P7-E	CAAGCAGAAGACGGCATACGAGATAGTGGGTGACTGGAGTTCAGACG TGTGCTCTTCCGATCT	reverse	63
P7-F	CAAGCAGAAGACGGCATACGAGATTTGACGTGACTGGAGTTCAGACG TGTGCTCTTCCGATCT	reverse	63
P7-G	CAAGCAGAAGACGGCATACGAGATCCTATGTGACTGGAGTTCAGACG TGTGCTCTTCCGATCT	reverse	63
P7-H	CAAGCAGAAGACGGCATACGAGATGGATGGTGACTGGAGTTCAGACG TGTGCTCTTCCGATCT	reverse	63
P7-I	CAAGCAGAAGACGGCATACGAGATCTAGGGTGACTGGAGTTCAGACG TGTGCTCTTCCGATCT	reverse	63
P7-K	CAAGCAGAAGACGGCATACGAGATCACCTGTGACTGGAGTTCAGACG TGTGCTCTTCCGATCT	reverse	63
P7-L	CAAGCAGAAGACGGCATACGAGATGTCAAGTGACTGGAGTTCAGACG TGTGCTCTTCCGATCT	reverse	63
P7-M	CAAGCAGAAGACGGCATACGAGATGAAGTGTGACTGGAGTTCAGACG TGTGCTCTTCCGATCT	reverse	63
P7-N	CAAGCAGAAGACGGCATACGAGATCGGTTGTGACTGGAGTTCAGACG TGTGCTCTTCCGATCT	reverse	63
P7-O	CAAGCAGAAGACGGCATACGAGATACCGAGTGACTGGAGTTCAGACG TGTGCTCTTCCGATCT	reverse	63
P7-P	CAAGCAGAAGACGGCATACGAGATACGTCGTGACTGGAGTTCAGACG TGTGCTCTTCCGATCT	reverse	63
P7-Q	CAAGCAGAAGACGGCATACGAGATAGACTGTGACTGGAGTTCAGACG TGTGCTCTTCCGATCT	reverse	63
P7-R	CAAGCAGAAGACGGCATACGAGATAGGAAGTGACTGGAGTTCAGACG TGTGCTCTTCCGATCT	reverse	63
P7-T	CAAGCAGAAGACGGCATACGAGATCAATCGTGACTGGAGTTCAGACG TGTGCTCTTCCGATCT	reverse	63
P7-V	CAAGCAGAAGACGGCATACGAGATCATGAGTGACTGGAGTTCAGACG TGTGCTCTTCCGATCT	reverse	63
P7-W	CAAGCAGAAGACGGCATACGAGATCCACAGTGACTGGAGTTCAGACG TGTGCTCTTCCGATCT	reverse	63
P7-X	CAAGCAGAAGACGGCATACGAGATGCTTAGTGACTGGAGTTCAGACG TGTGCTCTTCCGATCT	reverse	63

primer name	primer sequence	direction	primer length [bp]
P7-Y	CAAGCAGAAGACGGCATACGAGATGGTACGTGACTGGAGTTCAGACGTGTGCTCTTCCGATCT	reverse	63
P7-Z	CAAGCAGAAGACGGCATACGAGATAACACGTGACTGGAGTTCAGACGTGTGCTCTTCCGATCT	reverse	6
