# Supplementary material for: An efficient and robust laboratory workflow and tetrapod database for larger scale environmental DNA studies
Source: Gigascience. 2019 Apr 13;8(4):giz029. doi: 10.1093/gigascience/giz029 (PMC6461710; doi:10.1093/gigascience/giz029)
Supplement: Supplemental Files [file giz029_supplemental_files.zip › Supplemental table 2.pdf]

Supplemental table 2: List of Bornean species that were weighted in the PROTAX assignment.

**Species**	**Species**	**Species**
Bos,javanicus	*Arctictis,binturong*	*Chiropodomys,muroides*
Bos,taurus	*Arctogalidia,trivirgata*	*Leopoldamys,sabanus*
Bubalus,bubalis	*Cynogale,bennettii*	*Maxomys,baeodon*
Capra,hircus	*Diplogale,hosei*	*Maxomys,ochraceiventer*
Muntiacus,atherodes	*Hemigalus,derbyanus*	*Maxomys,rajah*
Muntiacus,muntjak	*Paguma,larvata*	*Maxomys,surifer*
Rusa,timorensis	*Paradoxurus,hermaphroditus*	*Maxomys,whiteheadi*
Rusa,unicolor	*Prionodon,linsang*	*Niviventer,cremoriventer*
Sus,barbatus	*Viverra,tangalunga*	*Niviventer,rapit*
Sus,scrofa	*Galeopterus,variegatus*	*Rattus,argentiventer*
Tragulus,javanicus	*Echinosorex,gymnura*	*Rattus,exulans*
Tragulus,napu	*Hylomys,suillus*	*Rattus,norvegicus*
Canis,lupus	*Dicerorhinus,sumatrensis*	*Rattus,rattus*
Catopuma,badia	*Manis,javanica*	*Rattus,tiomanicus*
Felis,catus	*Macaca,fascicularis*	*Sundamys,muelleri*
Neofelis,diardi	*Macaca,nemestrina*	*Aeromys,tephromelas*
Pardofelis,marmorata	*Nasalis,larvatus*	*Aeromys,thomasi*
Prionailurus,bengalensis	*Presbytis,hosei*	*Callosciurus,adamsi*
Prionailurus,planiceps	*Presbytis,rubicunda*	*Callosciurus,notatus*
Herpestes,brachyurus	*Presbytis,sabana*	*Callosciurus,prevostii*
Herpestes,semitorquatus	*Trachypithecus,cristatus*	*Dremomys,everetti*
Mydaus,javanensis	*Pongo,pygmaeus*	*Exilisciurus,exilis*
Aonyx,cinereus	*Hylobates,muelleri*	*Hylopetes,spadiceus*
Lutra,lutra	*Nycticebus,menagensis*	*Iomys,horsfieldii*
Lutra,sumatrana	*Cephalopachus,bancanus*	*Petaurillus,hosei*
Lutrogale,perspicillata	*Elephas,maximus*	*Petaurista,petaurista*
Martes,flavigula	*Hystrix,brachyura*	*Petinomys,genibarbis*
Melogale,everetti	*Hystrix,crassispinis*	*Pteromyscus,pulverulentus*
Mustela,nudipes	*Trichys,fasciculata*	*Ratufa,affinis*
Helarctos,malayanus	*Chiropodomys,gliroides*	*Rheithrosciurus,macrotis*
Rhinosciurus,laticaudatus	*Tupaia,dorsalis*	*Crocidura,monticola*
Sundasciurus,brookei	*Tupaia,gracilis*	*Suncus,etruscus*

**Species**	**Species**	**Species**
Sundasciurus,hippurus	*Tupaia,longipes*	*Suncus,murinus*
Sundasciurus,lowii	*Tupaia,minor*	
Ptilocercus,lowii	*Tupaia,tana*	
