# Supplementary material for: An efficient and robust laboratory workflow and tetrapod database for larger scale environmental DNA studies
Source: Gigascience. 2019 Apr 13;8(4):giz029. doi: 10.1093/gigascience/giz029 (PMC6461710; doi:10.1093/gigascience/giz029)
Supplement: Supplemental Files [file giz029_supplemental_files.zip › Supplemental table 3.pdf]

Supplemental table 3: Summary of the read losses of each sample during the read processing steps for each sequencing run seperately. The first line gives the raw read number per sample. The losses are given as percentage of each step; 1. merging of the R1/R2 reads of the Illumina sequencing done by *usearch* [43; 44], 2. clipping of primers and trimming of reads using *cutadapt* [45], 3. quality filtering and 4. dereplication, both using usearch.

	Step	Mean	SD	Median	Min	Max
**SeqRun01**	raw	72977	96466	74	1	422271
	merging	7%	11%	2%	1%	50%
	clipping & trimming	2%	14%	0%	0%	100%
	filtering	4%	11%	2%	1%	100%
**SeqRun02**	raw	97372	83870	117626	1	409999
	merging	22%	23%	13%	2%	98%
	clipping & trimming	2%	13%	0%	0%	100%
	filtering	6%	3%	6%	5%	43%
**SeqRun03**	raw	57359	123971	48	1	1105978
	merging	5%	3%	5%	1%	11%
	clipping & trimming	43%	40%	28%	0%	100%
	filtering	37%	20%	29%	24%	100%
**SeqRun04**	raw	8629	10184	2075	1	37592
	merging	8%	2%	8%	6%	14%
	clipping & trimming	79%	34%	100%	0%	100%
	filtering	38%	18%	34%	0%	92%
**SeqRun05**	raw	77936	193818	36	1	1081947
	merging	34%	17%	36%	4%	89%
	clipping & trimming	50%	41%	59%	0%	100%
	filtering	53%	19%	51%	0%	100%
**SeqRun06**	raw	80816	80656	87013	1	407872
	merging	10%	15%	3%	1%	69%
	clipping & trimming	0%	0%	0%	0%	1%
	filtering	5%	1%	4%	4%	7%
**SeqRun07**	raw	90040	91022	81026	1	383072
	merging	23%	25%	10%	2%	99%
	clipping & trimming	1%	8%	0%	0%	100%
	filtering	6%	1%	6%	4%	10%
**SeqRun08**	raw	52951	132500	64	1	993255
	merging	14%	8%	17%	1%	26%
	clipping & trimming	89%	24%	100%	1%	100%
	filtering	49%	37%	28%	0%	100%
