# Supplementary material for: An efficient and robust laboratory workflow and tetrapod database for larger scale environmental DNA studies
Source: Gigascience. 2019 Apr 13;8(4):giz029. doi: 10.1093/gigascience/giz029 (PMC6461710; doi:10.1093/gigascience/giz029)
Supplement: Supplemental Files [file giz029_supplemental_files.zip › Supplemental table 4.pdf]

Supplemental table 4: Number of merged R1/R2 reads per sample that were used for the taxonomic assignment for each of the eight sequencing runs. Displayed are the median, minimum, maximum read numbers per PCR replicate, the mean and its standard deviation as well as the number of PCR replicates with less than 500 reads.

		SeqRun01	SeqRun02	SeqRun03	SeqRun04	SeqRun05	SeqRun06	SeqRun07	SeqRun08
median	16S	172,566	122,890				132,313	138,584	
min	16S	15	106				14,343	422	
max	16S	408,924	293,765				385,649	309,591	
mean	16S	162,487	110,274				126,365	120,850	
sd	16S	65,214	62,835				54,000	68,996	
< 500	16S	1	1				0	1	
median	12S			46,597	9,628	9,383			52,260
min	12S			2	3	3			1,164
max	12S			380,936	19,961	19,621			516,686
mean	12S			64,377	8,747	8,551			70,999
sd	12S			66,703	4,824	4,736			97,161
< 500	12S			9	62	62			49
median	CytB				8,428	8,218			53,104
min	CytB				3	3			2
max	CytB				19,961	19,621			608,948
mean	CytB				7,815	7,638			79,434
sd	CytB				5,473	5,365			120,055
< 500	CytB				21	21			13
