# Supplementary figures and images for: An efficient and robust laboratory workflow and tetrapod database for larger scale environmental DNA studies
Source: Gigascience. 2019 Apr 13;8(4):giz029. doi: 10.1093/gigascience/giz029 (PMC6461710; doi:10.1093/gigascience/giz029)

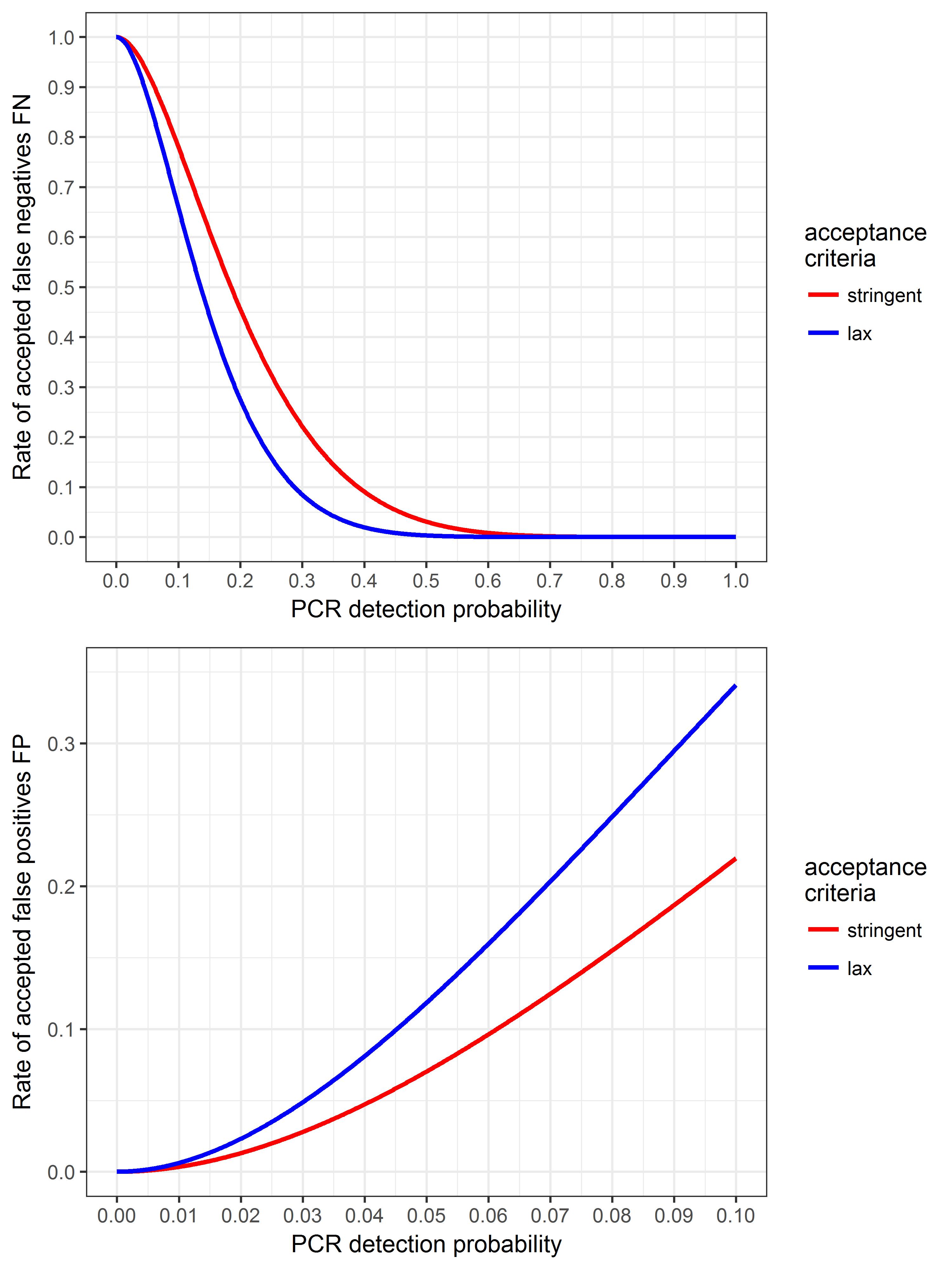

Supplement: Supplemental Files [file giz029_supplemental_files.zip › Suppl_Fig1.jpg]
