# Supplementary material for: An efficient and robust laboratory workflow and tetrapod database for larger scale environmental DNA studies
Source: Gigascience. 2019 Apr 13;8(4):giz029. doi: 10.1093/gigascience/giz029 (PMC6461710; doi:10.1093/gigascience/giz029)
Supplement: Response_to_Reviewer_Comments_Original_Submission.pdf [file giz029_response_to_reviewer_comments_original_submission.pdf]

Author's Response To Reviewer Comments

Close

Reviewer #1: In this manuscript Axtner et al. 1) used metabarcoding to assess mammal diversity from leeches and 2) developed a pipeline to build a curated reference database for taxonomic assignment. An emphasis of the metabarcoding was replication, including replication at the extraction, amplification, and locus levels. This work is of interest because 1) more robust inferences from metabarcoding may be possible by looking for concordance across replicates at multiple levels in the analysis process and 2) accurate taxonomic assignment is often limited by database accuracy and completeness. Thus, the manuscript is likely of interest not only to other iDNA users, but to metabarcoding users generally (e.g., eDNA, diet analysis, plant-pollinator interactions). I have three major comments and some more minor comments below. Generally, I think the authors could build a stronger case for their extensive lab and database work by taking a more quantitative approach to assessing success. Also, it was not very clear throughout the manuscript where to access the raw sequence data (FASTQ files from bcl2fastq probably fine), taxonomic assignments, scripts, etc. Places like Line 404 should include info on where to get the script.

Major comment 1:

Although I appreciate the value of replication, I think the manuscript would benefit from a quantitative assessment of the effect that replication had on inference accuracy. In the title the authors say this workflow is "improved". How can you demonstrate this?
-->We agree with the reviewer that we should have been more careful with our word choice in the title and that the word improved could raise the expectation of the reader that our approach was tested against existing protocols. As this was not the intention of this manuscript, we have replaced the word "improved" with "robust". The title now reads:
"An efficient and robust laboratory workflow and tetrapod database for larger scale eDNA studies"
We used the word improved in the first version as we wanted to highlight that there are currently no gold-standard protocols for e/iDNA studies, and results are often presented without a thorough reporting on how they were generated. This is the sense in which we expect our workflow to improve current practice.
The development of our workflow was driven by the need to analyse over 1000 samples with over 7500 leeches. Afterwards, we realized during discussions with colleagues that our approach and the pipelines we developed would be of value for other researchers. In particular, despite its obvious value to the community, PROTAX is very difficult to deploy, the original developer is focused on other projects, and so this manuscript is the only step-by-step explanation of how to implement it properly. We think the word robust is very defensible, given results from other publications (e.g. Protax shown to be improved over standard methods in Rodgers et al. 2017, and the use of multiple PCR replicates shown to be improved over standard methods in Zepeda-Mendoza 2016). Bringing this all together has not been done, which is where our publication comes in.

One of the main points of this manuscript is the value of technical replication to reduce false positive errors. Thus, each sample has replicate extractions, each extraction replicate loci, and each loci replicate PCR. As described, this is probably intuitively of value to folks who work with low-DNA applications. The idea being that something that is real should be something that you can detect repeatedly. What's lacking to me is a quantitative justification or assessment of these replication levels and the thresholds assigned to them for interpretation. Can you provide a quantitative answer to these questions?
- Does the rule of detection in 2/2 extraction replicates reduce estimated false positive rates compared to only 1/2 replicates?
-->Yes. We have added a simulation study to the supplements using different PCR detection rates (please see supplemental figure 1). This simulation shows that false positive detections can be reduced by the stringent A/B acceptance criterion (see below and supplemental figure 1) compared to more a lax acceptance criterion where we accepted assignment with at least two positives in any the PCR replicates. Our lax approach refers to one of the approaches of Ficetola et al. 2015 where they evaluated different

statistical approaches developed to estimate occupancy in the presence of observational errors and has been applied in other studies (e.g. Schnell et al. 2018). At the same time we see in this simulation that the rate of false negatives increased. We argue in the discussion that in some cases, e.g. rare and threatened species, it is more important to reduce false positives than to reduce false negatives. This is because there already exists a well-developed statistical procedure, occupancy modelling, that can correct for false negatives, whereas nothing nearly as well developed exists for correcting for false positives (see l. 550-61 in the manuscript). Non-detection within the laboratory process is just one of the factors influencing overall detection probability, which can be modeled in hierarchical occupancy models (see also Schnell et al. 2015). However, our workflow design allows the flexibility to address this issue if necessary and to use different acceptance criteria. To show and to address this we included now results on both, the stringent and the more lax approach in comparison in the manuscript.

- Is only requiring detection in 1/2 PCR replicates per marker sufficient, or would requiring 2/2 PCR replicates reduce the estimate false positive rate?
-->It turns out that requiring even 1/2 detections per marker would be unachievable for the 12S and CytB markers, because those markers had low amplification success, since the amplicon length is longer than the more successful 16S and we are working with degraded DNA. Furthermore the read quality decreased significantly with read length as we could demonstrate (see l. 566-68). Nevertheless these longer fragments helped often to disentangle the taxonomic inconsistencies caused by too high inter-specific genetic similarities in the 16S sequences or missing references in the database. In line with other publications (Ficetola et al 2015, Zepeda-Mendoza 2016, Schnell et al 2018) we think that reproducibility is important and that a detection of minimum two detection out of the PCR replicates (independently of the loci) is needed (added relaxed approach). But, in the manuscript, we also advocate using the criterion of requiring replicates from both A and B extractions to detect species if necessary, as this filter also removes possible contamination during DNA extraction and gives even higher confidence in the results. Our workflow does allow to apply such a more strict view if it is needed.

- What is the effect of the used 10 reads threshold versus other thresholds (e.g., 5, 50, 100) on the estimate false positive rate? How did you --determine that this threshold could be dropped if the taxon was detected with >1 locus?
-->10 reads was chosen because we realized that often a low number of reads were assigned to a sister taxon due to amplification or sequencing errors etc. (see l. 642-47). Obviously a threshold of 10 is chosen somewhat arbitrarily and it certainly depends on many factors such as sequencing depth, used PCR cycles, amplicon length and/or other factors affecting PCR stochasticity. Thus it needs to be evaluated based on the individual setup and dataset, but after analyzing hundreds of leech samples, this threshold proved to give internally consistent results. As the most likely contaminations in the lab would be amplicons we dropped the threshold if a taxon occurred in more than one marker as we believe it is unlikely that amplicons of two markers of the same taxon contaminate the same other sample. Note that other researchers are not held to this specific threshold and will need to make a judgement as well, guided by the internal consistency of the results.

I'd suggest that if you can't answer these with empirical data or a reasonable probability model, then you can't really argue that your replication approaches were any "better" than any other given approach.
-->As said above, we have changed our title and wording to emphasize that our goal is to present a detailed workflow with many internal checks, all of which are expected to reduce false-positive detections. Perhaps the biggest value of this manuscript is that it will help other researchers consider all the possible sources of error in their own e/iDNA pipelines. We of course provide a cost-efficient mitigation for those sources of error, but other researchers will likely come up with others. However, our workflow offers some advantages, 1. it reduces the risk of false identifications due to wrong annotated references, 2. the multiple-marker approach allows to identify more species with regard to incomplete reference databases, 3. it gives you more control over different levels uncertainties during the laboratory steps and thus more confidence if this is needed.
That said, we did compare our results to the DAMe pipeline (now added in l. 588-94), which also uses twin-tagging to detect and remove tag-jump events, but they used a blunt-ligation technique to carry out library prep (in contrast to our PCR-based method). DAMe’s authors report tag jumping in 19.15% and 23.1% of sequences (Zepeda-Mendoza 2016, table 1), whereas we found only 4.6% of reads with non-matching tags (t1 or t2) (table 4). Our number might not be one to one comparable as they

counted unique sequences and we reported on read numbers, but this is a big difference and is in favour of our method.
More generally, as the reviewer alludes to, the field of metabarcoding (in a/i/eDNA) has already identified many sources of error and also suggested remedies (e.g. Ficetola et al. 2015). Our contribution here is to synthesize this knowledge in an integrated workflow that considers both the wet- and dry-lab portion, including extensive work on the taxonomic-assignment step, which is, to our mind, the least well developed step in metabarcoding currently.
Also, we do not pretend to define universal filtering thresholds for all studies, as the desirable stringency depends in part on one's research question. For occupancy modelling, it is crucial to avoid false-positives, even at the expense of false-negatives. False-positive detections can lead to misdirected conservation effort, especially for very rare, very high-value species like the Saola, the large-antlered Muntiac or Sumatran rhino. If, on the other hand, one is working on more abundant and widespread species, then a low level of false-positives will not likely influence the inferred species distributions. Finally, identifying assignments as likely false negatives still allows practitioners to flag up those unreliable assignments and make a management decision on what to do, such as to combine with other information about the sample site and perhaps to increase sampling effort in those sites. We have highlighted this more clearly in the manuscript now by adding the following section and supplemental figure 1:
L. 543-57 "...We only accepted sequences that appeared in a minimum of two independent PCRs for the lax and for the stringent criterion, where it has to occur in each extraction replicate A and B (figure 1). The latter acceptance criterion is quite conservative and produces higher false negative rates than e.g. accepting occurrence of at least two positives. However, it also reduces the risk of accepting a false positives compared to it (see supplemental figure 1. for a simulation of false positive and false negatives rates within a PCR), especially with increasing risk of false positive occurrence in a PCR for e.g. example due to higher risk of contamination etc.. Metabarcoding studies are very prone to false negatives, and downstream analyses like occupancy models for species distributions can account for imperfect detection and false negatives. However, methods for discounting false positive detections are not well developed [60]. Thus we think it is more important to avoid false positives, especially if the results will be used to make management decisions regarding rare or endangered species. In contrast, it might be acceptable to use a relaxed acceptance criterion for more common species, as long as the rate false-positives/true-positives is small and does not affect species distribution estimates. Employing both of our tested criteria researchers could flag unreliable assignments and management decisions can still use this information, but now in a forewarned way. ..."

There's another potential issue here, which is not discussed, which is the false negative rate. By requiring replicated detections, you drive down your false positive rate, but drive up your false negative rate. If the false negative rate per extraction/PCR is very low, maybe this doesn't matter much, but it could be quite large. For example, there is a recent discussion in the literature related to this idea with a focus on PCR replicates:
Ficetola et al. 2015. Replication levels, false presences and the estimation of the presence/absence from eDNA metabarcoding data. Molecular Ecology Resources. https://doi.org/10.1111/1755-0998.12338
Lahoz-Monfort et al. 2016. Statistical approaches to account for false-positive errors in environmental DNA samples. Molecular Ecology Resources
Ficetola et al. 2016. How to limit false positives in environmental DNA and metabarcoding? Molecular Ecology Resources
--> Yes, we are aware of this discussion and have cited the relevant papers. As said above we have expanded our discussion on false positives and false negative rates in the manuscript and also added the simulation in the supplements. As we discuss above, we generally lean toward trying to reduce the false-positive rate at the cost of driving up the false-negative rate, due to the fact that occupancy modelling is built for the former error. And also, driving up the false-negative rate does not mean deleting the data. It just means flagging some assignments as being unreliable, and management decisions can still use this information, but now in a forewarned way.
More quantitatively, with our stringent criteria we are increasing our false negative rate (see added supplementary figure 1). Here is a simple example from our simulation: If we assume a low detection probability of 0.25 for every PCR, and if we treat all 12 replicates alike and accept every assignment that occurs in at least 2 replicates (lax criteria), we would have a chance of 84% to detect a species correctly. If we instead use the stringent criteria, this chance decreases to 64%. But if we assume a 50% chance to detect a present DNA with the PCR both acceptance criteria are already below a 5% to accept a false negative. The tricky thing remains to decide what our chances are to detect a present

DNA correctly.
However, the stringent criterion evidently also decreases the risk of accepting false positives especially for higher risks (see supplementary figure 1), e.g. if you have low PCR detection probabilities due to low amounts of target DNA and you have to run high cycle PCR. We agree and think researchers have to be aware of these risks and due to your comment we decided to present now the two acceptance criteria. To us most important is that our workflow design gives users the flexibility to apply a acceptance criteria that fits their needs and that users are aware of the risks applying too lax approaches. In some cases it is more important to avoid false positives than having false negatives. Lahoz-Monfort et al. (2016) also pointed out that multiscale occupancy models that account for both FN and FP errors are not yet developed and that FP errors can originate at several stages of eDNA collection and analysis. As said, occupancy model are very sensitive to false positives in the datasets but assume an imperfect detection and thus are less affected by false negatives. In the manuscript we also discuss the meaning of false positives for conservation measurements and why we think it is most important to avoid them while accounting for false negatives (see l. 107-110).

Major comment 2:

For the database, I would have liked to see the authors show that their database curation decreased the false positive rate or in some other way increased the accuracy of their inferences. Is this curated database approach necessary to apply PROTAX, or could the original sequences with redundancy and mislabeling have been used as well? If this pipeline is a major product in the manuscript (as the Abstract suggests) how can you quantitatively demonstrate to the reader that it is worth using?
-->The curated database is not required for using Protax, and Protax of course is not required for using the curated database (or for using an uncurated database, of course). We do not have estimates here of the reduction in error when using Protax, but Rodgers et al. (2017) did this analysis already, using an earlier version of Protax in Barro Colorado Island, Panama. The benefit of that study is that the vertebrate community is very well characterized, and the fly metabarcoding results could be checked against this a priori knowledge. In their Figure 2, Protax resulted in a much lower false positive rate (esp. for 12S species-rank assignments, and especially if we weighted the results to favor species known from Panama or from BCI itself).
On the other side, Protax was as good or better than BLAST or BLAST+Megan for true-positive detections (although this varied a bit depending on which gene and which taxonomic rank). These results were run on a non-curated database.
The other part of the question is whether curation is necessary. We think the answer to this is a self-evident yes. Errors in a reference database cannot help but result in errors, either to an overly confident assignment or to an overly vague assignment. As an easily replicated example, human sequences are often assigned by BLAST+Megan to very high ranks, such as Eukarya, because human sequences have been incorrectly uploaded in place of other taxa of interest (ranging all over the Eukarya). There is good reason to believe that Genbank contains a large number of errors, as our own results showed (e.g. the many sequences flagged by SATIVA showing inconsistency of taxonomy with phylogenetic location) and also by Nilsson et al. (2006), who suggest that as many as 20% of GenBank fungal sequences are mislabeled.

Major comment 3:

It took be a bit of time to grasp the potential value of doing two rounds of PCR with sets of doubly-indexed primers. It wasn't completely clear to me that different combinations of first round and second round indices were used to increase the level of multiplexing. I think the proposed benefits of this approach could use a bit more explanation, including some caveats.
-->We apologize that the presentation including the benefits of our approach were not clear enough. We rephrased this section to make it clearer for the reader. These sections read now:
L. 294-309, Methods: "...We modified primers of the three markers to avoid the production of unlabelled PCR products, to allow the detection and deletion of tag-jumping events [43], and to reduce the cost of primers and library preparation. We used two rounds of PCR. The first round amplified the target gene and attached one of 25 different 'twin-tag' pairs (tag 1), identifying the sample within a given PCR. By 'twin-tag,' we mean that both the forward and reverse primers were given the same sample-identifying

sequence (‘tags’) added as primer extensions (Fig. 2). The tags differed with a minimum pairwise distance of three nucleotides ([43]; Supplemental Table 1). These primers also contained different forward and reverse sequences (Read 1 & Read 2 sequence primers) (Supplemental Table 1) to act priming sites for the second PCR round (Fig. 2).
The second round added the Illumina adapters for sequencing and attached one of 20 twin-tag pairs (tag 2) identifying the PCR, with a minimum pairwise distance of three [44]. These primers also contained the Illumina P5 and P7 adapter sequences (Fig. 2). Thus no unlabelled PCR products were ever produced, and the combination of tags 1 and 2 allowed the pooling of up to 480 (=24 X 20) samples in a single library preparation step (one tag 1 was reserved for controls). Twin tags allowed us later to detect and delete tag jumping events [43] (Fig. 2).....”
L. 575-85, Findings: “...This ensures that unlabelled PCR products are never produced and allows us to multiplex a large number of samples on a single run of Illumina MiSeq run. Just 24 sample tags 1 and 20 plate tags 2 allow the differentiation of up to 480 samples with matching tags on both ends. The same number of individual primers would have needed longer tags to maintain enough distance between them and would have resulted in an even longer adapter-tag overhang compared to primer length. This would have most likely resulted in lower binding efficiencies due to steric hindrances of the primers. Furthermore, this would have resulted in increased primer costs. Thus our approach reduced sequencing and primer purchase costs while at the same time largely eliminating sample mis-assignment via tag jumping, because tag-jump sequences have non-matching forward and reverse tag 1 sequences [43]......”
L. 601-05, Findings: “...However, we would not be able to detect a contamination prior the second PCR from one plate to another, as we used the same 24 tags (tag 1) for all plates. Nevertheless such a contamination is very unlikely to result in any accepted false positive as it would be improbable to contaminate both the A and B replicates, given the exchange of all reagents and the time gap between the PCRs....”
Although we do not necessarily see important caveats we highlighted now that the costs to purchase the long oligos are more expensive and that the laboratory analysis might be more time consuming, as each sample requires an individual PCR master mix. Besides this we actually see only advantages of the double twin-tagging (see l. 591-99)

1) This approach *does* potentially reduce contamination risk as compared to two-round PCR metabarcoding protocols where the first round of amplification is done with tailed, unlabeled primers (or when adaptors are ligated). However, if you re-use first-round indices for multiple libraries, you will generate PCR products with the *same* labels in the first round of PCR. Perhaps this issue may be somewhat mitigated by preparing libraries in batches so that no libraries with the same indexing primers are prepared simultaneously. This caveat probably also applies to the discussion in Line 545. If you re-use first-round indices for multiple projects, PCR products from one study can show up in another. Re-using indexing primers seems highly likley given the expense of long, purified oligos - it doesn't seem affordable to use the first-round primers for only a single library prep. The risk *is* probably lower (because 1/25 libraries have that index, as opposed to 25/25 when unlabeled), but it's not completely unambiguous.
-->Yes, we can never rule out the risk of contamination in the lab, we can only try to minimize it. One option would have been to increase the number of available tags, but this would result –as said by the reviewer- in an increase in costs and in an even longer adapter and tag overhang compared to primer length. This might as well result in lower binding efficiencies due to steric hindrances. Being limited from this side we decided to go for the two-round PCR. At the moment we have about 1200 leech samples so there is no way not to re-use a tag for another sample. However, we tried to minimize contamination risk by lab-workflow measures. As the reviewer suggested, we never used tags simultaneously, we used always 24 different t1 tags on one PCR plate. The only chance would be a contamination of amplicons from the first PCR round to another PCR plate prior the second PCR round. Our laboratory workflow tried to minimize that risk. But even if we have a contamination of a PCR product of the first round, it would have to have contaminated at least two replicates of the same sample, which seems unlikely to us, as we took care to use fresh reagents for each PCR replicate.

2) I am not convinced by the current description that this approach allows removal of chimeric sequences. However, my uncertainty may largely stem from my confusion about what you mean by

"chimeric sequence". My understanding is that a "chimera" or "chimeric sequence" is a single molecule that came from two different transcripts. For example, an incompletely-extended PCR product anneals to and extends on a similar, but different template from the original. Resulting reads reflect a composite sequence formed by PCR.
Such a "chimeric sequence" that forms *within* a single library cannot be detected based on paired index sequences. All of the PCR products have the same index sequences on each both ends. Thus, a chimera formed between species A and species B is indistinguishable from a PCR product from species A based on the index sequences alone. I don't think that this is the type of "chimeric sequence" that you're worried about, but it can affect taxonomic assignment (perhaps the authors can explain the sensitivity of PROTAX to these types of errors).
The other type of chimeric sequence that is more problematic is when a molecule has an index for library #1 on one end and an index for library #2 on the other. If you have double-indexed libraries with only one P5/P7 combination per library, then you can remove reads from these PCR products. I think this is the type of chimeric sequence the authors are concerned about? In which case, I'm a bit confused about two points: First, how is it possible to form physical chimeras if each library is amplified by itself and pooled only for sequencing? My understanding is that incorrectly-tagged reads from this protocol come from sequencing errors on the flow cell, rather than being due to the presence of chimeric molecules. Maybe carefully distinguishing between sequences (molecules) and reads (MiSeq output) would help me to track with you. Second why would two-rounds of indexing be better at detecting these types of errors than a single round? Can you show me with a cartoon on Figure 2?
-->You are right; our wording was not precise enough in this context. Real chimeric sequences, as you describe them, cannot be detected within a single sample. If they occur they are, however, very unlikely to result in any proper assignment. We were talking of reads with different tags on both ends and -as the reviewer points out correctly- the two-rounds PCR does not help to detect them. Only the twin-tagging described here allows the identification of such non-matching tags. Such reads have been observed before (Schnell et al. 2015) and they increase the risk of mis-identification of reads. Hypotheses to explain their occurrence are so far that such “tag jumps” happen during blunt-ending of indexed amplicons or during bulk amplification during library index PCR (see Schnell et al. 2015). We see this hypothesis confirmed by that fact that tag-jumps increased if we pooled the samples prior to the second PCR. Thus the bulk amplification increased the tag-jump rate drastically. However both explanations fail for our described protocol. Thus we were quite surprised to see, nonetheless, 2.5% reads with non-matching tags. We see only two potential explanations here, both would require further testing; first contaminated primers or second, mixed clusters during sequencing. However, we exchanged the word chimeric to non-matching to avoid any confusion.

More minor comments:

Line 187: Later you report a range of values for percent reads from Mammalia, so these must be 58 individually-indexed libraries? How were the libraries prepared (e.g., shearing, indexing, how was quantity assessed for pooling)? Bioinformatics for these unclear. We assume there was some quality filtering steps and rules associated with assignment? If your goal is to assess enrichment success with PCR, would you want to use a comparable pipeline across this experiment and the amplified libraries?
--> We apologize that we have not provided the details about the shot-gun sequencing. We rather saw this as additional data. However for completeness we have now added the protocols and details. The section reads now:
L. 440-450: “...As the success of the metabarcoding largely depends on the mammal DNA quantity in our leech bulk samples we quantified the mammalian DNA content in a subset of 58 of our leech samples using shotgun sequencing. Extracted DNA was sheared with a Covaris M220 focused-ultra-sonicator to a peak target size of 100-200 bp, and re-checked for size distribution. Double-stranded Illumina sequencing libraries were prepared according to a ligation protocol designed by Fortes and Paijmans [51] with single 8 nt indices. All libraries were pooled equimolarly and sequenced on the MiSeq using the v3 150-cycle kit. We demultiplexed reads using bcl2fastq and cutadapt for trimming the adapters. We used BLAST search to identify reads and applied Metagenome Analyzer MEGAN [30] to explore the taxonomic content of the data based on the NCBI taxonomy. Finally we used KRONA [52] for visualisation of the results. ...”

Line 191: Would be helpful to justify these primer sets a bit. Why would we expect them to be suitable

for this application?
--> We added the following justification to the manuscript:
L. 285-88: "...The primers were chosen on the expectation of successful DNA amplification over a large number of tetrapod species [41; 42], and we tested the fit of candidate primers on an alignment of available mitochondrial sequences of 134 Southeast-Asian mammal species. Primer sequences are in Table 1 …"

Line 253: Spell out acronym on first use.
-->It seems like MIDORI is not an acronym (although it sounds like one) but a name and we have found inconsistent writing of it in the according papers (Machida et al. 2017, Leray et al. 2018). In Japanese it seems to be the word for "green" thus we assume it is just a name and changed it to "Midori" throughout the manuscript.

Line 266: If there are 7 previously unpublished mitochondrial genomes, why are there 13 Accession Numbers here? Are these GenBank Accession Numbers? Entering a few of them into GenBank did not result in any sequences.
-->For Mustela nuidpes and Nesolagus timminsi we only uploaded the 12S, 16S, COI and CytB fragments that were part of our reference database (8 Acc.No.). The full mitogenomes of these two species are part of studies of collaborators and thus we could not release the full mitogenoms. For Herpestes semitorquatus, Diplogale hosei, Hemigalus derbyanus, Viverra tangalunga, Paradoxurus jerdoni we uploaded complete or almost complete mitogenomes (5 Acc.No.). During submission the GenBank entries were still held confidential but we released them now.
In addition to the sequences submitted to Genbank, fastq files from the sequencing runs for each leech sample are uploaded to the European Nucleotide Archive ENA and can be found via the study accession number ERP109441.

Line 485: Not sure which 554 species this is. I thought we were talking about the 103 species expected in the sampling area.
--> Thank you for reading so carefully, it is in fact a mistake and we apologize for it. The correct number is 55, we corrected it.

It was not super clear - when a locus did not amplify (checked via gel electrophoresis), did you drop those PCR products from the library pool? Were all amplicons pooled equimolarly (you say "samples" here)?
-->First we tried not to drop the samples from the library pool in order not to miss something in case the staining did not work correct. But including these "DNA-free" samples resulted in much diluted libraries, which could not be sequenced as a certain molarity is needed for sequencing. Thus we needed to exclude those samples after attempts failed to up-concentrate the libraries (a step which might also bring additional risks of contamination). We pooled the amplicons of a genetic marker equimolarly, so all A1-16S PCR products were pooled equimolarly together, all A2-16S PCR products.... We clarified in the manuscript which amplicons were pooled together. This section reads now:
L 336-42: "....Sequencing libraries were made by equimolar pooling of all positive amplifications; final concentrations were between 2 and 4 nmol. Because of different amplicon lengths and therefore different binding affinities to the flow cell, 12S and CytB products were combined in a single library, whereas positive 16S products were always combined in a separate library. Apart from our negative controls, we did not include samples that did not amplify, as this would have resulted in highly diluted libraries. Up to 11 libraries were sequenced on each run of Illumina MiSeq, following standard protocols...."

How did you make the list of 103 mammal species known to be present? Why is Homo sapiens not in this list?
-->We are working on Borneo for more than ten years, mostly biodiversity studies based on camera

trapping, thus we have accumulated extensive knowledge about the occurrence of species within our study site. For smaller mammalian groups we used additional information from collaborators working in Sabah and for species without existing knowledge about their distribution we included these species in the list. In fact Homo sapiens and our Myodes glareolus, our positive control, were added also to the prior list. So in fact it was 103 Bornean species plus human and bank vole. We apologize for causing confusion on this and have changed the lines 185-187 accordingly. However we eliminated Homo sapiens from the final results as we could not exclude that this was contamination during collection of leeches.

Structure: In the Methods section, the lab work comes first, in the Results/Discussion, the database construction comes first. Consider selecting a structure that is repeated throughout the paper and use corresponding sub-headers to help the reader track the flow throughout.
-->We apologize for this. The reason was that we wanted to start with the samples and this is followed by the lab procedure and later only the bioinformatics methods (the information about the study site given in the description of the samples is required for this). However, in the results we actually first needed to have the bioinformatics to then present the results of the laboratory work. We realized however that this is confusing and now have changed the order. The new order in the method section is now that the database curation comes first, followed by the lab work, followed by the bioinformatics on read processing and taxonomic assignment.

I was a bit confused - why was COI of interest, and what portion of it was of interest, if it wasn't one of the three loci in the empirical work?
-->COI is one of the standard markers for metabarcoding and particularly widely used for invertebrates. Therefore we initially wanted to use COI as a fourth marker. But it turned out that the primers did not perform reliable during PCR thus we stopped using COI. But as we had the database already built we decided to publish this together with the bioinformatics pipeline, as we expect that other studies might use this additional marker.

Figure 5: Colors are too close for differentiating loci. Consider simply labeling the rows.
-->We changed this figure using a consistent color code for all four markers for all figures. We the colors are discriminable enough we are happy to label the rows if necessary.

Figure 6: Small points make figure difficult to read.
-->Thank you for the comment. We changed that figure to a barplot using a consistent color code for the four genetic markers for all figures. We hope it improved.

-->cited literature:
Ficetola et al.. Replication levels, false presences and the estimation of the presence/absence from eDNA metabarcoding data. Mol Ecol Res. 2014; 15(3): 543-56.
Lahoz-Monfort et al.. Statistical approaches to account for false-positive errors in environmental DNA samples. Mol Ecol Res. 2015; 16: 673-85.
Leray et al.. MIDORI server: a webserver for taxonomic assignment of unknown metazoan mitochondrial-encoded sequences using a curated database. Bioinformatics 2018; 1: 2.
Machida et al.. Metazoan mitochondrial gene sequence reference datasets for taxonomic assignment of environmental samples. Sci Data. 2017; 4: 170027.
Nilsson & Ryberg. Taxonomic Reliability of DNA Sequences in Public Sequence Databases: A Fungal Persepctive. PLoS ONE. 1, 2006; 1.
Rodgers et al.. Carrion fly-derived DNA metabarcoding is an effective tool for mammal surveys: Evidence from a known tropical mammal community. Mol Ecol Res. 2017; 17(6):1-13
Schnell et al.. Tag jumps illuminated–reducing sequence-to-sample misidentifications in metabarcoding studies. Mol Ecol Res. 2015; 15(6): 1289-1303.
Schnell et al.. Debugging diversity - a pan-continental exploration of the potential of terrestrial blood-feeding leeches as a vertebrate monitoring tool. Mol Ecol Res. 2018.

Zepeda-Mendoza et al.. DAMe: a toolkit for the initial processing of datasets with PCR replicates of double-tagged amplicons for DNA metabarcoding analyses. BMC Res Notes 2016; 9.

Reviewer #2:
Despite the advent of high throughput sequencing, incorporating this technology particularly for confident detection of rare species via iDNA/eDNA is still technically challenging. Hence, I applaud the authors for taking the initiave to investigate and tackle this problem.
There are a lot of variables for this sort of work that are not likely to be fully addressed in a single manuscript so I do not fault the authors for not being overly comprehensive in doing benchmarks. However, I feel there are a few additional works to need be done prior to making a recommendation that potentially will change or pave the way forward for such study.

1. Performing an iniail analysis on a mock community - will this be a good start rather than diving straight into to real test?
-->Our manuscript should be viewed in the context that the community has learned a great deal about the errors that arise in metabarcoding (e.g. Schnell et al. 2015, Somervuo et al. 2016, Evans et al. 2017, Racimo et al. 2016) and what can be about those errors (e.g. Zepeda-Mendoza et al. 2016, Somervuo et al. 2017, Rodgers et al. 2017). Thus, while an analysis of a mock community would be useful to show the problems, these problems and their solutions are largely known. The challenge now is to design cost-effective pipelines that use these solutions systematically to mitigate these known sources of error. Our workflow was developed out of the need to analyse over 1000 samples with over 7500 leeches. We initially started published methods but experienced both contamination and/or tag jumping issues. Our use of double twin-tagging solves the tag-jump problem at a relatively low cost (compared to buying, say, a hundred+ pairs of twin-tagged primers). And our curation and Protax pipeline confronts known problems of overconfidence and outright error in taxonomic assignment.
Thus, we think the workflow will be of value for other researchers facing the same problems as we did and we fully expect other researchers to alter and even improve our workflow. To facilitate this, we are using this manuscript to make all our protocols and scripts available. Due to the exponential increase in e/iDNA metabarcoding studies, we think that our manuscript will be of great interest for many researchers.
Nevertheless we have now added additional quantifications on false negative and false positive rates (l. 418-30 & l. 543-561, supplemental figure 1), comparison of our results if we had used a more relaxed acceptance criteria (l. 648-61, table 5) and comparisons to other approaches as far as this was possible.

2. mtDNA markers are of diff fragment size, with that you will have a few variables .
-primer binding efficiency should be tested in-silico for a start, could that be why taxonomic assignment/recovery was affected
- Would it be a good time to design a more suitable primers with in-silico validation for such work? Tetrapod specific but targeting more variable region and with a amplicon size that give better compromise between PCR recovery of high degraded DNA and taxonomic resolution.
--> The reviewer raised important issues and challenges here and we absolutely agree to his concerns. We were also worried on the performance of our primer system and tried to validate their performance using real-time PCR. We tested qPCR conditions on a BioRad plate Cycler and cycling conditions were 5min at 95°C, followed by 50 cycles of 30s at 95°C, 30s at 54°C, and 45s at 72°C with a detection at the end of every cycle and a final extension of 5 min at 72°C. This was followed by a melt curve analysis from 60° to 95°C in 0.5°C steps. As you can see from the example below not all samples performed well and all came very late (> 30 cycles) which means it was impossible to run dilution series to estimate the PCR efficiency. Instead we used LinRegPCR (Ramakers et al., NeuroSci Lett 2003) to calculate the individual reaction efficiencies based on the amplification curve. This might give only a rough estimate of the PCR efficiency but they were all comparably well in a range of 1.8 to 2. However, the variance between the replicates of the tested samples in the qPCR was quite high due to the late fluorescence signal and as the estimated efficiencies were normal we believe that the amount of target DNA in a sample is much more important than the PCR performance of the primers.
At the same time our results clearly showed that amplification success and sequencing quality for the long PCR fragments (12S and Cytb) was much lower than for the short 16S marker (l. 560-67, suppl. table 3). While this reinforces the fact that DNA in leeches is highly degraded, it also means that

designing shorter Cytb and 12S primers might be a useful next step forward. We highlighted this in the manuscript in line 569-74, which reads: "...Despite the greater taxonomic resolution of the longer 12S and CytB fragments, our poorer amplification and sequencing results for these longer fragments emphasize that e/iDNA studies should generally focus on short PCR fragments to increase the likelihood of positive amplifications of the degraded target DNA. In the case of mammal-focussed e/iDNA studies, developing a shorter (100 bp) CytB fragment would likely be very useful. ..."

3. the cytb gene seem to be the most promising given its high representation for mammal, so that could be a good candidate for primer design to generate a smaller amplicon than its current primer pairs.
--> Yes, absolutely correct and we are certain that is could improve the workflow. But it is a challenging task to find primers that fulfill the conditions and amplify over such a large number of taxa. At this point we were unable to test additional primers, but hope that with the publication of this manuscript, other groups will be inspired to work on this. However, this does not affect the current workflow and one could also use more taxon-specific primers.

4. Authors should show the number of reads generated for each sample and also the number of reads per marker because sequencing depth obviously will have an effect as well on the detection sensitivity.
--> One of the outputs of the pipeline is a tab delimited txt-file reporting on the read numbers for each marker per sample and that were processed in the various steps of the read-preprocessing.sh script. This includes the raw number of reads, merging of R1/R2 reads, primer clipping and trimming of reads, quality filtering and dereplication. We added a note on this in line 363-65. Furthermore we summarized these results for all eight sequencing runs in the supplemental table 3. We also summarized the number reads per sample that entered the taxonomic assignment for each of the eight sequencing runs (supplemental table 4). In the main body of the text we refer to these results in lines 566-69 and lines 665-68.

5. Seems like authors used Ampli-Gold Taq instead of a high fidelity polymerase (such as but not limited to KAPA HIFI, Q5 polymerase)for their amplification. higher taq polymerase error rate coupled with the use of dereplication without error-correction is likely going to generate way more unique reads. So will using a proof reading high fidelity polymerase followed by chimera removal and error correction bioinformatic tool e.g. UNOISE3 be useful in eliminating spurious product from high amplification cycle?
-->This is true; a high-fidelity tag polymerase would decrease the noise signal around the real sequence. But it is a question if this lower noise signal affects the final assignment much in the end and if it is worth the additional costs, given that we are already using replication (and usearch filters) to filter out erroneous amplicons. Specifically, we don't expect chimeric sequences to be a problem because it is very unlikely that they result in a believable and high-confidence taxonomic assignment, given the very low probability that the same chimeric sequence would occur in two replicates of the same sample in the same way and thus it would never be accepted as a true positive.
The price for high fidelity taq polymerase is about twice the one for hot start polymerase, which goes against one of our goals, which is to increase cost-efficiency for large-scale biodiversity screenings. With over 1000 leech samples we have over 24,000 PCR reactions. Doubling the tag costs would certainly make it very difficult to implement such large scale projects. We have worked with high fidelity Taq in other projects and recognized the advantages, but for this work, and given our use of PCR and marker replicates, we do not believe that our assignment results would improve in a way which would justify these additional costs.

6. The read2 from MiSeq 600 cycle kit is known to be very poor in quality towards the end and that will affect overlap for the bigger fragment, authors might want to look into this and do proper trimming.
--> Yes, we are aware of this problem and addressed these issues in lines 356-365: "...In all cases, amplicons were short enough to expect paired reads to overlap. For libraries with more than 1000 reads pairs were merged with usearch (-fastq_mergepairs; [47; 48]), and only successfully merged pairs were retained. For libraries with more than 500 merged pairs the primer sequences were trimmed away with cutadapt [49], and only successfully trimmed reads at least 90% of expected amplicon length were passed to a quality filtering step with usearch (-fastq_filter). Lastly, reads were dereplicated with

usearch (-derep_fulllength), and singletons were discarded. The number of replicates that each unique sequence represented was also added to the read header at this step (option -sizeout). The number of reads processed at each step for each sample are reported in a standard tab delimited txt-file. ...", as well as in lines 566-69: "...Also the read losses due to trimming and quality filtering were significantly lower for the 16S sequencing runs (1.3% and 5.3% in average, Supplemental Table 3) compared to the sequencing runs for the longer fragments of 12S and CytB (65.3% and 44.3% in average, Supplemental Table 3). ..."

Because low quality reads were excluded with usearch we do not believe that read quality causes severe problems in our study. However, Taberlet et al. 2007 demonstrated that the presence of a homopolymer in a metabarcoding sequence leads to a systematic decrease of read quality. Thus average quality can change consistently from one species to another and they argue that quality based trimming and filtering might bias diversity estimates in metabarcoding samples to an unknown extant (see in Bonin et al. 2018).

7. From 6, it will be good to how # of reads generated then # of reads lost after each QC/overlap/binning.

--> Thank you for the comment, for trimming and merging of reads we use already well-established standard tools for post-run processing of Illumina sequencing reads (see our answer to your point 6). As we already said we have added the supplemental tables 3 reporting on the read losses during read processing. However, given length limits, we think it is much more important to focus on how we avoided contamination and false positives and ended up with a species list that stakeholders can trust and base decisions on. That we hope to find your agreement to leave those tables in the supplement as they refer more to sequencing quality than metabarcoding.

8. Can the authors also normalize the final reads prior to comparing their sensitivity/efficiency in recovering species? My concern is that some species was not recovered because of low sequencing depth in one of the replicates.

--> Regarding the sensitivity in recovering species we added some simulation data on false negative rates for varying detection probabilities (Supplemental Figure 1) and added another acceptance criteria (l. 418-30) more discussion on this topic in lines 543-574.

We understand the reviewers concern on non-detection due to missing sequencing depth. We analysed 242 samples and only pooled the PCR with a positive PCR equimolar together, so each sample in the sequenced library should contribute with the same amount of DNA to the pool. Even if all 242 samples would have been positive, if we assume 20 million reads from an Illumina V3 kit and if we lose 30% to spiked in PhiX control, we would end up with 14,000,000 reads/242 samples = 57,851 reads/sample. Thus we are less worried about sequencing depth for our study.

However, to address this concern we provided now an additional table on the number of reads per sample, as requested in point 4 (Supplemental Table 4). Although we understand the reviewer's concern on imperfect detection due to low read numbers per sample, it is unfortunately not feasible to adjust sequencing depth to individual samples of unknown content in a high-throughput workflow. The risk of an imperfect detection and such false negatives is always given in metabarcoding studies. However, our lowest median read number per sample per sequencing run was 8218 reads, which we think should be sufficient. Furthermore read numbers are largely influenced by PCR stochasticity and we believe that reproducibility is the more reliable factor for species detection than read numbers.

9. What sort of sequening depth would be recommended for detection of low abudance/rare species? What if increasing sequenicng depth to 1 million reads allow recovery ? It will be good to have a spike-in DNA as control to check that out coupled with qPCR + tqman probe to compare sensitivity. The last thing we want is making claims on species presence/absence based on a specific sequencing depth and without complementary data to support. Absence of evidence sometimes is not evidence of absence and this is really one of the biggest challenges in using NGS for such work - how deep is deep enough?

--> The main consideration for not using a spike-in is that such DNA is high quality and thus likely to outcompete our degraded target DNA in the PCR reaction, leading to even lower detection probabilities. DNA degradation varies to such a great extent in the leech samples that any spike-in experiment even if we use degraded ancient DNA could never represent the complexity of a bulk leech sample. We therefore decided to accept this trade off and accept that some false negative will occur in the dataset.

As outlined above such false negatives are incorporated in the detection probability estimation of the hierarchical occupancy models.
Increasing sequencing depth is another method for increasing detection, but sticking to the example with 1 million reads, we could sequence then 20 PCRs in one MiSeq run with 20 million reads. Even if only one third of our over 24,000 PCRs contains target DNA this would mean we would have to run 400 sequencing runs and one 600 cycle V3 kit is about 1500,- €...
We very much agree that there are options to improve detectability, but for large scale screening we are soon facing severe trade-offs in terms of costs and time.

10. Regarding the 103 mammal species. Are the authors assuming that their species designation is correct? And will it be correct to assume that leech will feed on all 103 mammal species in general? Also mtDNA database of the actual species sequenced from the location will be good. Borneo is an isolated island, there might be potentially new species despite morphological similarity to known species.
--> The 103 species are currently accepted species. That said, there will certainly be cryptic species/subspecies on Borneo. Our team has been involved in numerous phylogenetic studies in the region and is aware of these difficulties. You are right that genetic reference from the particular research area would be desirable to reflect geographic difference. But we simply have to work with what we have and hope that the intraspecific variance is still lower than the interspecific one.
In the course of this study, we helped to build a mitogenomic database of Southeast Asian mammals and we published in 2017 in Gigascience 57 additional mitogenomes. In addition, as a part of this manuscript we have released additional mitogenomes and mtDNA sequences. We realize the need for additional mitogenome sequencing and barcoding effort (as we say in l. 503-505 of the manuscript). However, obtaining samples, particularly of rare and endemic species is extremely difficult (both in terms of time and permission), and thus there will be always some questions in respect to the actual species designation. As said above the 103 species were selected based on over 10 years of field research in Sabah Malaysia and together with a number of long-term scientists working in Sabah. It is of course unknown if leeches feed on all of these 103 species (we are the first study looking at leeches from our sites), but it is important to include all potential species in the list to ensure that PROTAX does not assign a read to a sister species, just in consequence that the “true” species was not included.

11. "As the costs of HTS decrease, we expect that such gap-filling will increasingly shift towards whole mitochondrial genomes [36], reducing the effect of marker choice on detection likelihood " - I think authors need to tone down on this. Even with the Novaseq6000, the depth required for sequencing just to recover whole mtDNA will be hard because first, it will be the leech DNA/genome that will dominate the data. Second, from the remaining non-host reads, the chance of mtDNA recovery will depend on how many diff species of mammals the leech has attached to previously. More importantly, blood in genera is relatively low in mtDNA unlike muscle.
--> Sorry for the misunderstanding here. We did not mean that leeches could be used to construct mammalian mitogenomes. We meant that with the decreasing costs in HTS, there will hopefully be additional reference mitogenomes in the future including more species and covering larger geographical areas. We have rephrased this section:
l. 505-08: “...As the costs of HTS decrease, we expect that such gap-filling will increasingly shift towards sequencing of whole mitochondrial genomes of specimen obtained from museum collections, trapping campaigns etc. [34], reducing the effect of marker choice on detection likelihood.....”

12. I also couldn't find the term "SRA" in the manuscript so I wonder if the data has been submitted to NCBI database.
Fastq files from the sequencing runs for each leech sample are uploaded to the European Nucleotide Archive ENA and can be found via the study accession number ERP109441.
https://www.ebi.ac.uk/ena/data/view/ERP109441

--> cited literature:
Bonin et al.. Environmental DNA: For Biodiversity Research and Monitoring. 1st ed. Oxford University Press; 2018.
Evans et al. Fish community assessment with eDNA metabarcoding: effects of sampling design and bioinformatic filtering. Can J Fish Aquat Sci. 2017; 74(9):, 1362-74.

Racimo et al. Joint estimation of contamination, error and demography for nuclear DNA from ancient humans. PLoS Genet. 2016; 12(4): e1005972.
Rodgers et al.. Carrion fly-derived DNA metabarcoding is an effective tool for mammal surveys: Evidence from a known tropical mammal community. Mol Ecol Res. 2017; 17(6):1-13
Schnell et al.. Tag jumps illuminated–reducing sequence-to-sample misidentifications in metabarcoding studies. Mol Ecol Res. 2015; 15(6): 1289-1303.
Schnell et al.. Debugging diversity - a pan-continental exploration of the potential of terrestrial blood-feeding leeches as a vertebrate monitoring tool. Mol Ecol Res. 2018.
Somervuo P et al.. Unbiased probabilistic taxonomic classification for DNA barcoding. Bioinformatics. 2016; 32(19): 2920-7.
Somervuo et al.. Quantifying uncertainty of taxonomic placement in DNA barcoding and metabarcoding. Methods Ecol Evol. 2017; 8(4): 398-407.
Zepeda-Mendoza et al.. DAMe: a toolkit for the initial processing of datasets with PCR replicates of double-tagged amplicons for DNA metabarcoding analyses. BMC Res Notes 2016; 9.

Close
