# Supplementary material for: An efficient and robust laboratory workflow and tetrapod database for larger scale environmental DNA studies
Source: Gigascience. 2019 Apr 13;8(4):giz029. doi: 10.1093/gigascience/giz029 (PMC6461710; doi:10.1093/gigascience/giz029)
Supplement: Response_to_Reviewer_Comments_Revision_1.pdf [file giz029_response_to_reviewer_comments_revision_1.pdf]

Author's Response To Reviewer Comments

Close

Dear Hongling Zhou,

First we would like to thank both reviewers for their positive feedback and the editor for the potential interest to publish our paper in GigaScience. Below we provide a detailed response to the remaining comments and suggestions by the reviewers. These certainly helped to improve the manuscript further and we thank the reviewers for their valuable comments.
On behalf of our co-authors,

Jan Axtner & Andreas Wilting

Reviewer reports:

Reviewer #1: Thank you for taking the time to address all comments in detail. The corrections I think have improved the clarity of the piece, and I feel convinced where you explained where I misunderstood. One possible reference to consider (given a comment about the availability of models to account for errors at multiple levels):

Guillera-Arroita. 2017. Dealing with false-positive and false-negative errors about species occurrence at multiple levels. Methods in Ecology and Evolution. https://doi.org/10.1111/2041-210X.12743

Thank you for the positive feedback and the interesting article. So far we were not aware of it, but as it fit's so perfectly to our topic, thus we now refer to it in line 561

Reviewer #2: I am overall satisfied with the responses provided by the authors. In general, it is quite unlikely nowadays that there will be a consensus for the "right/best" way forward. It is always subject to practicality/funding. If i were to conduct my own amplicon seq project, will I follow this protocol to the dots - no. However, the bioinformatics scripts and data generated will be useful for better experimental design in the future. Furthermore, even if a method is robust, lab competency / human error (mislabeling, mixing the wrong index etc) is still going to be an issue.

Reviewer 1 raised the concern of similar tag1 being used repeatedly for multiple samples. I wonder if instead of using "Twin" tag, having a different tag1 combination (non-Twin tag?!) will be helpful (obviously for discussion). In other words, the forward and reverse primer combination in the 1st PCR round can be Tag1a for forward Tag1b for reverse. This is somewhat similar to dual indexing in Illumina but you're doing it at the initial stage and should will expand the 24 sample limitation for the tag1 based on my current understanding the twin-tag but happy to be proven wrong. With the increasing problem of index hoping particularly with the patterned flowcell for the Novaseq and Iseq (relevant to amplicon seq) , this should be useful and worth looking into.

See https://www.biorxiv.org/content/early/2017/10/19/205799

We agree that are other factors like lab skills or human errors that are an important issue and in fact our whole laboratory procedure is designed to minimize human-related errors. The whole workflow is designed to allow a high-throughput of samples in a maximum standardized way, i.e. sample aliquots are arranged already in eight-well stripes for the use of eight-channel pipets in order to minimize the risk of pipetting the wrong sample into the wrong well between the different replicates. That is also one of the reasons why we do not start mixing the tag1 combinations and re-use the 24 tags for each PCR plate. Our forward and reverse primers are already pre-mixed in an eight-well stripe and we use the same pipetting scheme with an eight-channel pipet for every 96-well PCR plate. If we would start using different tag combinations for each PCR plate we would have a much higher risk of pipetting errors

mixing the wrong indices (handling 48 tubes is much more error prone than handling just three 8-well stripes).
In addition to this rather practical lab-work related reason we highlighted (Line: 602 to 605) that it is still very unlikely that the repeated use of tags for multiple samples causes accepted false positives in the end, as the final acceptance is not based on single occurrence but on repeated occurrence in independent replicates. We fully agree that the use of non-matching tags (e.g. A/B) would increase the number of samples that could be analysed in one sequencing run. But at the same time it would make it much harder to identify contaminations or tag jumps as we discuss in line 575 to 599. Contaminations of a PCR with another differently labelled PCR product would increase the number of chimeras in your PCR which would remain undetected if you would also use non-matching tag combination. The same holds true for tag-jumps, which are an issue in Illumina sequencing (see Schnell et al. 2015) and where we could demonstrate that our PCR libraries reduce the read-losses compared to adapter-ligation techniques (lines 585-594).Particular for the last reasons we favoured to use only twin-tag combinations.

We also thank the reviewer for the interesting paper, which also used quadruple-indexed libraries. We do however not see the application of RAD sequencing to identify invertebrate-derived DNA of unknown origin. Generally RAD sequencing requires high molecular weight genomic DNA. Our samples have a mixed pools of genomic and mitochondrial DNA from different organisms and our target DNA is often highly degraded, of poor quality and of low quantities. In addition we have the presence of high amounts of leech DNA. Therefore we currently do not see an application of this sequencing method.

"Also the read losses due to trimming and quality filtering were significantly lower for the 16S sequencing runs (1.3% and 5.3% in average, Supplemental Table 3) compared to the sequencing runs for the longer fragments of 12S and CytB (65.3% and 44.3% in average, Supplemental Table 3)."

The Usearch read overlapping pipeline is sensitive to number of mismatches in alignment. The Read2 in MiSeq 600 cycles run is particularly notoriously for being low quality towards the end of the run. Try trimming both R1 and R2 to 250 bp (length trimming) and redo the overlap and read loss calculation.

Thank you, for this valuable advice. We tested it for one of our 12S runs and compared results. As you suggested we trimmed the reads to 250 base pairs adjusted the -fastq_minovlen parameter for usearch from 50bp to just 25bp as we would expect to have a smaller overlap of the trimmed reads. In fact we obtained more read after merging (13,129,505 vs. 13,388,933). However, most of those reads were lost again after filtering so that our original settings produced in fact the most reads I the end (4,694,624 vs. 4,227,346). Thus we think it is reasonable to stick to the current settings in the pipeline.
Results original pipeline:
raw reads: 13,766,169
merging: 13,129,505
clipping: 6,498,738
filtering: 4,694,624
Trimmed reads (trimm 250bp, overlap 25bp):
raw reads: 13,766,169
merging: 13,388,933
clipping: 6,684,766
filtering: 4,227,346

"All three markers were amplified simultaneously for each batch of samples in a single PCR plate".

In different individual well?
Sorry for the misunderstanding, we did not do multiplex-PCR and amplified in individual wells. We added this to the sentence in lines 324-325:
"… All three markers were amplified simultaneously in individual wells for each batch of samples in a single PCR plate. …"

Because of different amplicon lengths and therefore different binding affinities to the flow cell
Also due to clustering efficiency . smaller fragment = easier to amplify
We agree, also due to DNA degradation we had higher amplification success for the shortest fragment

(see lines 562 – 566). As we say in lines 337-340 "…Because of different amplicon lengths and therefore different binding affinities to the flow cell, 12S and CytB products were combined in a single library, whereas positive 16S products were always combined in a separate library. …" and these libraries were sequenced independently. To make this clearer we added a second sentence (line 340): "… 12S/CytB libraries were sequenced independently from 16S libraries…."

Close
