# Supplementary material for: An efficient and robust laboratory workflow and tetrapod database for larger scale environmental DNA studies
Source: Gigascience. 2019 Apr 13;8(4):giz029. doi: 10.1093/gigascience/giz029 (PMC6461710; doi:10.1093/gigascience/giz029)
Supplement: Reviewer_1_Report_Original_Submission -- Taylor Wilcox — 7/7/2018 Reviewed [file giz029_reviewer_1_report_original_submission.pdf]

Reviewer Report

Title: An efficient and robust laboratory workflow and tetrapod database for larger scale eDNA studies

Version: Original Submission **Date:** 7/7/2018

Reviewer name: Taylor Wilcox

Reviewer Comments to Author:

In this manuscript Axtner et al. 1) used metabarcoding to assess mammal diversity from leeches and 2) developed a pipeline to build a curated reference database for taxonomic assignment. An emphasis of the metabarcoding was replication, including replication at the extraction, amplification, and locus levels. This work is of interest because 1) more robust inferences from metabarcoding may be possible by looking for concordance across replicates at multiple levels in the analysis process and 2) accurate taxonomic assignment is often limited by database accuracy and completeness. Thus, the manuscript is likely of interest not only to other iDNA users, but to metabarcoding users generally (e.g., eDNA, diet analysis, plant-pollinator interactions). I have three major comments and some more minor comments below. Generally, I think the authors could build a stronger case for their extensive lab and database work by taking a more quantitative approach to assessing success. Also, it was not very clear throughout the manuscript where to access the raw sequence data (FASTQ files from bcl2fastq probably fine), taxonomic assignments, scripts, etc. Places like Line 404 should include info on where to get the script.
Major comment 1:
Although I appreciate the value of replication, I think the manuscript would benefit from a quantitative assessment of the effect that replication had on inference accuracy. In the title the authors say this workflow is "improved". How can you demonstrate this?
One of the main points of this manuscript is the value of technical replication to reduce false positive errors. Thus, each sample has replicate extractions, each extraction replicate loci, and each loci replicate PCR. As described, this is probably intuitively of value to folks who work with low-DNA applications. The idea being that something that is real should be something that you can detect repeatedly. What's lacking to me is a quantitative justification or assessment of these replication levels and the thresholds assigned to them for interpretation. Can you provide a quantitative answer to these questions?
- Does the rule of detection in 2/2 extraction replicates reduce estimated false positive rates compared to only 1/2 replicates?
- Is only requiring detection in 1/2 PCR replicates per marker sufficient, or would requiring 2/2 PCR replicates reduce the estimate false positive rate?
- What is the effect of the used 10 reads threshold versus other thresholds (e.g., 5, 50, 100) on the estimate false positive rate? How did you determine that this threshold could be dropped if the taxon was detected with >1 locus?
I'd suggest that if you can't answer these with empirical data or a reasonable probability model, then you can't really argue that your replication approaches were any "better" than any other given approach.

There's another potential issue here, which is not discussed, which is the false negative rate. By requiring replicated detections, you drive down your false positive rate, but drive up your false negative rate. If the false negative rate per extraction/PCR is very low, maybe this doesn't matter much, but it could be quite large. For example, there is a recent discussion in the literature related to this idea with a focus on PCR replicates:
Ficetola et al. 2015. Replication levels, false presences and the estimation of the presence/absence from eDNA metabarcoding data. Molecular Ecology Resources. https://doi.org/10.1111/1755-0998.12338
Lahoz-Monfort et al. 2016. Statistical approaches to account for falseâ€positive errors in environmental DNA samples. Molecular Ecology Resources
Ficetola et al. 2016. How to limit false positives in environmental DNA andmetabarcoding? Molecular Ecology Resources
Major comment 2:
For the database, I would have liked to see the authors show that their database curation decreased the false positive rate or in some other way increased the accuracy of their inferences. Is this curated database approach necessary to apply PROTAX, or could the original sequences with redundancy and mislabeling have been used as well? If this pipeline is a major product in the manuscript (as the Abstract suggests) how can you quantitatively demonstrate to the reader that it is worth using?
Major comment 3:
It took be a bit of time to grasp the potential value of doing two rounds of PCR with sets of doubly-indexed primers. It wasn't completely clear to me that different combinations of first round and second round indices were used to increase the level of multiplexing. I think the proposed benefits of this approach could use a bit more explanation, including some caveats.
1) This approach *does* potentially reduce contamination risk as compared to two-round PCR metabarcoding protocols where the first round of amplification is done with tailed, unlabeled primers (or when adaptors are ligated). However, if you re-use first-round indices for multiple libraries, you will generate PCR products with the *same* labels in the first round of PCR. Perhaps this issue may be somewhat mitigated by preparing libraries in batches so that no libraries with the same indexing primers are prepared simultaneously. This caveat probably also applies to the discussion in Line 545. If you re-use first-round indices for multiple projects, PCR products from one study can show up in another. Re-using indexing primers seems highly likley given the expense of long, purified oligos - it doesn't seem affordable to use the first-round primers for only a single library prep. The risk *is* probably lower (because 1/25 libraries have that index, as opposed to 25/25 when unlabeled), but it's not completely unambiguous.
2) I am not convinced by the current description that this approach allows removal of chimeric sequences. However, my uncertainty may largely stem from my confusion about what you mean by "chimeric sequence". My understanding is that a "chimera" or "chimeric sequence" is a single molecule that came from two different transcripts. For example, an incompletely-extended PCR product anneals to and extends on a similar, but different template from the original. Resulting reads reflect a composite sequence formed by PCR.
Such a "chimeric sequence" that forms *within* a single library cannot be detected based on paired index sequences. All of the PCR products have the same index sequences on each both ends. Thus, a chimera formed between species A and species B is indistinguishable from a PCR product from species A

based on the index sequences alone. I don't think that this is the type of "chimeric sequence" that you're worried about, but it can affect taxonomic assignment (perhaps the authors can explain the sensitivity of PROTAX to these types of errors).

The other type of chimeric sequence that is more problematic is when a molecule has an index for library #1 on one end and an index for library #2 on the other. If you have double-indexed libraries with only one P5/P7 combination per library, then you can remove reads from these PCR products. I think this is the type of chimeric sequence the authors are concerned about? In which case, I'm a bit confused about two points: First, how is it possible to form physical chimeras if each library is amplified by itself and pooled only for sequencing? My understanding is that incorrectly-tagged reads from this protocol come from sequencing errors on the flow cell, rather than being due to the presence of chimeric molecules. Maybe carefully distinguishing between sequences (molecules) and reads (MiSeq output) would help me to track with you. Second why would two-rounds of indexing be better at detecting these types of errors than a single round? Can you show me with a cartoon on Figure 2?

More minor comments:

Line 187: Later you report a range of values for percent reads from Mammalia, so these must be 58 individually-indexed libraries? How were the libraries prepared (e.g., shearing, indexing, how was quantity assessed for pooling)? Bioinformatics for these unclear. We assume there was some quality filtering steps and rules associated with assignment? If your goal is to assess enrichment success with PCR, would you want to use a comparable pipeline across this experiment and the amplified libraries?

Line 191: Would be helpful to justify these primer sets a bit. Why would we expect them to be suitable for this application?

Line 253: Spell out acronym on first use.

Line 266: If there are 7 previously unpublished mitochondrial genomes, why are there 13 Accession Numbers here? Are these GenBank Accession Numbers? Entering a few of them into GenBank did not result in any sequences.

Line 485: Not sure which 554 species this is. I thought we were talking about the 103 species expected in the sampling area.

It was not super clear - when a locus did not amplify (checked via gel electrophoresis), did you drop those PCR products from the library pool? Were all amplicons pooled equimolarly (you say "samples" here)?

How did you make the list of 103 mammal species known to be present? Why is Homo sapien not in this list?

Structure: In the Methods section, the lab work comes first, in the Results/Discussion, the database construction comes first. Consider selecting a structure that is repeated throughout the paper and use corresponding sub-headers to help the reader track the flow throughout.

I was a bit confused - why was COI of interest, and what portion of it was of interest, if it wasn't one of the three loci in the empirical work?

Figure 5: Colors are too close for differentiating loci. Consider simply labeling the rows.

Figure 6: Small points make figure difficult to read.

Level of Interest

Please indicate how interesting you found the manuscript: Choose an item.

Quality of Written English

Please indicate the quality of language in the manuscript: Choose an item.

Declaration of Competing Interests

Please complete a declaration of competing interests, considering the following questions:

- Have you in the past five years received reimbursements, fees, funding, or salary from an organisation that may in any way gain or lose financially from the publication of this manuscript, either now or in the future?
- Do you hold any stocks or shares in an organisation that may in any way gain or lose financially from the publication of this manuscript, either now or in the future?
- Do you hold or are you currently applying for any patents relating to the content of the manuscript?
- Have you received reimbursements, fees, funding, or salary from an organization that holds or has applied for patents relating to the content of the manuscript?
- Do you have any other financial competing interests?
- Do you have any non-financial competing interests in relation to this paper?

If you can answer no to all of the above, write 'I declare that I have no competing interests' below. If your reply is yes to any, please give details below.

I declare that I have no competing interests

I agree to the open peer review policy of the journal. I understand that my name will be included on my report to the authors and, if the manuscript is accepted for publication, my named report including any attachments I upload will be posted on the website along with the authors' responses. I agree for my report to be made available under an Open Access Creative Commons CC-BY license (http://creativecommons.org/licenses/by/4.0/). I understand that any comments which I do not wish to be included in my named report can be included as confidential comments to the editors, which will not be published.

I agree to the open peer review policy of the journal

To further support our reviewers, we have joined with Publons, where you can gain additional credit to further highlight your hard work (see: https://publons.com/journal/530/gigascience). On publication of this paper, your review will be automatically added to Publons, you can then choose whether or not to claim your Publons credit. I understand this statement.

Yes Choose an item.
