# Supplementary material for: An efficient and robust laboratory workflow and tetrapod database for larger scale environmental DNA studies
Source: Gigascience. 2019 Apr 13;8(4):giz029. doi: 10.1093/gigascience/giz029 (PMC6461710; doi:10.1093/gigascience/giz029)
Supplement: Reviewer_2_Report_Original_Submission -- Han Ming Gan — 7/22/2018 Reviewed [file giz029_reviewer_2_report_original_submission.pdf]

Reviewer Report

Title: An efficient and robust laboratory workflow and tetrapod database for larger scale eDNA studies

Version: Original Submission **Date:** 7/22/2018

Reviewer name: Han Ming Gan

Reviewer Comments to Author:

Despite the advent of high throughput sequencing, incorporating this technology particularly for confident detection of rare species via iDNA/eDNA is still technically challenging. Hence, I applaud the authors for taking the initiave to investigate and tackle this problem.
There are a lot of variables for this sort of work that are not likely to be fully addressed in a single manuscript so I do not fault the authors for not being overly comprehensive in doing benchmarks. However, I feel there are a few additional works to need be done prior to making a recommendation that potentially will change or pave the way forward for such study.
1. Performing an iniail analysis on a mock community - will this be a good start rather than diving straight into to real test ?
2. mtDNA markers are of diff fragment size, with that you will have a few variables .
-primer binding efficiency should be tested in-silico for a start, could that be why taxonomic assignment/recovery was affected
- Would it be a good time to design a more suitable primers with in-silico validation for such work? Tetrapod specific but targeting more variable region and with a amplicon size that give better compromise between PCR recovery of high degraded DNA and taxonomic resolution
3. the cytb gene seem to be the most promising given its high representation for mammal, so that could be a good candidate for primer design to generate a smaller amplicon than its current primer pairs.
4. Authors should show the number of reads generated for each sample and also the number of reads per marker because sequencing depth obviously will have an effect as well on the detection sensitivity.
5. Seems like authors used Ampli-Gold Taq instead of a high fidelity polymerase (such as but not limited to KAPA HIFI, Q5 polymerase)for their amplification. higher taq polymerase error rate coupled with the use of dereplication without error-correction is likely going to generate way more unique reads. So will using a proof reading high fidelity polymerase followed by chimera removal and error correction bioinformatic tool e.g. UNOISE3 be useful in eliminating spurious product from high amplification cycle?
6. The read2 from MiSeq 600 cycle kit is known to be very poor in quality towards the end and that will affect overlap for the bigger fragment, authors might want to look into this and do proper trimming.
7. From 6, it will be good to how # of reads generated then # of reads lost after each QC/overlap/binning
8. Can the authors also normalize the final reads prior to comparing their sensitivity/efficiency in recovering species? My concern is that some species was not recovered because of low sequencing depth in one of the replicates.
9. What sort of sequening depth would be recommended for detection of low abudance/rare species? What if increasing sequenicng depth to 1 million reads allow recovery ? It will be good to have a spike-in

DNA as control to check that out coupled with qPCR + tqman probe to compare sensitivity. The last thing we want is making claims on species presence/absence based on a specific sequencing depth and without complementary data to support. Absence of evidence sometimes is not evidence of absence and this is really one of the biggest challenges in using NGS for such work - how deep is deep enough?
10. Regarding the 103 mammal species. Are the authors assuming that their species designation is correct ? And will it be correct to assume that leech will feed on all 103 mammal species in general? Also mtDNA database of the actual species sequenced from the location will be good. Borneo is an isolated island, there might be potentially new species despite morphological similarity to known species.
11. "As the costs of HTS decrease, we
expect that such gap-filling will increasingly shift towards whole mitochondrial genomes
[36], reducing the effect of marker choice on detection likelihood " - I think authors need to tone down on this . Even with the Novaseq6000, the depth required for sequencing just to recover whole mtDNA will be hard because first, it will be the leech DNA/genome that will dominate the data. Second, from the remaining non-host reads, the chance of mtDNA recovery will depend on how many diff species of mammals the leech has attached to previously. More importantly, blood in genera is relatively low in mtDNA unlike muscle.
12. I also couldn't find the term "SRA" in the manuscript so I wonder if the data has been submitted to NCBI database.

Level of Interest

Please indicate how interesting you found the manuscript: Choose an item.

Quality of Written English

Please indicate the quality of language in the manuscript: Choose an item.

Declaration of Competing Interests

Please complete a declaration of competing interests, considering the following questions:

- Have you in the past five years received reimbursements, fees, funding, or salary from an organisation that may in any way gain or lose financially from the publication of this manuscript, either now or in the future?
- Do you hold any stocks or shares in an organisation that may in any way gain or lose financially from the publication of this manuscript, either now or in the future?
- Do you hold or are you currently applying for any patents relating to the content of the manuscript?
- Have you received reimbursements, fees, funding, or salary from an organization that holds or has applied for patents relating to the content of the manuscript?
- Do you have any other financial competing interests?
- Do you have any non-financial competing interests in relation to this paper?

If you can answer no to all of the above, write 'I declare that I have no competing interests' below. If your reply is yes to any, please give details below.

I declare that I have no competing interests

I agree to the open peer review policy of the journal. I understand that my name will be included on my report to the authors and, if the manuscript is accepted for publication, my named report including any attachments I upload will be posted on the website along with the authors' responses. I agree for my report to be made available under an Open Access Creative Commons CC-BY license (http://creativecommons.org/licenses/by/4.0/). I understand that any comments which I do not wish to be included in my named report can be included as confidential comments to the editors, which will not be published.

I agree to the open peer review policy of the journal

To further support our reviewers, we have joined with Publons, where you can gain additional credit to further highlight your hard work (see: https://publons.com/journal/530/gigascience). On publication of this paper, your review will be automatically added to Publons, you can then choose whether or not to claim your Publons credit. I understand this statement.

Yes Choose an item.
