# Supplementary material for: An efficient and robust laboratory workflow and tetrapod database for larger scale environmental DNA studies
Source: Gigascience. 2019 Apr 13;8(4):giz029. doi: 10.1093/gigascience/giz029 (PMC6461710; doi:10.1093/gigascience/giz029)
Supplement: Reviewer_2_Report_Revision_1 -- Han Ming Gan — 11/12/2018 Reviewed [file giz029_reviewer_2_report_revision_1.pdf]

Reviewer Report

Title: An efficient and robust laboratory workflow and tetrapod database for larger scale eDNA studies

Version: Revision 1 **Date:** 11/12/2018

Reviewer name: Han Ming Gan

Reviewer Comments to Author:

I am overall satisfied with the responses provided by the authors. In general, it is quite unlikely nowadays that there will be a consensus for the "right/best" way forward. It is always subject to practicality/funding. If i were to conduct my own amplicon seq project, will I follow this protocol to the dots - no. However, the bioinformatics scripts and data generated will be useful for better experimental design in the future. Furthermore, even if a method is robust, lab competency / human error (mislabeling, mixing the wrong index etc) is still going to be an issue.

Reviewer 1 raised the concern of similar tag1 being used repeatedly for multiple samples. I wonder if instead of using "Twin" tag, having a different tag1 combination (non-Twin tag?!) will be helpful (obviously for discussion). In other words, the forward and reverse primer combination in the 1st PCR round can be Tag1a for forward Tag1b for reverse. This is somewhat similar to dual indexing in Illumina but you're doing it at the initial stage and should will expand the 24 sample limitation for the tag1 based on my current understanding the twin-tag but happy to be proven wrong. With the increasing problem of index hoping particularly with the patterned flowcell for the Novaseq and Iseq (relevant to amplicon seq) , this should be useful and worth looking into.
See https://www.biorxiv.org/content/early/2017/10/19/205799

"Also the read losses due to trimming and quality filtering were significantly lower for the 16S sequencing runs (1.3% and 5.3% in average,Supplemental Table 3) compared to the sequencing runs for the longer fragments of 12S and CytB (65.3% and 44.3% in average, Supplemental Table 3)."
The Usearch read overlapping pipeline is sensitive to number of mismatches in alignment. THe Read2 in MiSeq 600 cycles run is particularly notoriously for being low quality towards the end of the run. Try trimming both R1 and R2 to 250 bp (length trimming) and redo the overlap and read loss calculation.

"All three markers were amplified simultaneously for each batch of samples in a single PCR plate".
In different individual well?

Because of different amplicon lengths and therefore different binding affinities to the flow cell
Also due to clustering efficiency . smaller fragment = easier to amplify
------

Level of Interest

Please indicate how interesting you found the manuscript: Choose an item.

Quality of Written English

Please indicate the quality of language in the manuscript: Choose an item.

Declaration of Competing Interests

Please complete a declaration of competing interests, considering the following questions:

- Have you in the past five years received reimbursements, fees, funding, or salary from an organisation that may in any way gain or lose financially from the publication of this manuscript, either now or in the future?
- Do you hold any stocks or shares in an organisation that may in any way gain or lose financially from the publication of this manuscript, either now or in the future?
- Do you hold or are you currently applying for any patents relating to the content of the manuscript?
- Have you received reimbursements, fees, funding, or salary from an organization that holds or has applied for patents relating to the content of the manuscript?
- Do you have any other financial competing interests?
- Do you have any non-financial competing interests in relation to this paper?

If you can answer no to all of the above, write 'I declare that I have no competing interests' below. If your reply is yes to any, please give details below.

No competing interests

I agree to the open peer review policy of the journal. I understand that my name will be included on my report to the authors and, if the manuscript is accepted for publication, my named report including any attachments I upload will be posted on the website along with the authors' responses. I agree for my report to be made available under an Open Access Creative Commons CC-BY license (http://creativecommons.org/licenses/by/4.0/). I understand that any comments which I do not wish to be included in my named report can be included as confidential comments to the editors, which will not be published.

I agree to the open peer review policy of the journal

To further support our reviewers, we have joined with Publons, where you can gain additional credit to further highlight your hard work (see: https://publons.com/journal/530/gigascience). On publication of this paper, your review will be automatically added to Publons, you can then choose whether or not to claim your Publons credit. I understand this statement.

Yes Choose an item.
